# Machine learning with physicochemical relationships: solubility prediction in organic solvents and water

**DOI:** 10.1038/s41467-020-19594-z

**Published:** 2020-11-13

**Authors:** Samuel Boobier, David R. J. Hose, A. John Blacker, Bao N. Nguyen

**Affiliations:** 1 Institute of Process Research & Development, School of Chemistry, University of Leeds, Woodhouse Lane, Leeds, LS2 9JT UK; 2 Chemical Development, Pharmaceutical Technology and Development, Operations, AstraZeneca, Macclesfield, SK10 2NA UK

**Keywords:** Cheminformatics, Computational chemistry, Computational science, Statistics

## Abstract

Solubility prediction remains a critical challenge in drug development, synthetic route and chemical process design, extraction and crystallisation. Here we report a successful approach to solubility prediction in organic solvents and water using a combination of machine learning (ANN, SVM, RF, ExtraTrees, Bagging and GP) and computational chemistry. Rational interpretation of dissolution process into a numerical problem led to a small set of selected descriptors and subsequent predictions which are independent of the applied machine learning method. These models gave significantly more accurate predictions compared to benchmarked open-access and commercial tools, achieving accuracy close to the expected level of noise in training data (LogS ± 0.7). Finally, they reproduced physicochemical relationship between solubility and molecular properties in different solvents, which led to rational approaches to improve the accuracy of each models.

## Abstract (web summary)

Accurate prediction of solubility represents a challenge for traditional computational approaches due to the complex nature of phenomena involved. Here the authors report a successful approach to solubility prediction in organic solvents and water using combination of machine learning and computational chemistry.

## Introduction

Solubility is a critical physical property of organic compounds in drug development, e.g., availability, distribution, metabolism, excretion and toxicity (ADMET)^1,2^, protein engineering^3–5^, chemical process design^6^, synthetic route prediction^7,8^, extraction and crystallisation^9,10^. Due to its importance in environmental predictions, biochemistry, and agrochemical and drug design, aqueous solubility prediction has been the subject of intensive research^11^. Previous solubility prediction approaches include fragment-based semi-empirical methods, *e.g*. general solubility equation^12^, UNIFAC^13^, thermodynamic cycle^14^, thermodynamic parameters, e.g., UNIQUAC^15,16^, Hansen solubility parameters and Hildebrandt solubility parameters^17,18^, different molecular dynamics methods^19–21^, and first principle ab initio calculations (COSMO-RS)^22,23^. More recent developments focused on quantitative structure-activity/property relationship (QSAR/QSPR)^24,25^, through statistical analysis and machine learning techniques^26–28^. Despite these advances, accurate prediction of solubility is still a major scientific challenge, as exemplified by the two solubility challenges issued to the research community in 2008 and 2019^29,30^. This is due to the complex nature of dissolution process, which involves lattice/sublimation energy, solvation energy, ionisation of solute and solution phase interactions. Each of these is a challenging property to predict and can be quite computationally expensive^31^. Statistical and machine learning approaches often employ a large number of descriptors (>100)^32^, which has led to difficulties in interpreting and rationally improving prediction models^33^. Finally, prediction is hindered by the poor quality of experimental solubility data^34^, which are affected by measurement techniques, and purity of solute and solvents.

**Fig. 1:**
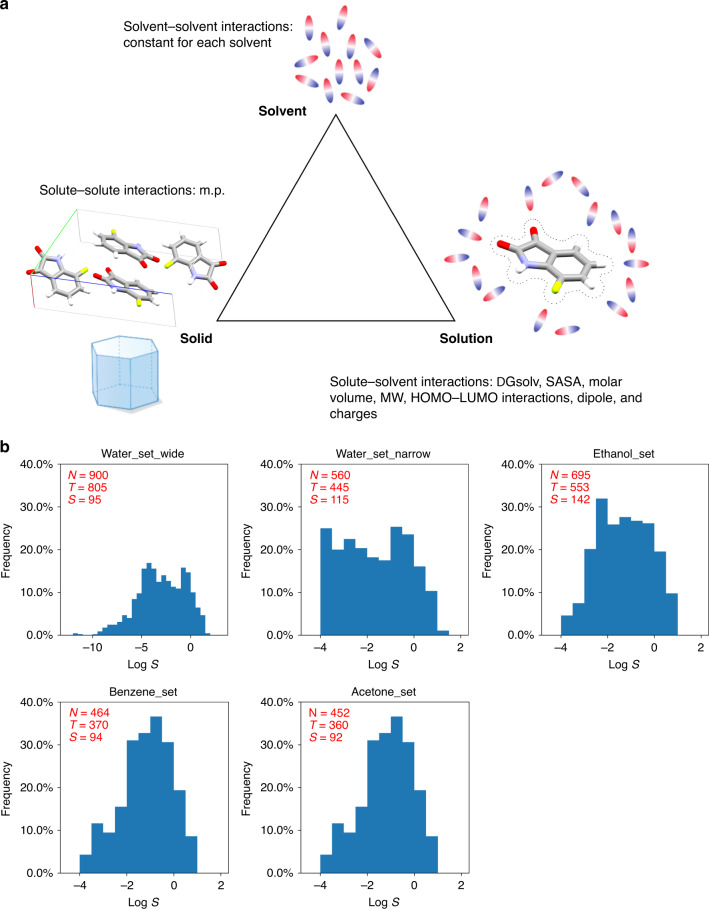
Concepts of solubility prediction and data availability. **a** Physical aspects of dissolution process of solid and corresponding descriptors. **b** Curated solubility datasets for this study and their LogS distributions (*N* *=* number of datapoints, *T* *=* number of datapoints in training set, *S* *=* number of datapoints in test set).

In this paper, we report our new approach to general solubility prediction in organic solvents, which has been understudied, and water using machine learning. In contrast to previous studies, a small number of descriptors (14 in contrast to the usual >100 descriptors employed in QSPR models) were rationally selected based on their relevance to the physicochemical aspects of dissolution process (Fig. 1a). Consequently, interpretable solubility prediction models, which reproduce physicochemical relationships between solubility and molecular properties in different solvents, with excellent accuracy were developed. Furthermore, these models were successfully validated against industrial targets and those of the solubility challenges^29,30^. Finally, our results were benchmarked against the AquaSol model^26^, EPI Suite^TM^ (the official tool of the EPA)^35^, and COSMOtherm as the standard ab initio tool for solubility prediction^36^.

## Results and discussion

### Data curation

Solubility data were collected from Open Notebook Science Challenge aqueous solubility dataset and the Reaxys database. For this study, only solubility data of neutral solutes in single component solvents were collected. While aqueous solubility data are numerous, our search of the Reaxys database resulted in a limited amount of data in organic solvents (Fig. 1b). Thus, ethanol, benzene and acetone were chosen as the solvents in this study to maximise the amount of training data and to cover the entire range of solvent polarity. Although benzene is not a commonly employed solvent in modern chemistry, it represents non-polar solvents with sufficient data availability.

Analysis of LogS values for the collected solubility data showed that while the range for LogS (measured in M) in water is −12 to 2, those in organic solvents are typically in the range of −4 to 1. To provide a consistent comparison, a second aqueous solubility dataset (***Water_set_narrow***, LogS = −4 to 1) was created from the first dataset (***Water_set_wide***). Although an even distribution of LogS values across the range in each dataset is preferable for model training (Fig. 1b), given the limitation on data availability no trimming based on LogS was carried out for the organic solvent datasets (***Ethanol_set***, ***Benzene_set***, and ***Acetone_set***).

Molecular weight (MW) was found to be normally distributed for all datasets, centred on MW = 200 with few above MW = 500 (Supplementary Fig. 4). For this study, compounds with MW > 504 were excluded to keep computational costs reasonable while maintaining their relevance to synthetic intermediates in drug discovery/development^37^. Interestingly, the distributions of organic functional groups are similar between the datasets with the exception of a higher percentage of halides in ***Water_set_wide*** and ***Water_set_narrow*** and a higher percentage of sulphur containing compounds in ***Benzene_set*** (Supplementary Fig. 5). A wide range of functional groups were found including halogen, 3- and 4-membered rings, although B and Si containing compounds, which may be valuable synthetic intermediates, were absent.

Thus, five open-access and curated solubility datasets were created for this study. Three are unique solubility datasets in organic solvents. Each of these was split into a training set and a validation set by LogS binning (Supplementary Note 4.1) and a randomly even distribution of data to ensure the representative nature of the validation set.

Descriptor development: In order to develop interpretable predictive models for solubility in different solvents, a small set of molecular descriptors, which represent solute-solute and solute-solvent interactions, was selected. This small set of descriptors will also benefit the statistical robustness of the models given the relatively small size of the datasets. All 22 descriptors are summarised in Table 1, covering sum of thermal and electronic energies of the solute molecule, solvation energy, orbital interaction between solute and solvent, dipole moment and charge distribution in the solute molecule, molecular volume, Solvent Accessible Surface Area, molecular weight and the number of atoms of the solute. Finally, the experimental melting point was included as a reflection of the sublimation energy of the solid form of the solute. Melting point prediction is still highly inaccurate (RMSE ≈ 38 °C), rendering experimental values a necessity^38^.

**Table 1:** List of descriptors and how they were calculated.

No.	Name^e^	Description	No.	Name^e^	Description
**1**^**e**^	***E0_gas***^a^	Zero-point energy of optimised gas structure (Hartrees)	12	*Solv_dip*^a^	Dipole moment of solution structure (Debye)
**2**	***E0_solv***^a^	Zero-point energy of optimised solution structure (Hartrees)	13	*O_charges*^a^	Sum of charges on solution structure oxygen atoms
**3**	***DeltaE0_sol***^a^	Solvation energy calculated as E0_solv - E0_gas (Hartrees)	14	*C_charges*^a^	Sum of charges on solution structure carbon atoms
**4**	***G_gas***^a^	Gibbs free energy of optimised gas structure (Hartrees)	15	*Most_neg*^a^	Charge on most negative atom of solution structure
5	*G_solv*^a^	Gibbs free energy of optimised solution structure (Hartrees)	16	*Most_pos*^a^	Charge on most positive atom of solution structure
6	*DeltaG_sol*^a^	Solvation energy calculated as G_solv - G_gas (Hartrees)	17	*Het_charges*^a^	Sum of charges on solution structure non-hydrogen/carbon atoms
**7**	***HOMO***^a^	HOMO energy of gas phase structure of the solute (eV)	18	*Volume*^a^	Molar volume (cm^−3^.mol)
**8**	***LUMO***^a^	LUMO energy of gas phase structure the solute (eV)	19	*SASA*^b^	Solvent Accessible Surface Area (Å^2^)
9	*L*_*solu*_*H*_*solv*_^a^	Energy gap between solute LUMO and solvent HOMO (eV)	20	*MW*^c^	Molecular weight (Daltons)
10	*L*_*solv*_*H*_*solu*_^a^	Energy gap between solvent LUMO and solute HOMO (eV)	**21**	***N_atoms***^c^	Number of all atoms in molecule
**11**	***Gas_dip***^a^	Dipole moment of gas structure (Debye)	22	*m.p*.^d^	Experimental melting point (°C)

^a^Gaussian 09 derived descriptors were computed using DFT B3LYP/6-31 + G(d), solution structures were calculated using Polarizable Continuum Model IEFPCM for the solvent; ^b^Derived using PyMOL and molecular structure optimised with Gaussian 09; ^c^Derived with Python; ^d^from Reaxys; ^e^The descriptors *N_atoms*, *E0_gas*, *E0_solv*, *DeltaE0_sol*, *G_gas*, *gas_dip*, *HOMO* and *LUMO* (in bold font) were removed from the descriptor list.

**Fig. 2:**
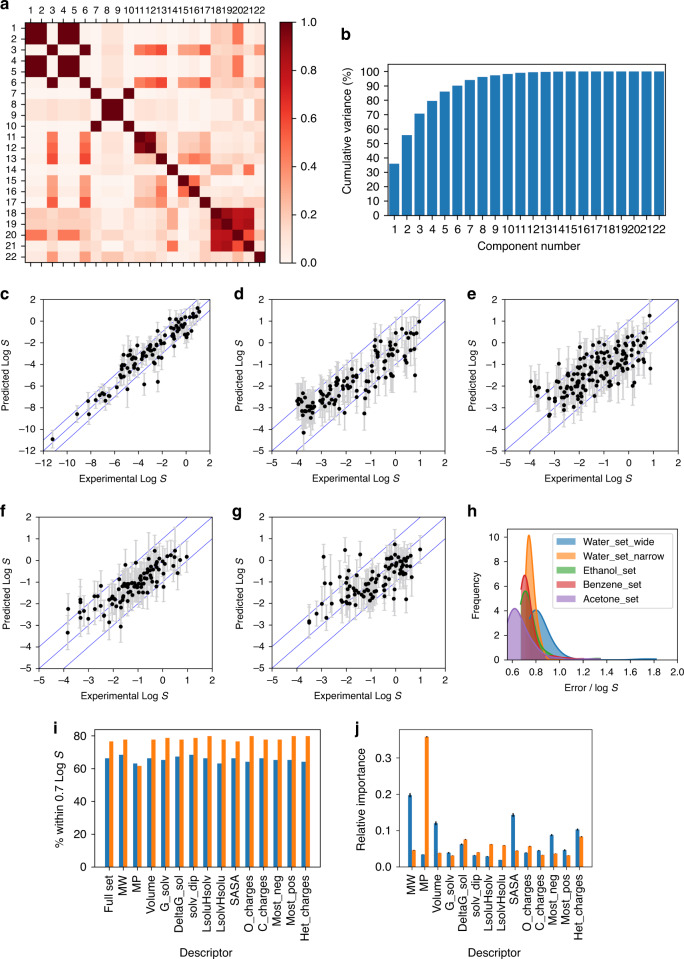
Results of initial machine learning prediction models. **a** Descriptor correlation analysis, **b** principal component analysis of the descriptors with Water_set_wide; and plots of predicted vs experimental LogS, with predicted errors, using GP algorithm for **c** Water_wide_set, **d** Water_narrow_set, **e** Ethanol_set, **f** Benzene_set, **g** Acetone_set; and **h** distributions of predicted errors (1 standard deviation) for each dataset with GP; and **i** impact of the removal of a single descriptor on ET prediction models (blue: Water_set_wide, orange: Benzene_set), **j** feature importance plot for ET prediction models (blue: Water_set_wide, orange: Benzene_set).

Correlation between the calculated descriptors were analysed and summarised in Fig. 2a. The only observed significant correlations were those expected between *E0_gas*, *E0_solv*, *DeltaE0_sol*, *G_gas*, *G_sol* and *DeltaG_sol*, between *gas_dip* and *solv_dip*, and between *HOMO*, *LUMO*, *L*_*solu*_*H*_*solv*_, and *L*_*solv*_*H*_*solu*_. Similarly, the scree plots indicated >10 principal components were needed to capture most of the variations in the descriptors (Fig. 2b). Using an acceptable threshold of correlation R^2^ ≤ 0.9, the descriptors *N_atoms*, *E0_gas*, *E0_solv*, *DeltaE0_sol*, *G_gas*, *gas_dip*, *HOMO* and *LUMO* (Table 1, in bold font) were removed. Consequently, the trimmed down set of 14 descriptors (white background) was taken forward for solubility prediction models.

Metrics for predictive models**:** In order to build and improve predictive models, reliable metrics to evaluate their accuracy and reliability are essential. Common practice in machine learning relies on R^2^ and RMSE to evaluate models. Both these values are highly dependent on the LogS range the model is applied to. Furthermore, despite consistency within in-house small datasets^29^, a typical experimental error of ± 0.5–0.7 for LogS in literature aqueous solubility measurements has been established by Mitchell and Palmer^34^. These are due to variations in pH, temperature and purity of solvents. Such errors in the training set render R^2^ and RMSE less reliable in evaluating solubility predictive models. Consequently, two new metrics were created for our evaluation: % of predictions within LogS ± 0.7 and within LogS ± 1.0 of experimental values (%LogS ± 0.7 and %LogS ± 1.0). The former reflects the maximum accuracy of the model based on the available data and the latter the limits of the usefulness of the model as a guiding tool for process/product development.

Evaluation and interpretation of models: Eight machine learning methods, *i.e*. MLR (Multiple Linear Regression), PLS (Partial Least Square), ANN (Artificial Neural Network), SVM (Support Vector Machine), GP (Gaussian Process), RF (Random Forest), ET (Extra Trees) and Bag (Bagging), were applied to all 5 datasets. Deep Neural Networks were not considered due to the small size of the datasets. Parameters for each model were optimised to maximise accuracy and avoid overfitting (Supplementary Note 4). The optimisation and cross-validation results are included in the Supplementary Notes. The split-test model metrics are summarised in Table 2.

**Table 2:** Table of prediction model metrics using machine learning methods with five datasets^a^.

No.	Dataset	Metric^b^	MLR	PLS	ANN	SVM	GP	RF	ET	Bag	Stdev^c^
1	***Water_set_wide*** (LogS = −12 −2)	R^2^	0.80	0.80	0.90	0.89	0.88	0.90	**0.93**	0.90	0.02
2	***Water_set_wide*** (LogS = −12 −2)	RMSE	1.15	1.16	0.84	0.85	0.89	0.83	**0.71**	0.82	0.06
3	***Water_set_wide*** (LogS = −12 −2)	%LogS±0.7	50.5	51.6	58.9	**71.6**	68.4 (91.6)^e^	60.0	66.3	58.9	5.64
4	***Water_set_wide*** (LogS = −12 −2)	%LogS±1.0	65.2	66.3	78.9	78.9	74.7 (94.7)^e^	75.8	**84.2**	76.8	3.24
5	***Water_set_wide*** (LogS = −4 −1)^d^	R^2^	0.58	0.57	0.73	0.69	0.68	0.69	**0.76**	0.69	0.03
6	***Water_set_wide*** (LogS = −4 −1)^d^	RMSE	1.07	1.08	0.77	0.87	0.86	0.81	**0.69**	0.81	0.07
7	***Water_set_wide*** (LogS = −4 −1)^**d**^	%LogS±0.7	62.1	62.1	67.2	**75.9**	72.4 (93.1)^e^	67.2	72.4	65.5	4.35
8	***Water_set_wide*** (LogS = −4 −1)^**d**^	%LogS±1.0	74.1	72.4	**84.5**	77.6	75.9 (96.6)^e^	81.0	**84.5**	81.0	2.89
9	***Water_set_narrow*** (LogS = −4 −1)	R^2^	0.68	0.68	0.74	**0.76**	**0.76**	0.72	0.75	0.72	0.02
10	***Water_set_narrow*** (LogS = −4 −1)	RMSE	0.82	0.83	0.74	**0.71**	**0.71**	0.76	0.73	0.77	0.03
11	***Water_set_narrow*** (LogS = −4 −1)	%LogS±0.7	61.7	61.7	68.7	65.2	**73.0** (98.3)^e^	60.9	66.1	60.9	3.41
12	***Water_set_narrow*** (LogS = −4 −1)	%LogS±1.0	80.0	80.0	**84.3**	81.7	81.7 (98.3)^e^	82.6	80.9	81.7	1.30
13	***Ethanol_set*** (LogS = −4 −1)	R^2^	0.29	0.29	0.49	0.51	0.51	**0.53**	0.50	0.52	0.02
14	***Ethanol_set*** (LogS = −4 −1)	RMSE	0.98	0.99	0.88	0.81	0.80	**0.79**	0.81	0.80	0.04
15	***Ethanol_set*** (LogS = −4 −1)	%LogS±0.7	50.7	51.4	64.1	64.1	66.2 (93.7)^e^	64.8	62.7	**65.5**	1.04
16	***Ethanol_set*** (LogS = −4 −1)	%LogS±1.0	72.5	71.8	76.8	78.9	77.5 (95.1)^e^	**82.4**	78.9	79.6	2.20
17	***Benzene_set*** (LogS = −4 −1)	R^2^	0.64	0.64	0.67	0.71	0.70	0.72	**0.75**	0.72	0.03
18	***Benzene_set*** (LogS = −4 −1)	RMSE	0.66	0.66	0.63	0.58	0.58	0.57	**0.54**	0.57	0.03
19	***Benzene_set*** (LogS = −4 −1)	%LogS±0.7	75.5	74.5	77.7	76.6	**79.8** (98.9)^e^	76.6	76.6	75.5	0.78
20	***Benzene_set*** (LogS = −4 −1)	%LogS±1.0	86.2	85.1	88.3	89.4	**90.4** (100)^e^	**90.4**	**90.4**	89.4	0.87
21	***Acetone_set*** (LogS = −4 −1)	R^2^	0.36	0.35	**0.42**	**0.42**	0.42	0.40	0.40	0.41	0.01
22	***Acetone_set*** (LogS = −4 −1)	RMSE	0.87	0.87	0.87	**0.83**	**0.83**	0.84	0.84	**0.83**	0.02
23	***Acetone_set*** (LogS = −4 −1)	%LogS±0.7	60.9	62.0	67.4	**72.8**	68.5 (91.3)^e^	62.0	63.0	62.0	4.68
24	***Acetone_set*** (LogS = −4 −1)	%LogS±1.0	78.3	80.4	79.3	81.5	**84.8** (93.5)^d^	80.4	78.2	80.4	1.25

^a^Machine learning methods were applied using *scikit-learnt* and *GPy* packages in Python^49,50^.

^b^The best model for each metric with each dataset is in **bold**.

^c^Standard deviation of the metrics for ANN, SVM, GP, RF, ET and Bag.

^d^Metrics obtained by limiting the evaluation to the LogS = –4 - 1 zone only.

^e^Metrics in brackets are calculated including the entire predicted error range of each predicted solubility.

All four metrics (*R*^2^, RMSE, %LogS ± 0.7, %LogS ± 1.0) clearly showed that linear regression models (MLR and PLS) perform poorly in solubility prediction compared to non-linear models. Importantly, across the five datasets, the performances of the five non-linear models are quite comparable and consistently good. In most cases, the standard deviations between their metrics are very small. The only exceptions are SVM, which gave notably better %LogS ± 0.7 with ***Water_set_wide*** and ***Acetone_set***, and GP with ***Water_set_narrow***. These suggested that the overall accuracy of these predictions is less dependent on the machine learning model and is more dependent on the descriptors and data quality. This is further supported by good agreement (*R*^2^ > 0.9) between individual predictions from each of the six non-linear methods (Supplementary Figs. 45–49). Consistent with this hypothesis, the R^2^ and RMSE metrics for the models for ***Ethanol_set*** and ***Acetone_set*** are much poorer compared to those of ***Water_set_wide***, ***Water_set_narrow*** and ***Benzene_set***, despite little decrease in %LogS ± 0.7 and %LogS ± 1.0. These reflect the quality of experimental solubilities in ethanol and acetone and the poor reliability of R^2^ and RMSE. Both solvents are often contaminated with water and acetone is a volatile solvent (b.p. 56 °C), leading to larger experimental errors in solubility measurements.

The non-linear models coped well with these datasets, with %LogS ± 0.7 = 60-80 and %LogS ± 1.0 = 74-90 for (LogS = −4–1), maintaining their effectiveness as predictive models for novel compounds. The best models were obtained with ***Benzene_set***, with the highest %LogS ± 0.7 = 79.8 and %LogS ± 1.0 = 90.4. When the predicted errors for each solubility by GP was included, very high values of %LogS ± 0.7 > 91.6 and %LogS ± 1.0 > 93.5 were obtained, further supporting our hypothesis that the accuracy of the predictions was limited by the descriptors and training data themselves. The distributions of predicted errors for each prediction using GP are shown in Fig. 2c–g, confirming the inherent errors LogS ± 0.7 in the training data. Finally, there was an expected deterioration of the R^2^ metric, although the other three metrics were improved, moving from ***Water_set_wide*** (LogS = −12–2, with R^2^ value comparable to those achieved by other methods)^28,32,39,40^ to ***Water_set_narrow*** (LogS = −4–1). When the predictions for ***Water_set_wide*** were narrowed to LogS = −4 –1, the obtained metrics are very similar to those of ***Water_set_narrow*** (Table 2, entries 5-8).

Analysis of the outliers in each model using ET algorithm (Supplementary Note 4.9), chosen for its consistent performance with all datasets, showed that they often include acidic and basic functional groups, extended conjugate/aromatic system, azo group, long and flexible carbon chains, or high density of polar functional groups. These are less well presented in the training data. The distribution of LogS of outliers and the BertzCT complexity descriptor for the inliers and outliers (Supplementary Fig. 56)^41^ also indicated that the outliers are on average more complex than the inliers and their LogS values are more likely at the limits of the LogS range, as expected from the uneven distribution LogS values in the datasets.

The interpretability of the models is one of the key aspects of their validation. As the six non-linear methods produced comparable results, the analysis was again carried out for the ET models. The effect of leaving one descriptor out on the model metrics were evaluated for all five datasets (Fig. 2i and Supplementary Figs. 59–61). Similar trends were observed for all 4 metrics: (i) minor changes for ***Water_set_wide*** and ***Water_set_narrow***, and (ii) significant decrease in accuracy with ***Ethanol_set***, ***Benzene_set***, and ***Acetone_set*** when melting points are excluded. This decrease is more pronounced with benzene than with the two polar solvents, ethanol and acetone.

Furthermore, feature importance plots of the 5 ET models showed very high dependence of the models for ***Ethanol_set***, ***Benzene_set***, and ***Acetone_set*** on melting point (Fig. 2j and Supplementary Fig. 62). The models for ***Water_set_wide*** and ***Water_set_narrow*** showed a more even distribution of importance across all the descriptors. In solvents other than benzene, MW, molar volume, SASA, charges on heteroatoms, which are linked to solvent-solute interactions, were also given high importance (Supplementary Figs. 59–62). These analyses showed crucial insights into the factors controlling solubility in the four solvents in this study. Aqueous solubility is dominated by solvation energy and solvent-solute interactions, due to the high polarity of water and its capability for hydrogen bonding^42^. Thus, the importance of melting point as a descriptor is low. In contrast, solubility in organic solvents is dominated by solute-solute interactions in the solid form, *i.e*. sublimation energy. Consequently, the models showed strong dependence on melting point, which is the only descriptor included to explicitly describe the solid state of the solute. As solvent-solute interaction is weaker in benzene, with only Van der Waal forces being available, the impact of removing melting point from the descriptor is more pronounced. Thus, the prediction models showed strong agreement with our understanding of the physical aspects of the dissolution process.

Finally, the 14 descriptors were recalculated using the semi-empirical method PM6 in order to evaluate the impact of the lower computational cost to the accuracy of these models. The %LogS ± 0.7 and %LogS ± 1.0 metrics for PM6 models are similar to those of the DFT models with a few exceptions for datasets with LogS = –1 to 4 (Supplementary Table 23), with the exception of the models for ***Water_set_wide***. With the highest quality dataset ***Benzene_set***, all metrics for PM6 and DFT models are nearly identical. The total CPU time for PM6 calculation of descriptors of 394 compounds is 219 hours, compared to 5458 hours for DFT descriptors.

Improvement of the models: While Fig. 2h indicated that the accuracy of our predictions is close to that of the training data, the values for %LogS ± 0.7 can still be improved. Based on our hypothesis that the predictions are more dependent on the descriptors than on the machine learning method, those which have the highest impacts were considered for improvement. SASA depends on the size of the probe and the conformer being measured, but the variation is small. MW, molar volume, and m.p. are fixed for each molecule, leaving charge descriptors and solvation energy (for ***Water_set_wide*** and ***Water_set_narrow***). Thus, four methods were evaluated to rationally improve the models: (i) by inclusion of conformers; (ii) by inclusion of descriptors for the molecular charge surface; (iii) by using more accurate calculation of the solvation energy (in water only) and (iv) by consensus of predictions.

Inclusion of conformers (PM6, descriptors averaged by population) did not result in any significant improvement to the model metrics (Supplementary Table 25). Boltzmann distribution based on the free energy of conformers indicated that most molecules have one stable conformer which accounts for more than 90% of the population, negating the potential benefit this approach. Descriptors for the charge isosurface (95% of the electron density, Supplementary Note 2.2.3) were included with the original 14. The only strong correlations within this new set of 27 descriptors were between Area2, Area3 and SASA as expected. While some improvements were observed with the metrics of the models for ***Acetone_set*** (Supplementary Table 17), the new models generally gave similar results with much larger computational cost.

Jensen and co-workers recently demonstrated that HF/SMD (Solvation Model Density) give more accurate aqueous solvation energy than other methods, *e.g*. IEFPCM and COSMO^43^. Thus, we recalculated *G_solv* and *DeltaG_sol* using the HF/SMD method and used these new descriptors to rebuild prediction models for ***Water_set_wide*** and ***Water_set_narrow*** (Table 3). Significant improvements to %LogS ± 0.7 and %LogS ± 1.0 were observed with ***Water_set_wide*** for all six machine learning methods. Notably, %LogS ± 0.7 increased 9.5% with ANN and 7.4% with Bag. For most models, approximately 70% of the predictions are within LogS ± 0.7, as accurate as the training data. The improvements obtained with ***Water_set_narrow*** were less significant, but the metrics are consistently better than those obtained with DFT/PCM method.

**Table 3:** Model metrics for Water_set_wide and Water_set_narrow using HF/SMD descriptors.

Dataset	Method	%LogS ± 0.7^a^	%LogS ± 1.0^a^
*Water_set_wide*	ANN	68.4 (+9.5)	84.2 (+5.3)
*Water_set_wide*	SVM	72.6 (+1.1)	83.2 (+4.2)
*Water_set_wide*	ET	69.5 (+3.2)	84.2 (+0.0)
*Water_set_wide*	GP	70.5 (+2.1)	82.1 (+8.4)
*Water_set_narrow*	ANN	70.4 (+1.7)	82.6 (−1.7)
*Water_set_narrow*	SVM	68.7 (+3.5)	85.2 (+3.5)
*Water_set_narrow*	ET	67.0 (+0.9)	81.7 (+0.9)
*Water_set_narrow*	GP	73.9 (+0.9)	81.7 (+0.0)

^a^The changes compared to those obtained using DFT/PCM descriptors are in brackets.

Finally, the similarity between predictions from different models (Supplementary Figs. 45–49) suggests that the few wrong predictions can be compensated through a wisdom-of-crowd approach^44^. Consequently, the consensus predictions were carried out for each compound in the validation set as the average and median of the predictions using ANN, SVM, GP and ET. The results are summarised in Supplementary Table 20. The predictions for all narrow datasets (LogS = -4 to 1) showed improved metrics compared to those of ET models. The consensus mean predictions are slightly better than the consensus median predictions, consistent with our assessment that the predictions from all four methods are very similar, with few outliers. Furthermore, the wrong predictions are not too different from the experimental LogS values, negating the benefit of median over mean. The best performance was observed with ***Benzene_set***, with 82.0% of the predicted solubilities inside LogS ± 0.7 and 90.4% inside LogS ± 1.0 (Supplementary Figs. 66 and 67).

**Fig. 3:**
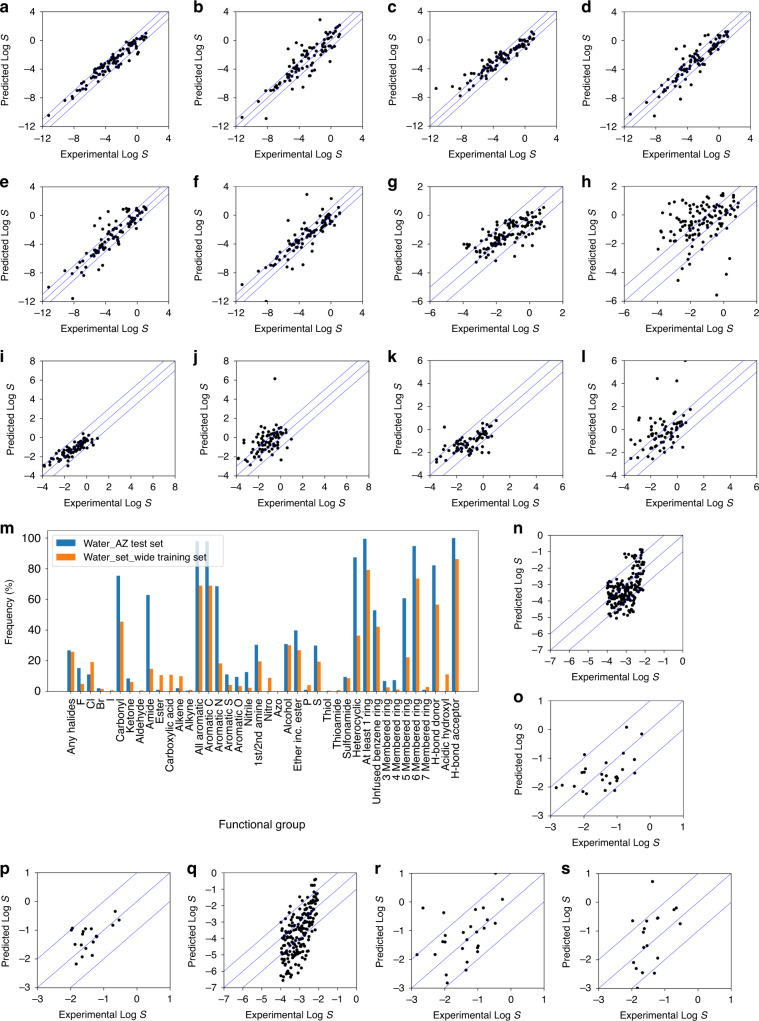
Benchmarking results against other predictive models. Predicted vs experimental LogS for ***Water_set_wide*** **a** ET model; **b** GSE model; **c** AquaSol model; **d** EPI Suite 1 model, **e** EPI Suite 2 model; **f** COSMOtherm calculations; for ***Ethanol_set*** **g**, ET model; **h** COSMOtherm calculations; for ***Benzene_set*** **i** ET model; **j** COSMOtherm calculations; for ***Acetone_set*** **k** ET model; **l** COSMOtherm calculations; and prediction results using datasets from AstraZeneca **m** functional group distribution analysis for dataset from AstraZeneca and ***Water_set_wide***; predicted vs experimental LogS for **n** ET model for ***AZ_water*** (without m.p.); **o** ET model for ***AZ_ethanol*** (without m.p.); **p** ET model for ***AZ_acetone*** (without m.p.); **q** COSMOtherm calculations for ***AZ_water***; **r** COSMOtherm calculations for ***AZ_ethanol***; and **s** COSMOtherm calculations for ***AZ_acetone***.

Benchmarking and external datasets: Our models were compared with standard prediction tools used in academia and industry, employing the same evaluation datasets. For aqueous solubility, AquaSol, which was developed based on undirected graph recursive neural networks^26^, gave less accurate predictions than our ET model, particularly at lower LogS values. EPI Suite, a fragment-based tool^35^, performed even more poorly as expected. Similarly poor results were obtained with COSMOtherm by COSMOlogic^45,46^. For solubilities in ethanol, benzene and acetone, COSMOtherm predictions were compared with our ET models. In all three cases, COSMOtherm produced significantly larger errors in its predictions, with multiple outliers. The results are summarised in Fig. 3.

While our models performed better than the established tools, a more rigorous test should be an application of the models to new unrelated test sets. For this purpose, the solubilities of three sets of compounds from AstraZeneca (in water, ethanol and acetone, without m.p. for a fair comparison against COSMOtherm) were evaluated against their measured values (Fig. 3n–p). As benchmarks, COSMOtherm was employed to predict solubilities for the same compounds and the results are shown in (Fig. 3q–s). The accuracy of water solubility predictions using our ET model decreased compared to those of the validation set, consistent with the increased in complexity and higher frequency of functional groups in these compounds (Fig. 3m). However, predictions made by COSMOtherm are much less accurate than ours in all three solvents. Importantly, all predictions made by ET models in ethanol and acetone (when m.p. is included, see Supplementary Table 32) were within LogS ± 1.0, albeit with small test sets.

In conclusion, we report the development, evaluation and improvement of interpretable solubility prediction models in organic solvents and water based on judicious interpretation of the dissolution phenomenon into numerical representations through physicochemical relationships. This approach, which we named Causal Structure-Property Relationship (CSPR), enabled the use of a small set of carefully selected descriptors and smaller training datasets compared to models which employ deep neural networks. Our models gave significantly more accurate predictions compared to benchmarked open-access and commercial tools, achieving accuracy close to the expected level of noise in training data (LogS  ±  0.7). Importantly, they reproduced physicochemical relationship between solubility and molecular properties in different solvents, which led to rational approaches to improve the accuracy of each models.

### Methods

Solubility data in water and ethanol were taken from Open Notebook Science Challenge aqueous solubility dataset. Further solubility data in ethanol and other solvents were mined from the Reaxys database. Solubilities measured at temperature specified outside the 14-30 °C range were discarded. Each compound was identified by its InChIKey and analysed using SMILES code. Where multiple measurements were acquired for a molecule, obvious outliers (LogS ± 1.0 from 2 or more measurements) and polymorphs were excluded and the median value of the remaining measurements was taken. For this study, only solubility data of neutral solutes in single component solvents were collected. Melting points were collected from Reaxys and ChemSpider databases. Initial 3D coordinates were generated with CIRpy^47^. Molecules were optimised in gas phase with B3LYP/6-31 + G(d) method using Gaussian 09^48^. The solution phase optimisation was carried out with an implicit polarisable continuum solvent model (IEFPCM) or solvation model based on electron density (SMD), pre-parametrised for each solvent.

Initial 3D structures were generated with Corina software and then optimised at BP-TZVPD-FINE DFT level in COSMOConf^45^ to create the requisite input files for COSMOtherm. COSMOtherm was used to calculate the solubility, where the sublimation energy was estimated via the inbuilt QSPR protocol instead of reference solubility data.

For machine learning, data was pre-processed by scaling descriptors to between 0 and 1, using the Python/*scikit-learn* standard scaler protocol. MLR, PLS, ANN, SVM, RF, ET, and BG were performed using *scikit-learn*. GP models were built using GPy platform with error bars obtained to 1 standard deviation by finding the upper and lower limits for the predictions which encompassed 68% of the prediction distribution. In all cases, radial basis function (rbf) was the best kernel. For correlation between descriptors, Pearson’s R^2^ was calculated pairwise for each descriptor combination using *scipy* python module. These were plotted in 2×2 matrices as heat maps.

## Supplementary information

Supplementary Information

## Data Availability

The datasets from open literature, including calculated descriptors, in this manuscript can be downloaded from this link: https://doi.org/10.5281/zenodo.3686212 Citations should refer directly to this manuscript.

## Code availability

Relevant Python code are included in Supplementary Note 7: Python code examples.
