# Supplementary material for: Machine learning with physicochemical relationships: solubility prediction in organic solvents and water
Source: Nat Commun. 2020 Nov 13;11:5753. doi: 10.1038/s41467-020-19594-z (PMC7666209; doi:10.1038/s41467-020-19594-z)
Supplement: Supplementary file 1 — Supplementary Information [file 41467_2020_19594_MOESM1_ESM.pdf]

Machine Learning with Physicochemical Relationships: Solubility Prediction in Organic Solvents and Water

Samuel Boobier, David R. J. Hose, A. John Blacker, Bao N. Nguyen*

Supplementary Information

Table of contents

Supplementary Note 1: Data mining and curation 4
1.1 Data availability 4
1.2 Solvent choice 4
1.3 Data extraction 5
1.4 Curation and analysis of datasets 6
Supplemtary Note 2: Calculation of Descriptors 12
2.1 Overview 12
2.2 Computational methods 14
Supplementary Note 3: Analysis of descriptors 17
3.1 Descriptor space analysis 17
3.2 Descriptors correlation analysis 21
Supplementary Note 4: Machine learning methodology 26
4.1 Creating training and test sets 26
4.2 Machine learning protocols 26
4.3 Model evaluation 27
4.4 Optimisation of machine learning methods 27
4.5 Impact of skipping more than 1 descriptor 41
4.6 Initial solubility prediction models 43
4.7 Effect of solubility range 58
4.8 Refinement of prediction models 65
4.9 Analysis of outliers 65
4.10 Improvement of descriptors 92
4.11 Consensus predictions 111
4.12 Rapid models with PM6 descriptors 115
4.13 Inclusion of conformation 121
Supplementary Note 5: Benchmarking 124
5.1 AquaSol & EPI Suite 124
5.2 COSMO-RS 125
5.3 General Solubility Equation (GSE) 137
Supplementary Note 6: Validation with external data 139
6.1 Solubility challenge 139
6.2 AstraZeneca datasets 147
Supplementary Note 7: Example Python codes 157
7.1 Filtering out molecules with disallowed elements and mixtures 157
7.2 Melting point data from ChemSpider 160

7.3 Obtain Molecular Weight using CIRpy 164
7.4 Functional group analysis using OpenBabel 165
7.5 Generating input structures with molecular mechanics and CIRpy 167
7.6 Calculating SASA using pymol 169
7.7 Creating shadow projections descriptors 170
7.8 Charge surface descriptors 180
7.9 Correlation Analysis 189
7.10 Evaluation of metrics 190
7.11 Descriptor importance in ET 191
Supplementary References 195

Supplementary Note 1: Data mining and curation

1.1 Data availability

Solubility data in water and ethanol were taken from Open Notebook Science Challenge aqueous solubility dataset.[1] Further solubility data in ethanol and other solvents were mined from the Reaxys database.[2] For solvents other than water and ethanol, there are less than 1200 data points for each solvent (**Figure 1**). Data were collected for compounds with 84 < MW ≤ 504 and in one structural fragment. Further curation to remove duplicates with poor consistency (e.g. ΔLogS > 1), charged species/metal complexes and data points without units further reduced the number of available data points.

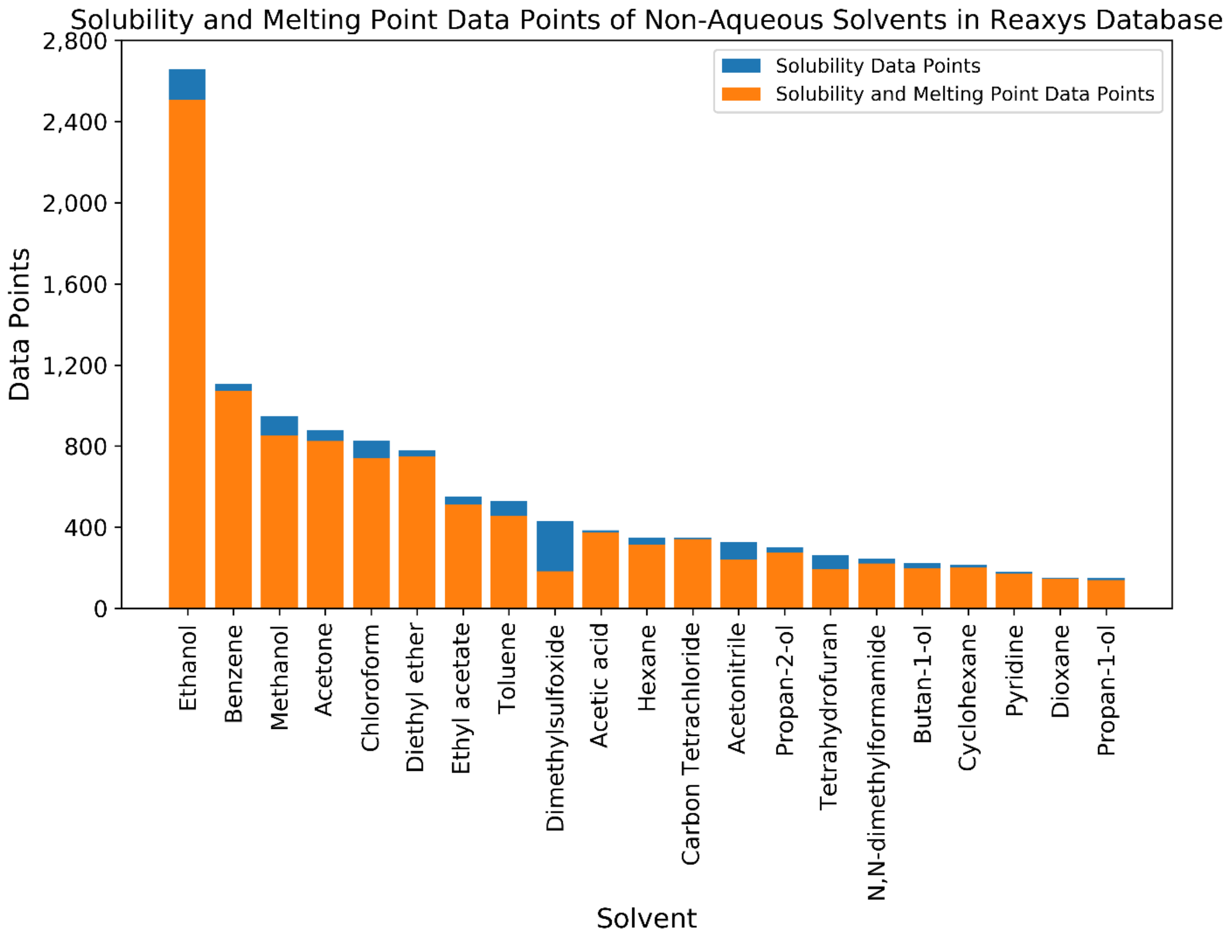

Figure 1. Available data for solubility and melting point in Reaxys Database

1.2 Solvent choice

Datasets in four solvents, i.e. water, ethanol, benzene and acetone, were created. These solvents were chosen for two reasons: (i) data availability; and (ii) a wide a range of solvent polarity and protic/aprotic nature. A comparison of the solvents is summarised in **Table 1**.

Table 1. Comparison between the four solvents in this study

Solvent	Boiling Point (°C)	Dielectric Constant, ε	Vapour Pressure (hPa at 20 °C)	Protic or aprotic?	Polar or non-polar?
Water	100	80.1	17.5	Protic	Polar
Ethanol	78	24.5	59	Protic	Polar
Benzene	80	2.27	101	Aprotic	Non-polar
Acetone	56	20.7	240	Aprotic	Polar

1.3 Data extraction

Solubility data, melting point data, SMILES codes and StdInChIKey codes (unique identifier) was collected for each molecule. Units were converted to LogS = $\log_{10}$C (C = concentration in mol/L) for solubility and °C for melting point. OpenBabel Python module was used to filter out disallowed functionality as molecules were only allowed the following elements: H, C, N, O, F, P, S, Cl, Br and I (see section 7). Data was extracted from Reaxys using its advanced search tool (including SMILES codes and StdInChIKey codes). Melting point data from ChemSpider was obtained using its Python interface (see section 7.2).[3] ONS Challenge is an existing database, which provided solubility data and SMILES codes. Missing StdInChIKey data was found by searching Chemical Identifier Resolver (CIR)[4] and PubChem databases.[5] The sources of data were summarised in **Table 2**.

Table 2. Sources of data used in this work

Dataset	Solubility Data (number of data points)	Melting Point Data (number of data points)
Water_set_wide	ONS Challenge (900)	ChemSpider (900)
Water_set_narrow	ONS Challenge (560)	ChemSpider (560)
Ethanol_set	ONSChallenge (56); Reaxys (639)	ChemSpider (56); Reaxys (639)
Benzene_set	Reaxys (464)	Reaxys (464)
Acetone_set	Reaxys (452)	Reaxys (452)

1.4 Curation and analysis of datasets

Molecules were also removed if they were charged or radicals. Data points with missing units were also removed. If there were more than one data point for a molecule, a median value was taken. Those with more than one data points and ΔLogS > 1, a decision was made on whether a data points was incorrectly recorded (e.g. LogS > 2). If no clear erroneous value can be removed, the molecule was discarded. This process is summarised in **Figure 2**.

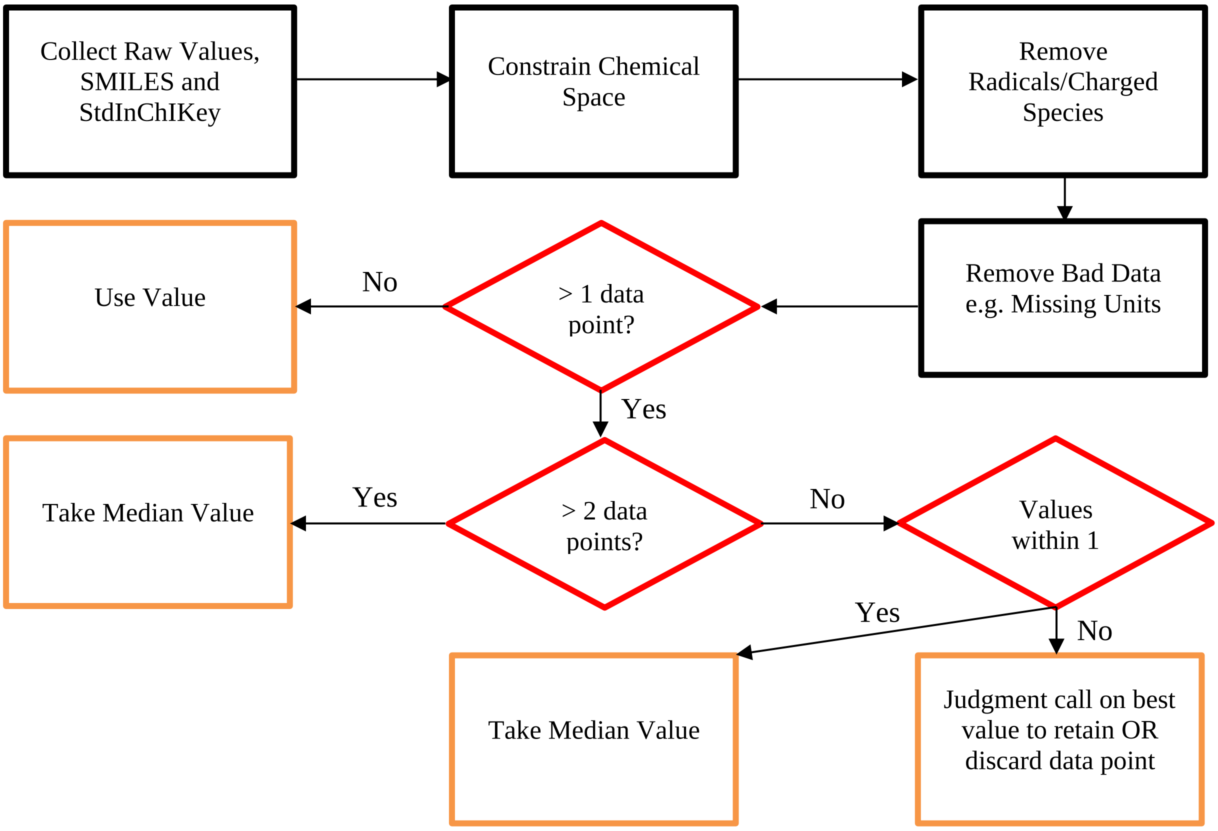

Figure 2. Protocol data collection and curation in this work

1.4.1 LogS range

Analysis of the ***Water_set_wide*** showed a range of LogS from -12 to 2. Non-aqueous solvent sets (***Ethanol_set***, ***Benzene_set*** and ***Acetone_set***) has a much narrower range of LogS from -4 to 1. Thus, a second dataset was created for water in this range (***Water_set_narrow***) to enable comparison between solubility models in different solvents. This is shown in **Figure 3**.

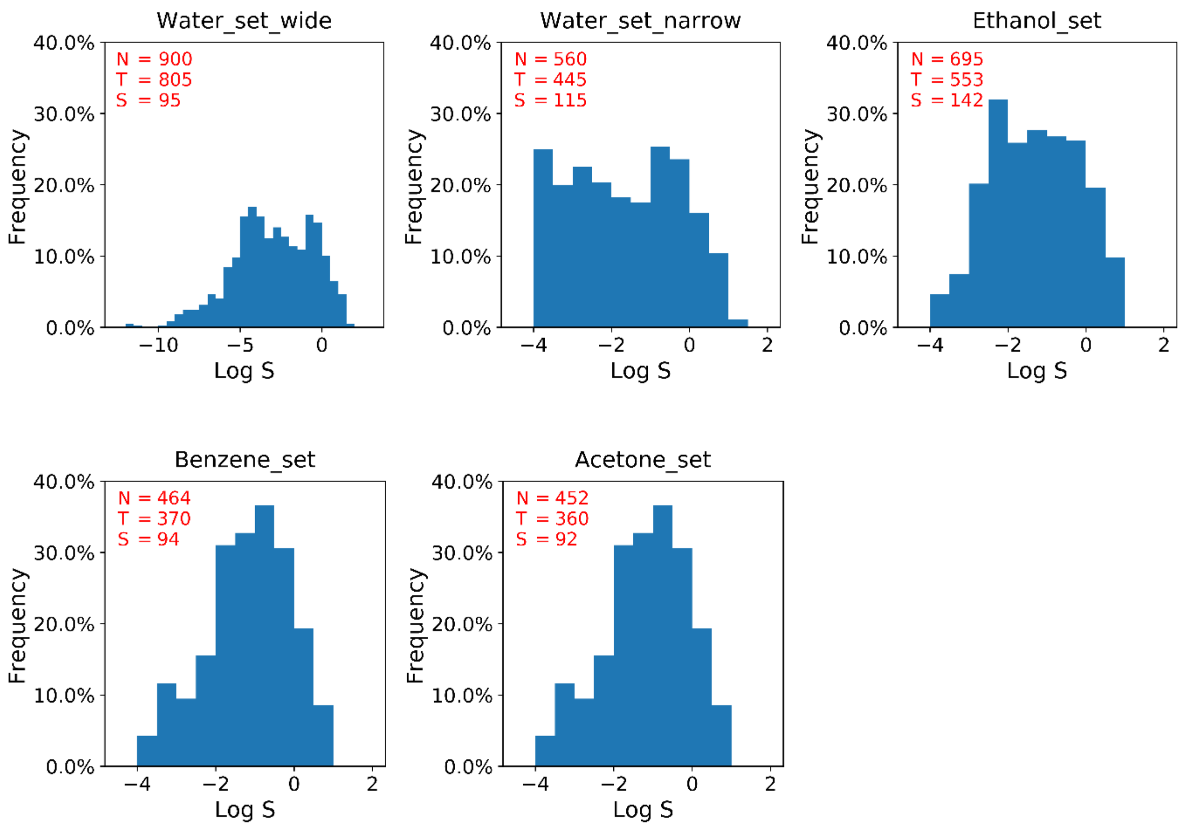

Figure 3. Solubility ranges of the datasets, N = number of datapoints, T = size of training set, S = size of test set.

1.4.2 Molecular weight

Molecular weights were calculated from the corresponding SMILES code using Chemical Identity Resolver Python interface, CIRpy, (see section 7.3). Molecular weight was confined to ≤ 504. This was to conform to Lipinski's rule,[6] an observation that most active pharmaceutical products are small organic molecules. This also keeps computational times reasonable. This is shown in **Figure 4**.

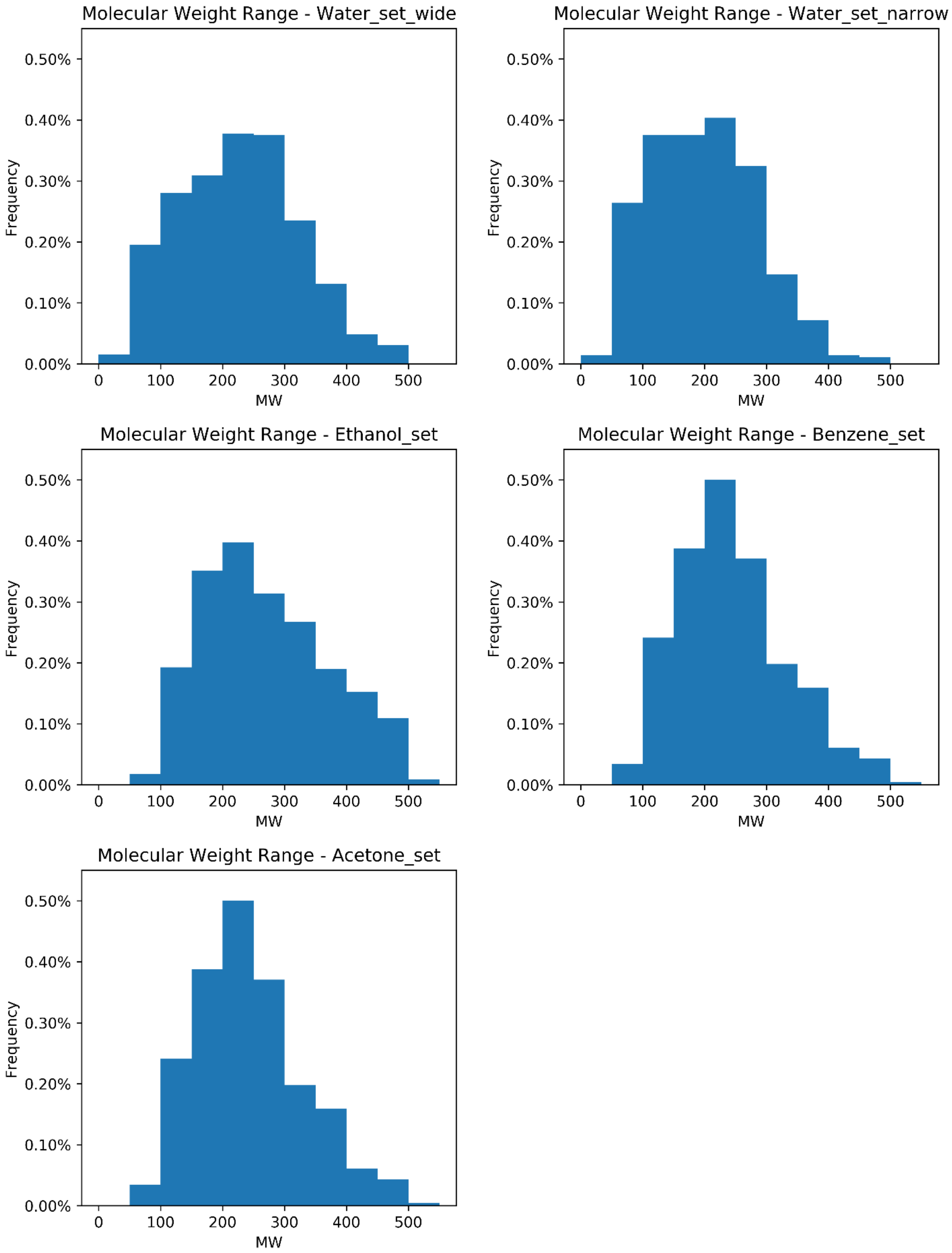

Figure 4. Molecular weight distribution of datasets

1.4.3 Functional group distribution

Functional groups were determined by matching their SMARTS codes to the SMILES strings using pybel/OpenBabel Python module (see section 7.4).[7,8] **Figure 5** shows the functional group distribution for the datasets. Aromatic groups are split into "all aromatic" (contains at least one aromatic atom, as

designated by the SMILES codes) and molecules that contain at least one specific aromatic atom, e.g. “aromatic N”.

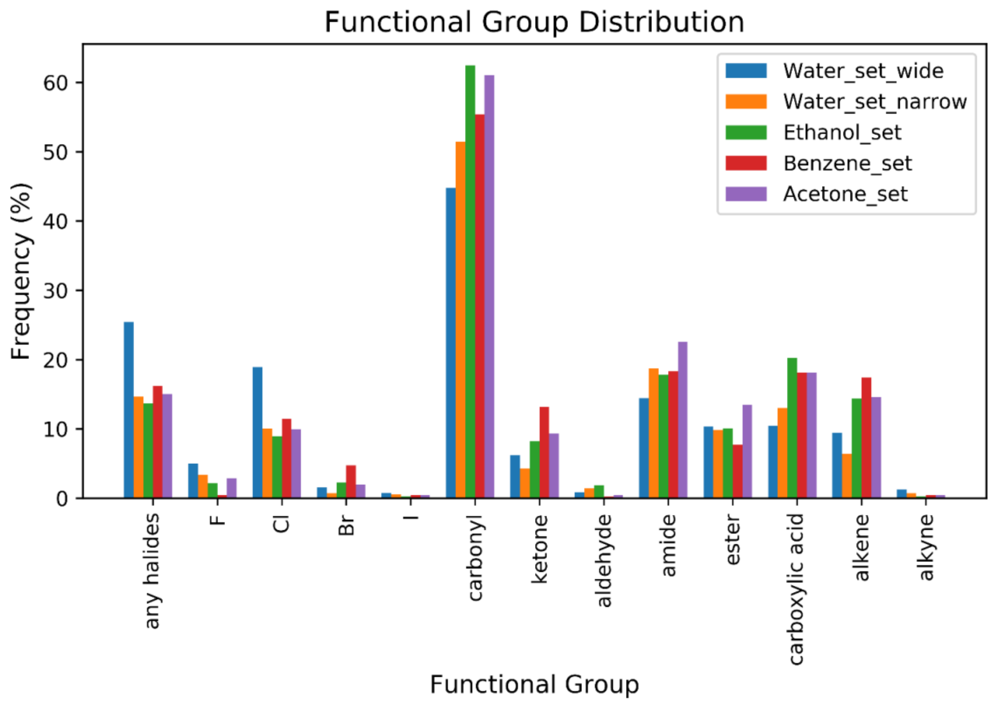
Functional Group Distribution
Water_set_wide
Water_set_narrow
Ethanol_set
Benzene_set
Acetone_set
Frequency (%)
0
10
20
30
40
50
60
any halides
F
Cl
Br
I
carbonyl
ketone
aldehyde
amide
ester
carboxylic acid
alkene
alkyne
Functional Group

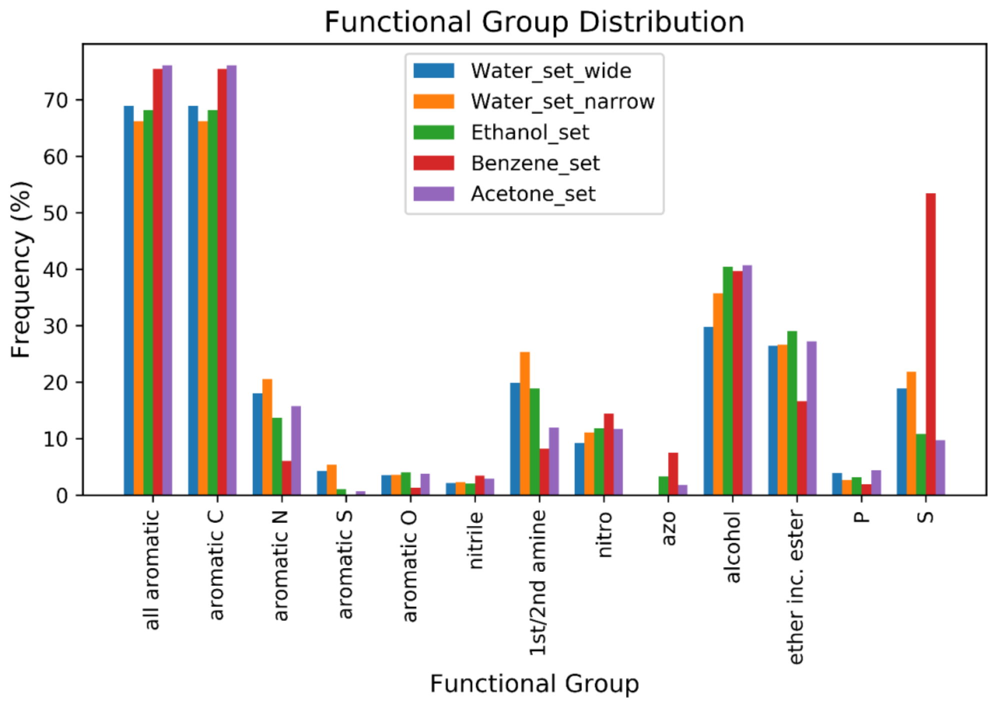
Functional Group Distribution
Water_set_wide
Water_set_narrow
Ethanol_set
Benzene_set
Acetone_set
Frequency (%)
0
10
20
30
40
50
60
70
all aromatic
aromatic C
aromatic N
aromatic S
aromatic O
nitrile
1st/2nd amine
nitro
azo
alcohol
ether inc. ester
P
S
Functional Group

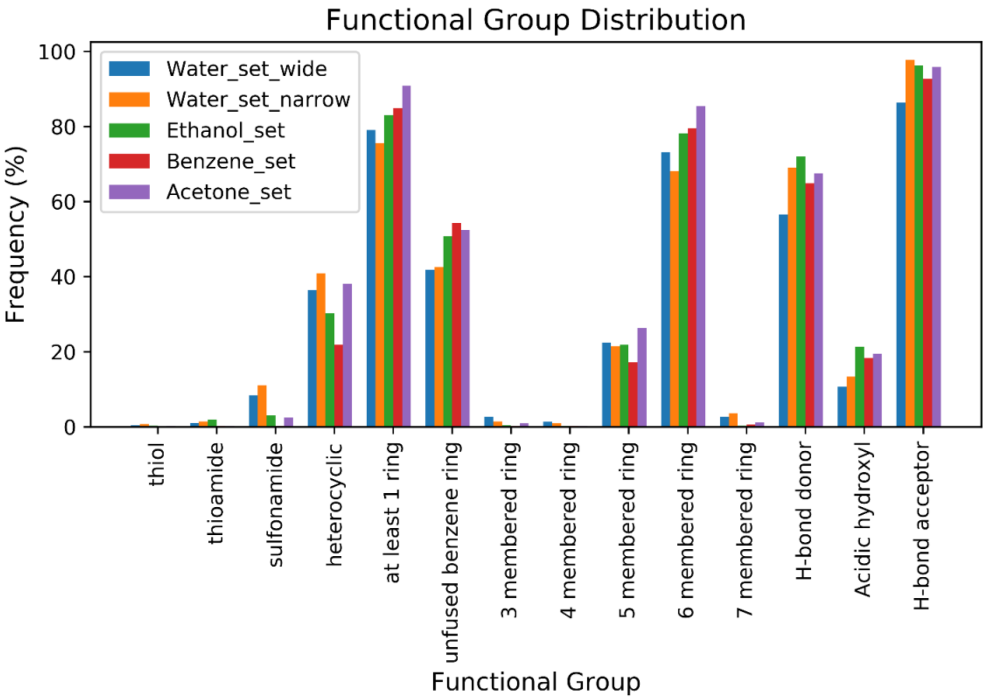

Figure 5. Functional group distribution of the datasets

Supplemtary Note 2: Calculation of Descriptors

2.1 Overview

Table 3 shows the descriptors used in this work. The first 22 descriptors, shaded in green, were considered to build the initial models. The other descriptors, shaded in orange, were evaluated in the later stage of the study.

Table 3. Descriptors in this study

No.	Name	Source	Description
1	E0_gas	G09	Zero-point energy of optimised gas structure (Hartrees)
2	E0_solv	G09	Zero-point energy of optimised solution structure (Hartrees)
3	DeltaE0_sol	G09	Solvation energy calculated as E0_solv - E0_gas (Hartrees)
4	G_gas	G09	Gibbs free energy of optimised gas structure (Hartrees)
5	G_solv	G09	Gibbs free energy of optimised solution structure (Hartrees)
6	DeltaG_sol	G09	Solvation energy calculated as G_solv - G_gas (Hartrees)
7	HOMO	G09	Energy of the HOMO of gas phase structure of the solute (eV)
8	LUMO	G09	Energy of the LUMO of gas phase structure the solute (eV)
9	$L_{solu}H_{solv}$	G09	The gap in energy between the LUMO of the solute and the HOMO of the solvent (eV)
10	$L_{solv}H_{solu}$	G09	The gap in energy between LUMO of the solvent and HOMO of the solute (eV)
11	Gas_dip	G09	Dipole of gas structure (Debye)
12	Solv_dip	G09	Dipole of solution structure (Debye)
13	O_charges	G09	Sum of charges on solution structure oxygen atoms (NPA)
14	C_charges	G09	Sum of charges on solution structure carbon atoms (NPA)
15	Most_neg	G09	Charge on most negative atom of solution structure (NPA)
16	Most_pos	G09	Charge on most positive atom of solution structure (NPA)
17	Het_charges	G09	Sum of charges on solution structure non-hydrogen/carbon atoms (NPA)
18	Volume	G09	Molar volume (cm^{-3}.mol)

19	SASA	G09/Pymol	Solvent Accessible Surface Area (Å^2)
20	MW	Python	Molecular weight (Daltons)
21	N_atoms	Python	Number of all atoms in molecule
22	m.p.	Reaxys/ ChemSpider	Experimental melting point (°C)
23	No_regions	G09/multiwfn	Number of charged regions (threshold ±0.02)
24	Tot_charge	G09/multiwfn	Sum of all charges
25	Neg_charge	G09/multiwfn	Sum of negative charges
26	Pos_charge	G09/multiwfn	Sum of positive charges
27	Big_area	G09/multiwfn	Area of biggest region of charge (pixels)
28	Big_charge	G09/multiwfn	Mean charge of biggest region of charge (pixels)
29	Big_std	G09/multiwfn	Standard deviation (spread) of biggest region of charge (pixels)
30	Area1	G09/OpenCV	Area of smallest shadow projection (pixels)
31	Area2	G09/OpenCV	Area of second smallest shadow projection (perpendicular to first) (pixels)
32	Area3	G09/OpenCV	Area of largest shadow projection (perpendicular to first and second) (pixels)
33	Asp1	G09/OpenCV	Aspect ratio of smallest shadow projection
34	Asp2	G09/OpenCV	Aspect ratio of second smallest shadow projection (perpendicular to first)
35	Asp3	G09/OpenCV	Aspect ratio of largest shadow projection (perpendicular to first and second)

The general flowchart for calculation of descriptors is shown in **Figure 6**. Details of the process are described below.

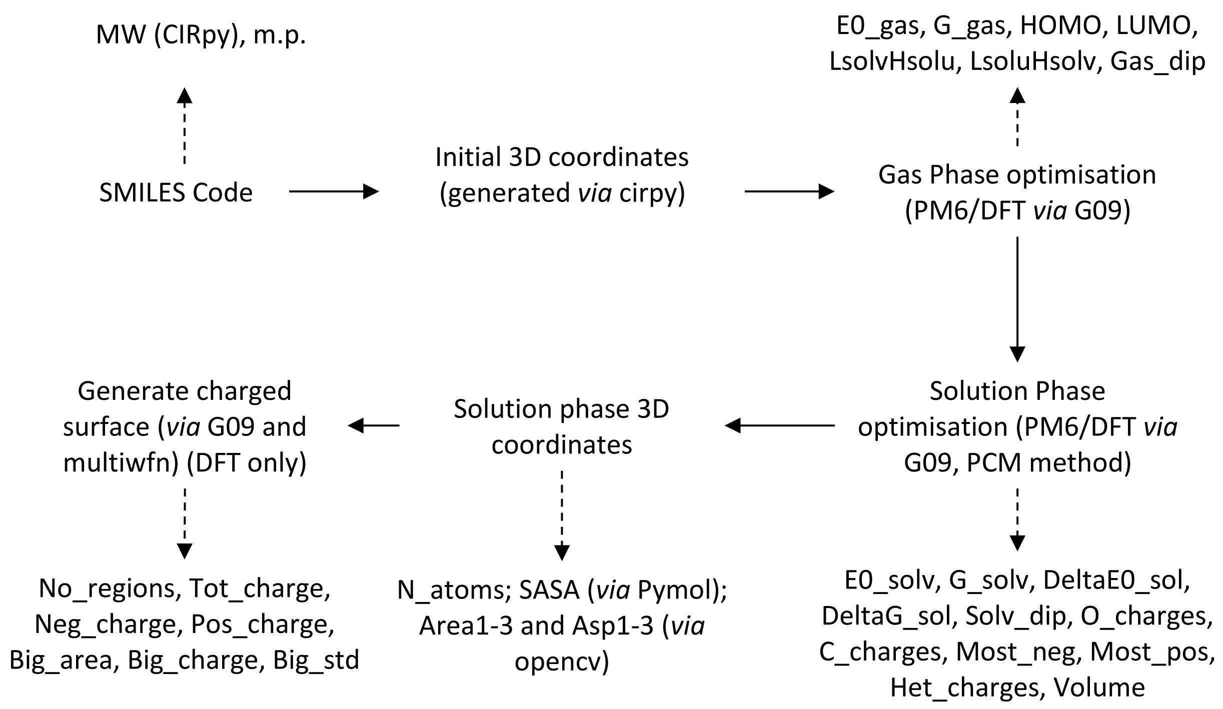

Figure 6. Flowchart for descriptors calculation

2.2 Computational methods

2.2.1 General methods

SMILES codes were provided with each solubility data point. Melting point (m.p.) was obtained from ChemSpider if not provided by Reaxys. Molecular weight (MW) was calculated from the SMILES codes using Chemical Identity Resolver Python interface, CIRpy (see section 7.3). Initial 3D structures were generated with MM using CIRpy Python modules (see section 7.5). These structures were then transferred to University of Leeds Advanced Research Computing (ARC) resource and optimised in gas and solution using Gaussian 09.[9] Both B3LYP/6-31+G(d) and PM6 methods were used with an implicit solvation model, using PCM protocols. From these optimised structures: number of atoms; charges; orbital energies; dipole; molar volume; zero-point energies; and Gibbs energies were calculated. For DFT calculations, charges were obtained using NPA (Natural Population Analysis). For PM6 calculations, Mulliken charges were used. Pymol was used to calculate SASA (Solvent Accessible Surface Area) using the Cartesian coordinates of the optimised solution structures,[10] with a solvent ratio of 1.4 Å (see section 7.6).

2.2.2 Descriptors for size and shape of the molecule

Six descriptors were obtained from the projection of the molecular surface to create a shadow from three perpendicular angles. A protocol was devised where random points on spheres of Van der Waal radius were generated.[11] These points were increased in size to create solid black images, which were saved as high-resolution images. These images were analysed for area and aspect ratio (where Aspect

Ratio = length/width to give number < 0) with OpenCV Python module.[12] Areas were calculated in pixels, since all images were saved at the same relative size. This is summarised in **Figure 7** and codes and more details are given in section 7.7.

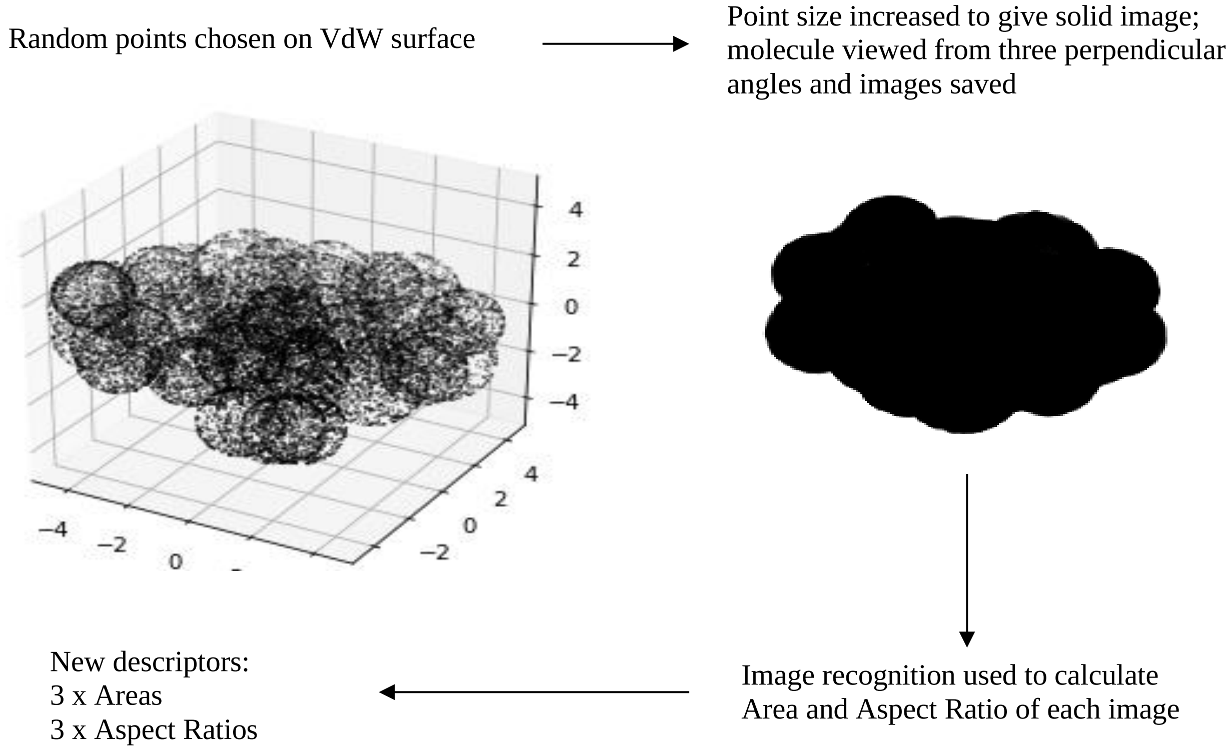

Figure 7. Protocol to generate molecular size and shape descriptors

2.2.3 Charged Surface Descriptors

Multiwfn was used generate points on an isosurface encompassing 95 % of the electron density from the previously optimised structures. These points were feed back into Gaussian 09 and multiwfn to generate the final charges on the isosurface.[13] The descriptors were generated *via* standard clustering techniques in Python. Neural charged points were removed (between -0.02 and 0.02; chosen from various thresholds to give the highest variation in the resulting descriptors) and the clusters found. Descriptors were based on the number of clusters, the total charge, the total negative and positive charges and the size, charge and charge standard deviation of the largest cluster. Sums of charges

weighted each point equally, assuming that each point represented an equal area on the isosurface. This is shown in **Figure 8** and elaborated on in section 7.8.

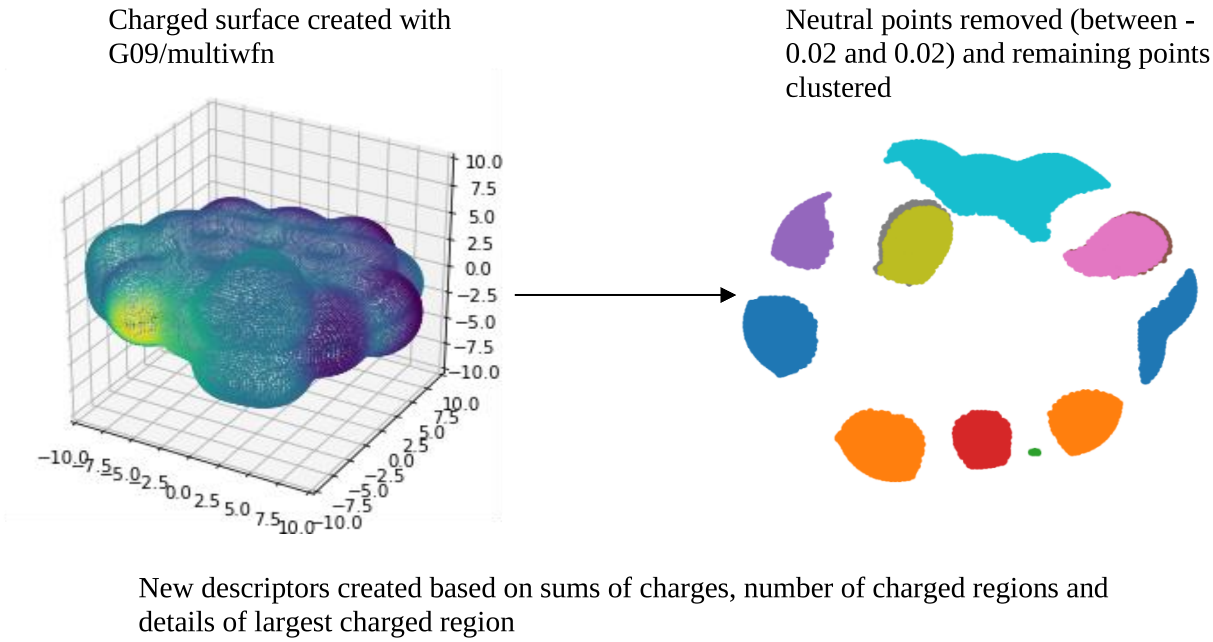

Figure 8. Protocol for generate surface charge descriptors, a charged surface is created with Gaussian 09/multiwfn and then the neutral points removed.

Supplementary Note 3: Analysis of descriptors

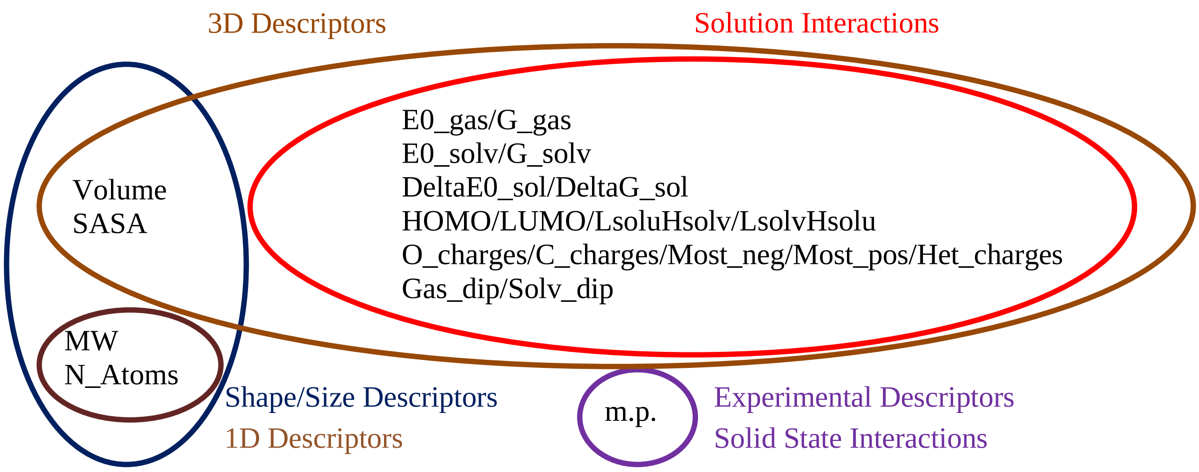

Figure 9. Different types of descriptors in this project and what they represent

3.1 Descriptor space analysis

Principal Component Analysis (PCA) was used to visualise the descriptor space. PCA is a dimension reducing method. This allows a visual representation of the potential predictive power of 50-60 % of the data by plotting the first two principal components. It also shows how many descriptors are required to describe a certain amount of the variance in the dataset, i.e. are there many correlated/redundant descriptors in the descriptor set. The PCA plots show that there is a relationship between the descriptors and water, especially in ***Water_set_side*** dataset. The scree plots show that after 14 components, most of the variance in the dataset has been described. This is shown in **Figure 10**, **Figure 11** and **Figure 12**.

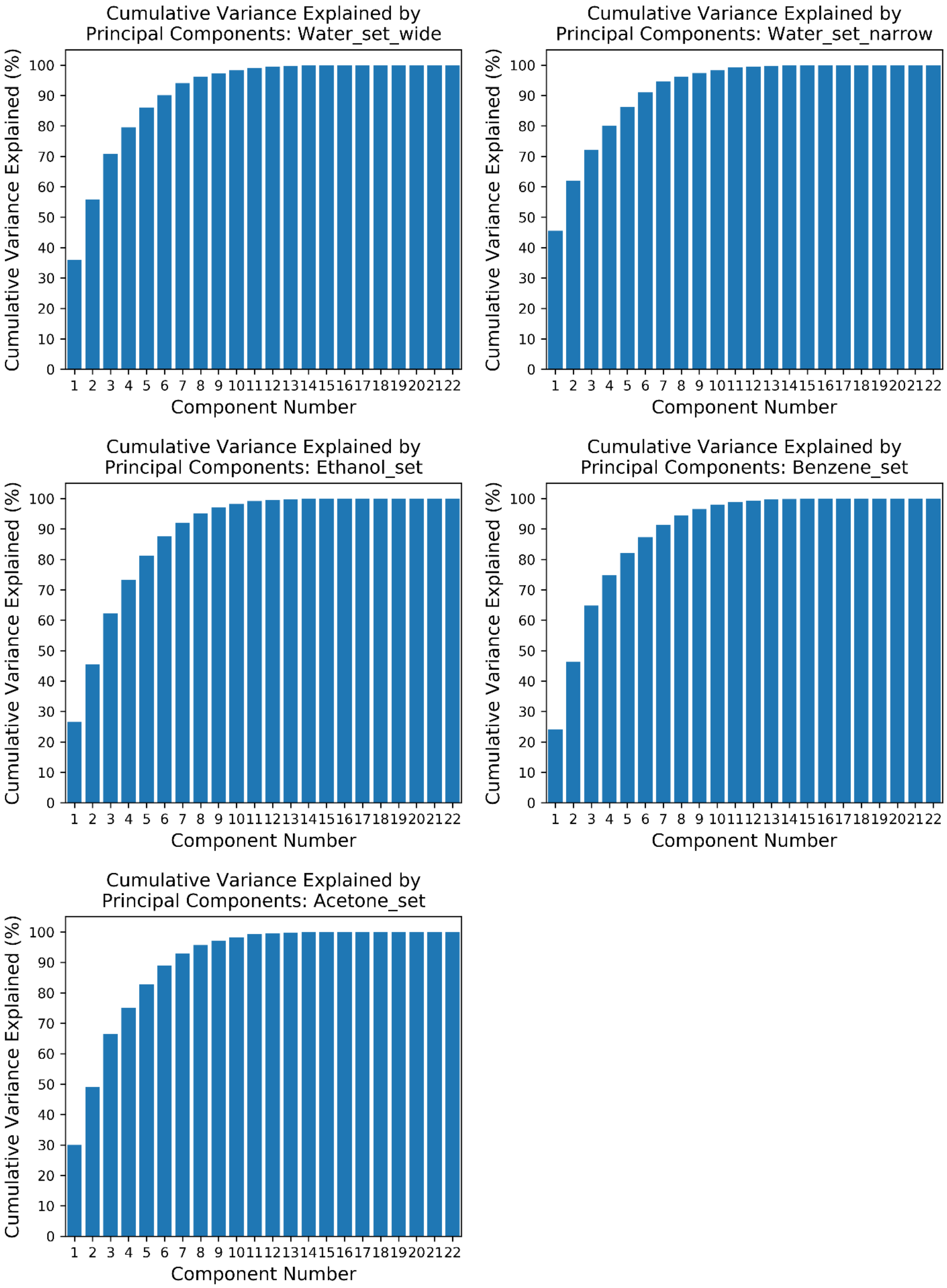

Figure 10. Scree plots of Principal Component Analysis for the datasets

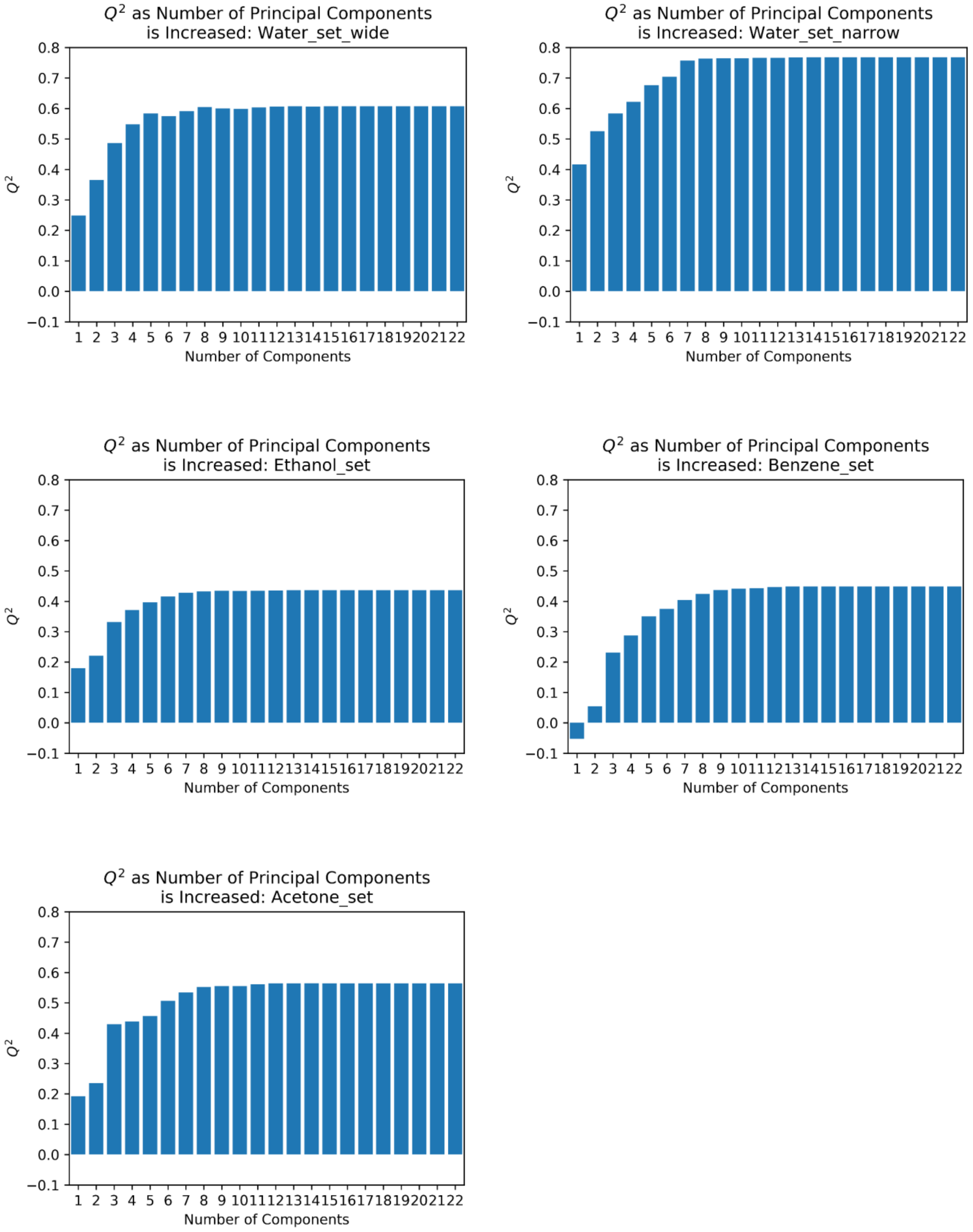

Figure 11. Q^2 plots of Principal Component Analysis for the datasets

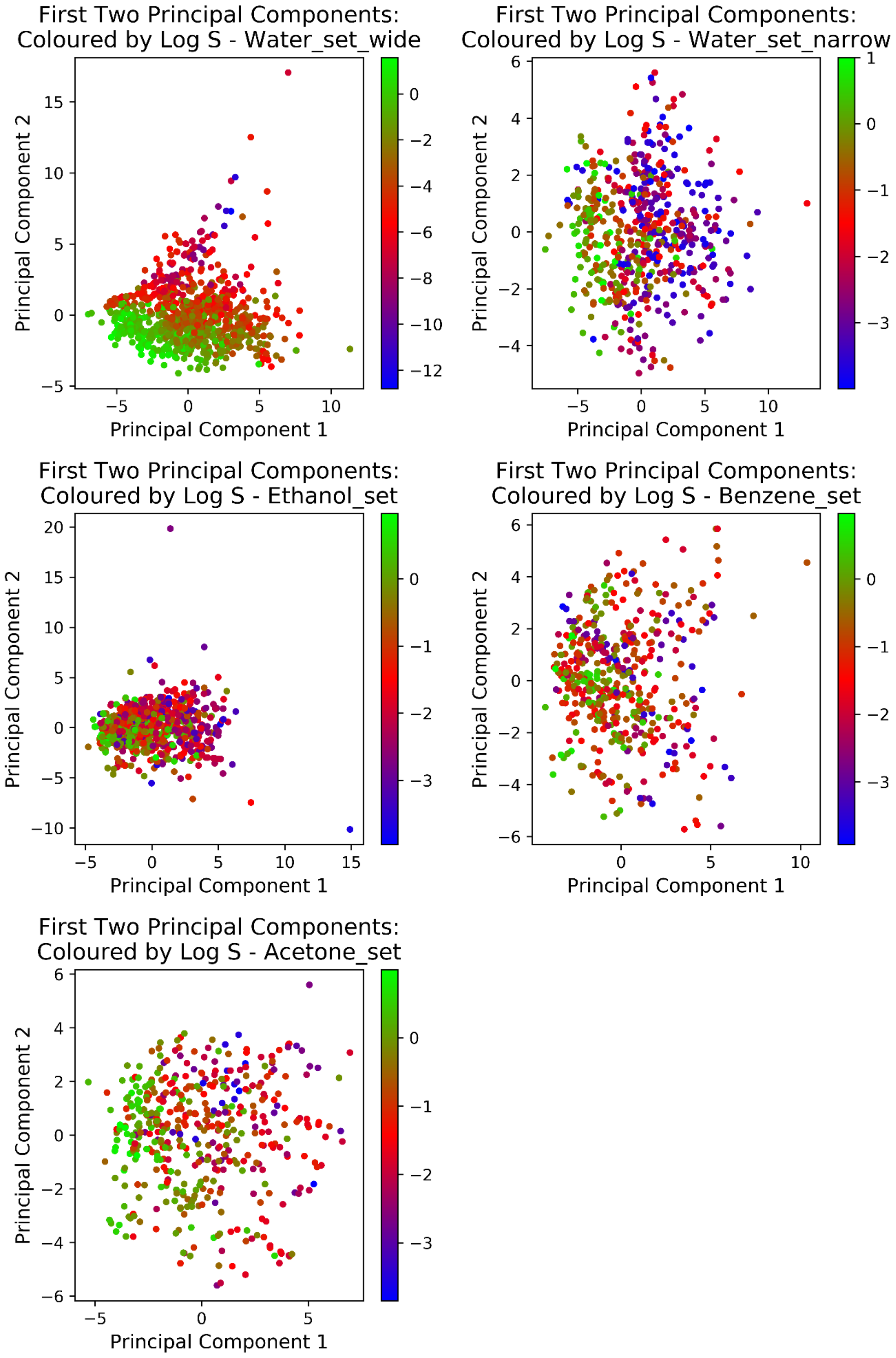

Figure 12. Principal Component Analysis for the datasets; describing 50-60 % of the variance in the descriptor space

3.2 Descriptors correlation analysis

Descriptors were also analysed for correlation using numpy correlation functions (details in section 7.9). In order to prevent the models from being biased by a number of descriptors which are highly correlated, these descriptors could be removed to form a smaller subset. The descriptor correlation is shown in **Figure 13**. The same trend was across all datasets. A threshold of $R^2 = 0.9$ was used to remove descriptors. Number of atoms (N_atoms) was also discarded due to its similarity to Molecular Weight (MW) and Solvent Accessible Surface Area (SASA). The new set of 14 descriptors is shown in **Table 4**.

(a)

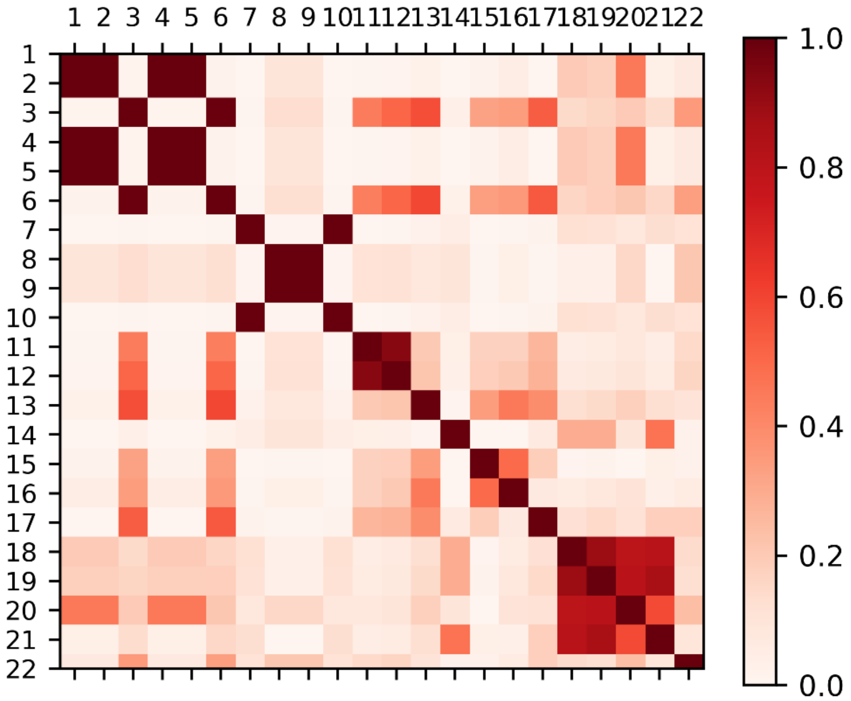

Descriptor 1	Descriptor 2	R^2
E0_gas	E0_solv	1.00
E0_gas	G_gas	1.00
E0_gas	G_solv	1.00
E0_solv	G_gas	1.00
E0_solv	G_solv	1.00
DeltaE0_sol	DeltaG_sol	0.99

G_gas	G_solv	1.00
gas_dip	solv_dip	0.93
HOMO	LsolvHsolu	1.00
LUMO	LsoluHsolv	1.00

(b)

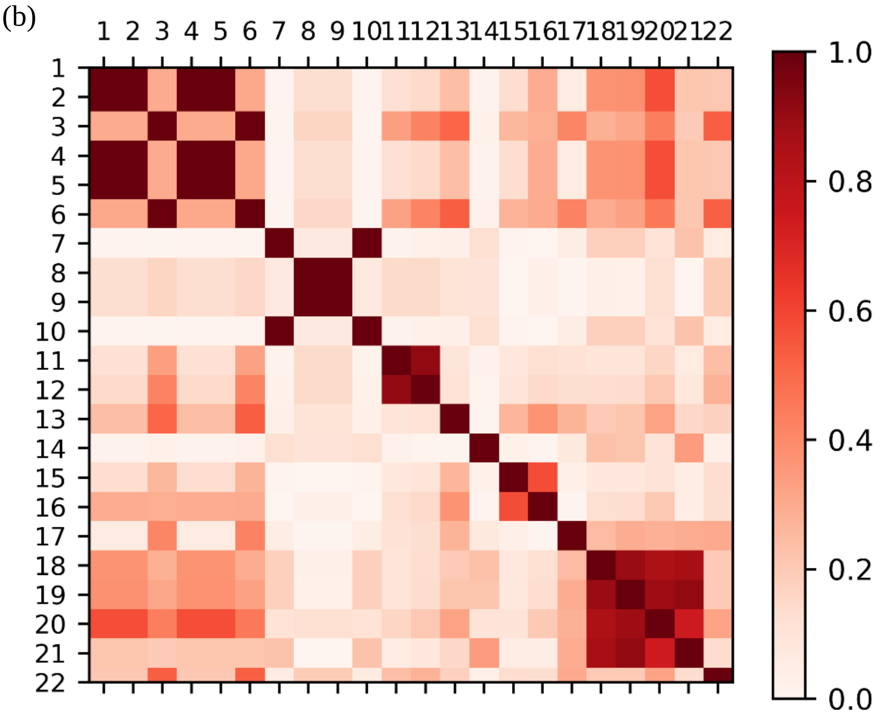

(c)

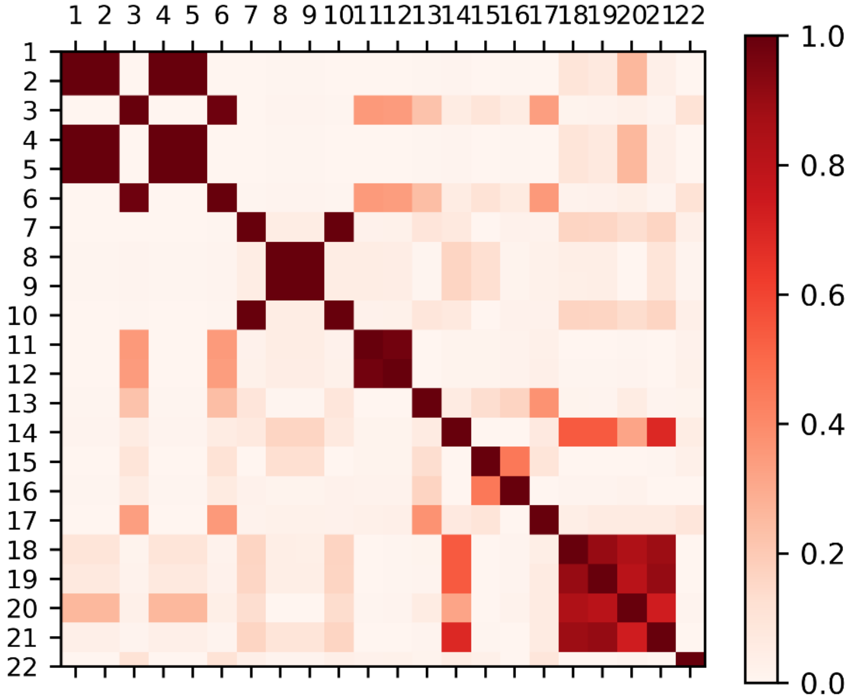
1 2 3 4 5 6 7 8 9 10 11 12 13 14 15 16 17 18 19 20 21 22
1.0
0.8
0.6
0.4
0.2
0.0

(d)

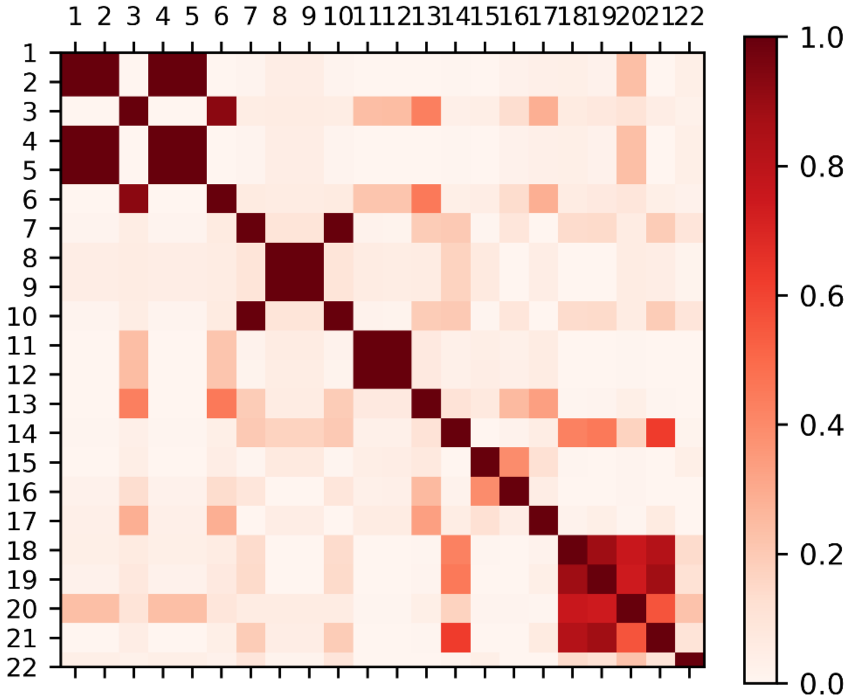
1 2 3 4 5 6 7 8 9 10 11 12 13 14 15 16 17 18 19 20 21 22
1.0
0.8
0.6
0.4
0.2
0.0

(e)

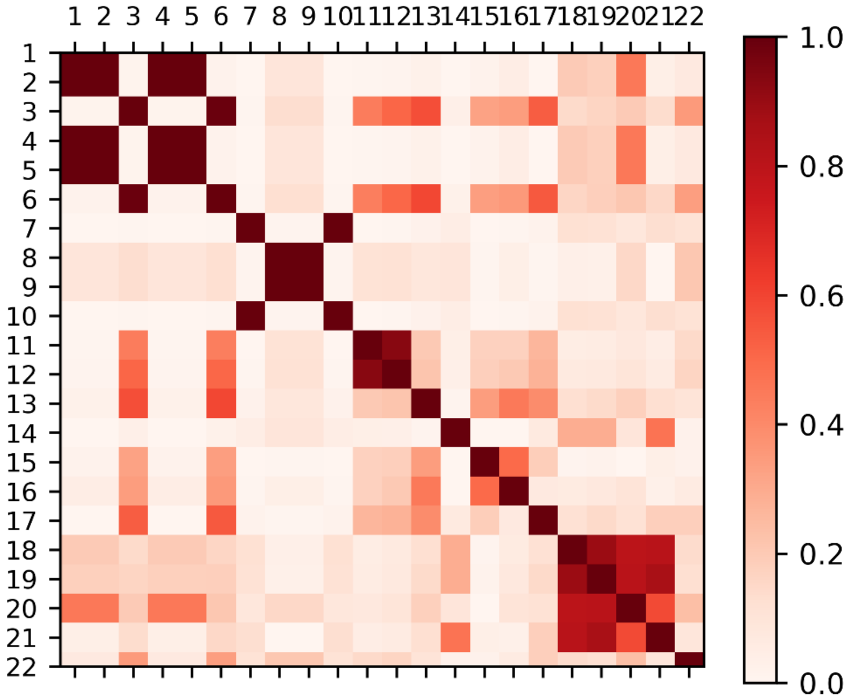

Figure 13. Descriptors correlation (see numbering in Table 1, manuscript) for all dataset. (a) ***Water_set_wide***: descriptors with $R^2 > 0.9$ are shown in the table. Highlighted in green are the descriptors retained, shown in orange are the descriptors discarded; (b) ***Water_set_narrow***; (c) ***Ethanol_set***; (d) ***Benzene_set***; and (e) ***Acetone_set***.

Table 4. Set of 14 uncorrelated descriptors used to build predictive models in this work

No.	Name	Source	Description
1	G_solv	G09	Gibbs free energy of optimised solution structure (Hartrees)
2	DeltaG_sol	G09	Solvation energy calculated as G_solv - G_gas (Hartrees)
3	$L_{solu}H_{solv}$	G09	The gap in energy between the LUMO of the solute and the HOMO of the solvent (eV)
4	$L_{solv}H_{solu}$	G09	The gap in energy between LUMO of the solvent and HOMO of the solute (eV)
5	Solv_dip	G09	Dipole of solution structure (Debye)
6	O_charges	G09	Sum of charges on solution structure oxygen atoms
7	C_charges	G09	Sum of charges on solution structure carbon atoms
8	Most_neg	G09	Charge on most negative atom of solution structure

9	Most_pos	G09	Charge on most positive atom of solution structure
10	Het_charges	G09	Sum of charges on solution structure non-hydrogen/carbon atoms
11	Volume	G09	Molar volume (cm^{-3}.mol)
12	SASA	G09/Pymol	Solvent Accessible Surface Area ($Å^2$)
13	MW	Python	Molecular weight (Daltons)
14	m.p.	Reaxys/ ChemSpider	Experimental melting point (°C)

Supplementary Note 4: Machine learning methodology

4.1 Creating training and test sets

A train/test split was creating by binning the data in intervals of 1 LogS. A proportional amount of data was taken to form a training set (~ 80 % data) and a test set (~ 20 % data). The models trained on the training set and tested on the unseen data of the test set.

K-fold cross-validation splits the data into k groups. Each of these groups are test set in turn, utilising the remaining data as training. Average metrics can then be calculated. K-fold cross validation is good for small datasets as the data is used efficiently (every data point is part of test set at some point) and avoids chance biases in a static train/test split. 10-fold cross validation and leave one out cross validation (a special case where k = number of instances in the dataset) were used in this work. However, it is important to test models on external datasets as the model could be reproducing consistencies within its own data rather than the predicted property. External validation shows how a model might perform in the field. The full dataset is used as the training set and data from an independent source is used as the test set.

4.2 Machine learning protocols

Machine learning was performed in Python 3 with the scikit-learn package[14], with the except of Gaussian Process Regression, which was run using GPy (Gaussian process toolkit https://github.com/SheffieldML/GPy). Prior to building models, descriptors were scaled to be between 0 and 1, to allow equal weighting. Models were built with 8 machine learning methods (Table 5). Default parameters were used in most cases, and then subsequently optimised. ANN architectures (n_estimators in scikit-learn) were analysed for the average of 100 runs with a single hidden layer of between 2 and 5000 nodes. SVM parameters were determined *via* a grid search using 10-fold cross validation of the training set. The number of trees in ExtraTrees (ET) was optimised for the test sets between 1 and 5000. Details of how parameters were optimised are explained in section 4.4. The radial basis function kernel was used for GP and error bars were obtained to 1 standard deviation by obtaining the upper and lower limit which encompassed 68 % of the prediction distribution. **Table 5** shows the machine learning methods and parameters used in this work.

Table 5. Machine learning methods

Abbreviation	Scikit-learn method	Parameters
MLR	linear_model.LinearRegression	Default
PLS	cross_decomposition.PLSRegressor	n_components=9
ANN	neural_network.MLPRegressor	n_estimators = 300

SVM	svm.SVR	Default; C, epsilon and gamma optimised per solvent
RF	ensemble.RandomForestRegressor	n_trees = 500
ET	ensemble.ExtraTreesRegressor	n_trees = 500
Bag	ensemble.BaggingRegressor	n_trees = 500
**Abbreviation**	**GPy method**	**Parameters**
GP	GPy.kern.RBF	variance=1, lengthscale=1

4.3 Model evaluation

The following metrics are commonly used to evaluate how well predictions compare to real (in our case experimental) values. Pearson's R^2, a common measure of correlation, and root mean square error (RMSE), a measure of the typical error associated with each observation, are defined in equations (1) and (2), where $\bar{x}$ and $\bar{y}$ are the mean values of x and y. x_{pred} and y_{pred} are the predicted values of x and y. n is the number of instances and $\hat{y}_i$ is the predicted value of y_i, which is the correct value.

$$R^2 = \left(\frac{\sum(x_{pred} - \bar{x})(y_{pred} - \bar{y})}{\sqrt{\sum(x_{pred} - \bar{x})^2 \sum(y_{pred} - \bar{y})^2}} \right)^2 \quad (1)$$

$$RMSE = \sqrt{\frac{1}{n}\sum_{i=1}^{n}(\hat{y}_i - y_i)^2} \quad (2)$$

In addition, % ± a certain interval can be defined. In the case of solubility prediction, as experimental error is around LogS±0.5-0.7, % LogS within ± 0.7 (%LogS±0.7) with experimental value is a useful measure of molecules within the experimental error. The % LogS within ± 1.0 (%LogS±1.0) of experimental is a more arbitrary decision but does allow easy comprehension of molecules within an order of magnitude of the experimental value. Metrics were calculated in Python, as shown in 7.10.

4.4 Optimisation of machine learning methods

4.4.1 SVM

The **GridsearchCV** from **sklearn** was used to optimise the parameters with default settings. The parameters in SVM are: “kernel” (the function used to transform non-linearity to linearity); “epsilon” (specifies the limits wherein no penalty is associated in the training loss function); “gamma” (kernel coefficient); and “C” (penalty parameter on the error term). Each parameter was optimised in turn with a 10-fold cross validation protocol to maximise the Coefficient of Determination, ***r2_score*** in ***sklearn*** (herein called “COD” to avoid confusion to Pearson’s R^2, which is referred to simply as “R^2” in this

paper), as the scoring function. Default parameters were compared to models with these optimised parameters. In each case the COD decreased from default to optimised parameters. This is shown in **Table 6** to **Table 10** and **Figure 14** to **Figure 18**.

Table 6. Performance metrics of default and optimised SVM parameters in ***Water_set_wide*** dataset

Parameter	Optimised Value	Metric	Default	Optimised
kernel	rbf	R^2	0.90	0.89
epsilon	0.1	RMSE	0.83	0.85
gamma	0.03	%LogS±0.7	66.3	71.6
C	13	%LogS±1.0	81.1	78.9
		COD	0.86	0.88

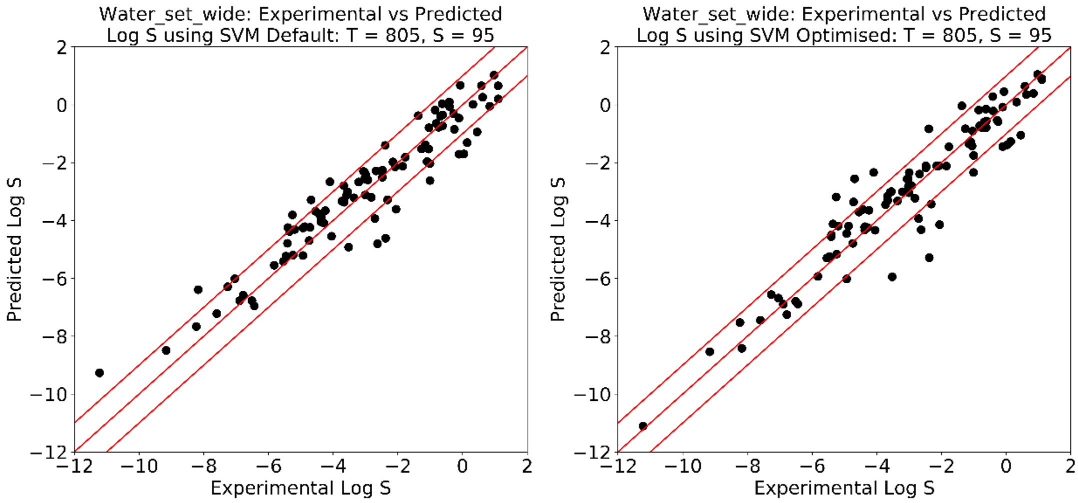

Figure 14. Performance graphs of default and optimised SVM parameters in ***Water_set_wide*** dataset

Table 7. Performance metrics of default and optimised SVM parameters in ***Water_set_narrow*** dataset

Parameter	Optimised Value	Metric	Default	Optimised
kernel	rbf	R^2	0.76	0.76
epsilon	0.1	RMSE	0.72	0.71
gamma	0.07	%LogS±0.7	68.7	65.2

C	3	%LogS±1.0	83.5	81.7
		COD	0.58	0.63

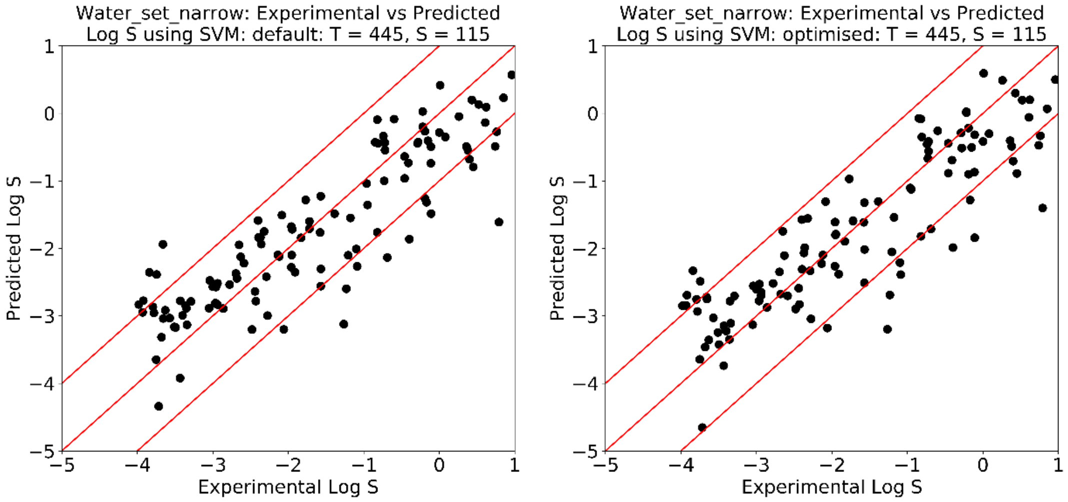

Figure 15. Performance graphs of default and optimised SVM parameters in ***Water_set_narrow*** dataset

Table 8. Performance metrics of default and optimised SVM parameters in ***Ethanol_set*** dataset

Parameter	Optimised Value	Metric	Default	Optimised
kernel	rbf	R^2	0.51	0.51
epsilon	0.1	RMSE	0.80	0.81
gamma	0.03	%LogS±0.7	65.5	64.1
C	6	%LogS±1.0	80.3	78.9
		COD	0.12	0.25

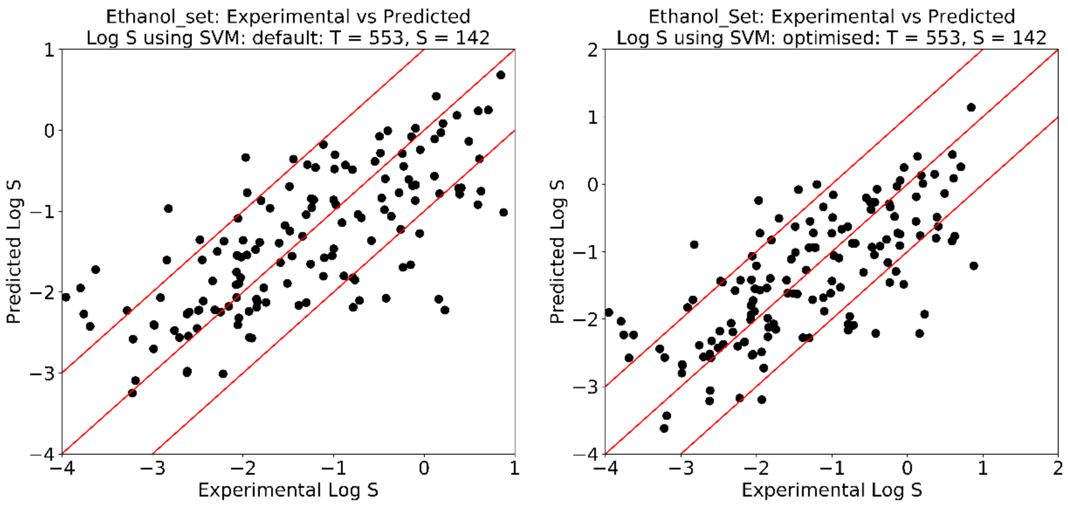

Figure 16. Performance graphs of default and optimised SVM parameters in ***Ethanol_set*** dataset

Table 9. Performance metrics of default and optimised SVM parameters in ***Benzene_set*** dataset

Parameter	Optimised Value	Metric	Default	Optimised
kernel	rbf	R^2	0.68	0.71
epsilon	0.1	RMSE	0.60	0.58
gamma	0.03	%LogS±0.7	77.7	76.6
C	4	%LogS±1.0	88.3	89.4
		COD	0.51	0.62

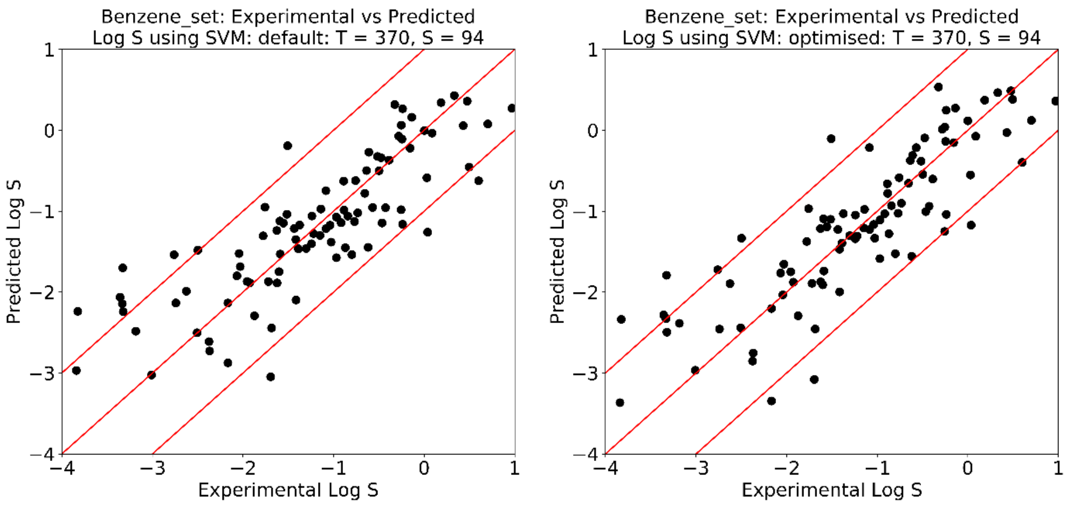

Figure 17. Performance graphs of default and optimised SVM parameters in ***Benzene_set*** dataset

Table 10. Performance metrics of default and optimised SVM parameters in ***Acetone_set*** dataset

Parameter	Optimised Value	Metric	Default	Optimised
kernel	rbf	R^2	0.39	0.42
epsilon	0.0002	RMSE	0.85	0.83
gamma	0.08	%LogS±0.7	69.6	72.8
C	1.2	%LogS±1.0	83.7	81.5
		COD	-0.07	-0.01

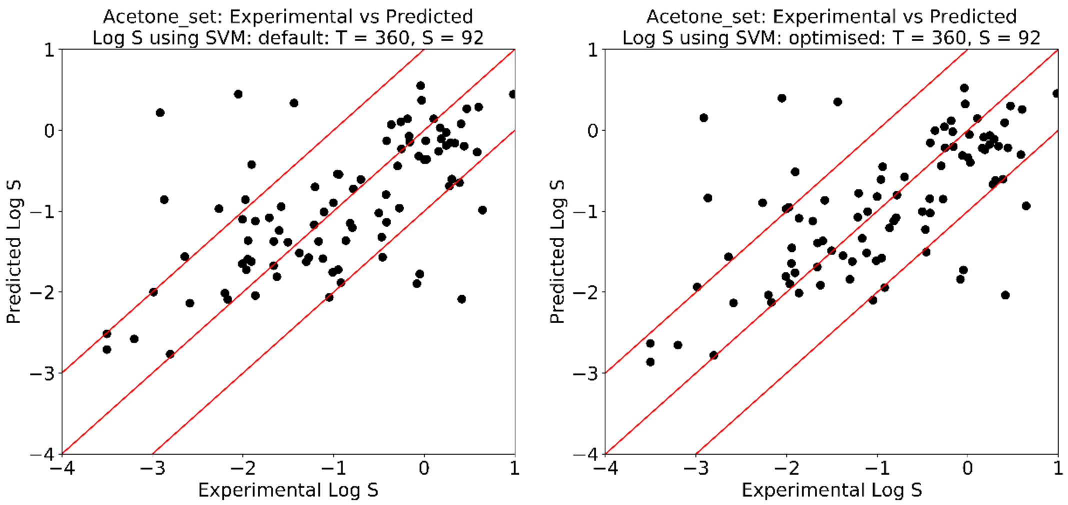

Figure 18. Performance graphs of default and optimised SVM parameters in ***Acetone_set*** dataset

4.4.2 ExtraTrees, Random Forest & Bag

To optimise the computational cost and avoid overfitting, number of trees was varied to see how few trees were required to get good models, as shown in **Figure 19**.

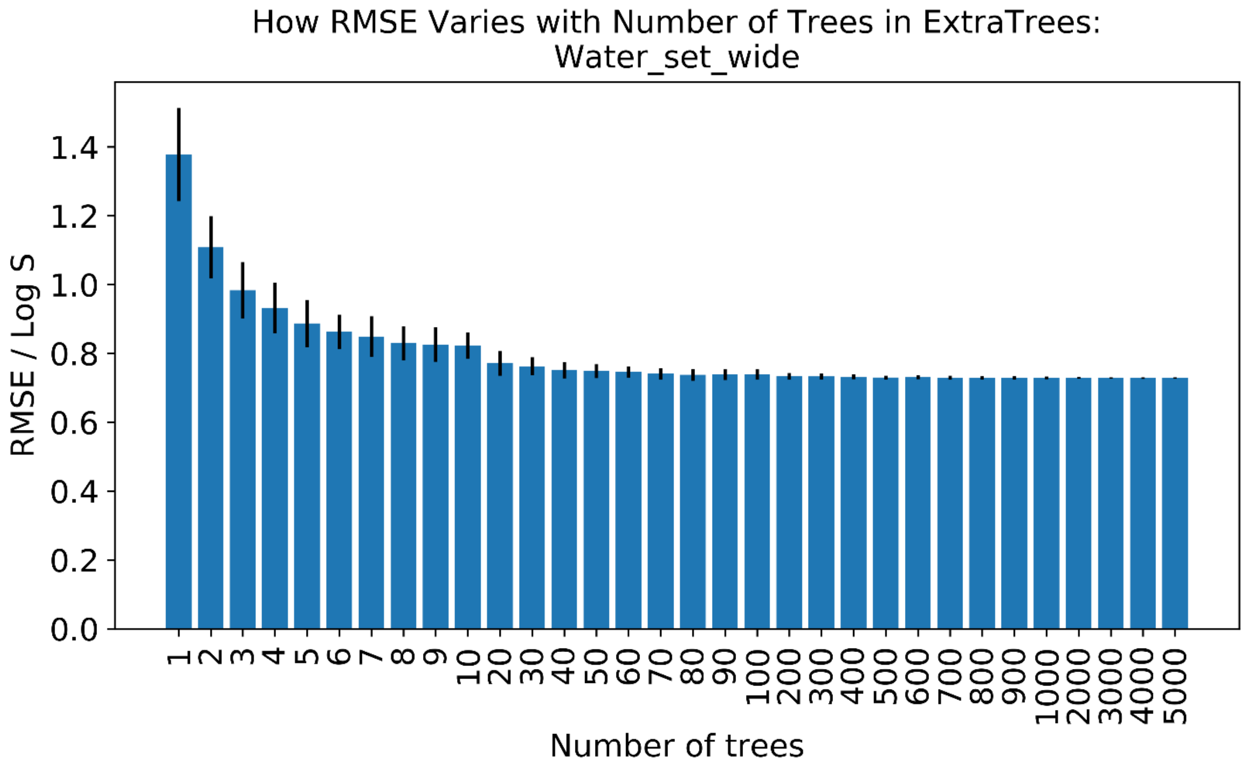

Figure 19. Dependence of RMSE of LogS on the number of trees in ET models

The same pattern was seen across all datasets. Random Forest and Bag are very similar to ET and produced almost identical plots. As the number of trees increases the RMSE decreases as well as the

variance (standard deviation over 100 runs). 500 trees were accepted as the standard parameter for this method.

4.4.3 Neural Networks

The number of nodes in a single layer was varied from 1 to 5000. In each case the average metrics of 100 runs was calculated. Convergence problems, where the neural network training terminated before consistency is achieved, meant that some architectures with a very small number of nodes in the hidden layer were skipped. The maximum number of cycles allowed, before skipping, was 800. If < 3 successful neural networks were built, this architecture was discarded entirely. This is shown in **Figure 20** to **Figure 34**.

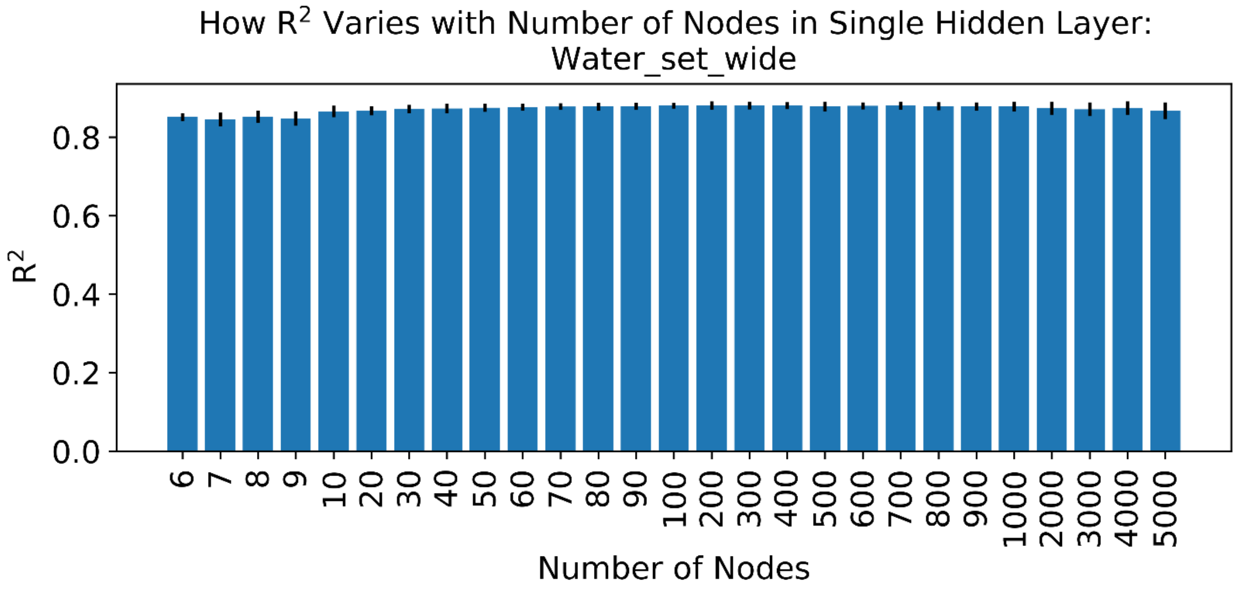

Figure 20. How R^2 varies as the number of nodes in a single hidden layer for ***Water_set_wide*** is increased. Artificial Neural Network Multi-Layer Perceptron set up

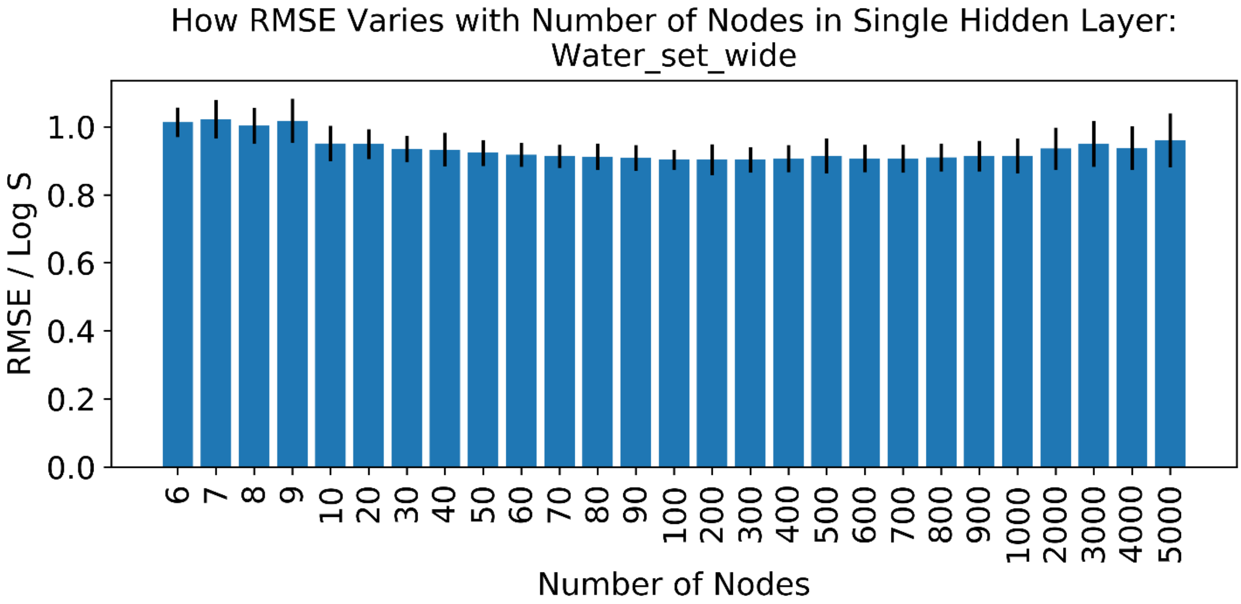

Figure 21. How RMSE varies as the number of nodes in a single hidden layer for ***Water_set_wide*** is increased. Artificial Neural Network Multi-Layer Perceptron set up

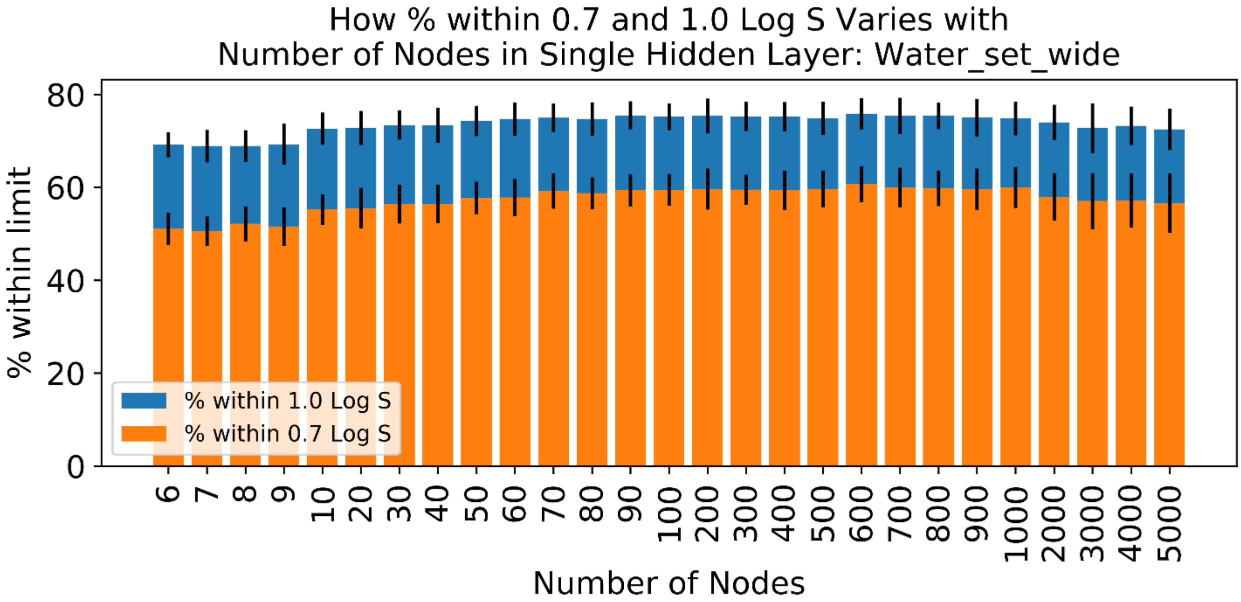

Figure 22. How %LogS±0.7 and %LogS±1.0 varies as the number of nodes in a single hidden layer for ***Water_set_wide*** is increased. Artificial Neural Network Multi-Layer Perceptron set up

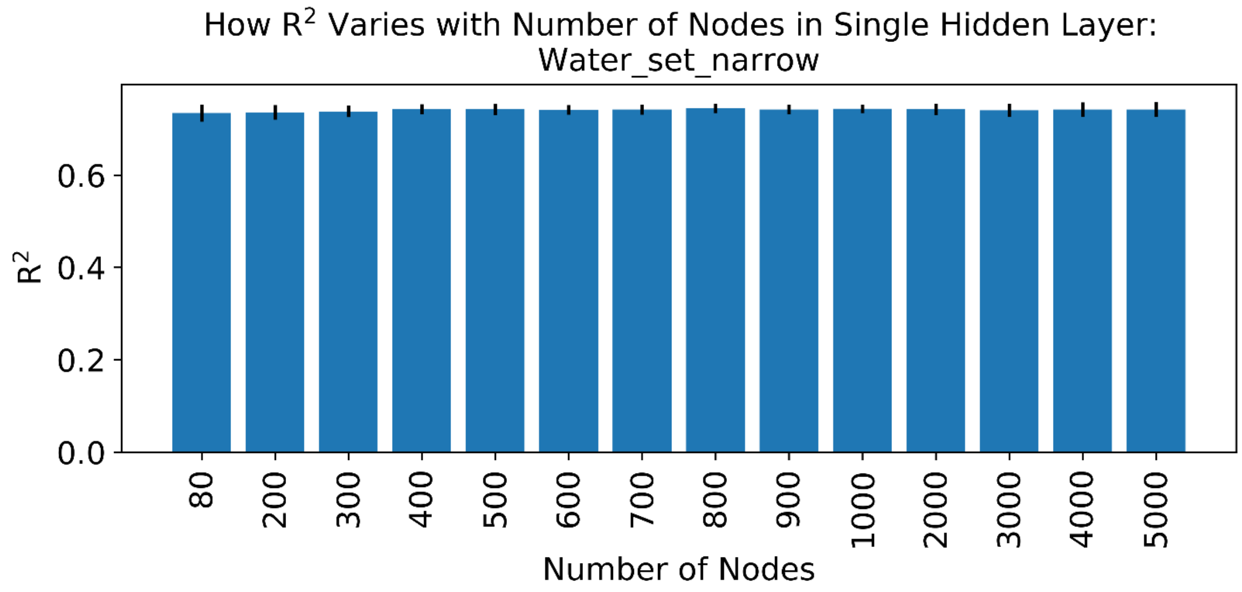

Figure 23. How R^2 varies as the number of nodes in a single hidden layer for ***Water_set_narrow*** is increased. Artificial Neural Network Multi-Layer Perceptron set up

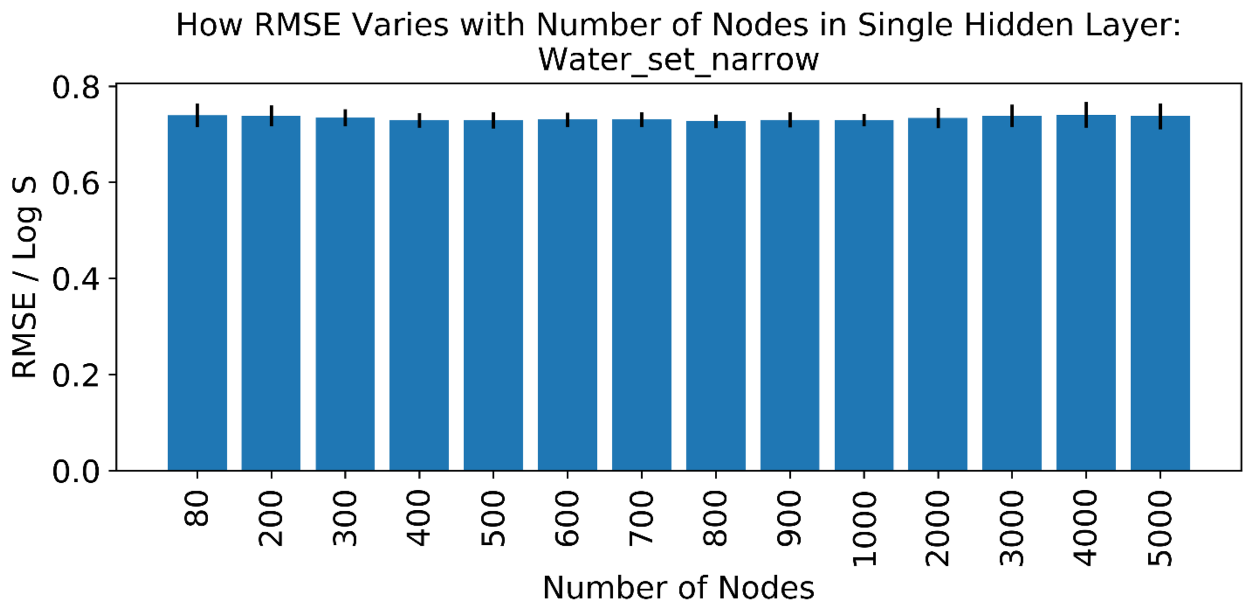

Figure 24. How RMSE varies as the number of nodes in a single hidden layer for ***Water_set_narrow*** is increased. Artificial Neural Network Multi-Layer Perceptron set up

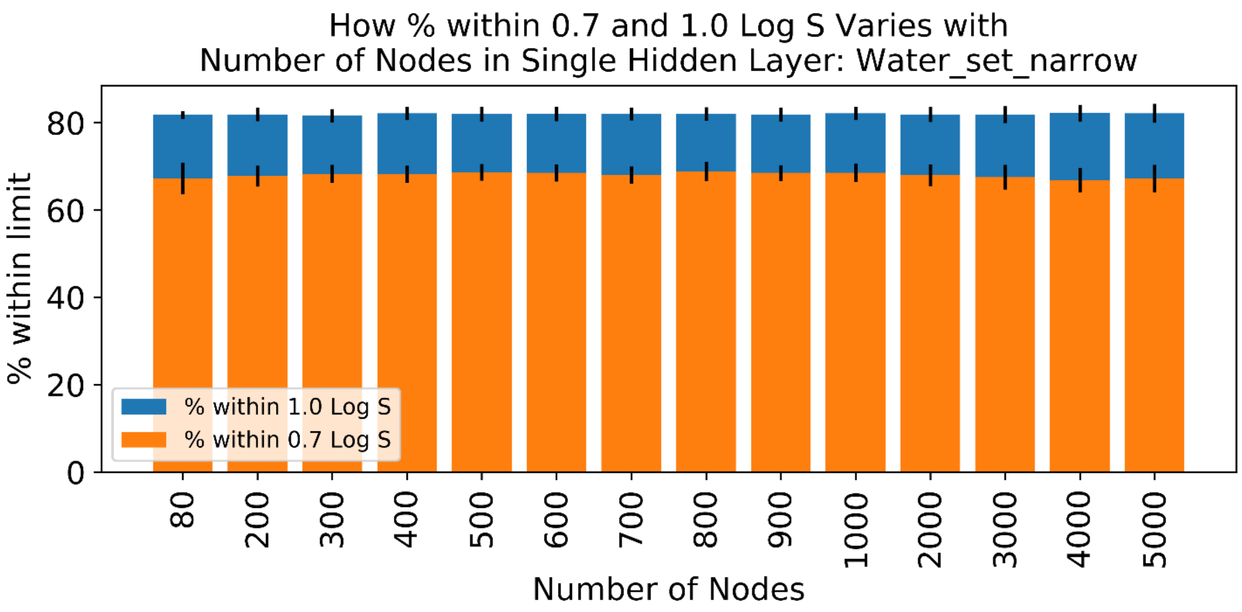

Figure 25. How %LogS±0.7 and %LogS±1.0 varies as the number of nodes in a single hidden layer for ***Water_set_narrow*** is increased. Artificial Neural Network Multi-Layer Perceptron set up

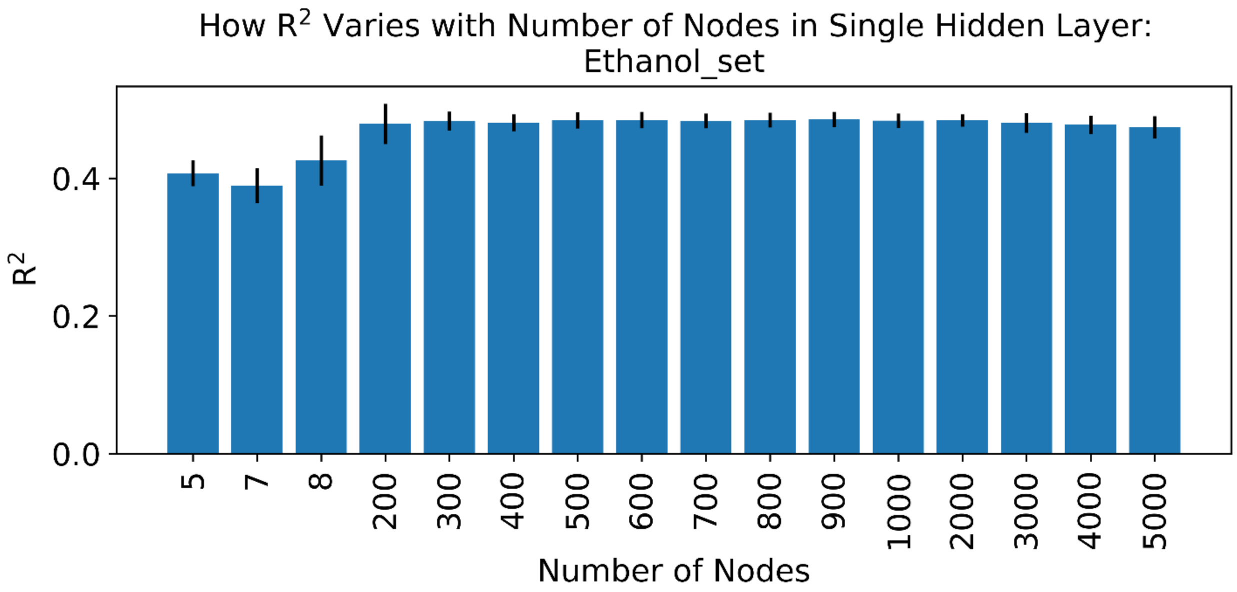

Figure 26. How R^2 varies as the number of nodes in a single hidden layer for ***Ethanol_set*** is increased. Artificial Neural Network Multi-Layer Perceptron set up

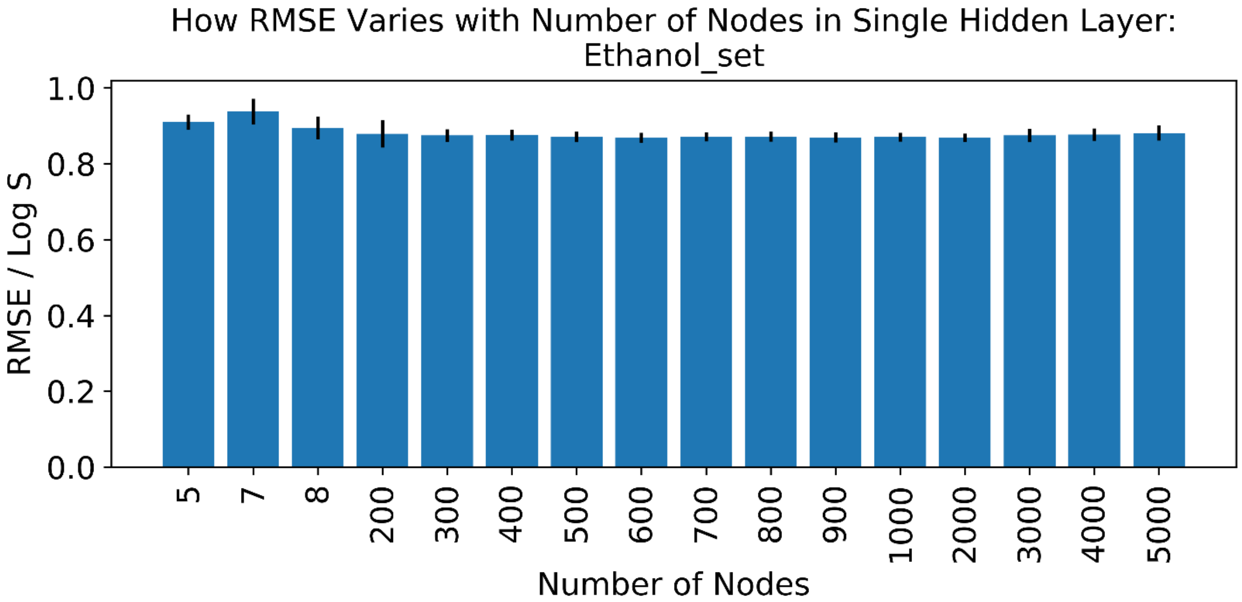

Figure 27. How RMSE varies as the number of nodes in a single hidden layer for ***Ethanol_set*** is increased. Artificial Neural Network Multi-Layer Perceptron set up

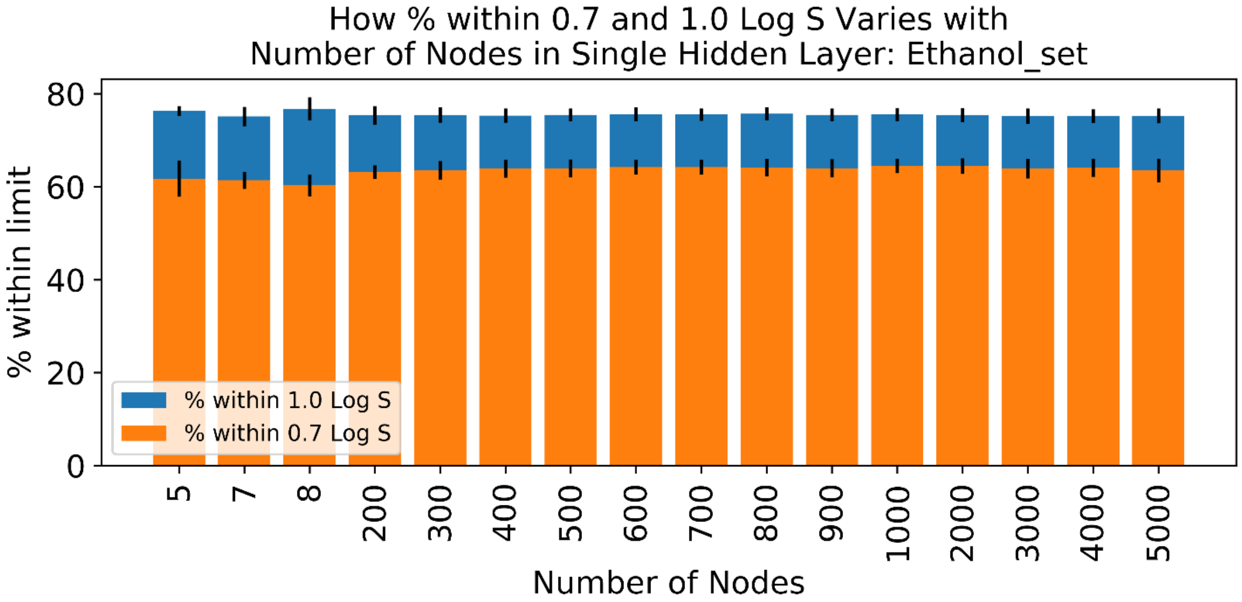

Figure 28. How %LogS±0.7 and %LogS±1.0 varies as the number of nodes in a single hidden layer for ***Ethanol_set*** is increased. Artificial Neural Network Multi-Layer Perceptron set up

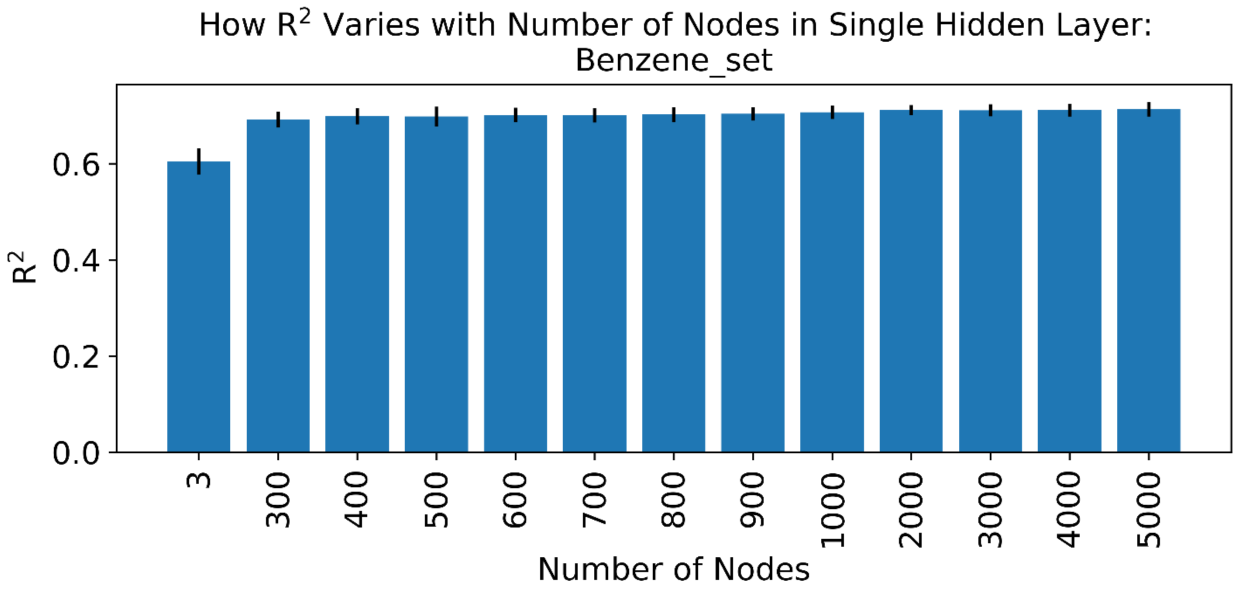

Figure 29. How R^2 varies as the number of nodes in a single hidden layer for ***Benzene_set*** is increased. Artificial Neural Network Multi-Layer Perceptron set up

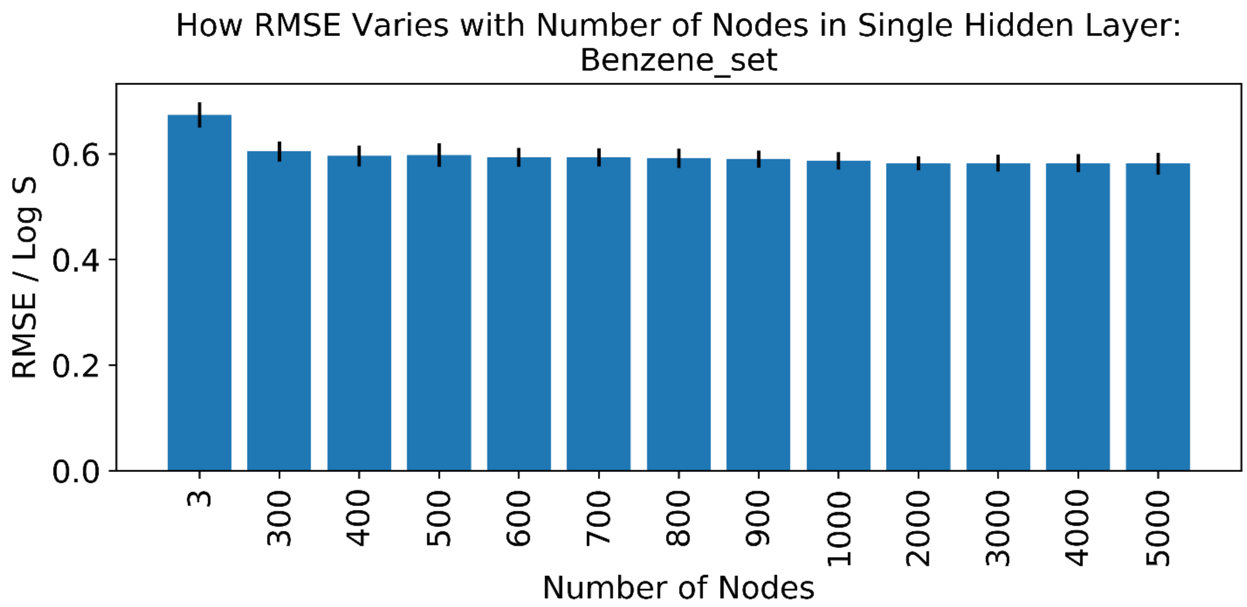

Figure 30. How RMSE varies as the number of nodes in a single hidden layer for ***Benzene_set*** is increased. Artificial Neural Network Multi-Layer Perceptron set up

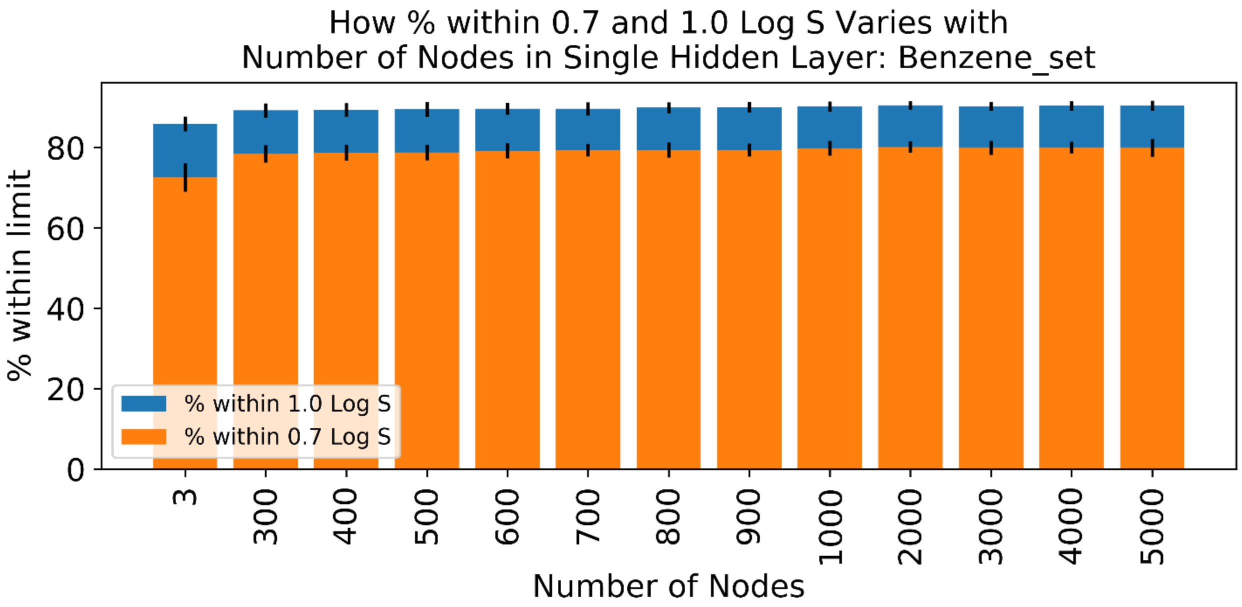

Figure 31. How %LogS±0.7 and %LogS±1.0 varies as the number of nodes in a single hidden layer for ***Benzene_set*** is increased. Artificial Neural Network Multi-Layer Perceptron set up

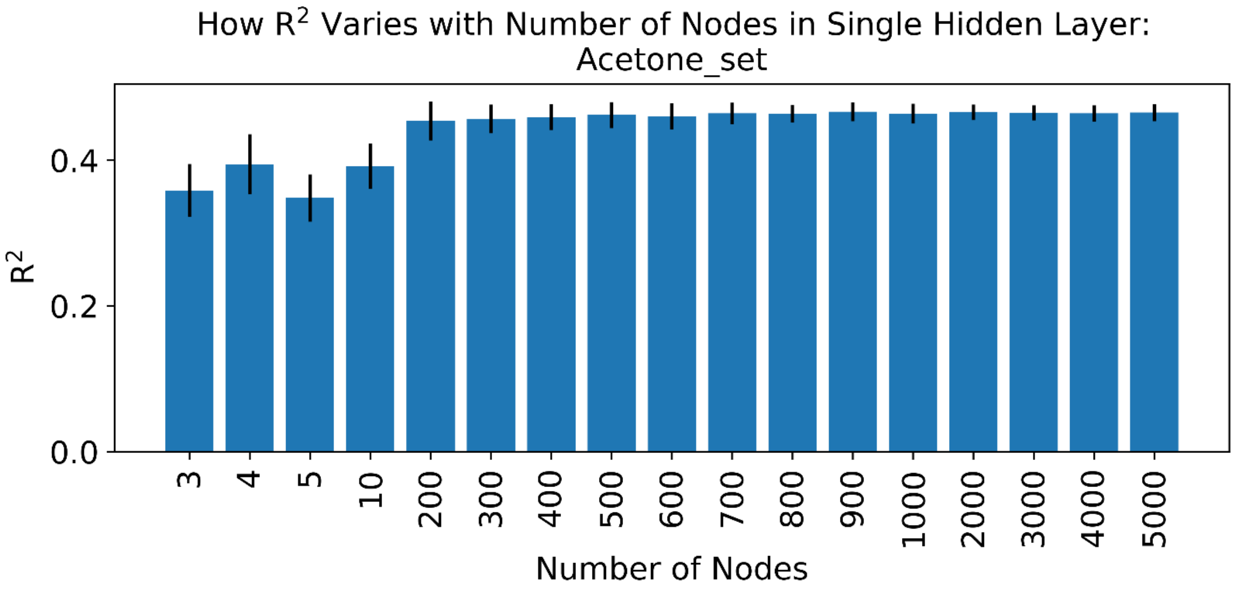

Figure 32. How R^2 varies as the number of nodes in a single hidden layer for ***Acetone_set*** is increased. Artificial Neural Network Multi-Layer Perceptron set up

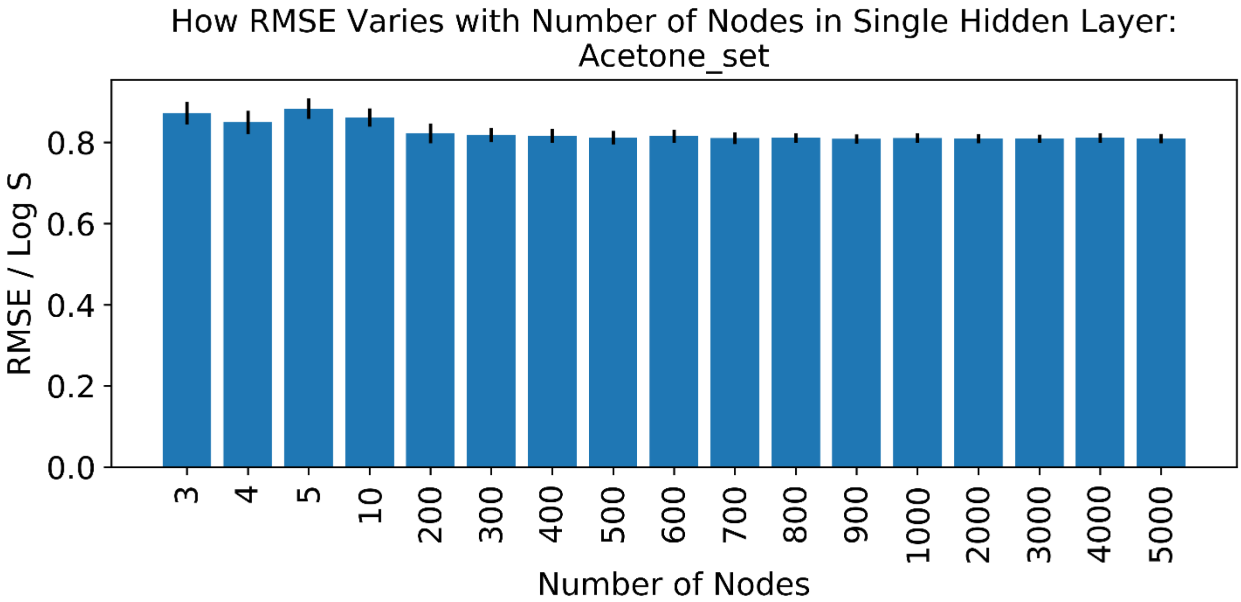

Figure 33. How RMSE varies as the number of nodes in a single hidden layer for ***Acetone_set*** is increased. Artificial Neural Network Multi-Layer Perceptron set up

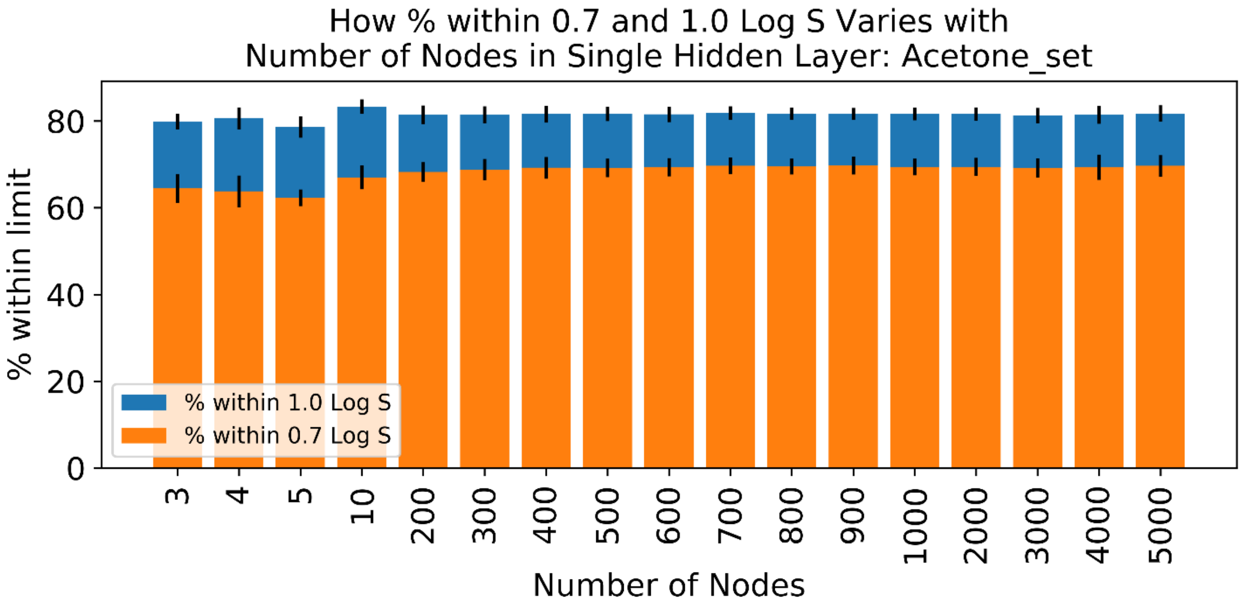

Figure 34. How %LogS±0.7 and %LogS±1.0 varies as the number of nodes in a single hidden layer for ***Acetone_set*** is increased. Artificial Neural Network Multi-Layer Perceptron set up

As the number of nodes increases, the R^2 increases, RMSE decreases and %LogS±0.7 and %LogS±1.0 increases with the error (standard deviation of 100 runs) until 300 nodes. After that the metrics become

worse (or remain constant), due to overfitting. Thus, 300 nodes were set as the standard parameters for all neural network models. Multi-layer ANN models were not evaluated due to the limited size of datasets in this study.

4.5 Impact of skipping more than 1 descriptor

This analysis was performed with the ExtraTrees model for ***Water_set_wide***. Combinations of 1, 2 (14x13/2 = 91 combinations) and 3 (14x13x12/6=364 combinations) descriptors were sequentially excluded, and the resulted models were evaluated their impact on the R^2, RMSE, %LogS±0.7 and %LogS±1.0. ***Water_set_wide*** was selected for this to minimise the interference from the importance of MP so we can evaluate the role of other descriptors.

The impact of the set of missing descriptors is summarised below (red points for full descriptor set). Similar trends were observed with all 4 metrics.

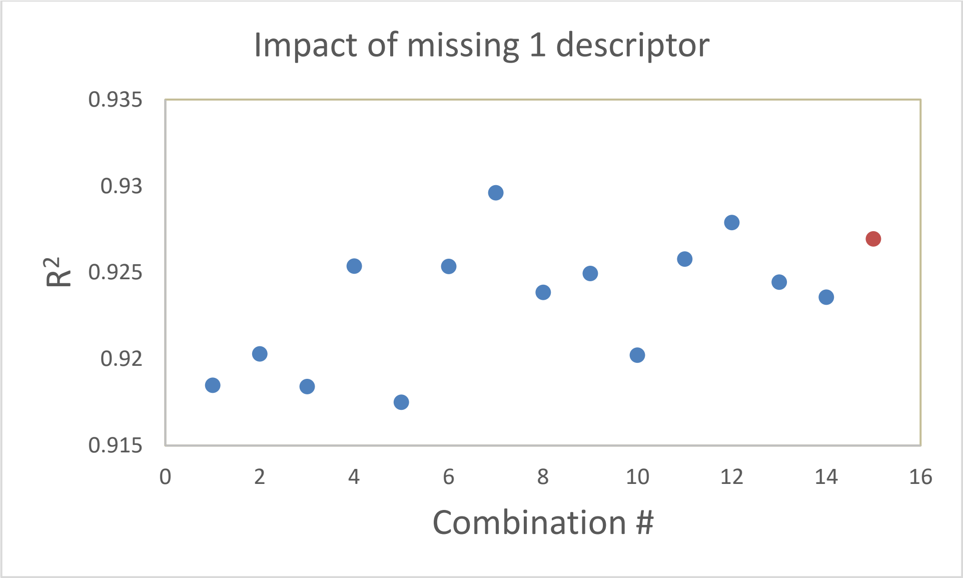

Figure 35. Impact of missing each one descriptor at a time on R^2 of the ET model for ***Water_set_wide***

The descriptors with the highest impact are Het_charges, Most_pos, Most_neg, O_charges and DeltaG_sol.

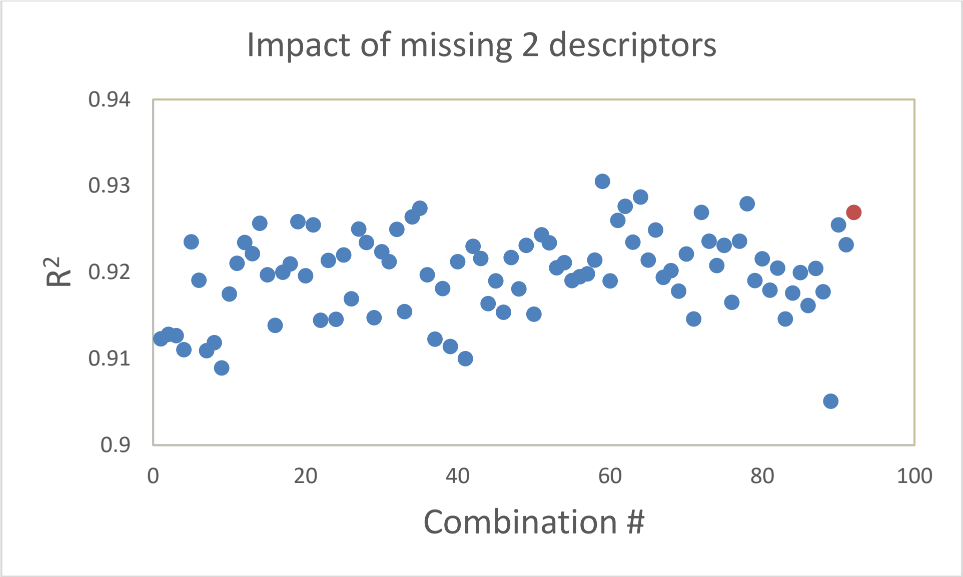

Figure 36. Impact of missing each two descriptors at a time on R^2 of the ET model for ***Water_set_wide***

The three combinations with the highest impact are:

[O_charges, Most_neg]

[DeltaG_sol, O_charges]

[MW, G_solv]

These included the individual descriptors which have high impact in the first plot, but the one with the highest impact, i.e. [MW, G_solv], did not include any of those descriptors.

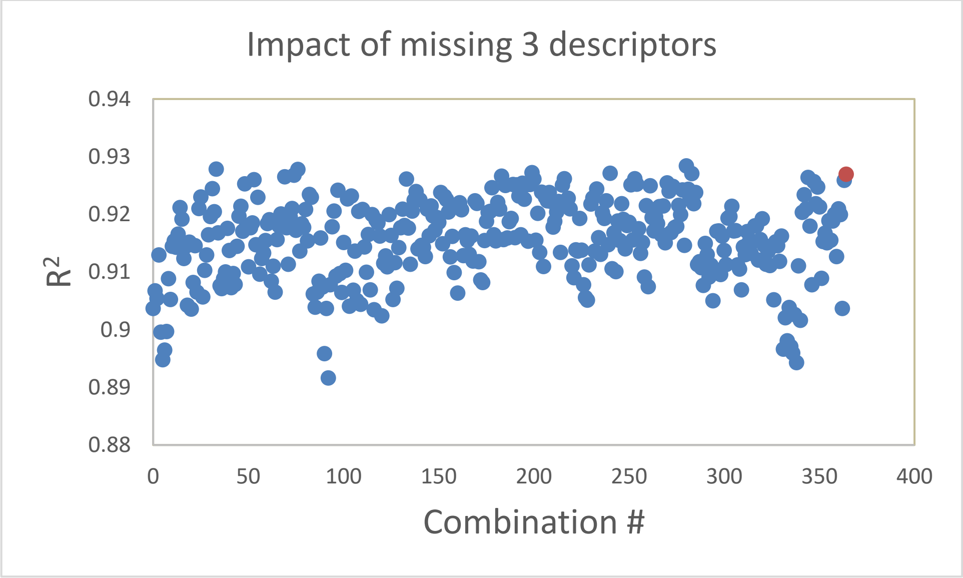

Figure 37. Impact of missing each three descriptors at a time on R^2 of the ET model for ***Water_set_wide***

The three combinations with the highest impact are

[O_charges, Most_neg, Het_charges]

[O_charges, Most_neg, Most_pos]

[DeltaG_sol, O_charges, Het_charges]

[DeltaG_sol, O_charges, Most_neg]

[MW, G_solv, Het_charges]

[MW, G_solv, Most_neg]

[MW, G_solv, O_charges]

[MW, G_solv, SASA]

[MW, G_solv, LsoluHsolv]

4.6 Initial solubility prediction models

The initial predictive models were built using the subset of 14 uncorrelated DFT descriptors. They were tested with an explicit test set and with 10-fold cross validation.

4.6.1 Cross Validation Results

The full datasets were analysed with 10-fold cross validation. The metrics are summarised in **Table 11**.

Table 11. 10-fold Cross Validation Results for 5 datasets and 8 machine learning protocols

Method	R^2	RMSE	%LogS±0.7	%LogS±1.0
Water_set_wide				
MLR	0.77	1.16	52.1	67.0
ANN	0.87	0.89	64.9	79.9
SVM	0.88	0.85	68.3	81.7
PLS	0.77	1.16	52.2	66.9
RF	0.84	0.95	62.3	75.7
ET	0.87	0.88	66.3	79.4
Bag	0.84	0.95	62.1	76.4
GP	0.88	0.86	67.0	79.2
Water_set_narrow				

MLR	0.60	0.88	61.2	76.3
ANN	0.66	0.83	67.3	79.8
SVM	0.70	0.76	68.6	82.9
PLS	0.60	0.88	61.4	76.1
RF	0.67	0.80	64.7	80.5
ET	0.69	0.77	66.4	82.3
Bag	0.67	0.80	64.8	81.1
GP	0.70	0.77	68.0	82.7
Ethanol_set				
MLR	0.39	0.87	59.9	77.1
ANN	0.51	0.80	67.8	81.6
SVM	0.54	0.76	69.8	83.6
PLS	0.39	0.87	59.7	76.7
RF	0.54	0.76	68.5	82.2
ET	0.54	0.76	69.1	81.7
Bag	0.55	0.76	69.1	82.7
GP	0.53	0.77	68.8	82.0
Benzene_set				
MLR	0.47	0.79	68.8	81.3
ANN	0.58	0.72	71.4	84.5
SVM	0.57	0.72	70.7	84.5
PLS	0.47	0.80	67.5	81.3
RF	0.62	0.67	72.2	87.3

ET	0.64	0.66	72.7	88.4
Bag	0.62	0.67	71.4	87.5
GP	0.57	0.72	70.1	83.8
Acetone_set				
MLR	0.47	0.77	69.2	81.4
ANN	0.54	0.73	71.7	83.4
SVM	0.57	0.70	76.3	84.3
PLS	0.47	0.77	68.8	81.6
RF	0.57	0.69	74.8	85.4
ET	0.56	0.70	73.7	84.5
Bag	0.57	0.70	73.7	84.9
GP	0.52	0.74	72.6	83.4

4.6.2 Internal Test Set Results

Internal test sets were formed by explicit removing dataset from the dataset and using it to test the models. The metrics are summarised in **Table 12** and graphs of experimental against predictions for the best single performing machine learning method, ET, is shown in **Figure 38**. **Figure 39** to **Figure 43** show a comparison of the seven machine learning methods for each dataset.

Table 12. Internal test set metrics

Method	R^2	RMSE	%LogS±0.7	%LogS±1.0
Water_set_wide				
MLR	0.80	1.16	50.5	65.3
ANN	0.90	0.84	58.9	78.9
SVM	0.89	0.85	71.6	78.9
PLS	0.80	1.16	51.6	66.3

RF	0.90	0.83	60.0	75.8
ET	0.93	0.71	66.3	84.2
Bag	0.90	0.82	57.9	76.8
GP	0.88	0.89	68.4	73.7
Water_set_narrow				
MLR	0.68	0.82	61.7	80.0
ANN	0.74	0.74	68.7	84.3
SVM	0.76	0.71	65.2	81.7
PLS	0.68	0.83	61.7	80.0
RF	0.72	0.76	60.9	82.6
ET	0.75	0.73	66.1	80.9
Bag	0.72	0.77	60.9	81.7
GP	0.76	0.71	73.0	81.7
Ethanol_set				
MLR	0.29	0.98	50.7	72.5
ANN	0.49	0.88	64.1	76.8
SVM	0.51	0.81	64.1	78.9
PLS	0.29	0.99	51.4	71.8
RF	0.53	0.79	64.8	82.4
ET	0.50	0.81	62.7	78.9
Bag	0.52	0.80	65.5	79.6
GP	0.51	0.80	66.2	77.5
Benzene_set				

MLR	0.64	0.66	75.5	86.2
ANN	0.67	0.63	77.7	88.3
SVM	0.71	0.58	76.6	89.4
PLS	0.64	0.66	74.5	85.1
RF	0.72	0.57	76.6	90.4
ET	0.75	0.54	76.6	90.4
Bag	0.72	0.57	75.5	89.4
GP	0.70	0.58	79.8	90.4
Acetone_set				
MLR	0.36	0.87	60.9	78.3
ANN	0.42	0.87	67.4	79.3
SVM	0.42	0.83	72.8	81.5
PLS	0.35	0.87	62.0	78.3
RF	0.40	0.84	62.0	80.4
ET	0.40	0.84	63.0	78.3
Bag	0.41	0.83	62.0	80.4
GP	0.42	0.83	68.5	84.8

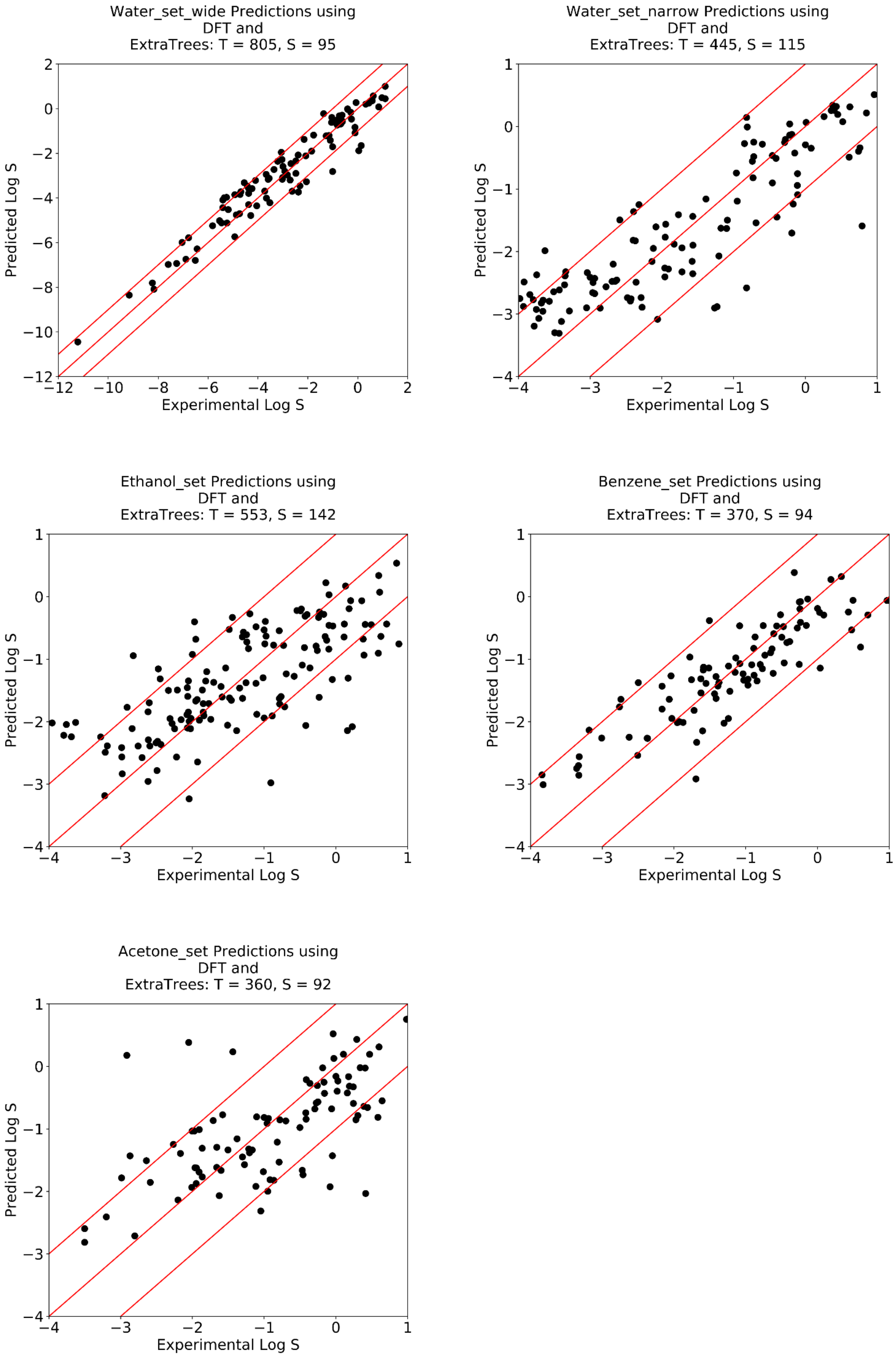

Figure 38. Performance of internal test set predictions compared to experimental for the 5 datasets, for the best overall method, ET

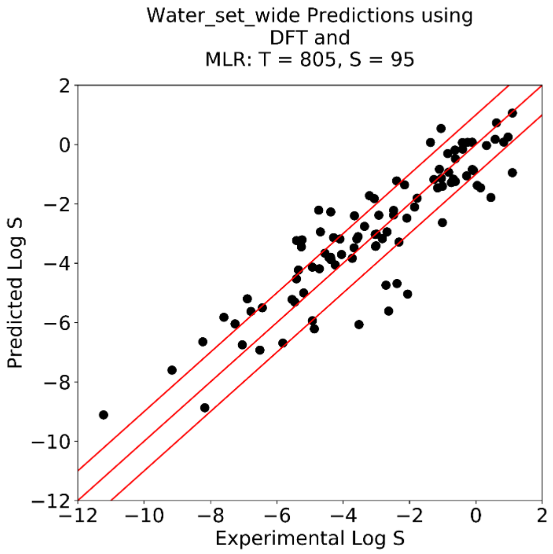
Water_set_wide Predictions using
DFT and
MLR: T = 805, S = 95
Predicted Log S
Experimental Log S

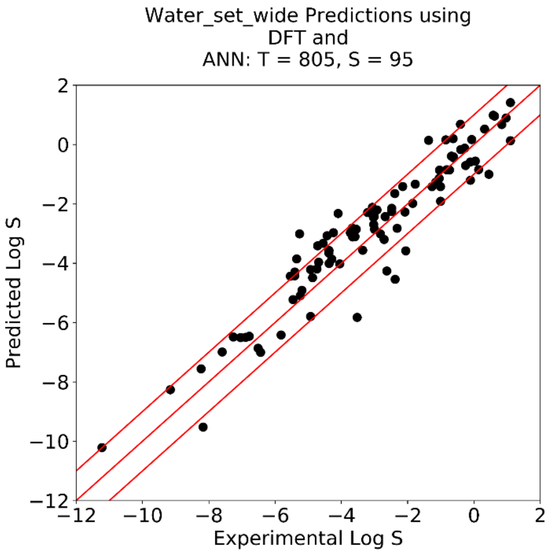
Water_set_wide Predictions using
DFT and
ANN: T = 805, S = 95
Predicted Log S
Experimental Log S

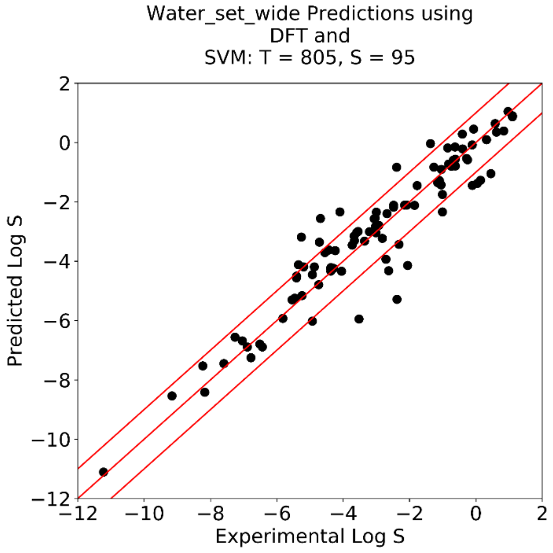
Water_set_wide Predictions using
DFT and
SVM: T = 805, S = 95
Predicted Log S
Experimental Log S

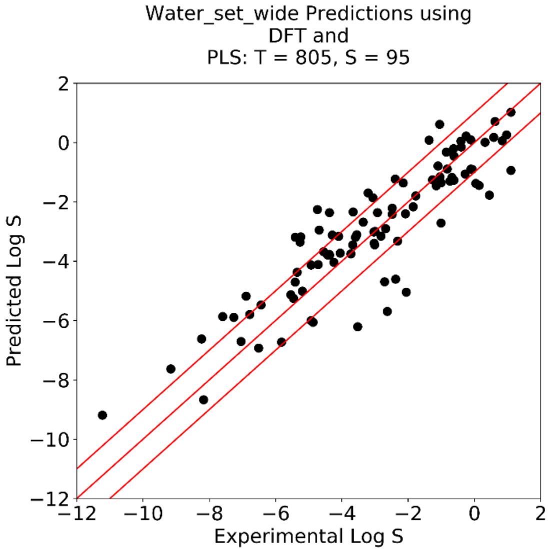
Water_set_wide Predictions using
DFT and
PLS: T = 805, S = 95
Predicted Log S
Experimental Log S

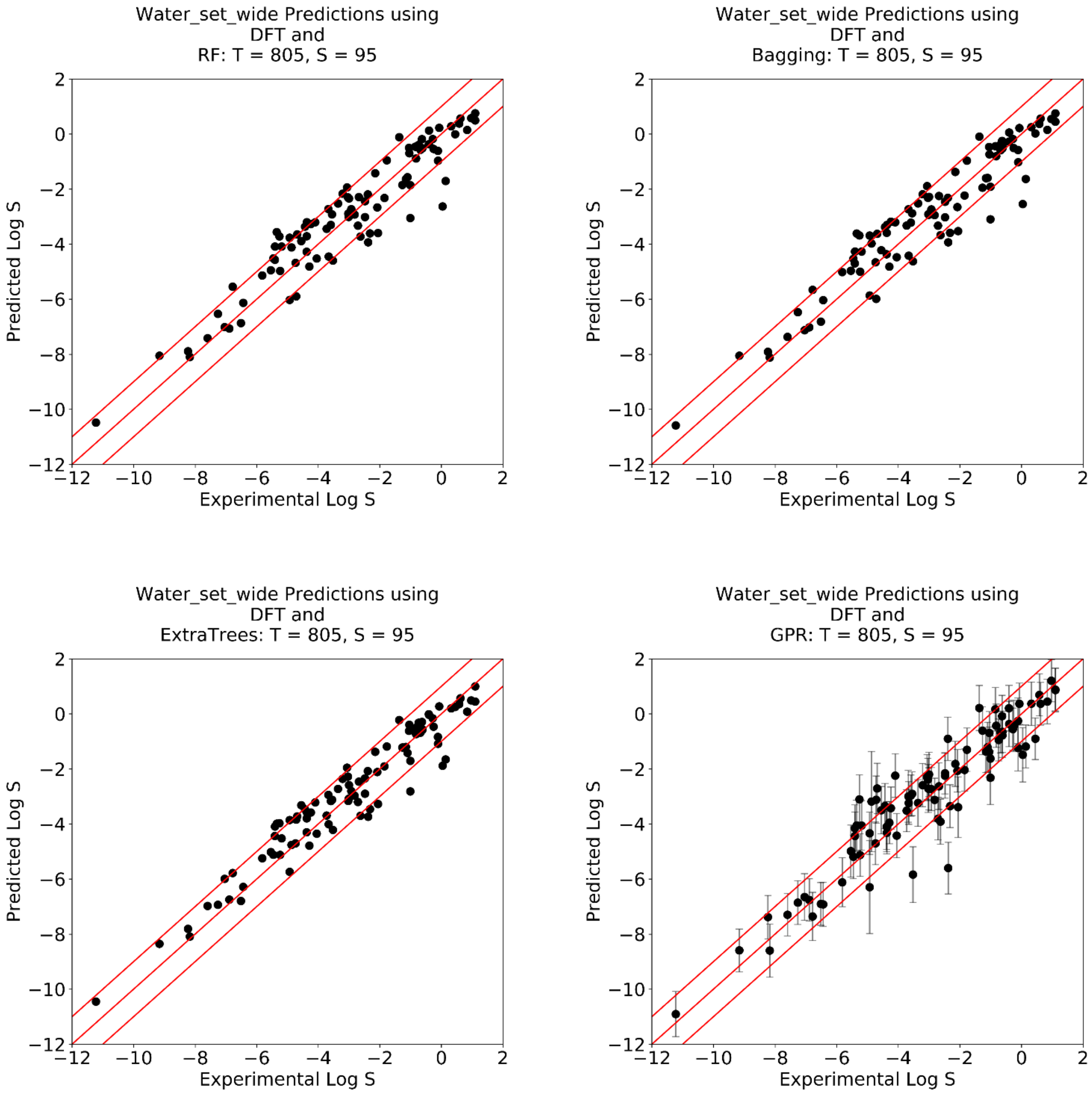

Figure 39. Comparison of seven machine learning methods for ***Water_set_wide***

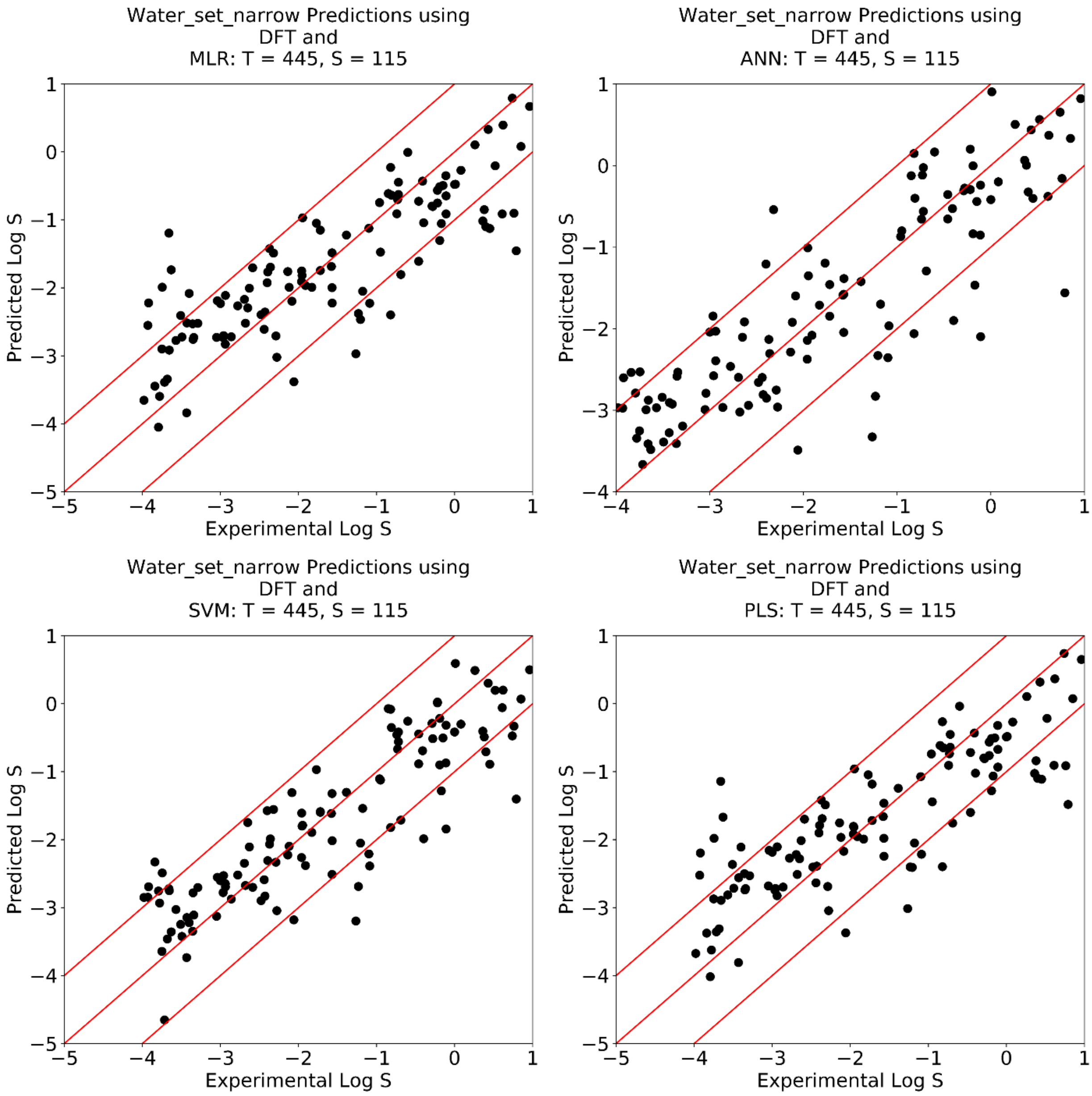
Water_set_narrow Predictions using
DFT and
MLR: T = 445, S = 115
Predicted Log S
Experimental Log S
Water_set_narrow Predictions using
DFT and
ANN: T = 445, S = 115
Predicted Log S
Experimental Log S
Water_set_narrow Predictions using
DFT and
SVM: T = 445, S = 115
Predicted Log S
Experimental Log S
Water_set_narrow Predictions using
DFT and
PLS: T = 445, S = 115
Predicted Log S
Experimental Log S

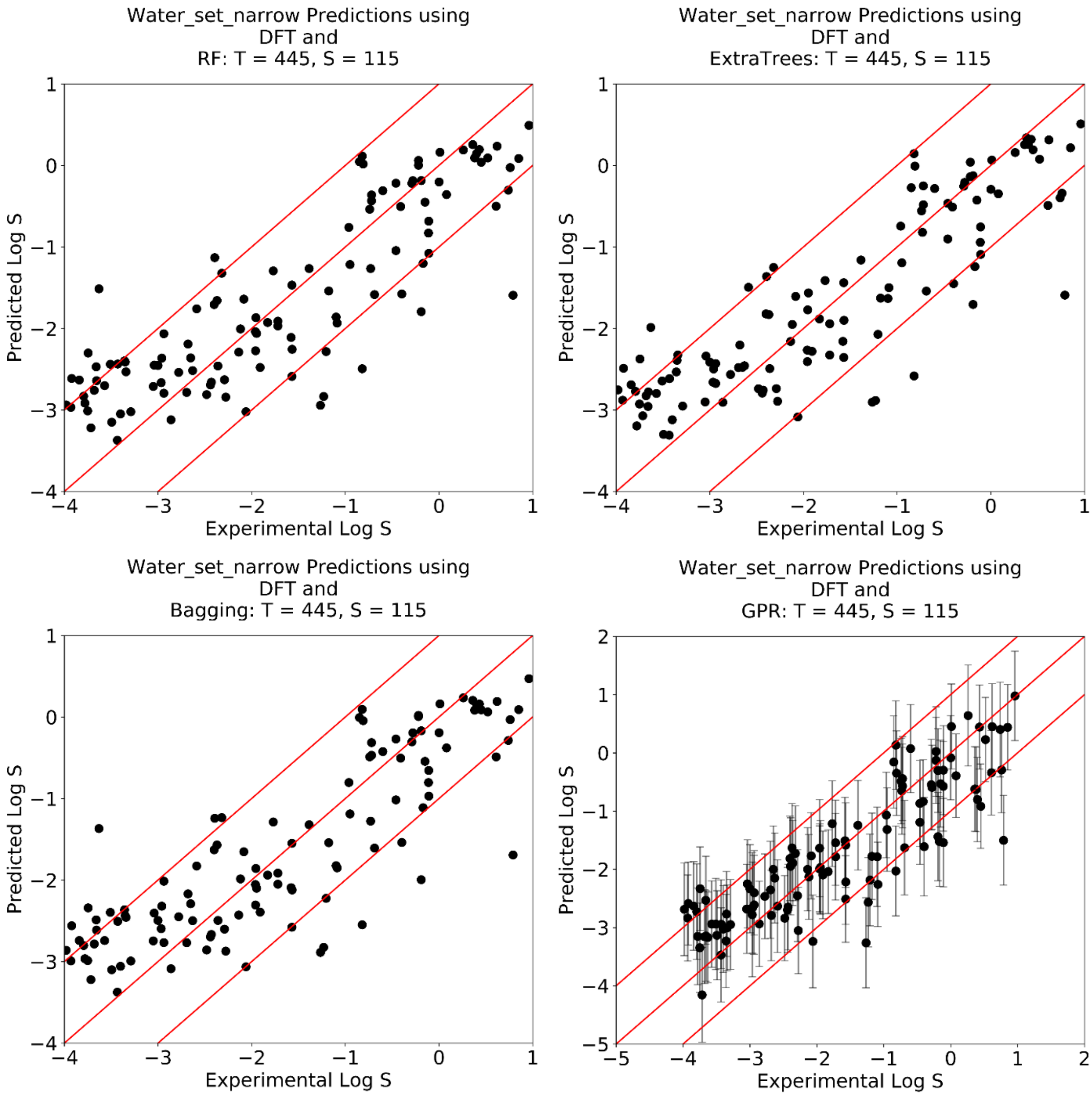

Figure 40. Comparison of seven machine learning methods for ***Water_set_narrow***

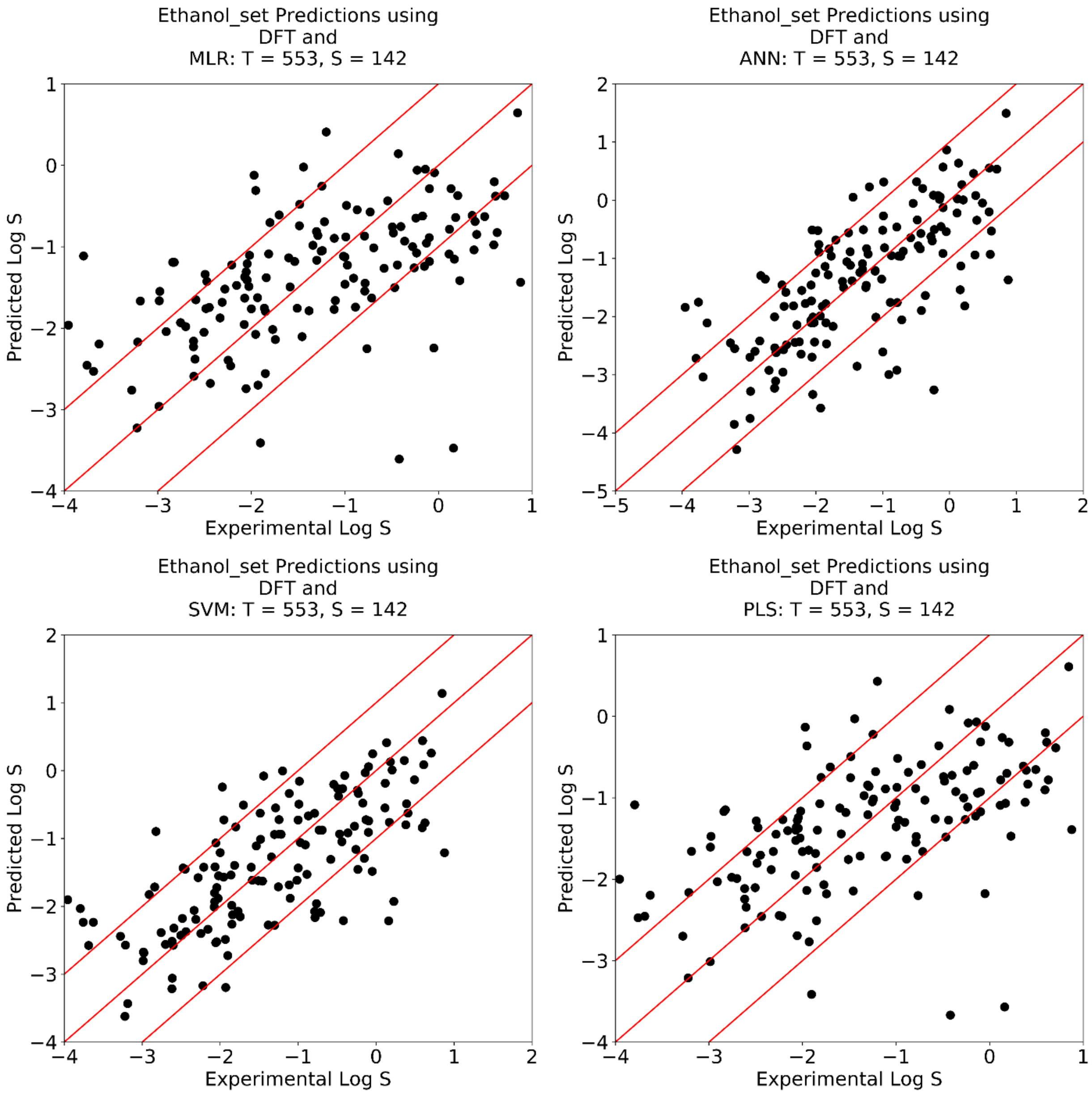
Ethanol_set Predictions using
DFT and
MLR: T = 553, S = 142
Predicted Log S
Experimental Log S
Ethanol_set Predictions using
DFT and
ANN: T = 553, S = 142
Predicted Log S
Experimental Log S
Ethanol_set Predictions using
DFT and
SVM: T = 553, S = 142
Predicted Log S
Experimental Log S
Ethanol_set Predictions using
DFT and
PLS: T = 553, S = 142
Predicted Log S
Experimental Log S

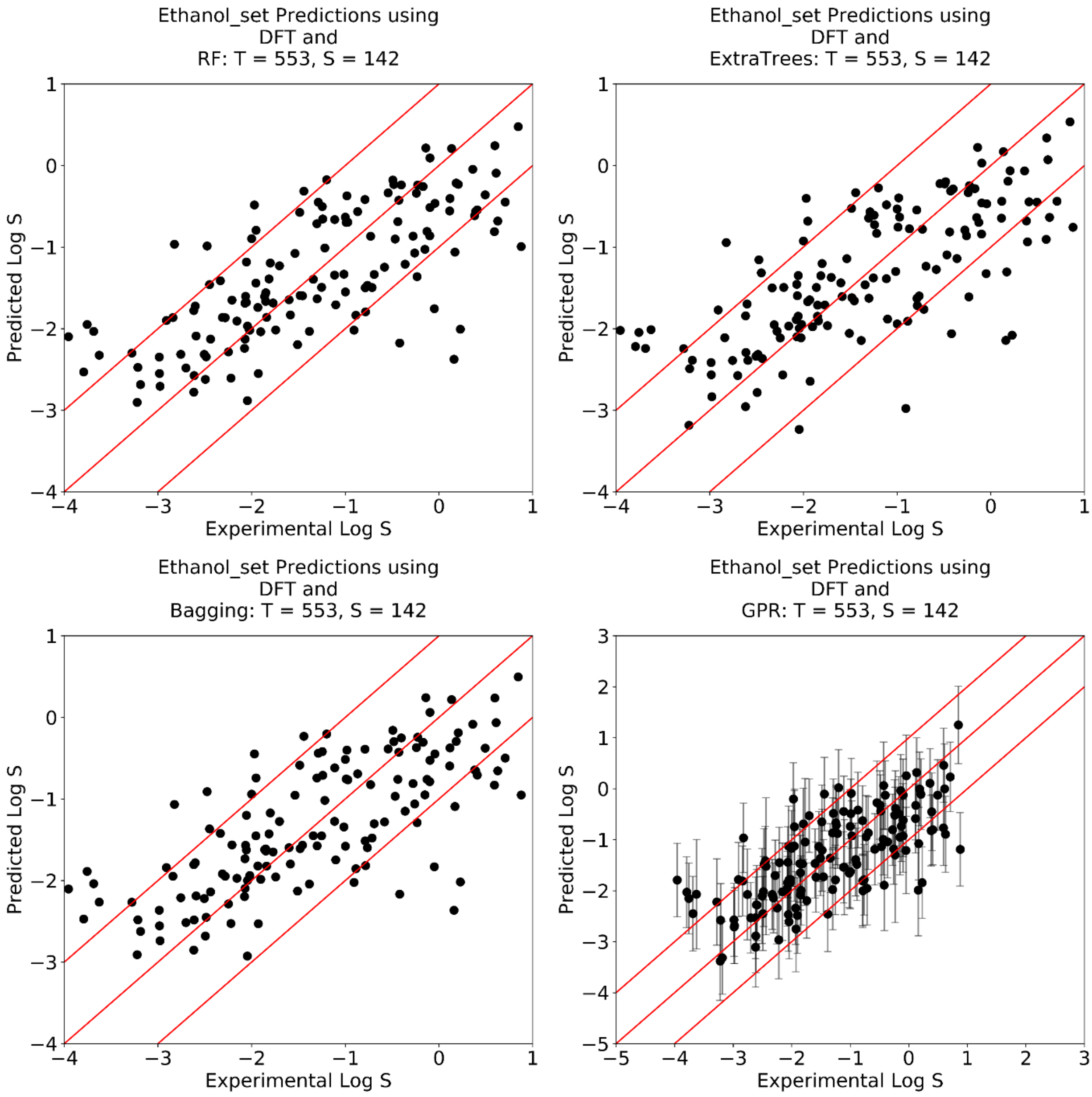

Figure 41. Comparison of seven machine learning methods for ***Ethanol_set***

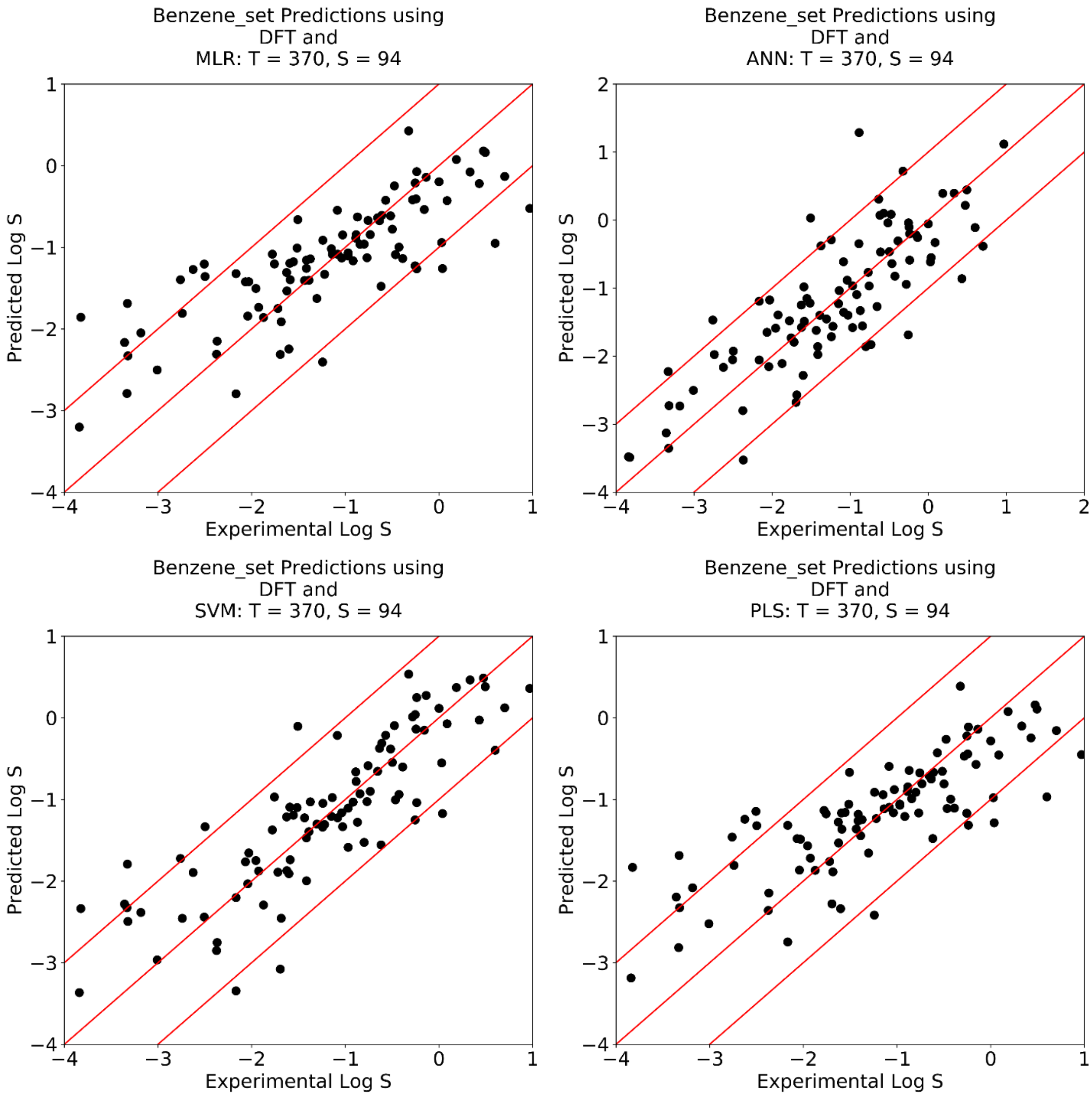
Benzene_set Predictions using
DFT and
MLR: T = 370, S = 94
Predicted Log S
Experimental Log S
Benzene_set Predictions using
DFT and
ANN: T = 370, S = 94
Predicted Log S
Experimental Log S
Benzene_set Predictions using
DFT and
SVM: T = 370, S = 94
Predicted Log S
Experimental Log S
Benzene_set Predictions using
DFT and
PLS: T = 370, S = 94
Predicted Log S
Experimental Log S

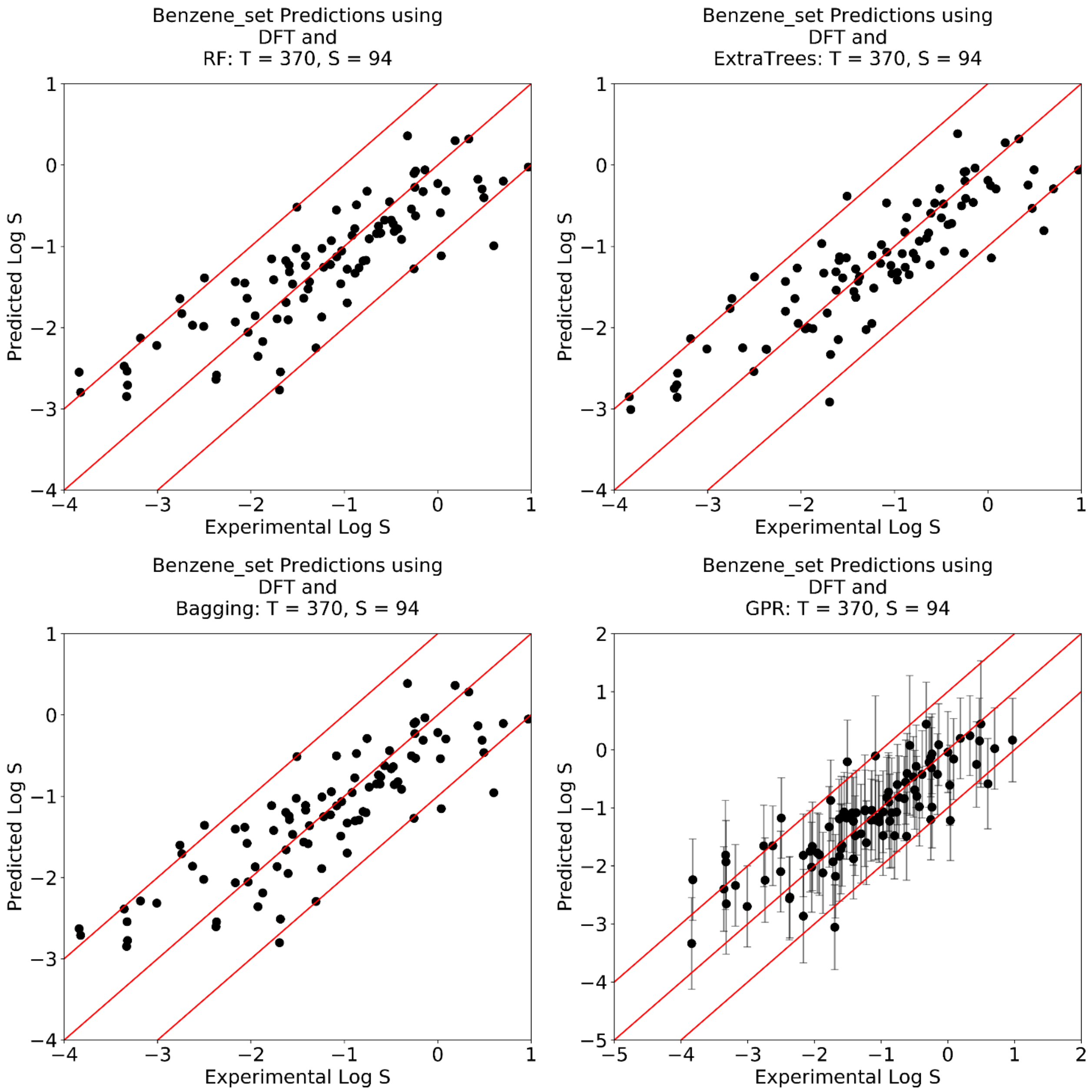

Figure 42. Comparison of seven machine learning methods for ***Benzene_set_wide***

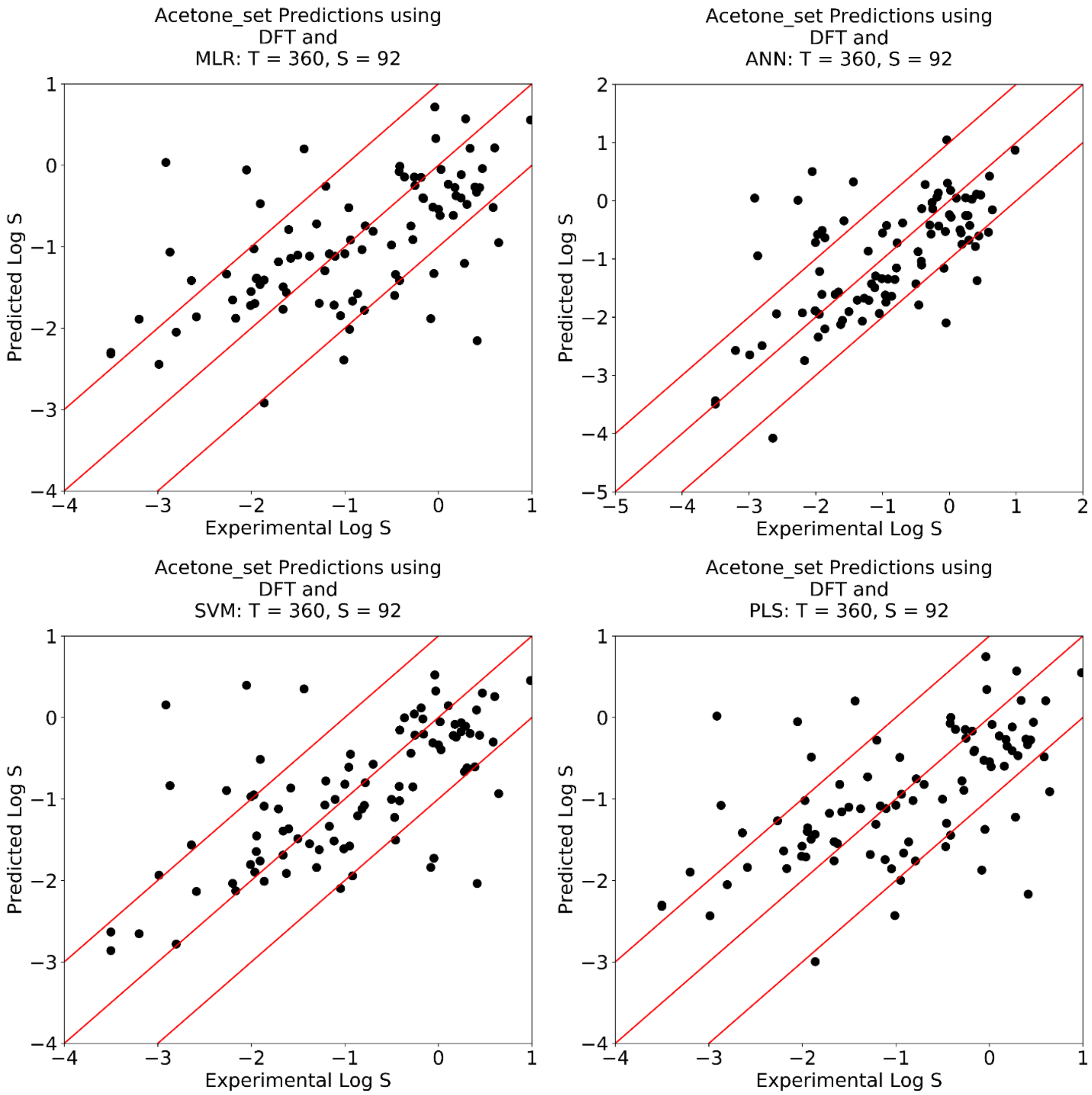
Acetone_set Predictions using
DFT and
MLR: T = 360, S = 92
Predicted Log S
Experimental Log S
Acetone_set Predictions using
DFT and
ANN: T = 360, S = 92
Predicted Log S
Experimental Log S
Acetone_set Predictions using
DFT and
SVM: T = 360, S = 92
Predicted Log S
Experimental Log S
Acetone_set Predictions using
DFT and
PLS: T = 360, S = 92
Predicted Log S
Experimental Log S

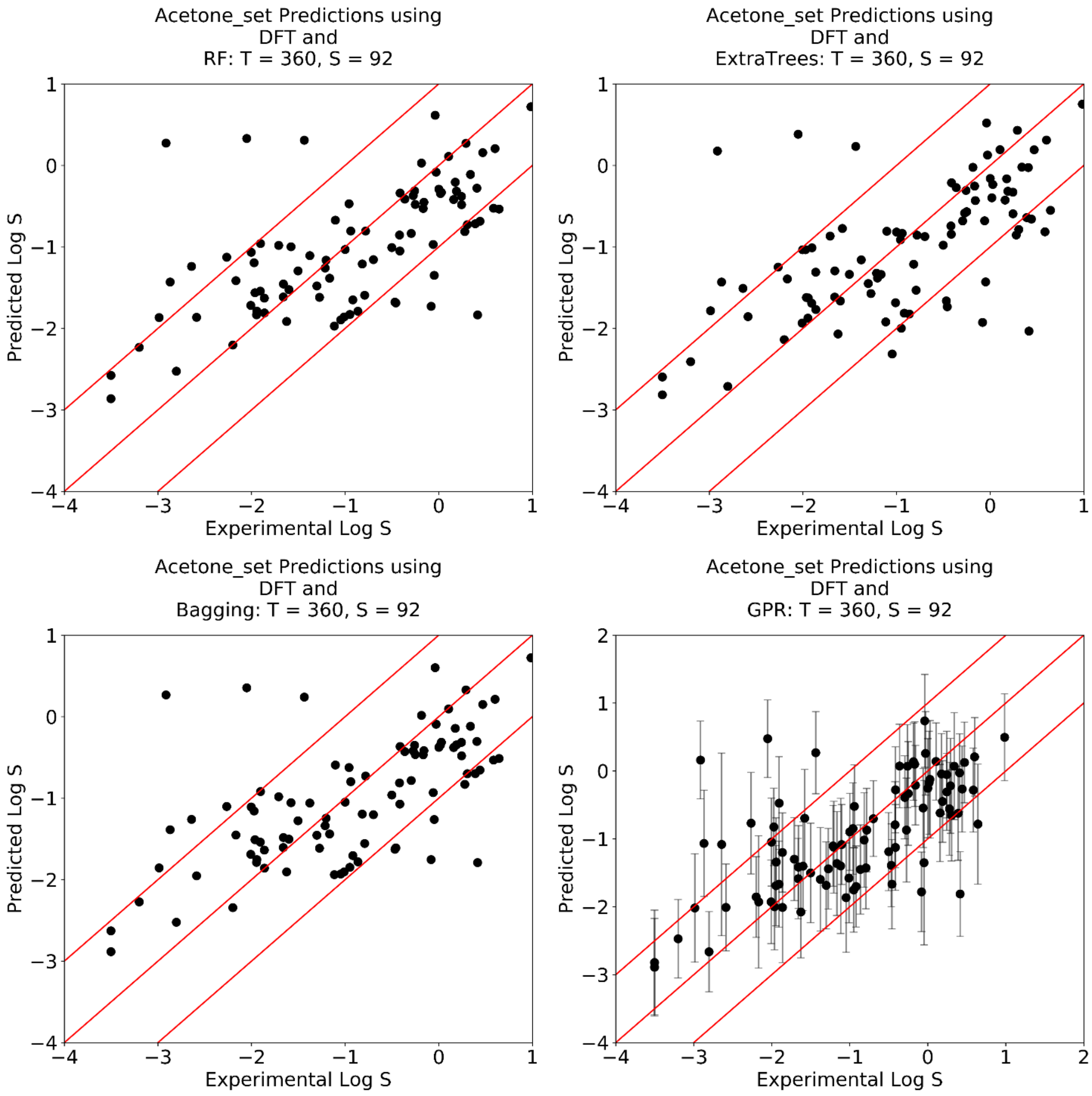

Figure 43. Comparison of seven machine learning methods for ***Acetone_set***

4.7 Effect of solubility range

The comparison between models for ***Water_set_wide*** (LogS range -12 to 2 and -4 to 1) are summarised below in **Figure 44**.

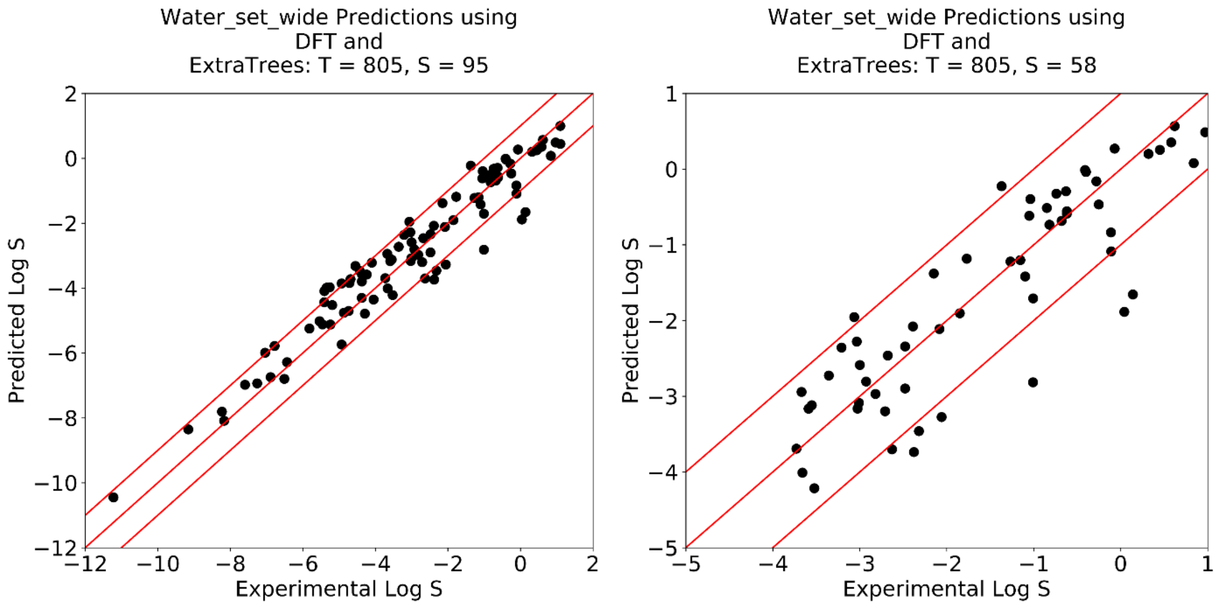

Figure 44. The effect of limiting the solubility range for ***Water_set_wide***

Table 13. Metrics for predictions in the full solubility range and limiting the range to -4 to 1 LogS

Method	R^2	RMSE	%LogS±0.7	%LogS±1.0
Water_set_wide				
MLR	0.80	1.16	50.5	65.3
ANN	0.90	0.84	58.9	78.9
SVM	0.89	0.85	71.6	78.9
PLS	0.80	1.16	51.6	66.3
RF	0.90	0.83	60.0	75.8
ET	0.93	0.71	66.3	84.2
Bag	0.90	0.82	57.9	76.8
GP	0.88	0.89	68.4	73.7
Water_set_wide (limited to -4 to 1 log S range)				
MLR	0.58	1.07	62.1	74.1
ANN	0.73	0.77	67.2	84.5

SVM	0.69	0.87	75.9	77.6
PLS	0.57	1.08	62.1	72.4
RF	0.69	0.82	67.2	81.0
ET	0.76	0.69	72.4	84.5
Bag	0.69	0.81	65.5	81.0
GP	0.68	0.86	72.4	75.9

The correlation between the predictions using 8 different ML methods. Also shown are the mean and median predictions of four ML methods (ET, SVM, ANN and GP), "Consensus_mean" and "Consensus_median". This is shown in **Figure 45** to **Figure 49**. Consensus metrics and more details about consensus predicting can be found in section 4.11.

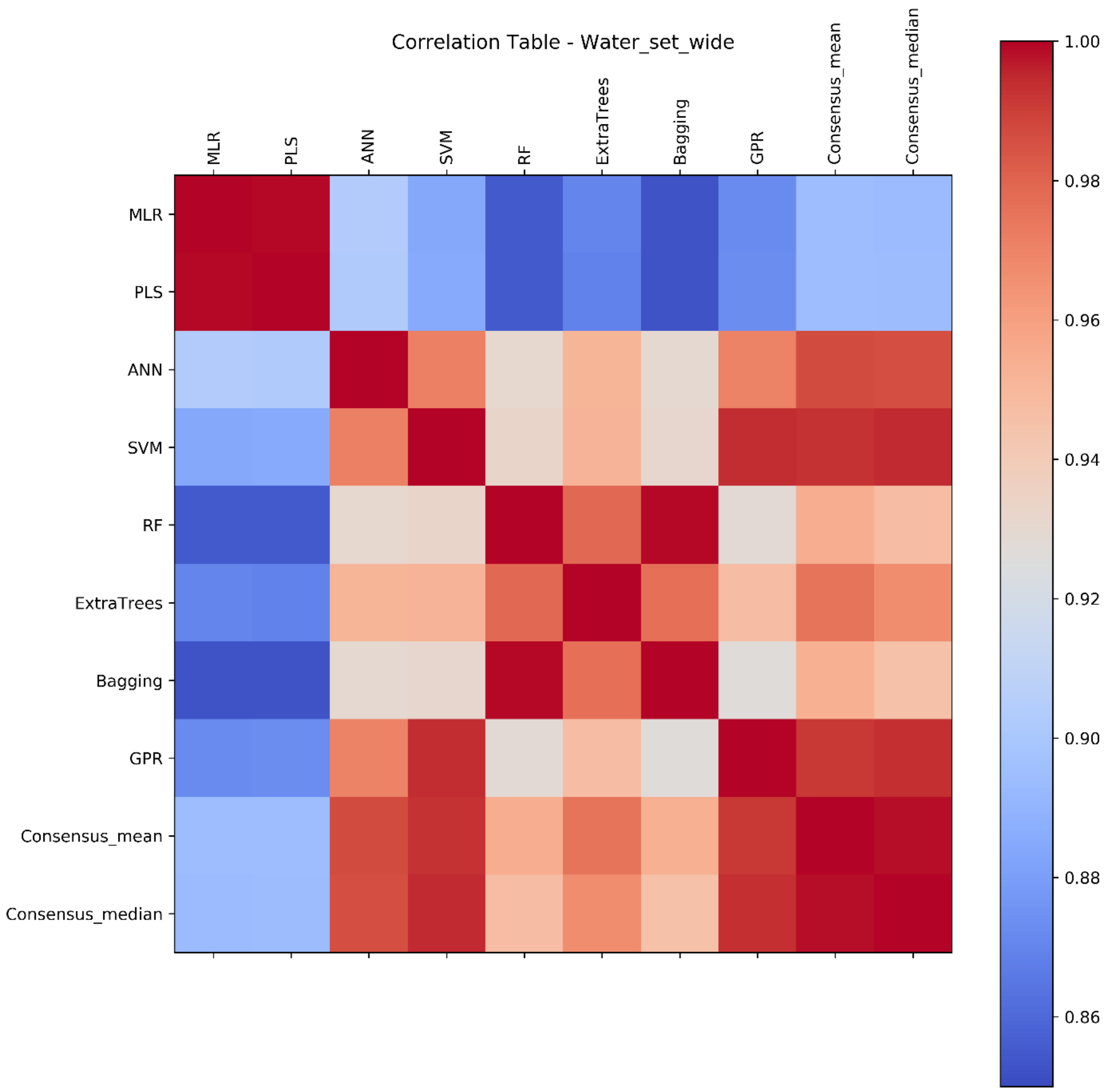

Figure 45. Correlation map for the predictions of internal test sets using different ML methods for ***Water_set_wide***

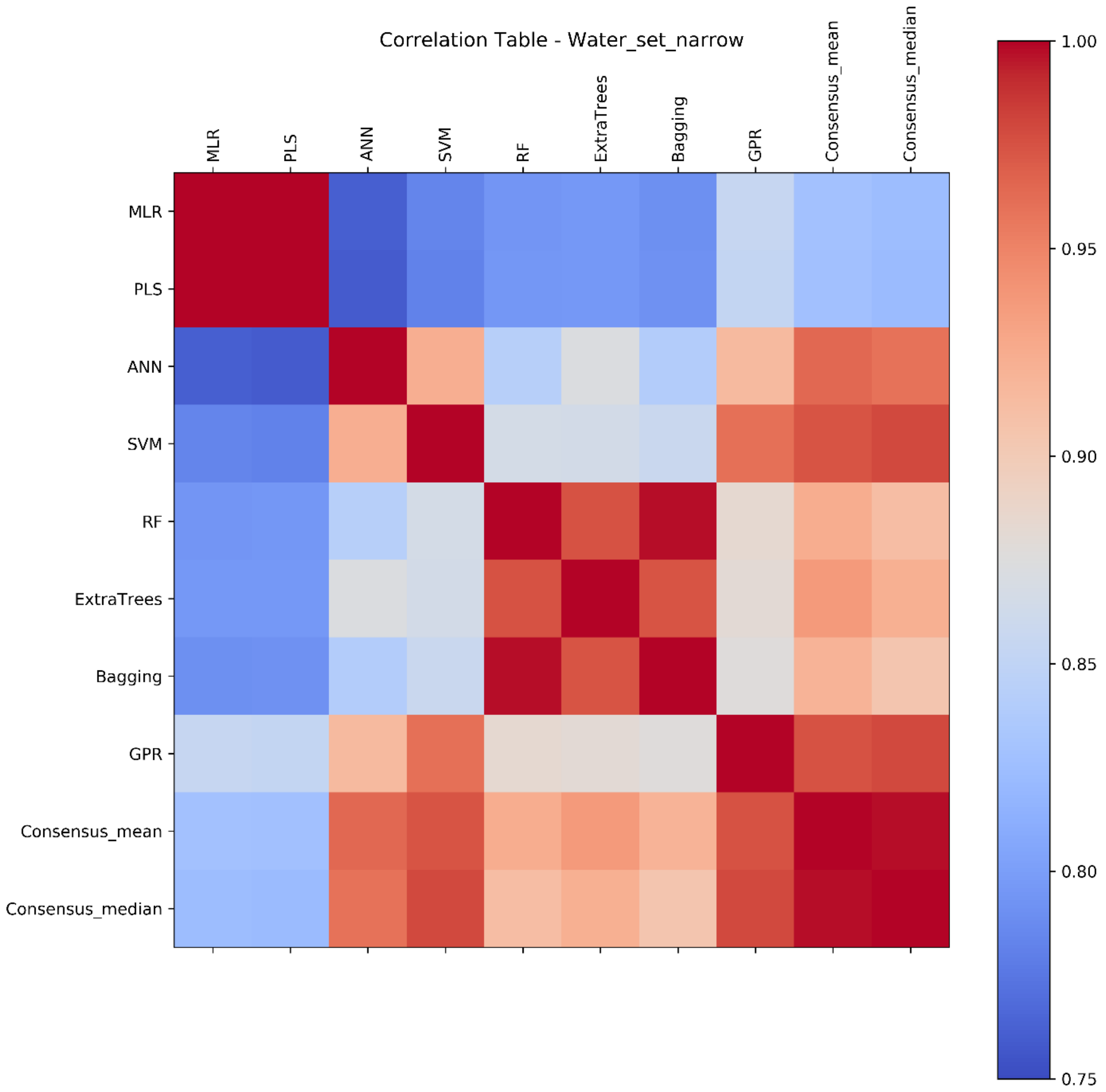

Figure 46. Correlation map for the predictions of internal test sets using different ML methods for ***Water_set_narrow***

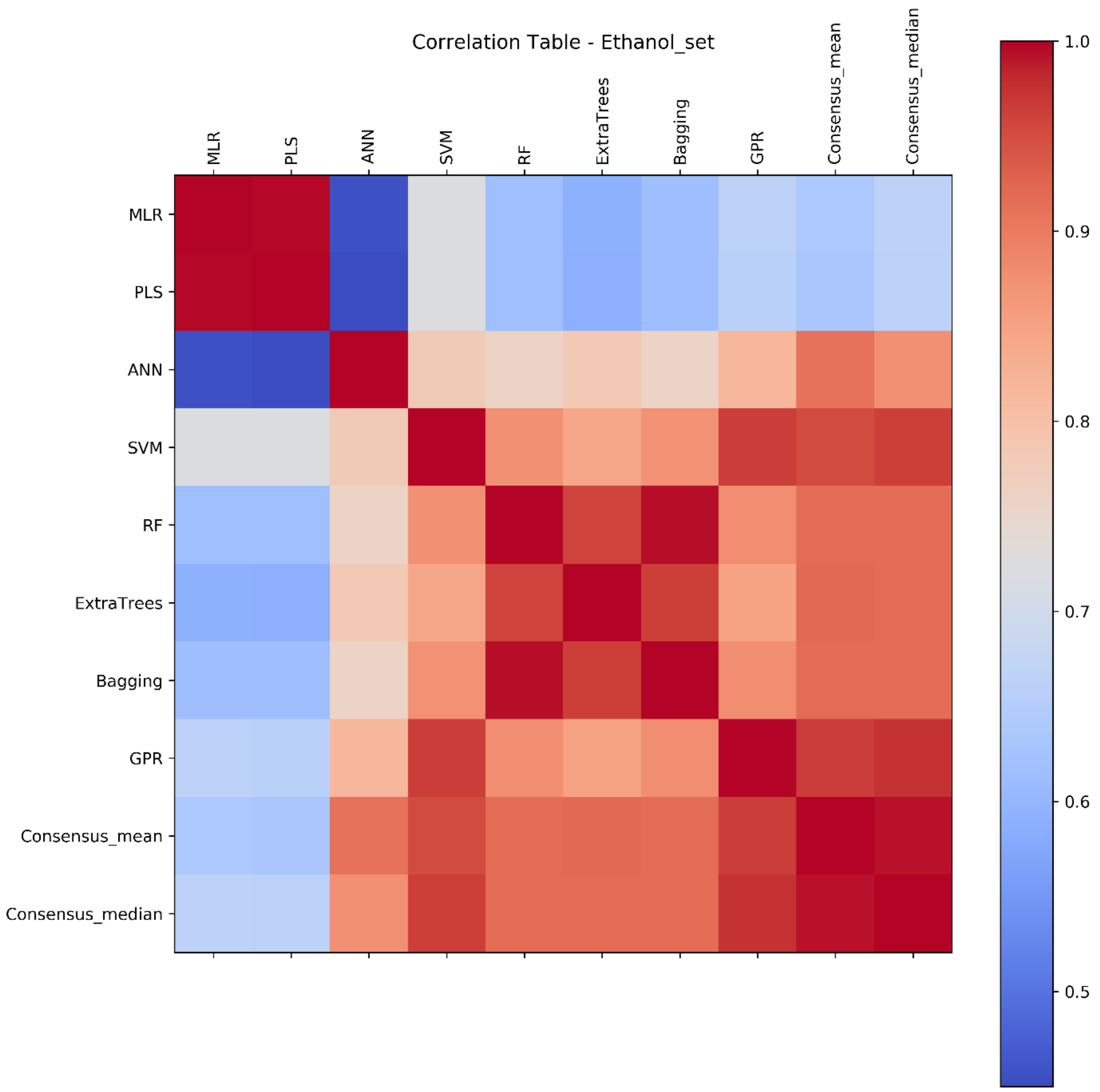

Figure 47. Correlation map for the predictions of internal test sets using different ML methods for ***Ethanol_set***

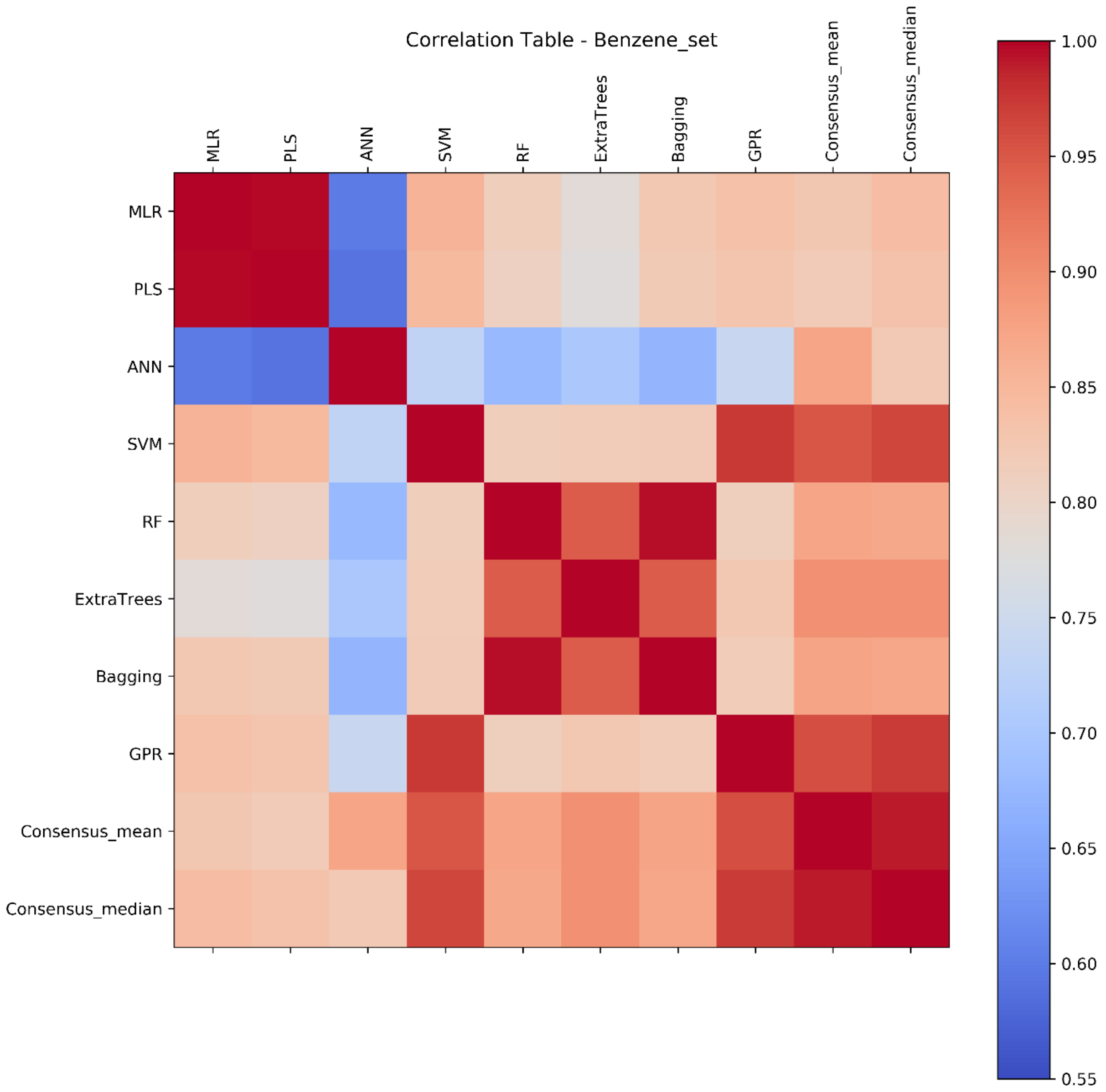

Figure 48. Correlation map for the predictions of internal test sets using different ML methods for ***Benzene_set***

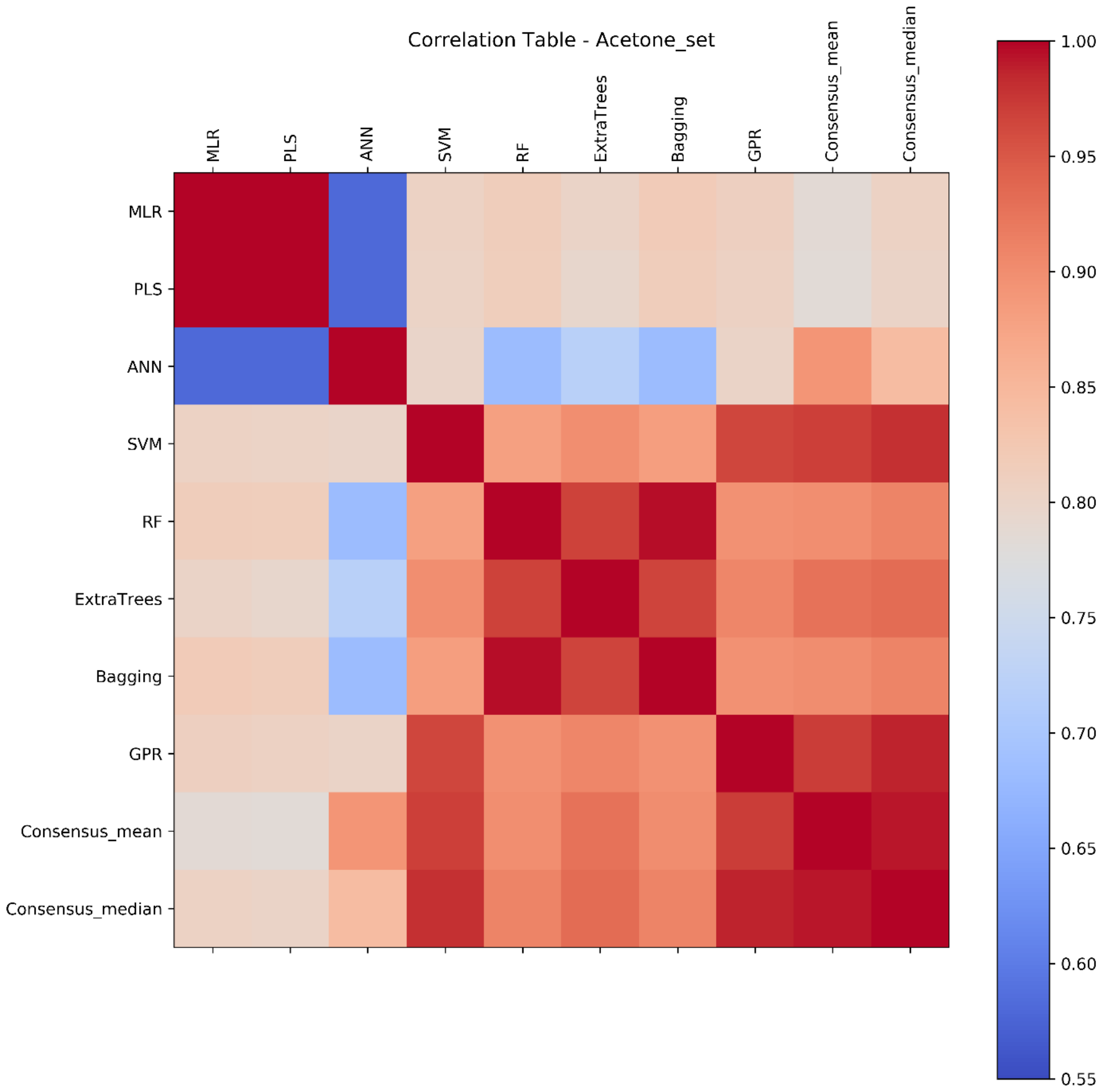

Figure 49. Correlation map for the predictions of internal test sets using different ML methods for ***Acetone_set***

4.8 Refinement of prediction models

4.9 Analysis of outliers

An outlier was defined as a prediction whose error was > 1 LogS. Prediction using ET algorithm were examined. Details are these molecules are found in **Table 14** and **Figure 50** to **Figure 54**.

Table 14. Details of molecules whose prediction error > 1.0 LogS

Dataset	Structure	StdInChIKey/Unique Identifer	SMILES	Error (LogS)
Water_set_wide	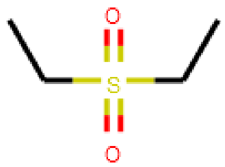	MBDUIEKYVPVZJH-UHFFFAOYSA-N	S(=O)(=O)(CC)CC	1.92
Water_set_wide	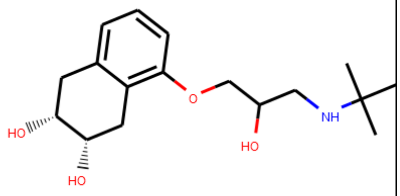	VWPOSFSPZNDTMJ-UCWKZMIHSA-N	O(CC(O)CNC(C)(C)C)c1c2c(ccc1)C[C@@H](O)[C@@H](O)C2	1.81
Water_set_wide	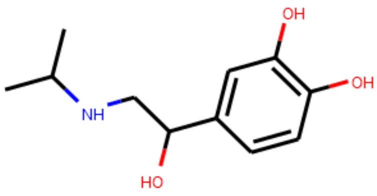	JWZZKOKVBUJMES-UHFFFAOYSA-N	OC(CNC(C)C)c1cc(O)c(O)cc1	1.80
Water_set_wide	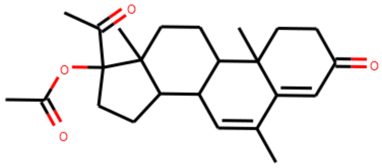	RQZAXGRLVPAYTJ-UHFFFAOYSA-N	O=C(OC1(C(=O)C)C2(C)C(C3C=C(C)C4=CC(=O)CCC4(C)C3CC2)CC1)C	1.36
Water_set_wide	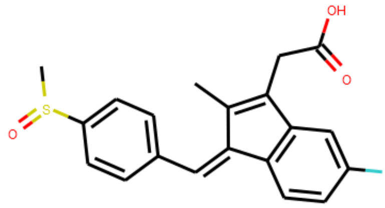	MLKXDPUZXIRXEP-RQZCQDPDSA-N	S(=O)(C)c1ccc(/C=C/2\C(=C(CC(=O)O)c3c2ccc(F)c3)C)cc1	1.36
Water_set_wide	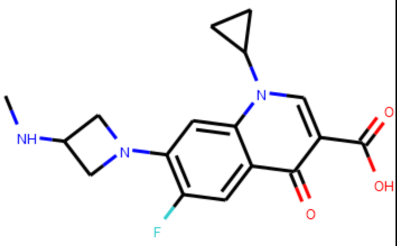	XDLPWPNVUPKKSN-UHFFFAOYSA-N	Fc1c(N2CC(NC)C2)cc2n(C3CC3)cc(C(=O)O)c(=O)c2c1	1.31

Water_set_wide	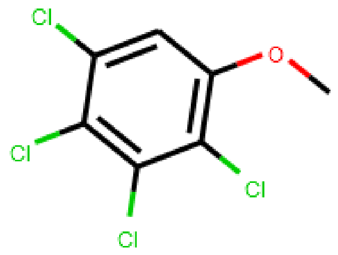	FUUHMSUPRUNWRQ -UHFFFAOYSA-N	Clc1c(Cl)c(Cl)cc(OC)c1Cl	1.29
Water_set_wide	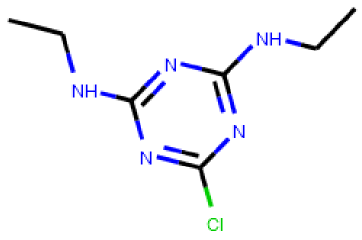	ODCWYMIRDDJXKW -UHFFFAOYSA-N	Clc1nc(NCC)nc(NCC)n1	1.23
Water_set_wide	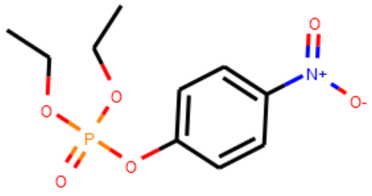	WYMSBXTXOHUIGT-UHFFFAOYSA-N	P(=O)(OCC)(OCC)Oc1ccc([N+](=O)[O-])cc1	1.21
Water_set_wide	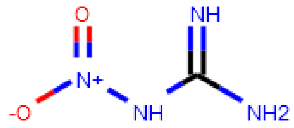	IDCPFAYURAQKDZ-UHFFFAOYSA-N	O=[N+]([O-])NC(=N)N	1.15
Water_set_wide	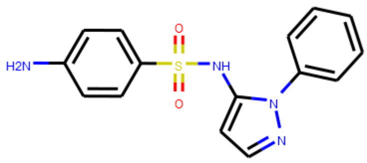	QWCJHSGMANYXCW -UHFFFAOYSA-N	S(=O)(=O)(Nc1n(c2ccccc2)ncc1)c1ccc(N)cc1	1.19
Water_set_wide	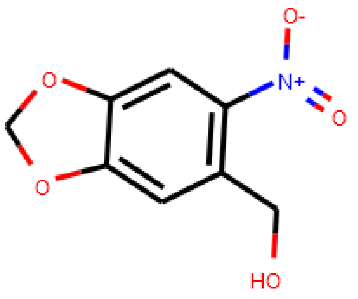	XSKQKDTZQNFCCB-UHFFFAOYSA-N	O=[N+]([O-])c1c(CO)cc2OCOc2c1	1.11

Water_set_wide	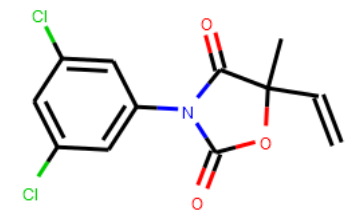	FSCWZHGZWWDELK-UHFFFAOYSA-N	Clc1cc(Cl)cc(N2C(=O)C(C=C)(C)OC2=O)c1	1.08
Water_set_wide	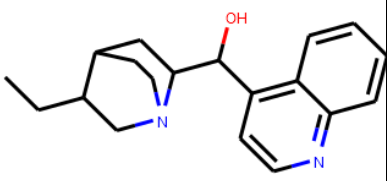	WFJNHVWTKZUUTR-UHFFFAOYSA-N	OC(c1c2c(ncc1)cccc2)C1N2CC(CC)C(C1)CC2	1.07
Water_set_wide	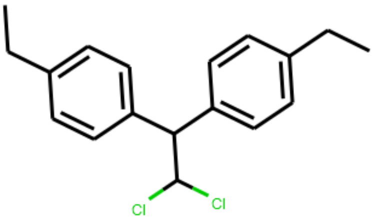	QFMDFTQOJHFVNR-UHFFFAOYSA-N	ClC(Cl)C(c1ccc(CC)cc1)c1ccc(CC)cc1	1.05
Water_set_narrow	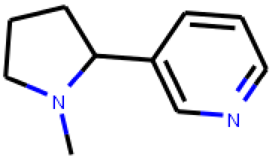	SNICXCGAKADSCV-UHFFFAOYSA-N	CN1C(c2cnccc2)CCC1	2.38
Water_set_narrow	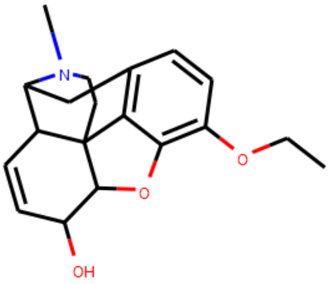	OGDVEMNWJVYAJL-UHFFFAOYSA-N	O(CC)c1c2OC3C(O)C=CC4C53c2c(cc1)CC4N(C)CC5	1.76
Water_set_narrow	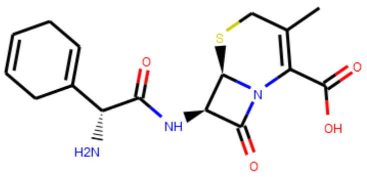	RDLPVSKMFDYCOR-UEKVPHQBSA-N	O=C(N[C@@H]1C(=O)N2C(=C(C)CS[C@H]12)C(=O)O)[C@H](N)C1=CCC=CC1	1.65

Water_set_narrow	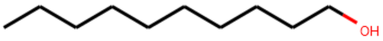	MWKFXSUHUHTGQN-UHFFFAOYSA-N	OCCCCCCCCCC	1.64
Water_set_narrow	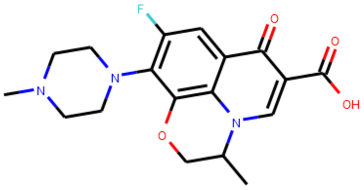	GSDSWSVVBLHKDQ-UHFFFAOYSA-N	Fc1c(N2CCN(C)CC2)c2OCC(C)n3c2c(c(=O)c(C(=O)O)c3)c1	1.64
Water_set_narrow	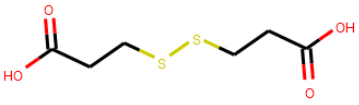	YCLSOMLVSHPPFV-UHFFFAOYSA-N	S(SCCC(=O)O)CCC(=O)O	1.51
Water_set_narrow	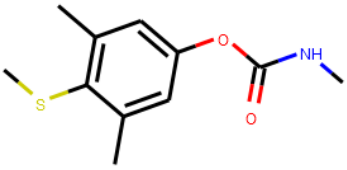	YFBPRJGDJKVWAH-UHFFFAOYSA-N	S(C)c1c(C)cc(OC(=O)NC)cc1C	1.43
Water_set_narrow	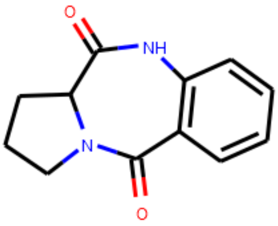	MXBNEEHQIDLPLQ-UHFFFAOYSA-N	O=C1N2C(C(=O)Nc3c1cccc3)CCC2	1.37
Water_set_narrow	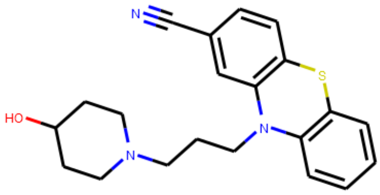	LUALIOATIOESLM-UHFFFAOYSA-N	OC1CCN(CCCN2c3c(Sc4c2cccc4)ccc(C#N)c3)CC1	1.23
Water_set_narrow	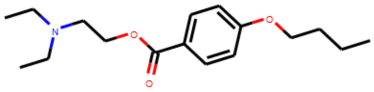	QNIUOGIMJWORNZ-UHFFFAOYSA-N	O=C(OCCN(CC)CC)c1ccc(OCCCC)cc1	1.15

Water_set_narrow	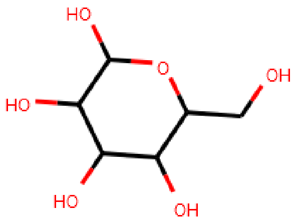	WQZGKKKJIJFFOK-UHFFFAOYSA-N	OCC1C(O)C(O)C(O)C(O)O1	1.14
Water_set_narrow	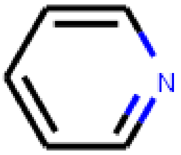	JUJWROOIHBZHMG-UHFFFAOYSA-N	n1ccccc1	1.10
Water_set_narrow	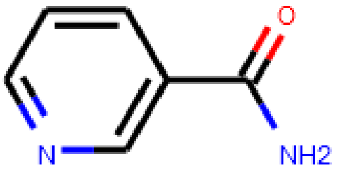	DFPAKSUCGFBDDF-UHFFFAOYSA-N	O=C(N)c1cnccc1	1.10
Water_set_narrow	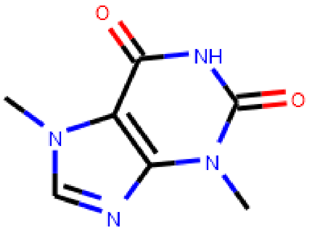	YAPQBXQYLJRXSA-UHFFFAOYSA-N	O=c1n(C)c2ncn(C)c2c(=O)[nH]1	1.09
Water_set_narrow	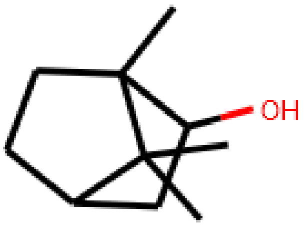	DTGKSKDOIYIVQL-UHFFFAOYSA-N	OC1C2(C)C(C)(C)C(C1)CC2	1.07

Water_set_narrow	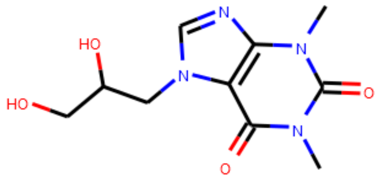	KSCFJBIXMNOVSH-UHFFFAOYSA-N	O=c1n(C)c(=O)c2n(CC(O)CO)cnc2n1C	1.07
Water_set_narrow	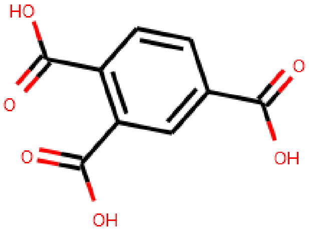	ARCGXLSVLAOJQL-UHFFFAOYSA-N	O=C(O)c1c(C(=O)O)ccc(C(=O)O)c1	1.06
Water_set_narrow	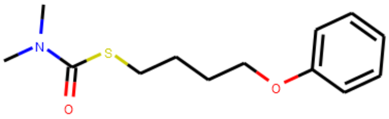	HMIBKHHNXANVHR-UHFFFAOYSA-N	S(C(=O)N(C)C)CCCCOc1ccccc1	1.05
Water_set_narrow	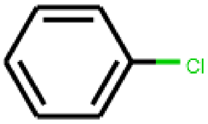	MVPPADPHJFYWMZ-UHFFFAOYSA-N	Clc1ccccc1	1.03
Water_set_narrow	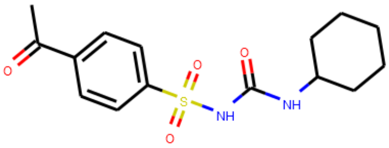	VGZSUPCWNCWDAN-UHFFFAOYSA-N	S(=O)(=O)(NC(=O)NC1CCCCC1)c1ccc(C(=O)C)cc1	1.02
Water_set_narrow	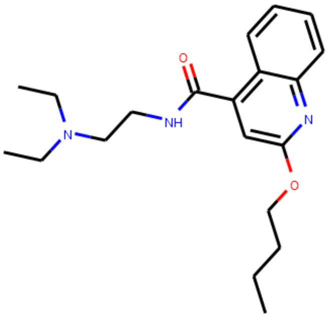	PUFQVTATUTYEAL-UHFFFAOYSA-N	O=C(NCCN(CC)CC)c1c2c(nc(OCCCC)c1)cccc2	1.02
Water_set_narrow	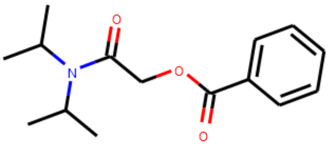	ZWUAUTPCFIIFAS-UHFFFAOYSA-N	O=C(N(C(C)C)C(C)C)COC(=O)c1ccccc1	1.02

Ethanol_set	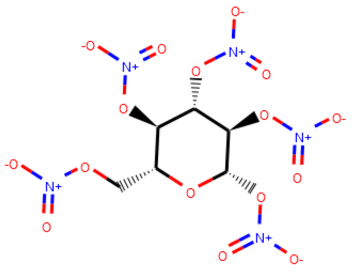	TWRPLCZREDAZEZ-DVKNGEFBSA-N	C([C@@H]1[C@H]([C@@H]([C@H]([C@@H](O1)O[N+](=O)[O-])O[N+](=O)[O-])O[N+](=O)[O-])O[N+](=O)[O-])O[N+](=O)[O-]	2.31
Ethanol_set	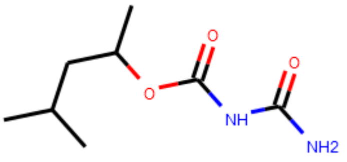	RENUKYOICMBIKE-UHFFFAOYSA-N	CC(C)CC(C)OC(=O)NC(=O)N	2.30
Ethanol_set	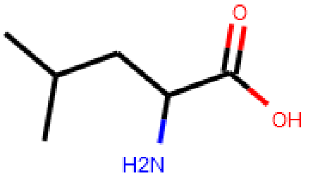	ROHFNLRQFUQHCH-UHFFFAOYSA-N	CC(C)CC(C(=O)O)N	2.07
Ethanol_set	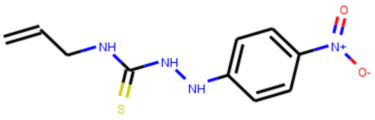	RUBNOJFDOZNHSQ-UHFFFAOYSA-N	C=CCNC(=S)NNc1ccc(cc1)[N+](=O)[O-]	1.94
Ethanol_set	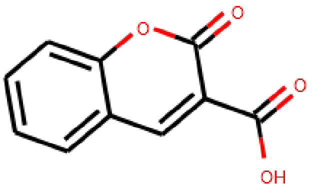	ACMLKANOGIVEPB-UHFFFAOYSA-N	c1ccc2c(c1)cc(C(=O)O)c(=O)o2	1.88
Ethanol_set	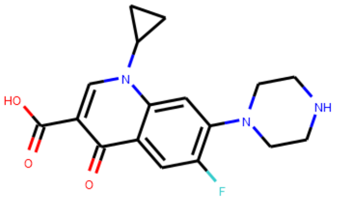	MYSWGUAQZAJSOK-UHFFFAOYSA-N	C1CC1n1cc(c(=O)c2cc(c(cc12)N1CCNCC1)F)C(=O)O	1.71

Ethanol_set	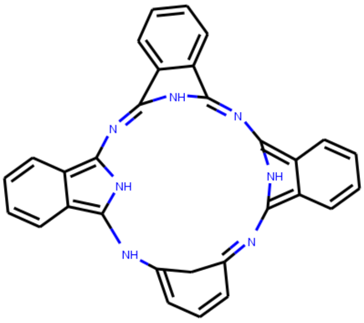	KJKGTLBNWUUCKQ-UHFFFAOYSA-N	c1ccc2c(c1)c1NC3=CC=CC(=Nc4c5ccccc5c([nH]4)N=c4c5ccccc5c(=Nc2[nH]1)[nH]4)C3	1.64
Ethanol_set	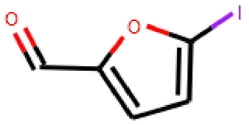	QPGPCPKDVSPJAY-UHFFFAOYSA-N	c1cc(I)oc1C=O	1.63
Ethanol_set	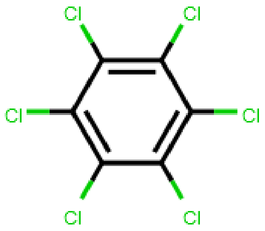	CKAPSXZOOQJIBF-UHFFFAOYSA-N	c1(c(c(c(c(c1Cl)Cl)Cl)Cl)Cl)Cl	1.62
Ethanol_set	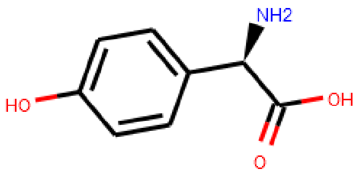	LJCWONGJFPCTTL-SSDOTTSWSA-N	c1cc(ccc1[C@H](C(=O)O)N)O	1.58
Ethanol_set	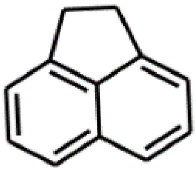	CWRYPZZKDGJXCA-UHFFFAOYSA-N	c1cc2cccc3CCc(c1)c23	1.57
Ethanol_set	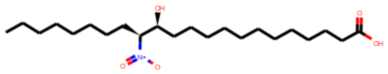	RJUJQBSIFOSCCE-SFTDATJTSA-N	CCCCCCCC[C@@H]([C@H](CCCCCCCCCCCC(=O)O)O)N(=O)=O	1.50
Ethanol_set	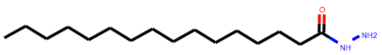	SSVSELJXJJCANX-UHFFFAOYSA-N	CCCCCCCCCCCCCCCC(=O)NN	1.47

Ethanol_set	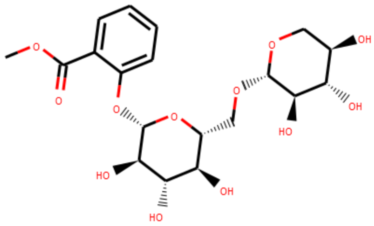	VHUNCYDAXJGCLO-AHMNSWSSSA-N	COC(=O)c1ccccc1O[C@H]1[C@@H]([C@H]([C@@H]([C@@H](CO[C@H]2[C@@H]([C@H]([C@@H](CO2)O)O)O)O1)O)O)O	1.45
Ethanol_set	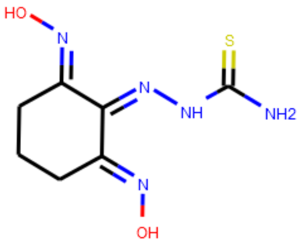	YZIXRDWVUVBLFL-JLYPQOKGSA-N	C1C/C(=N\O)/C(=N/NC(=S)N)/C(=N/O)/C1	1.38
Ethanol_set	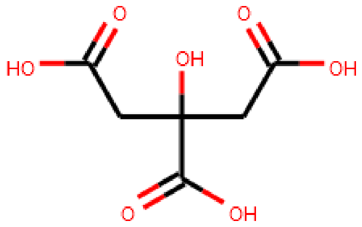	KRKNYBCHXYNGOX-UHFFFAOYSA-N	C(C(=O)O)C(CC(=O)O)(C(=O)O)O	1.33
Ethanol_set	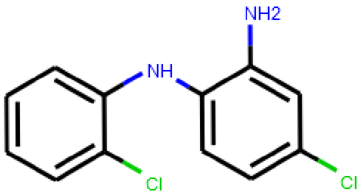	UTQSUDKKBZEXHH-UHFFFAOYSA-N	c1ccc(c(c1)Cl)Nc1ccc(cc1N)Cl	1.32
Ethanol_set	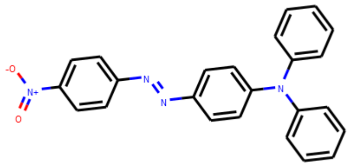	SEHYCGWJONNDKG-OCEACIFDSA-N	c1ccc(cc1)N(c1ccccc1)c1ccc(cc1)/N=N/c1ccc(cc1)N(=O)=O	1.27
Ethanol_set	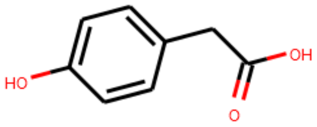	ons21	c1cc(ccc1CC(=O)O)O	1.27

Ethanol_set	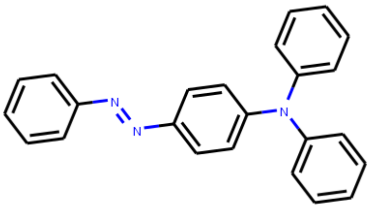	QRBSSDMZLRKOGR-OCEACIFDSA-N	c1ccc(cc1)N=Nc1ccc(cc1)N(c1ccccc1)c1ccccc1	1.26
Ethanol_set	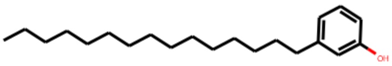	PTFIPECGHSYQNR-UHFFFAOYSA-N	CCCCCCCCCCCCCCCc1cccc(c1)O	1.19
Ethanol_set	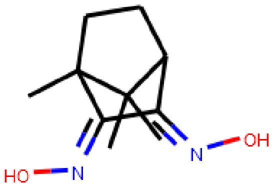	JCFJSQZXNOMXCP-UHFFFAOYSA-N	CC1(C)C2CCC1(C)C(=NO)C2=NO	1.15
Ethanol_set	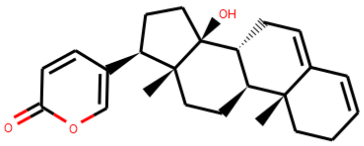	KBOQXVVZFSWICE-FIJLMTKSA-N	C[C@]12CCC=CC1=CC[C@@H]1[C@@H]2CC[C@]2(C)[C@H](CC[C@]12O)c1ccc(=O)oc1	1.14
Ethanol_set	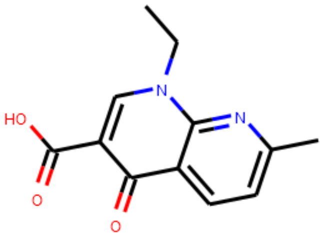	MHWLWQUZZRMNGJ-UHFFFAOYSA-N	CCn1cc(c(=O)c2ccc(C)nc12)C(=O)O	1.14
Ethanol_set	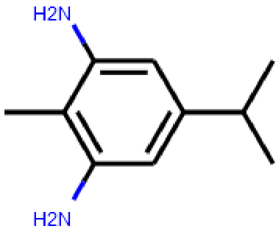	LPHRUDILDCGDJM-UHFFFAOYSA-N	CC(C)c1cc(c(C)c(c1)N)N	1.11
Ethanol_set	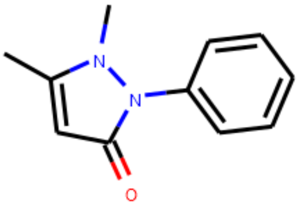	VEQOALNAAJBPNY-UHFFFAOYSA-N	Cc1cc(=O)n(c2ccccc2)n1C	1.08

Ethanol_set	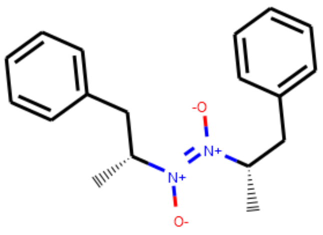	JWBYUXWEHYCVHA-BVYOUEPQSA-N	C[C@@H](Cc1ccccc1)[N](=O)=[N](=O)[C@H](C)Cc2ccccc2	1.06
Ethanol_set	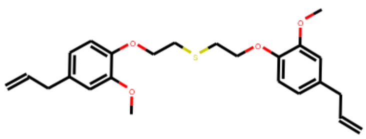	RTOHDISBHWDUCE-UHFFFAOYSA-N	C=CCc1ccc(c(c1)OC)OCCSCCOc1ccc(CC=C)cc1OC	1.05
Ethanol_set	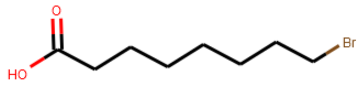	ons25	C(CCCC(=O)O)CCCBr	1.03
Ethanol_set	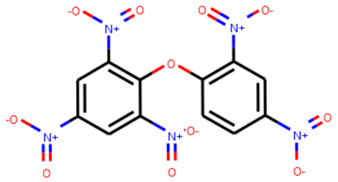	UCAHGXFXBRSTPX-UHFFFAOYSA-N	c1cc(c(cc1[N+](=O)[O-])[N+](=O)[O-])Oc1c(cc(cc1[N+](=O)[O-])[N+](=O)[O-])[N+](=O)[O-]	1.02
Benzene_set	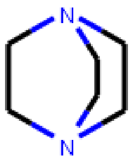	IMNIMPAHZVJRPE-UHFFFAOYSA-N	C1CN2CCN1CC2	1.41
Benzene_set	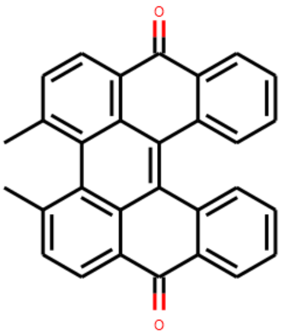	IJFDOVWXJGQCGY-UHFFFAOYSA-N	Cc1ccc2C(=O)c3ccccc3c4c2c1c5c(C)ccc6C(=O)c7ccccc7c4c56	1.22

Benzene_set	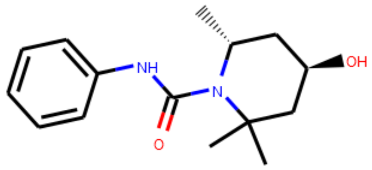	KXGNFVJBSLJUTL-DGCLKSJQSA-N	c1ccc(cc1)NC(=O)N1C(C[C@@H](C[C@H]1C)O)(C)C	1.18
Benzene_set	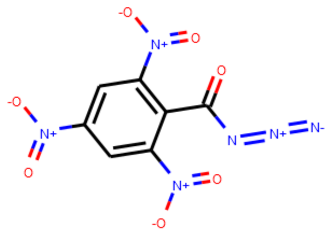	YDCFNPFXXJRYCZ-UHFFFAOYSA-N	[O-][N+](=O)c1cc(c(C(=O)N=[N+]=[N-])c(c1)[N+]([O-])=O)[N+]([O-])=O	1.13
Benzene_set	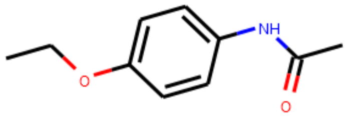	CPJSUEIXXCENMM-UHFFFAOYSA-N	CCOc1ccc(NC(C)=O)cc1	1.12
Benzene_set	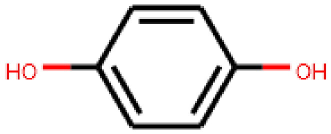	QIGBRXMKCJKVMJ-UHFFFAOYSA-N	Oc1ccc(O)cc1	1.10
Benzene_set	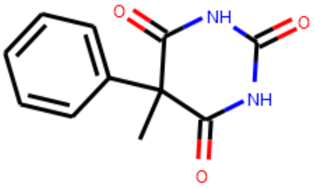	LSAOZCAKUIANSQ-UHFFFAOYSA-N	CC1(C(=O)NC(=O)NC1=O)c2ccccc2	1.05
Benzene_set	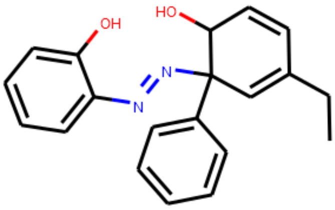	CTVADTSNUUHIPU-FOCLMDBBSA-N	CCC1=CC(N=Nc2ccccc2O)(C(O)C=C1)c3ccccc3	1.03

Benzene_set	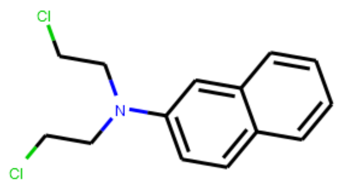	XCDXSSFOJZZGQC-UHFFFAOYSA-N	ClCCN(CCCl)c1ccc2ccccc2c1	1.01
Acetone_set	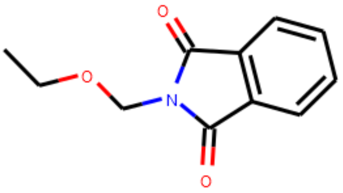	IMOPXBPHKARACH-UHFFFAOYSA-N	CCOCN1C(=O)C2=C(C=CC=C2)C1=O	3.09
Acetone_set	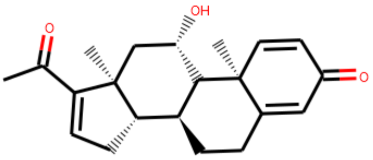	UVOTVQIUSZDLAM-GGLFOPPFSA-N	[H][C@@]12CC=C(C(C)=O)[C@@]1(C)C[C@H](O)[C@@]1([H])[C@@]2([H])CCC2=CC(=O)C=C[C@]12C	2.45
Acetone_set	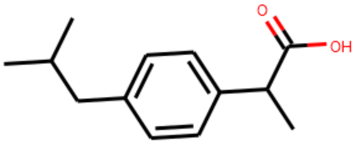	HEFNNWSXXWATRW-UHFFFAOYSA-N	CC(C)CC1=CC=C(C=C1)C(C)C(O)=O	2.44
Acetone_set	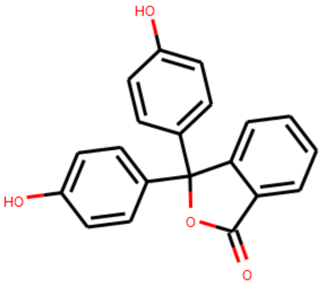	KJFMBFZCATUALV-UHFFFAOYSA-N	OC1=CC=C(C=C1)C1(OC(=O)C2=CC=CC=C12)C1=CC=C(O)C=C1	1.85
Acetone_set	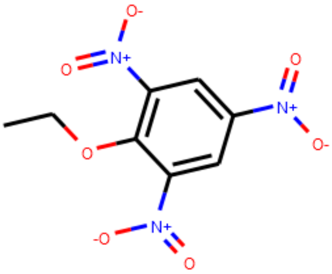	AOZXETLGPZAZNK-UHFFFAOYSA-N	CCOC1=C(C=C(C=C1N(=O)=O)N(=O)=O)N(=O)=O	1.67

Acetone_set	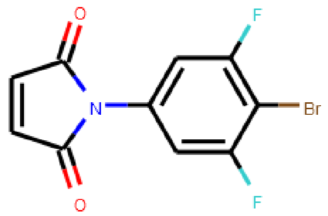	HEWGVSGLMQJYHJ-UHFFFAOYSA-N	FC1=CC(=CC(F)=C1Br)N1C(=O)C=CC1=O	1.44
Acetone_set	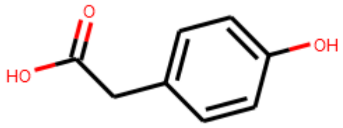	XQXPVVBIMDBYFF-UHFFFAOYSA-N	OC(=O)CC1=CC=C(O)C=C1	1.40
Acetone_set	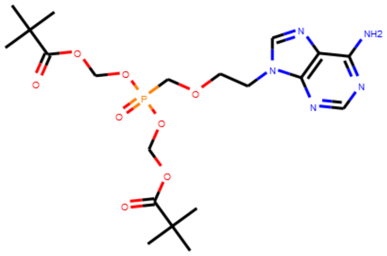	WOZSCQDILHKSGG-UHFFFAOYSA-N	CC(C)(C)C(=O)OCOP(=O)(COCCN1C=NC2=C1N=CN=C2N)OCOC(=O)C(C)(C)C	1.38
Acetone_set	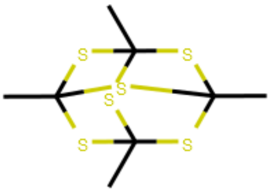	APZGKGHAXUURNO-UHFFFAOYSA-N	CC12SC3(C)SC(C)(S1)SC(C)(S2)S3	1.28
Acetone_set	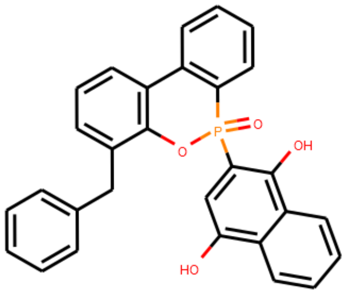	ZABCGYUGVOPKIL-UHFFFAOYSA-N	OC1=C2C=CC=CC2=C(O)C(=C1)P1(=O)OC2=C(C=CC=C2CC2=CC=CC=C2)C2=C1C=CC=C2	1.27
Acetone_set	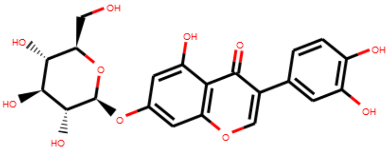	WJHSRFQBVYHKKL-CMWLGVBASA-N	OC[C@H]1O[C@@H](OC2=CC3=C(C(=O)C(=CO3)C3=CC(O)=C(O)C=C3)C(O)=C2)[C@H](O)[C@@H](O)[C@@H]1O	1.20

Acetone_set	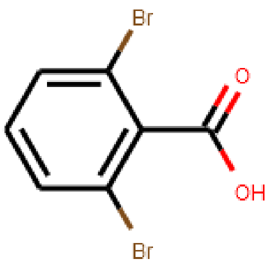	HQLOEBRPCVIFCT-UHFFFAOYSA-N	OC(=O)C1=C(Br)C=CC=C1Br	1.19
Acetone_set	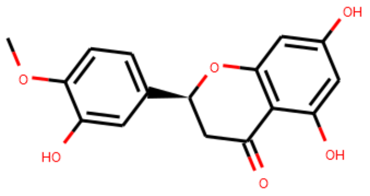	AIONOLUJZLIMTK-AWEZNQCLSA-N	COC1=CC=C(C(=C1O)[C@@H]1CC(=O)C2=C(O1)C=C(O)C=C2O	1.19
Acetone_set	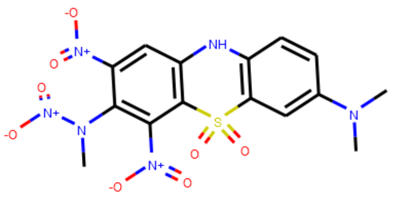	HCTVORVYPUEFSX-UHFFFAOYSA-N	CN(C)C1=CC2=C(NC3=C(C(=C(N(C)N(=O)=O)C(=C3)N(=O)=O)N(=O)=O)S2(=O)=O)C=C1	1.13
Acetone_set	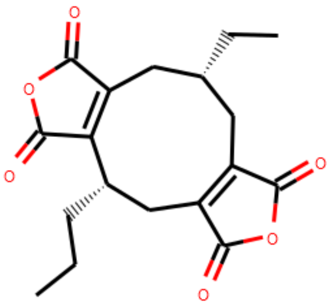	NRDWUPPIGBHWAS-ZJUUUORDSA-N	CCC[C@H]1CC2=C(C[C@@H](CC)CC3=C1C(=O)OC3=O)C(=O)OC2=O	1.13
Acetone_set	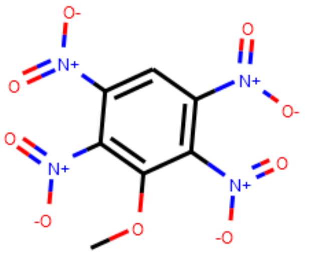	NHRIWEKOARVHSN-UHFFFAOYSA-N	COC1=C(C(=CC(=C1N(=O)=O)N(=O)=O)N(=O)=O)N(=O)=O	1.10
Acetone_set	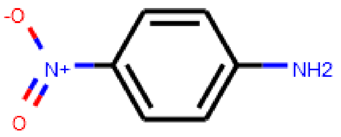	TYMLOMAKGOJONV-UHFFFAOYSA-N	NC1=CC=C(C=C1)N(=O)=O	1.09

Acetone_set	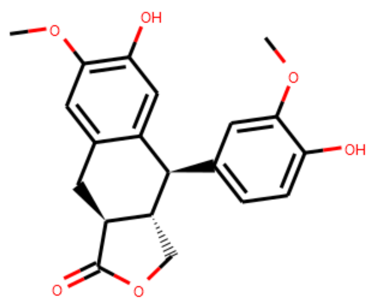	CAYMSCGTKZIVTN-IQUTYRLHSA-N	[H][C@@]12COC(=O)[C@@]1([H])CC1=CC(OC)=C(O)C=C1[C@H]2C1=CC=C(O)C(OC)=C1	1.05
Acetone_set	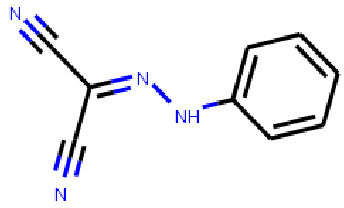	MENUYOGJCXAFFU-UHFFFAOYSA-N	N#C\C(=N/NC1=CC=CC=C1)C#N	1.03
Acetone_set	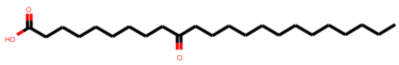	LIKSPRLIPHRBCL-UHFFFAOYSA-N	CCCCCCCCCCCCCC(=O)CCCCCCCCC(O)=O	1.02

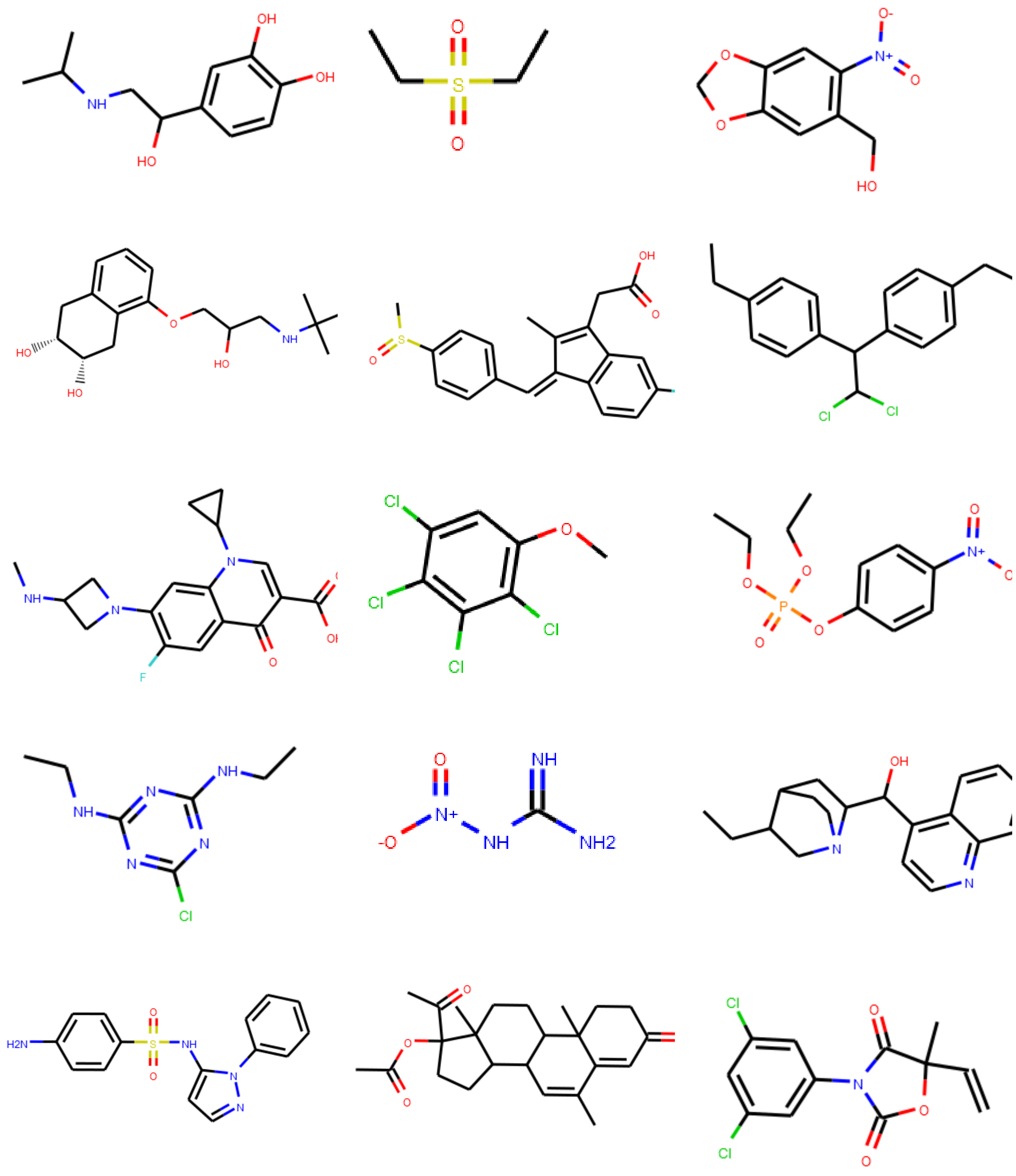

Figure 50. Outliers for ***Water_set_wide***

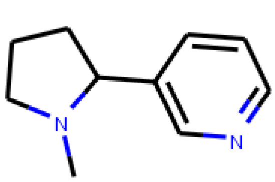
N
N
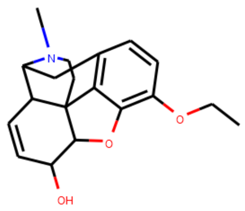
N
O
O
OH
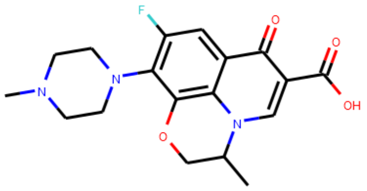
F
O
O
N
N
OH
O
N
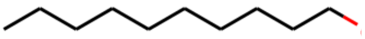
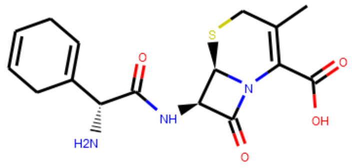
S
O
N
O
NH
H2N
OH
O
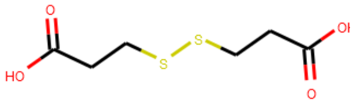
O
S
S
HO
O
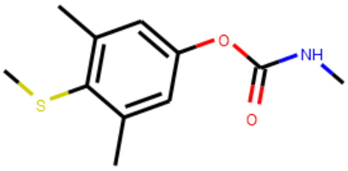
O
NH
S
O
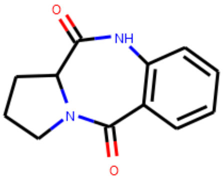
O
NH
N
O
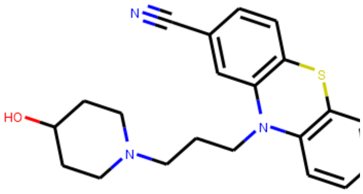
N
S
HO
N
N
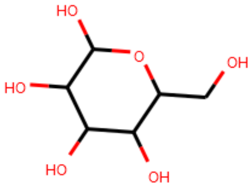
HO
O
OH
HO
HO
OH
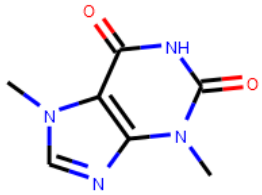
O
NH
N
O
N
N
N
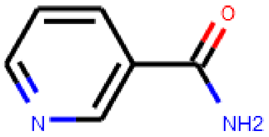
O
N
NH2
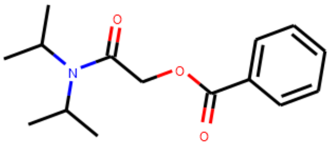
O
N
O
O
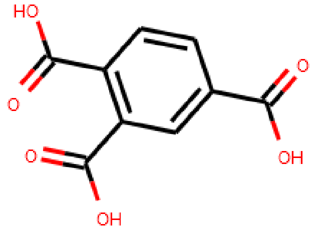
HO
O
O
O
OH
OH
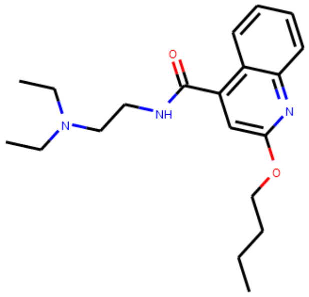
O
N
NH
N
O
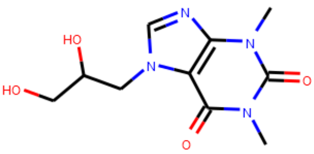
N
HO
N
O
HO
N
O
N
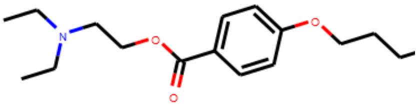
N
O
O
O
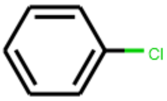
Cl
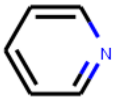
N
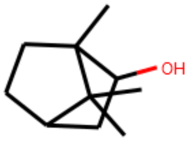
OH
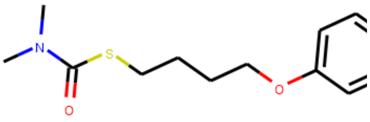
N
S
O
O

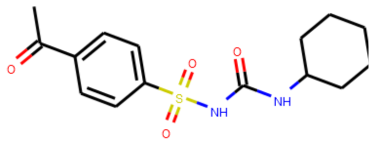

Figure 51. Outliers for ***Water_set_narrow***

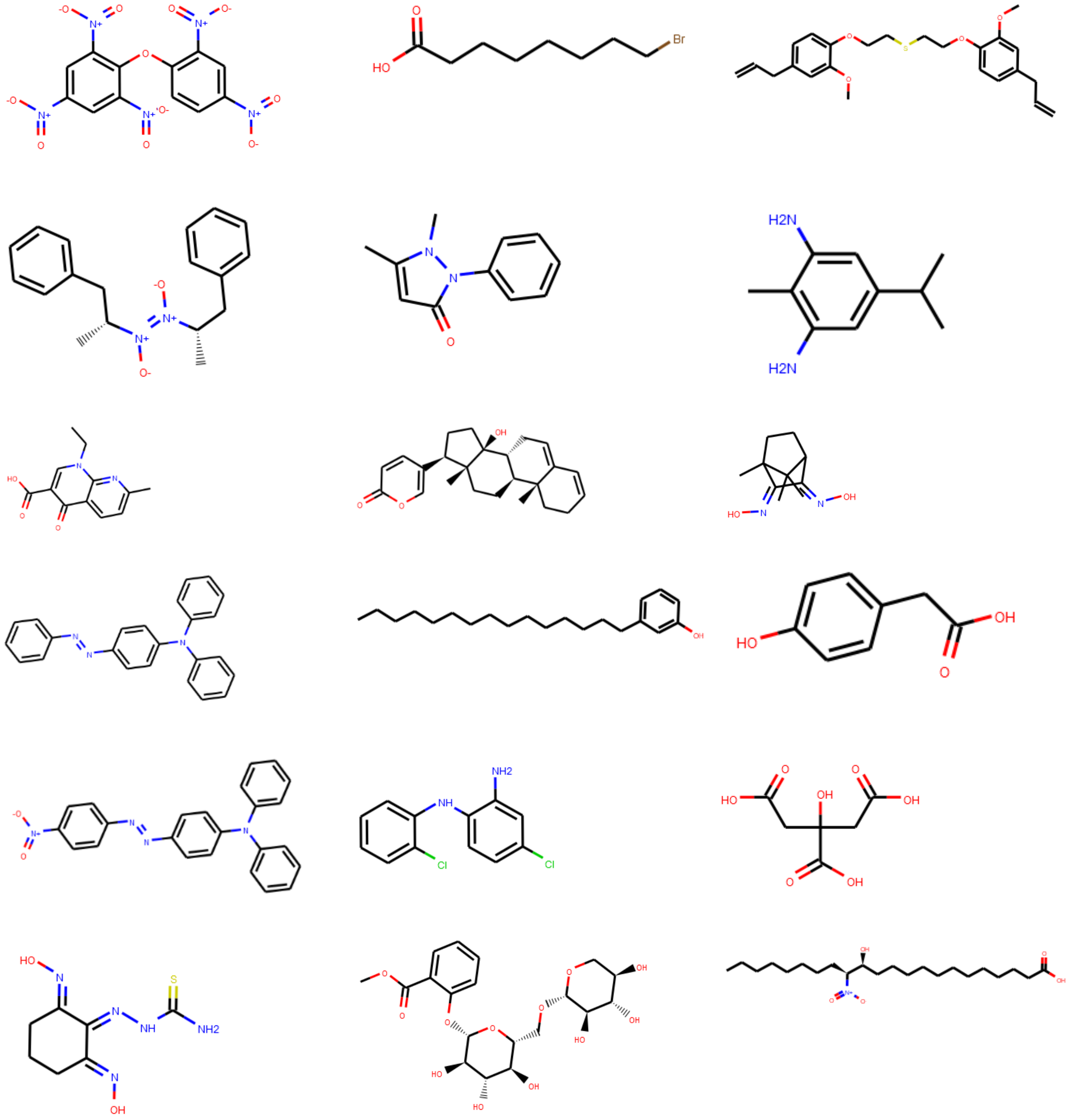

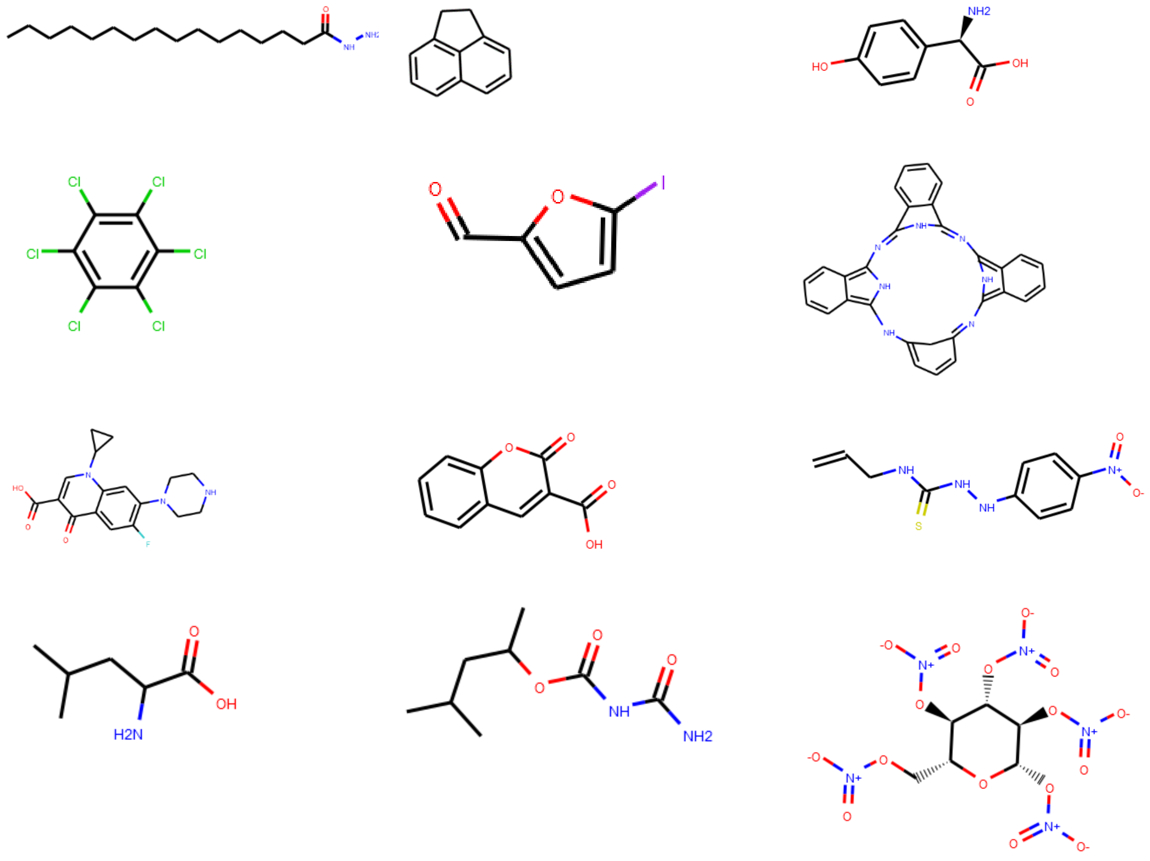

Figure 52. Outliers for ***Ethanol_set***

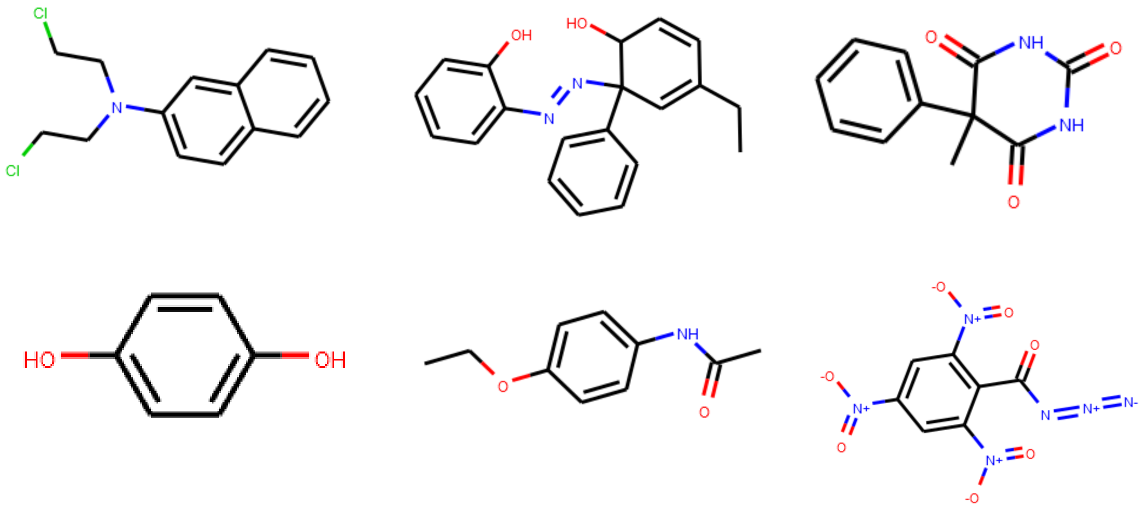

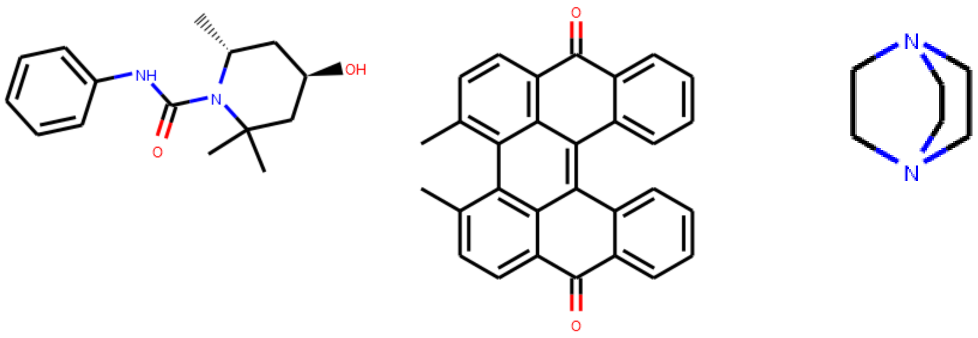

Figure 53. Outliers for ***Benzene_set***

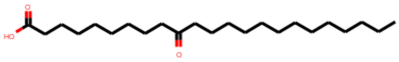
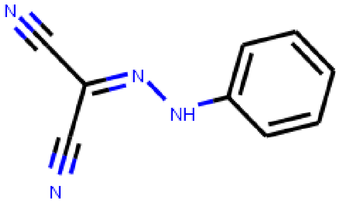
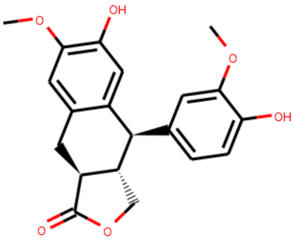
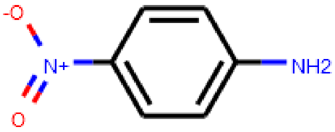
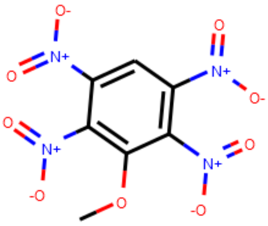
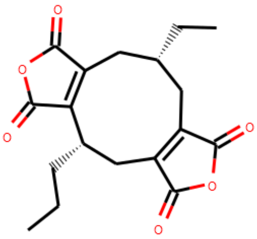
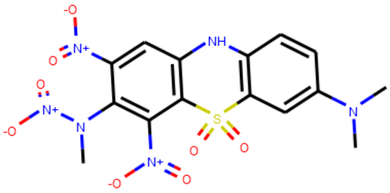
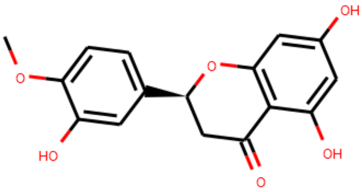
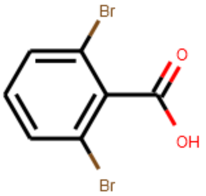
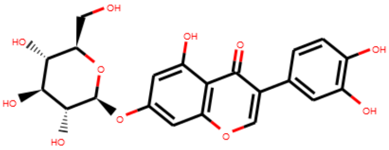
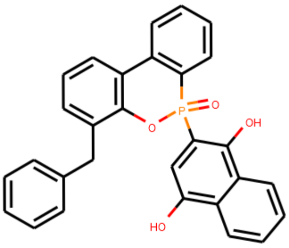
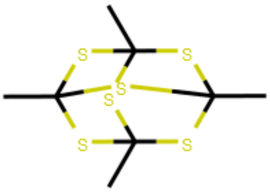
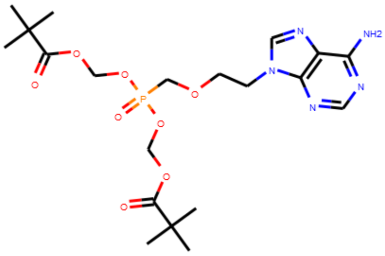
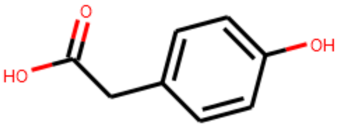
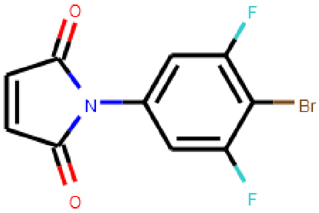
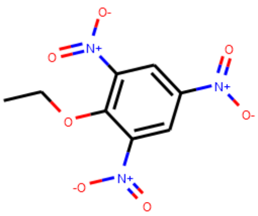
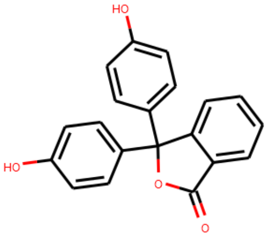
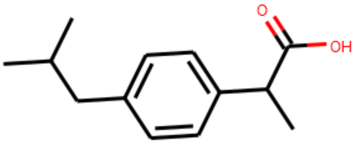

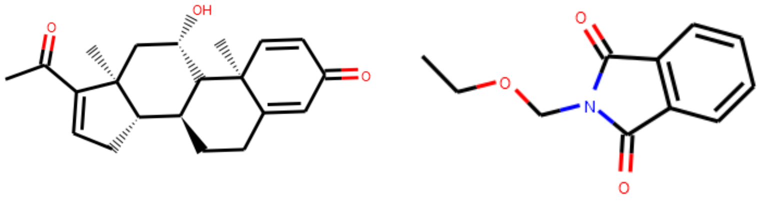

Figure 54. Outliers for ***Acetone_set***

4.9.1 Functional group analysis

For the best models using ET algorithm and 14 DFT descriptors, all molecules with predicted LogS outside the ±1 range of experimental values were considered outliers. These poorly predicted molecules were analysed to identify limitations of the models to inform further improvements. The common functional groups in the outliers are summarised in **Figure 55**.

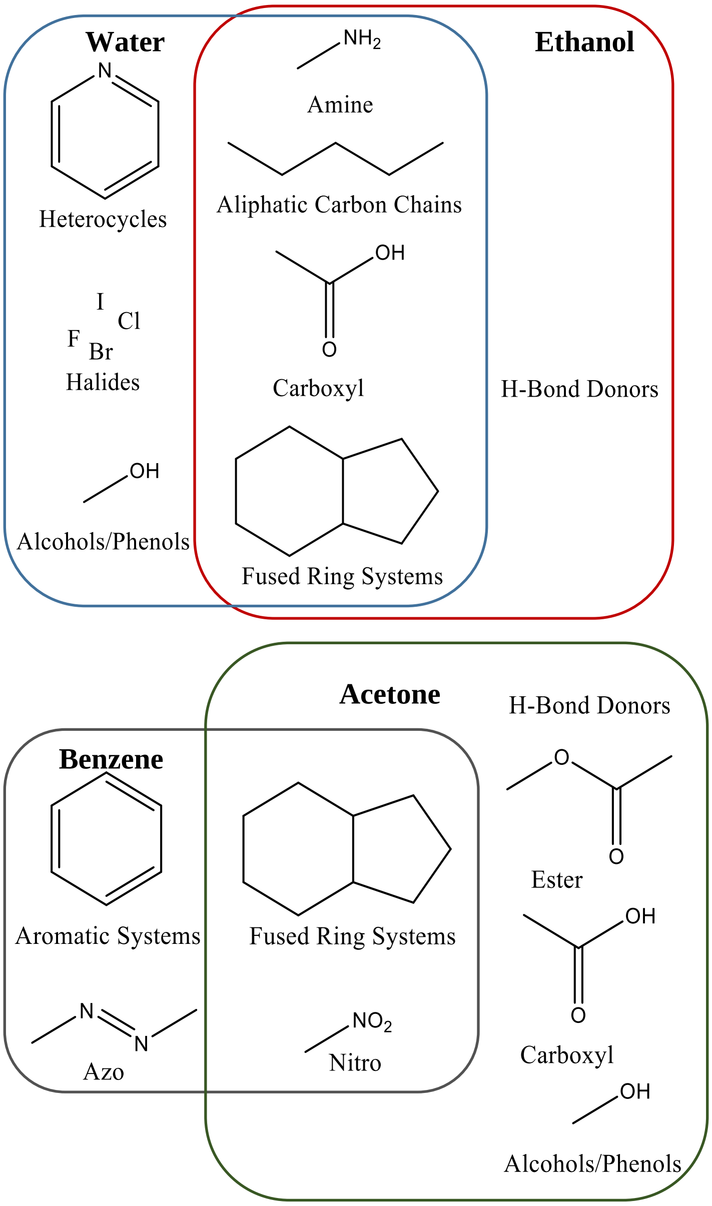

Figure 55. Common functional groups for outliers in different datasets

4.9.2 Complexity analysis

Correlation was also observed between molecular complexity and prediction accuracy. The BertzCT topological index was calculated for each outlier and inlier based on two terms: complexity of bonding and distribution of heteroatoms.[15] The outcome is summarised in **Figure 56**, where an inlier is defined as any molecule which is not an outlier.

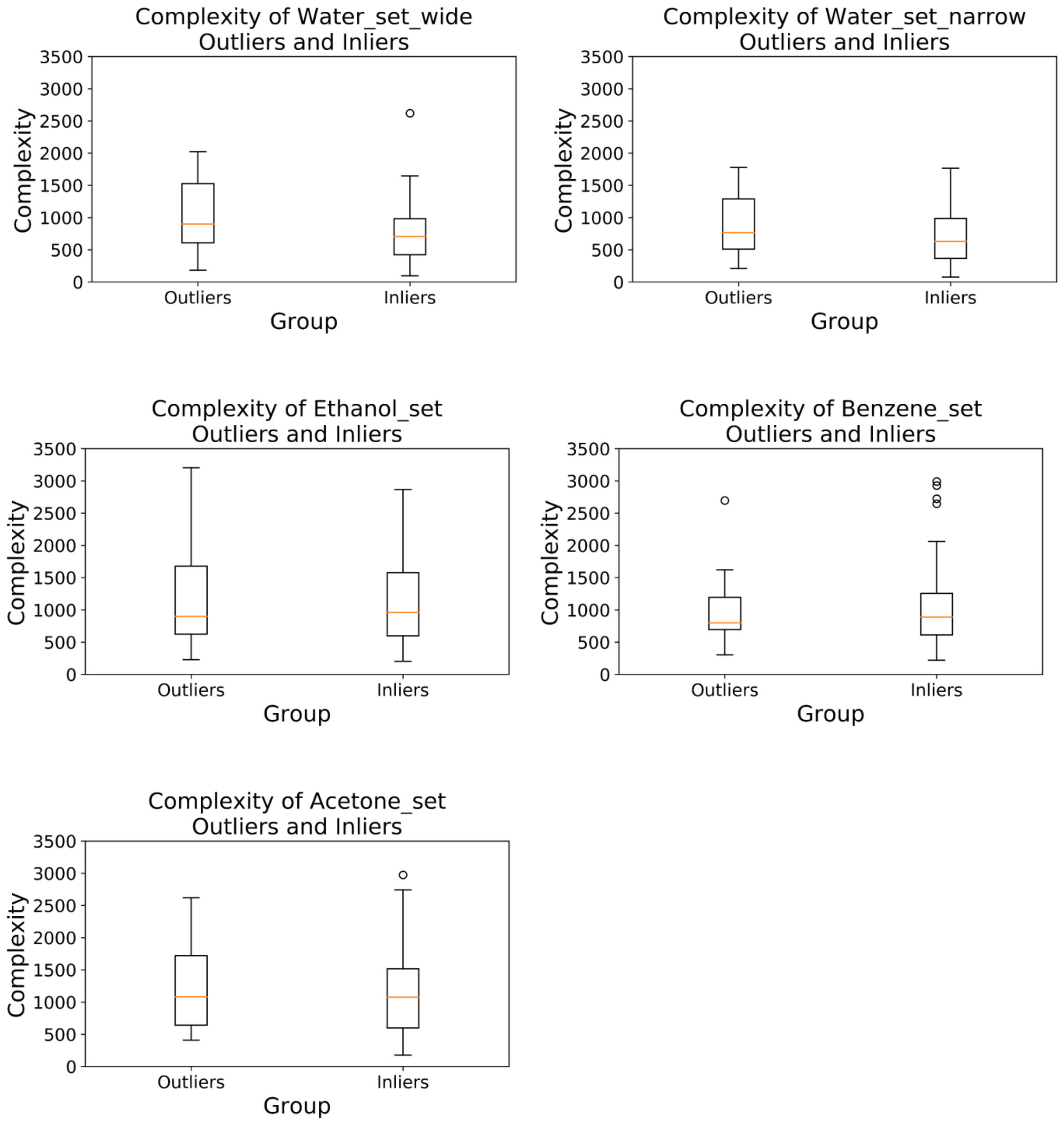

Figure 56. Complexity of outliers and inliers for ***Water_set_wide*** dataset

4.9.3 Solubility range analysis

Outliers were analysed to show if which solubility regions were poorly predicted. This is summarised in **Figure 57** and **Figure 58**.

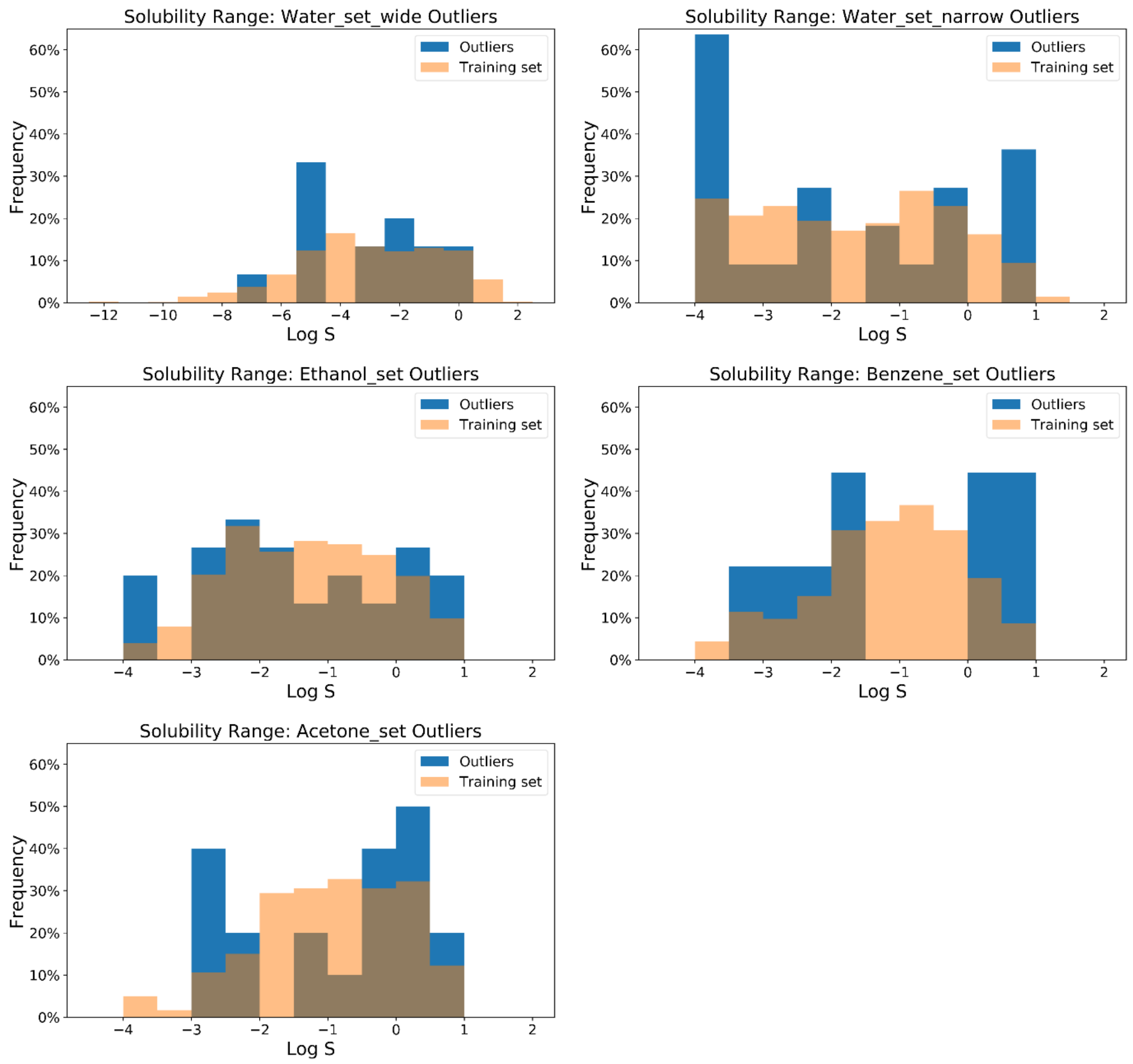

Figure 57. Distribution of LogS in training sets and outliers.

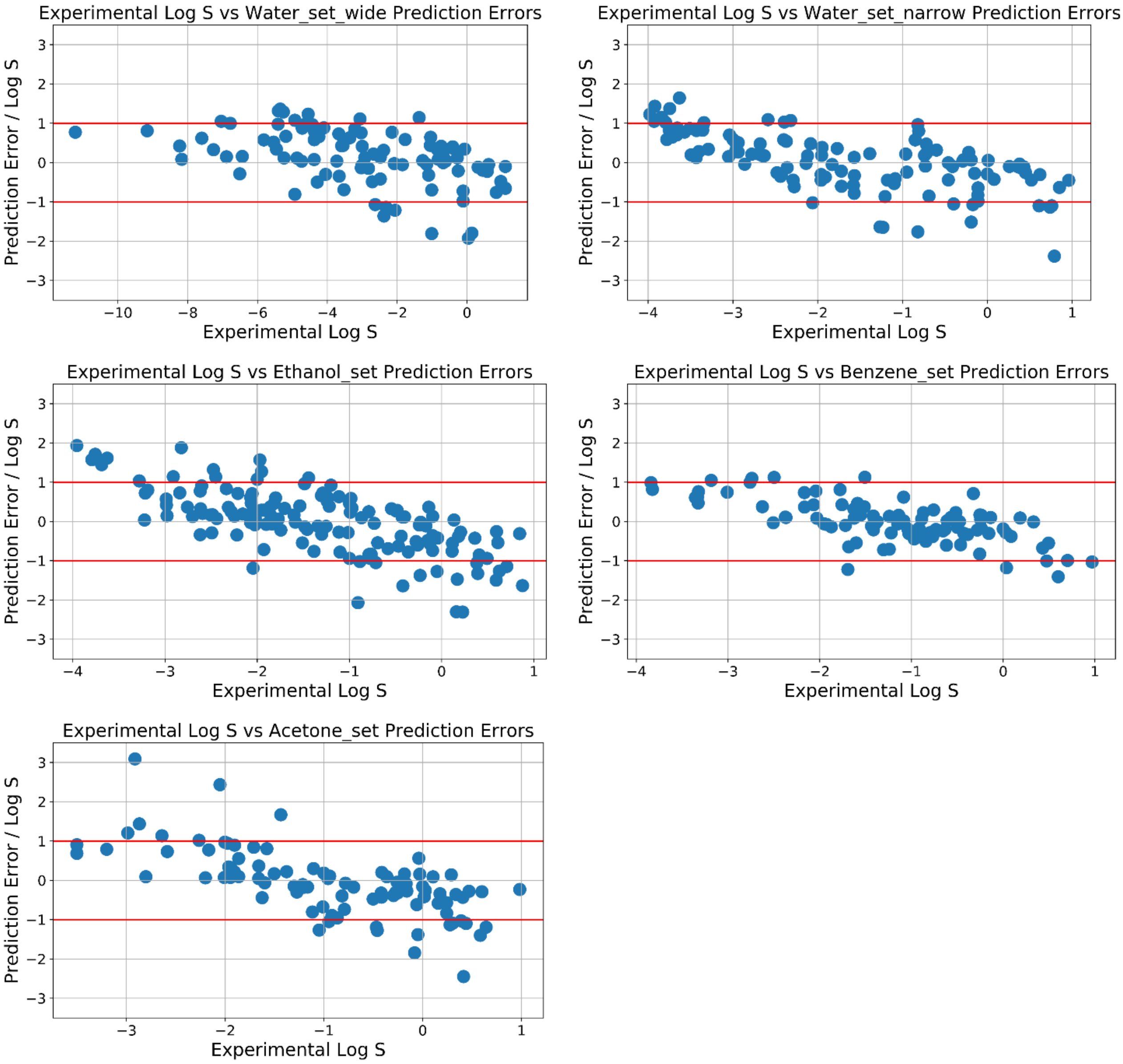

Figure 58. Prediction error *vs* LogS for different datasets

4.10 Improvement of descriptors

4.10.1 Feature importance

The feature important analysis was performed on ET models. Each descriptor was removed in turn and the model rebuilt. How the metrics changed in shown in **Figure 59** to **Figure 61**. In the full model, the average weighting and standard deviation of each descriptor in ET algorithm was plotted (100 runs). This is shown in **Figure 62** and explained in section 7.11.

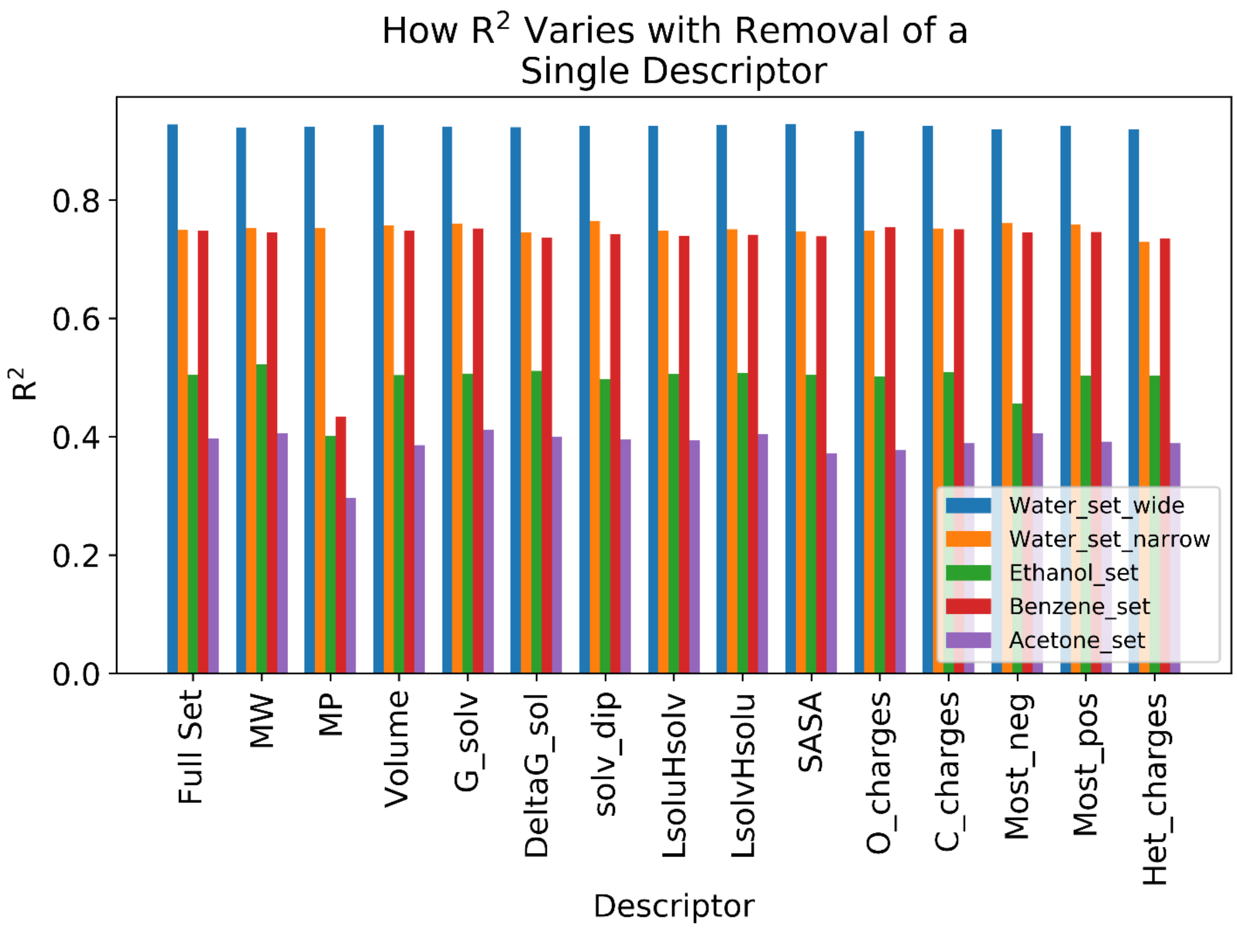

Figure 59. Effect of leaving each descriptor out on the predictive model for each dataset on R^2

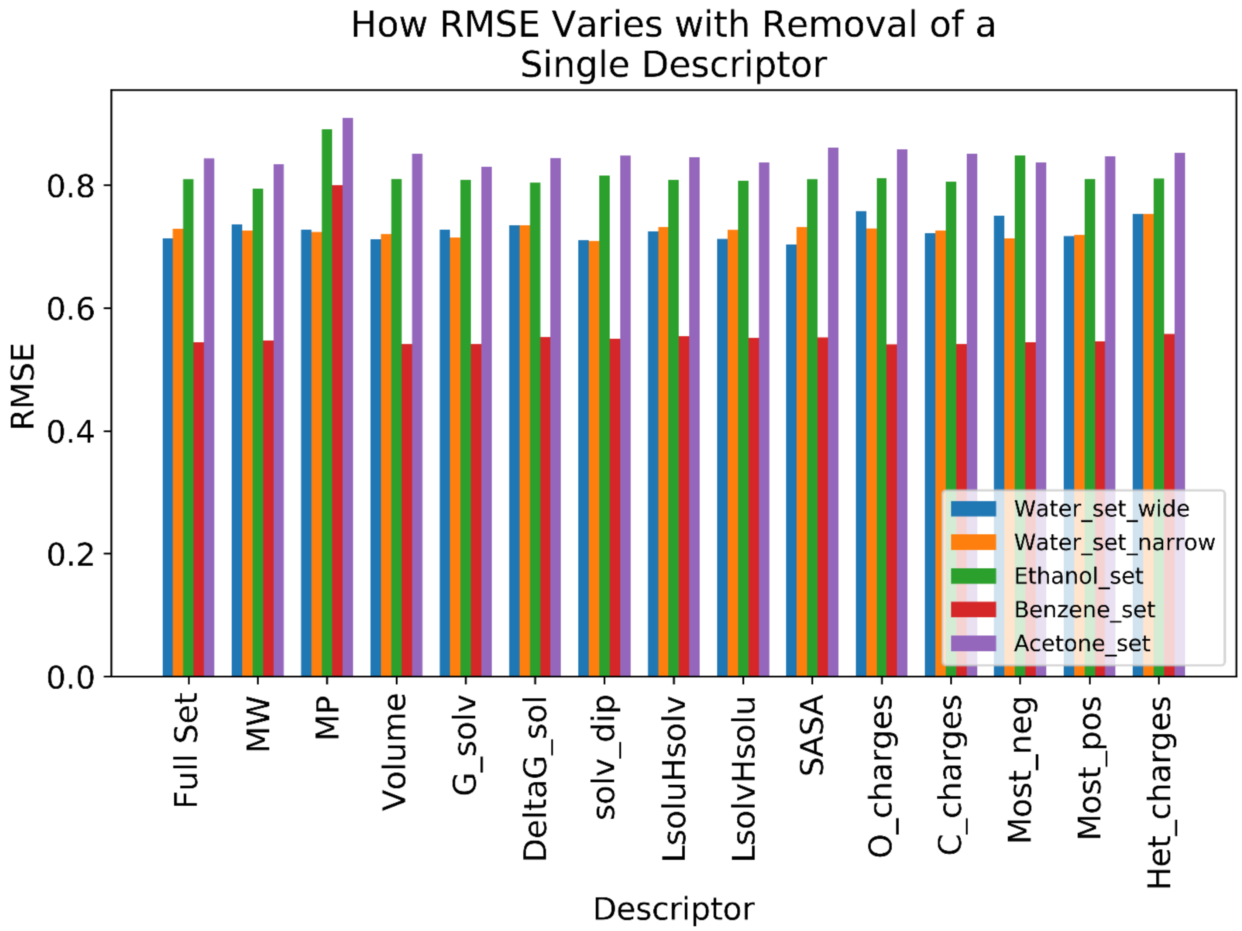

Figure 60. Effect of leaving each descriptor out on the predictive model for each dataset on RMSE

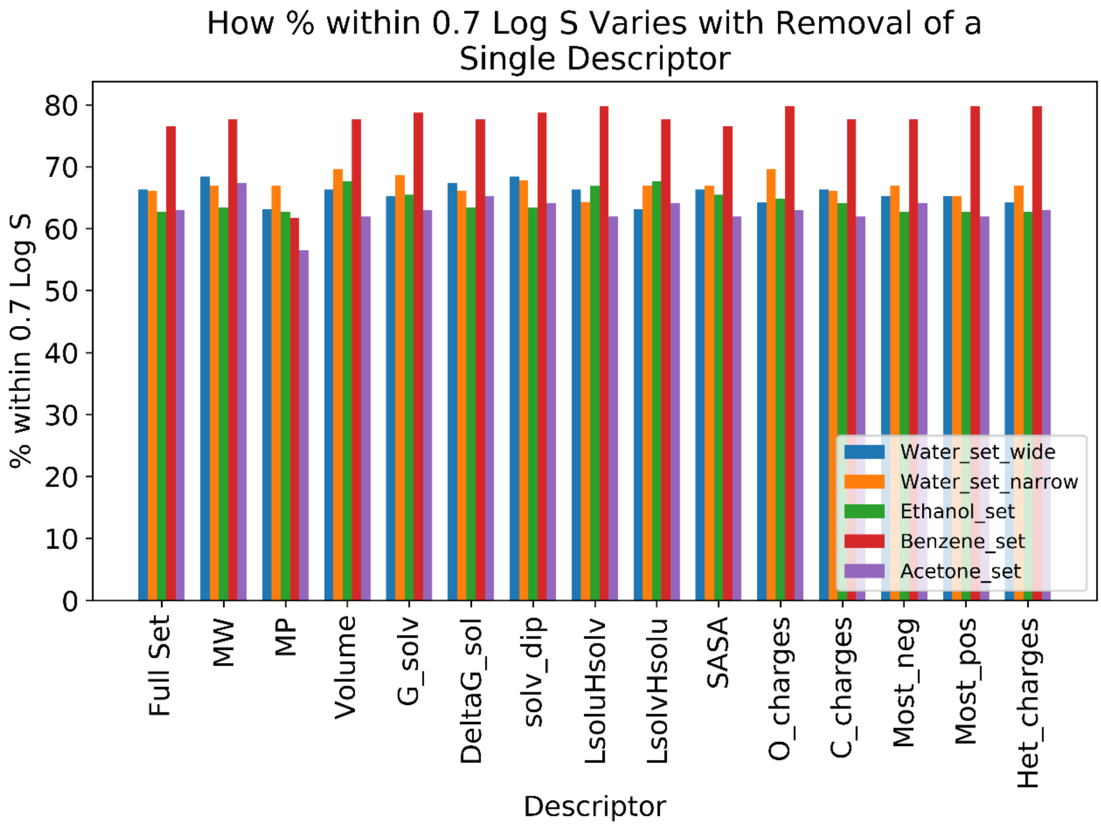
How % within 0.7 Log S Varies with Removal of a Single Descriptor
% within 0.7 Log S
0
10
20
30
40
50
60
70
80
Water_set_wide
Water_set_narrow
Ethanol_set
Benzene_set
Acetone_set
Full Set
MW
MP
Volume
G_solv
DeltaG_sol
solv_dip
LsoluHsolv
LsolvHsolu
SASA
O_charges
C_charges
Most_neg
Most_pos
Het_charges
Descriptor

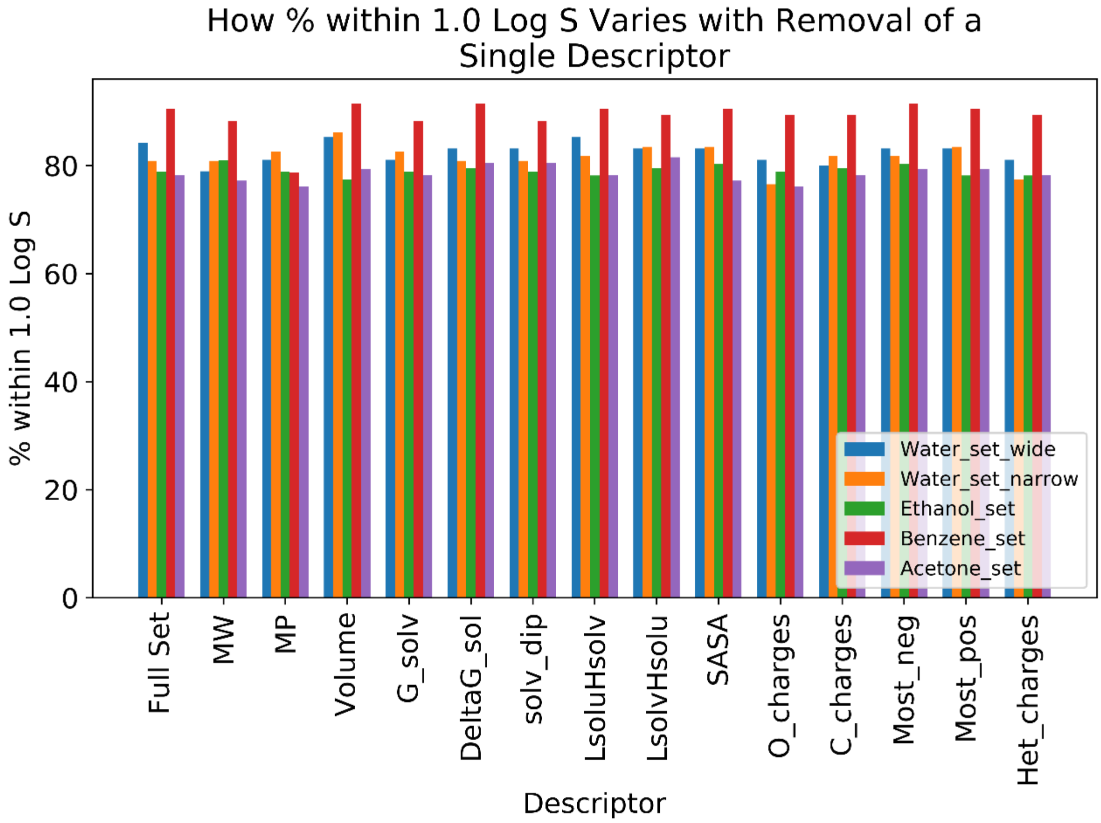
How % within 1.0 Log S Varies with Removal of a Single Descriptor
% within 1.0 Log S
0
20
40
60
80
Water_set_wide
Water_set_narrow
Ethanol_set
Benzene_set
Acetone_set
Full Set
MW
MP
Volume
G_solv
DeltaG_sol
solv_dip
LsoluHsolv
LsolvHsolu
SASA
O_charges
C_charges
Most_neg
Most_pos
Het_charges
Descriptor

Figure 61. Effect of leaving each descriptor out on the predictive model for each dataset on %LogS±0.7 and %LogS±1.0

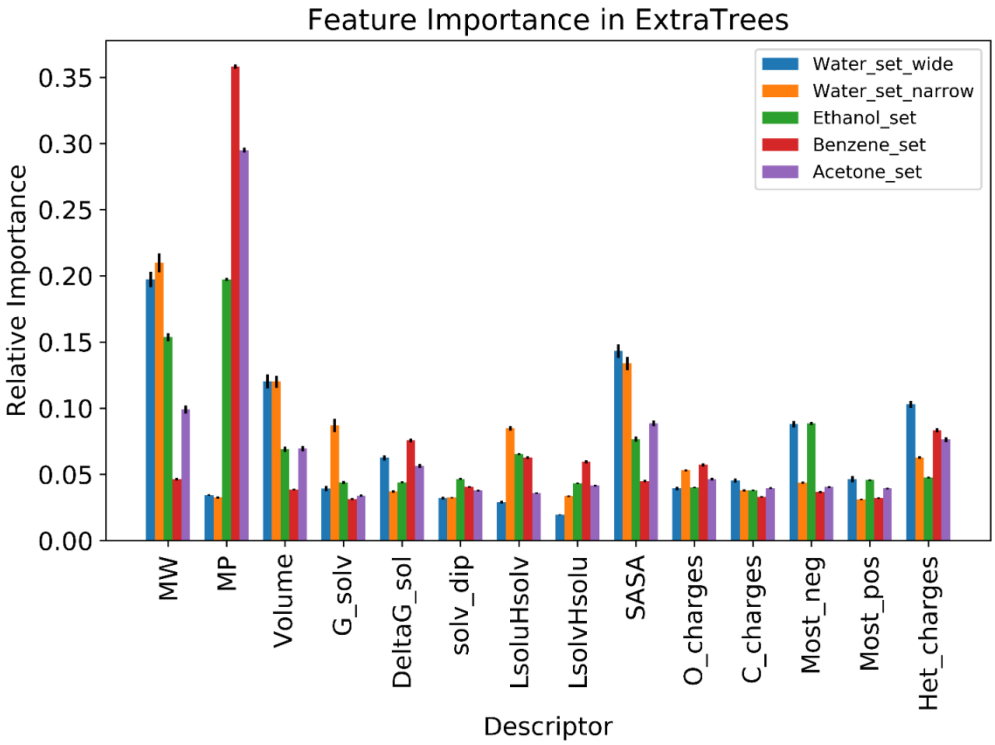

Figure 62. Feature importance plots for ET models of five datasets

4.10.2 Inclusion of shape/size/charge descriptors

A set of 13 additional shape/size/charge descriptors, were included with the original 14 descriptors. Using these new set of 27 descriptors, new models were generated as before for all 5 datasets. The new descriptor set are summarised in **Table 15**.

Table 15. New set of 27 descriptor; 14 original and 13 additional

No.	Name	Source	Description
1	G_solv	G09	Gibbs free energy of optimised solution structure (Hartrees)
2	DeltaG_sol	G09	Solvation energy calculated as G_solv - G_gas (Hartrees)
3	$L_{solu}H_{solv}$	G09	The gap in energy between the LUMO of the solute and the HOMO of the solvent (eV)
4	$L_{solv}H_{solu}$	G09	The gap in energy between LUMO of the solvent and HOMO of the solute (eV)
5	Solv_dip	G09	Dipole of solution structure (Debye)

6	O_charges	G09	Sum of charges on solution structure oxygen atoms
7	C_charges	G09	Sum of charges on solution structure carbon atoms
8	Most_neg	G09	Charge on most negative atom of solution structure
9	Most_pos	G09	Charge on most positive atom of solution structure
10	Het_charges	G09	Sum of charges on solution structure non-hydrogen/carbon atoms
11	Volume	G09	Molar volume (cm^{-3}.mol)
12	SASA	G09/Pymol	Solvent Accessible Surface Area ($Å^2$)
13	MW	Python	Molecular weight (Daltons)
14	m.p.	Reaxys/ ChemSpider	Experimental melting point (°C)
15	No_regions	G09/multiwfn[13]	Number of charged regions
16	Tot_charge	G09/multiwfn	Sum of all charges
17	Neg_charge	G09/multiwfn	Sum of negative charges
18	Pos_charge	G09/multiwfn	Sum of positive charges
19	Big_area	G09/multiwfn	Area of biggest region of charge (pixels)
20	Big_charge	G09/multiwfn	Mean charge of biggest region of charge (pixels)
21	Big_std	G09/multiwfn	Standard deviation (spread) of biggest region of charge (pixels)
22	Area1	G09/OpenCV	Area of smallest shadow projection (pixels)
23	Area2	G09/OpenCV	Area of second smallest shadow projection (perpendicular to first) (pixels)
24	Area3	G09/OpenCV	Area of largest shadow projection (perpendicular to first and second) (pixels)
25	Asp1	G09/OpenCV	Aspect ratio of smallest shadow projection
26	Asp2	G09/OpenCV	Aspect ratio of second smallest shadow projection (perpendicular to first)
27	Asp3	G09/OpenCV	Aspect ratio of largest shadow projection (perpendicular to first and second)

The correlation between the set of 27 descriptors is shown in **Figure 63**. The only new correlations observed with $R^2 > 0.90$ was between area descriptors and SASA (Area2, SASA = 0.91 and Area3, SASA = 0.91 for ***Water_set_wide***). A comparison of models built with 14 and 27 descriptors is shown in **Table 16**, **Table 17** and **Figure 64**.

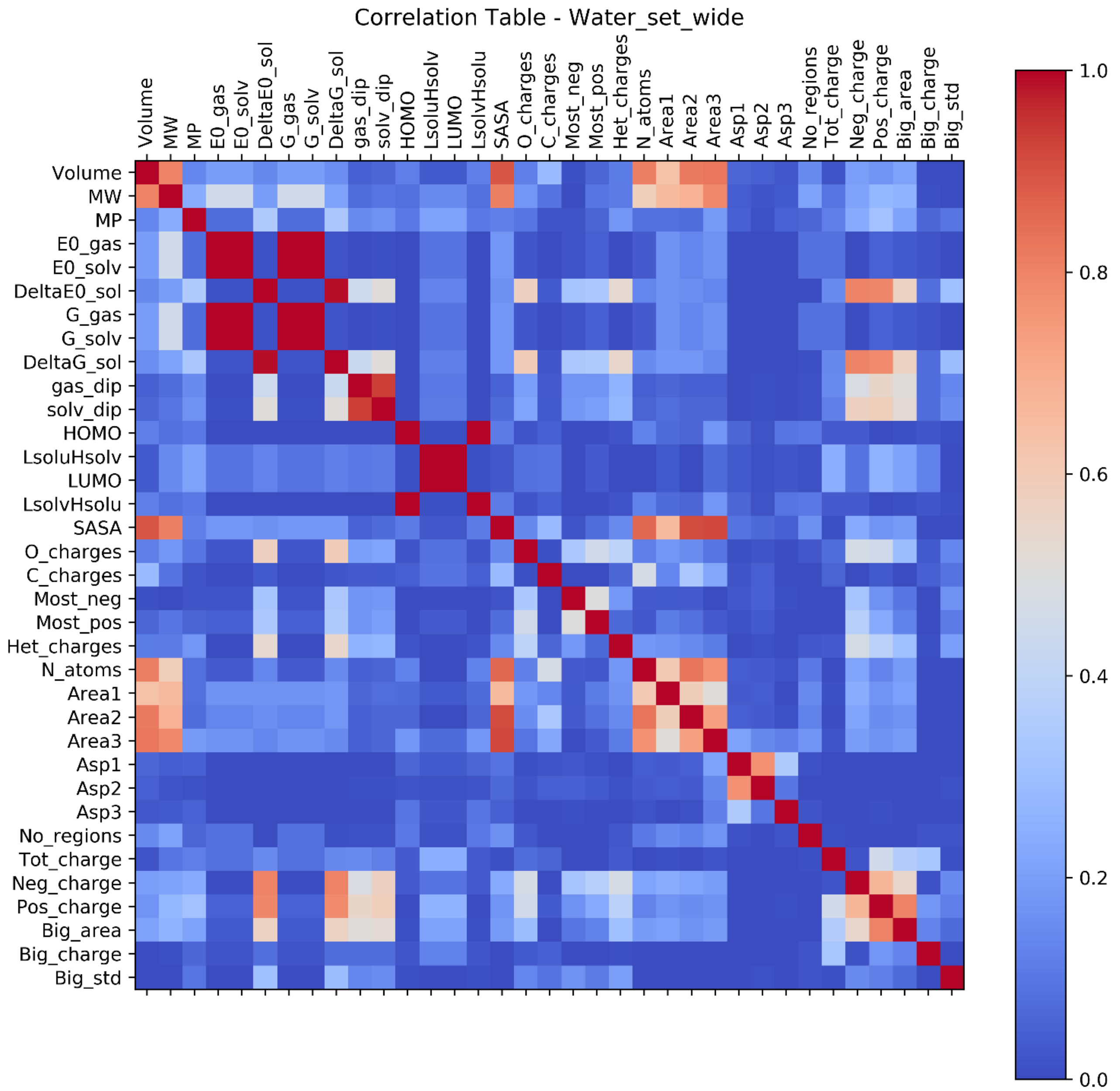

Figure 63. Correlation table for ***Water_set_wide*** for the full set of all descriptors considered in this work

Table 16. Comparison of 10-fold cross validation results with 14 and 27 descriptors

Method	R^2	RMSE	%LogS±0.7	%LogS±1.0	R^2	RMSE	%LogS±0.7	%LogS±1.0

Water_set_wide								
	**14 Descriptors**				**27 Descriptors**			
MLR	0.77	1.16	52.1	67.0	0.77	1.15	52.3	67.7
ANN	0.87	0.89	64.9	79.9	0.87	0.87	67.1	79.8
SVM	0.88	0.85	68.3	81.7	0.87	0.85	69.3	80.6
PLS	0.77	1.16	52.2	66.9	0.77	1.15	52.7	67.1
RF	0.84	0.95	62.3	75.7	0.84	0.95	62.3	76.7
ET	0.87	0.88	66.3	79.4	0.86	0.90	66.1	78.2
Bag	0.84	0.95	62.1	76.4	0.84	0.95	62.6	76.9
GP	0.88	0.86	67.0	79.2	0.88	0.85	67.7	81.2
Water_set_narrow								
MLR	0.60	0.88	61.2	76.3	0.60	0.89	59.8	76.8
ANN	0.66	0.83	67.3	79.8	0.66	0.82	66.6	80.2
SVM	0.70	0.76	68.6	82.9	0.71	0.76	69.1	84.1
PLS	0.60	0.88	61.4	76.1	0.60	0.89	60.7	76.1
RF	0.67	0.80	64.7	80.5	0.66	0.81	64.8	80.0
ET	0.69	0.77	66.4	82.3	0.69	0.77	66.8	81.4
Bag	0.67	0.80	64.8	81.1	0.66	0.81	65.2	80.2
GP	0.70	0.77	68.0	82.7	0.69	0.77	68.4	83.2
Ethanol_set								
MLR	0.39	0.87	59.9	77.1	0.40	0.87	59.7	76.1
ANN	0.51	0.80	67.8	81.6	0.52	0.80	68.5	82.0
SVM	0.54	0.76	69.8	83.6	0.54	0.76	69.3	83.3

PLS	0.39	0.87	59.7	76.7	0.40	0.87	59.7	75.7
RF	0.54	0.76	68.5	82.2	0.55	0.76	68.0	83.3
ET	0.54	0.76	69.1	81.7	0.54	0.76	69.8	82.3
Bag	0.55	0.76	69.1	82.7	0.54	0.76	67.8	83.3
GP	0.53	0.77	68.8	82.0	0.51	0.78	67.6	80.9
Benzene_set								
MLR	0.47	0.79	68.8	81.3	0.46	0.80	67.2	80.4
ANN	0.58	0.72	71.4	84.5	0.61	0.70	72.4	85.8
SVM	0.57	0.72	70.7	84.5	0.58	0.72	72.0	83.4
PLS	0.47	0.80	67.5	81.3	0.45	0.80	66.4	80.4
RF	0.62	0.67	72.2	87.3	0.62	0.68	72.5	86.9
ET	0.64	0.66	72.7	88.4	0.65	0.66	72.2	88.4
Bag	0.62	0.67	71.4	87.5	0.62	0.68	72.9	86.6
GP	0.57	0.72	70.1	83.8	0.56	0.73	71.5	83.8
Acetone_set								
MLR	0.47	0.77	69.2	81.4	0.47	0.78	70.4	81.9
ANN	0.54	0.73	71.7	83.4	0.53	0.73	72.3	84.5
SVM	0.57	0.70	76.3	84.3	0.56	0.71	75.0	83.6
PLS	0.47	0.77	68.8	81.6	0.47	0.78	69.3	81.6
RF	0.57	0.69	74.8	85.4	0.57	0.71	73.9	85.0
ET	0.56	0.70	73.7	84.5	0.56	0.71	73.9	82.7
Bag	0.57	0.70	73.7	84.9	0.56	0.72	72.6	84.7
GP	0.52	0.74	72.6	83.4	0.56	0.73	71.5	83.8

Table 17. Comparison of results of explicit train test split; 14 against 27 descriptors

Method	R^2	RMSE	%LogS±0.7	%LogS±1.0	R^2	RMSE	%LogS±0.7	%LogS±1.0
Water_set_wide								
	**14 Descriptors**				**27 Descriptors**			
MLR	0.80	1.16	50.5	65.3	0.80	1.16	49.5	63.2
ANN	0.90	0.84	58.9	78.9	0.88	0.91	63.2	76.8
SVM	0.89	0.85	71.6	78.9	0.88	0.87	65.3	74.7
PLS	0.80	1.16	51.6	66.3	0.80	1.16	48.4	58.9
RF	0.90	0.83	60.0	75.8	0.89	0.85	60.0	77.9
ET	0.93	0.71	66.3	84.2	0.92	0.77	63.2	76.8
Bag	0.90	0.82	57.9	76.8	0.90	0.84	63.2	75.8
GP	0.88	0.89	68.4	73.7	0.90	0.83	62.1	78.9
Water_set_narrow								
MLR	0.68	0.82	61.7	80.0	0.67	0.83	61.7	76.5
ANN	0.74	0.74	68.7	84.3	0.68	0.81	64.3	81.7
SVM	0.76	0.71	65.2	81.7	0.71	0.78	67.0	80.9
PLS	0.68	0.83	61.7	80.0	0.66	0.84	63.5	76.5
RF	0.72	0.76	60.9	82.6	0.69	0.81	55.7	79.1
ET	0.75	0.73	66.1	80.9	0.73	0.75	66.1	79.1
Bag	0.72	0.77	60.9	81.7	0.69	0.81	57.4	78.3
GP	0.76	0.71	73.0	81.7	0.72	0.77	67.0	80.9
Ethanol_set								

MLR	0.29	0.98	50.7	72.5	0.28	1.00	53.5	72.5
ANN	0.49	0.88	64.1	76.8	0.45	0.91	63.4	76.8
SVM	0.51	0.81	64.1	78.9	0.50	0.83	64.8	76.8
PLS	0.29	0.99	51.4	71.8	0.30	0.98	54.9	73.9
RF	0.53	0.79	64.8	82.4	0.49	0.82	60.6	81.0
ET	0.50	0.81	62.7	78.9	0.49	0.82	59.2	79.6
Bag	0.52	0.80	65.5	79.6	0.49	0.82	61.3	81.7
GP	0.51	0.80	66.2	77.5	0.47	0.84	61.3	75.4
Benzene_set								
MLR	0.64	0.66	75.5	86.2	0.63	0.65	74.5	84.0
ANN	0.67	0.63	77.7	88.3	0.70	0.59	75.5	90.4
SVM	0.71	0.58	76.6	89.4	0.67	0.61	76.6	89.4
PLS	0.64	0.66	74.5	85.1	0.64	0.64	75.5	81.9
RF	0.72	0.57	76.6	90.4	0.73	0.57	78.7	90.4
ET	0.75	0.54	76.6	90.4	0.74	0.56	80.9	87.2
Bag	0.72	0.57	75.5	89.4	0.72	0.57	79.8	89.4
GP	0.70	0.58	79.8	90.4	0.72	0.57	81.9	91.5
Acetone_set								
MLR	0.36	0.87	60.9	78.3	0.41	0.84	63.0	81.5
ANN	0.42	0.87	67.4	79.3	0.48	0.80	73.9	80.4
SVM	0.42	0.83	72.8	81.5	0.37	0.86	64.1	80.4
PLS	0.35	0.87	62.0	78.3	0.40	0.84	66.3	82.6
RF	0.40	0.84	62.0	80.4	0.45	0.80	65.2	79.3

ET	0.40	0.84	63.0	78.3	0.46	0.80	68.5	83.7
Bag	0.41	0.83	62.0	80.4	0.46	0.80	66.3	80.4
GP	0.42	0.83	68.5	84.8	0.47	0.79	67.4	83.7

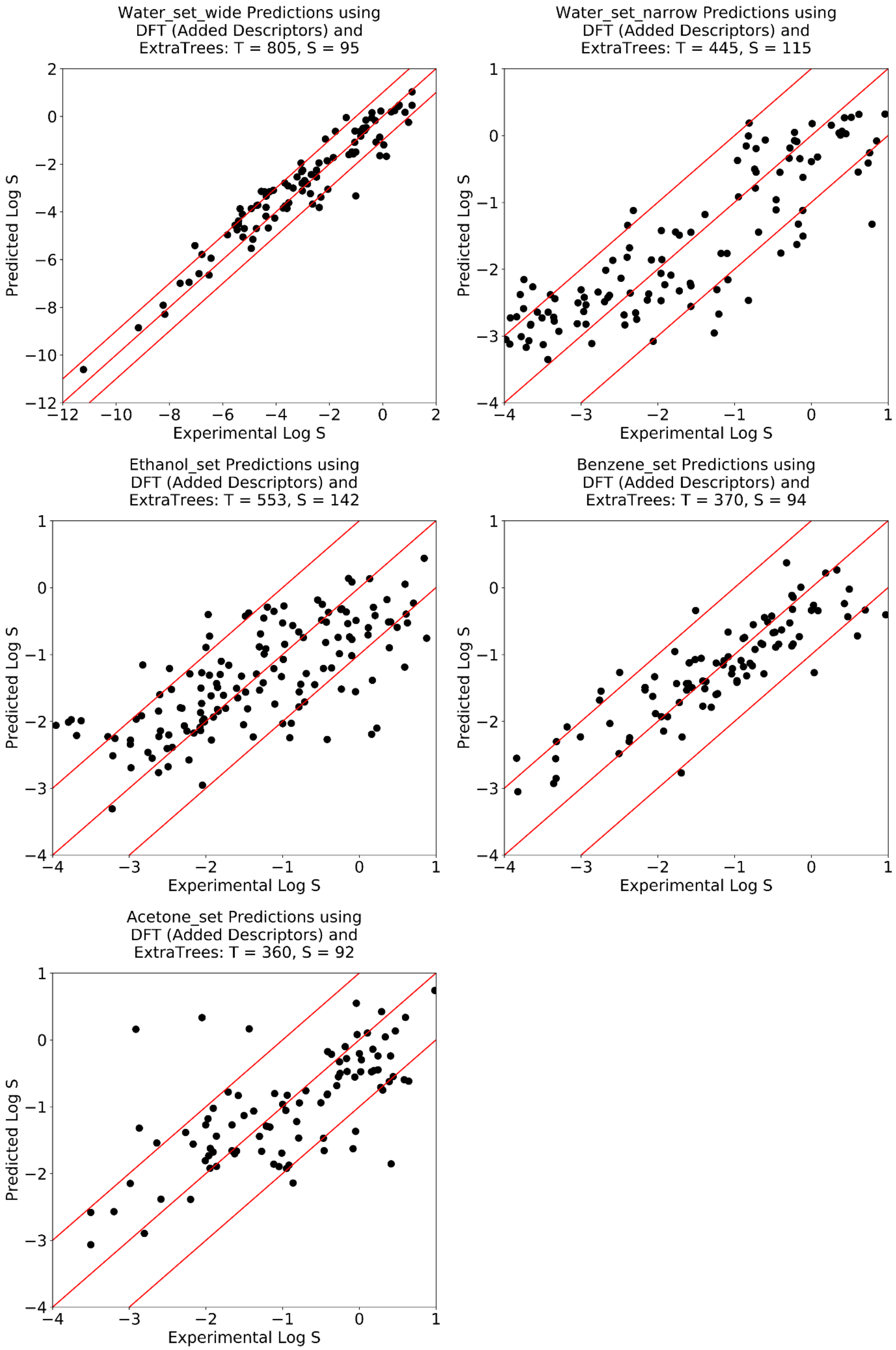
Water_set_wide Predictions using
DFT (Added Descriptors) and
ExtraTrees: T = 805, S = 95
Predicted Log S
Experimental Log S
Water_set_narrow Predictions using
DFT (Added Descriptors) and
ExtraTrees: T = 445, S = 115
Predicted Log S
Experimental Log S
Ethanol_set Predictions using
DFT (Added Descriptors) and
ExtraTrees: T = 553, S = 142
Predicted Log S
Experimental Log S
Benzene_set Predictions using
DFT (Added Descriptors) and
ExtraTrees: T = 370, S = 94
Predicted Log S
Experimental Log S
Acetone_set Predictions using
DFT (Added Descriptors) and
ExtraTrees: T = 360, S = 92
Predicted Log S
Experimental Log S

Figure 64. Explicit train test split plots for ET method and 27 descriptors

4.10.3 Improved solvation energy descriptor

Energy descriptors were recalculated using the literature method suggested by Kromann *et al.* (2018): HF SMD and PM6 SMD solvation models.[16] There descriptors were G_solv and DeltaG_sol. All other descriptors remained unchanged. These were used instead of those calculated using B3LYP and PM6 PCM methods in the original 14 descriptors. The original DFT/PM6 geometry of the molecule was fixed and the HF SMD protocol only used to recalculate the energy (single-point calculation). A summary of these results for DFT can be found in **Table 18**, **Table 19** and **Figure 65**.

Table 18. Comparison of 10-fold cross validation results for original DFT PCM compared to energy descriptors replaced with those obtained with HF SMD method

Method	R^2	RMSE	%LogS±0.7	%LogS±1.0	R^2	RMSE	%LogS±0.7	%LogS±1.0
Water_set_wide								
	**Original DFT PCM Procedure**				**Energy Descriptors Replaced by HF SMD**			
MLR	0.77	1.16	52.1	67.0	0.76	1.18	51.6	67.3
ANN	0.87	0.89	64.9	79.9	0.87	0.87	65.4	80.7
SVM	0.88	0.85	68.3	81.7	0.88	0.82	69.9	81.1
PLS	0.77	1.16	52.2	66.9	0.76	1.18	51.7	67.2
RF	0.84	0.95	62.3	75.7	0.85	0.94	63.4	78.0
ET	0.87	0.88	66.3	79.4	0.87	0.86	65.9	80.1
Bag	0.84	0.95	62.1	76.4	0.85	0.95	63.6	76.3
GP	0.88	0.86	67.0	79.2	0.88	0.85	68.2	80.8
Water_set_narrow								
MLR	0.60	0.88	61.2	76.3	0.59	0.90	62.5	76.4
ANN	0.66	0.83	67.3	79.8	0.71	0.77	68.6	82.7
SVM	0.70	0.76	68.6	82.9	0.71	0.76	69.8	83.0

PLS	0.60	0.88	61.4	76.1	0.59	0.90	62.5	76.1
RF	0.67	0.80	64.7	80.5	0.68	0.79	64.5	82.0
ET	0.69	0.77	66.4	82.3	0.70	0.76	67.1	82.7
Bag	0.67	0.80	64.8	81.1	0.68	0.79	65.4	81.8
GP	0.70	0.77	68.0	82.7	0.69	0.77	69.1	82.0
Ethanol_set								
MLR	0.39	0.87	59.9	77.1	0.39	0.88	59.7	76.4
ANN	0.51	0.80	67.8	81.6	0.55	0.78	69.2	81.7
SVM	0.54	0.76	69.8	83.6	0.54	0.76	70.1	82.5
PLS	0.39	0.87	59.7	76.7	0.39	0.88	59.6	76.7
RF	0.54	0.76	68.5	82.2	0.55	0.75	68.3	82.9
ET	0.54	0.76	69.1	81.7	0.55	0.76	68.8	82.7
Bag	0.55	0.76	69.1	82.7	0.55	0.76	67.9	82.0
GP	0.53	0.77	68.8	82.0	0.52	0.77	67.9	82.2
Benzene_set								
MLR	0.47	0.79	68.8	81.3	0.48	0.79	66.1	78.0
ANN	0.58	0.72	71.4	84.5	0.57	0.73	70.0	85.3
SVM	0.57	0.72	70.7	84.5	0.57	0.71	69.3	83.2
PLS	0.47	0.80	67.5	81.3	0.47	0.79	65.5	78.2
RF	0.62	0.67	72.2	87.3	0.62	0.67	71.7	86.4
ET	0.64	0.66	72.7	88.4	0.62	0.67	70.7	87.1
Bag	0.62	0.67	71.4	87.5	0.62	0.67	71.1	86.4
GP	0.57	0.72	70.1	83.8	0.58	0.71	70.9	83.8

Acetone_set								
MLR	0.47	0.77	69.2	81.4	0.48	0.77	69.7	82.3
ANN	0.54	0.73	71.7	83.4	0.55	0.72	72.5	86.3
SVM	0.57	0.70	76.3	84.3	0.58	0.70	75.9	84.5
PLS	0.47	0.77	68.8	81.6	0.48	0.77	69.2	82.5
RF	0.57	0.69	74.8	85.4	0.56	0.71	73.4	85.2
ET	0.56	0.70	73.7	84.5	0.57	0.70	72.8	84.7
Bag	0.57	0.70	73.7	84.9	0.56	0.71	72.6	84.9
GP	0.52	0.74	72.6	83.4	0.56	0.71	75.9	85.0

Table 19. Comparison of fixed train test split results for original DFT PCM compared to energy descriptors replaced with those obtained with HF SMD method

Method	R^2	RMSE	%LogS±0.7	%LogS±1.0	R^2	RMSE	%LogS±0.7	%LogS±1.0
Water_set_wide								
	**Original DFT PCM Procedure**				**Energy Descriptors Replaced by HF SMD**			
MLR	0.80	1.16	50.5	65.3	0.79	1.17	49.5	65.3
ANN	0.90	0.84	58.9	78.9	0.91	0.81	68.4	84.2
SVM	0.89	0.85	71.6	78.9	0.90	0.82	72.6	83.2
PLS	0.80	1.16	51.6	66.3	0.80	1.17	49.5	66.3
RF	0.90	0.83	60.0	75.8	0.90	0.82	63.2	80.0
ET	0.93	0.71	66.3	84.2	0.93	0.70	69.5	84.2
Bag	0.90	0.82	57.9	76.8	0.90	0.83	65.3	81.1
GP	0.88	0.89	68.4	73.7	0.90	0.80	70.5	82.1

Water_set_narrow								
MLR	0.68	0.82	61.7	80.0	0.66	0.84	63.5	76.5
ANN	0.74	0.74	68.7	84.3	0.78	0.68	70.4	82.6
SVM	0.76	0.71	65.2	81.7	0.79	0.67	68.7	85.2
PLS	0.68	0.83	61.7	80.0	0.66	0.85	62.6	76.5
RF	0.72	0.76	60.9	82.6	0.74	0.74	61.7	83.5
ET	0.75	0.73	66.1	80.9	0.77	0.71	67.0	81.7
Bag	0.72	0.77	60.9	81.7	0.74	0.74	61.7	84.3
GP	0.76	0.71	73.0	81.7	0.79	0.66	73.9	81.7
Ethanol_set								
MLR	0.29	0.98	50.7	72.5	0.29	0.98	52.8	71.8
ANN	0.49	0.88	64.1	76.8	0.49	0.87	63.4	74.6
SVM	0.51	0.81	64.1	78.9	0.52	0.80	64.1	77.5
PLS	0.29	0.99	51.4	71.8	0.29	0.99	52.8	73.2
RF	0.53	0.79	64.8	82.4	0.54	0.78	64.1	83.1
ET	0.50	0.81	62.7	78.9	0.52	0.80	64.1	79.6
Bag	0.52	0.80	65.5	79.6	0.53	0.79	65.5	81.0
GP	0.51	0.80	66.2	77.5	0.54	0.78	64.8	77.5
Benzene_set								
MLR	0.64	0.66	75.5	86.2	0.56	0.70	74.5	81.9
ANN	0.67	0.63	77.7	88.3	0.66	0.63	74.5	89.4
SVM	0.71	0.58	76.6	89.4	0.69	0.60	77.7	87.2
PLS	0.64	0.66	74.5	85.1	0.54	0.72	73.4	83.0

RF	0.72	0.57	76.6	90.4	0.70	0.59	73.4	89.4
ET	0.75	0.54	76.6	90.4	0.71	0.58	78.7	88.3
Bag	0.72	0.57	75.5	89.4	0.70	0.58	74.5	88.3
GP	0.70	0.58	79.8	90.4	0.69	0.59	79.8	88.3
Acetone_set								
MLR	0.36	0.87	60.9	78.3	0.37	0.86	65.2	79.3
ANN	0.42	0.87	67.4	79.3	0.46	0.82	68.5	82.6
SVM	0.42	0.83	72.8	81.5	0.43	0.82	72.8	81.5
PLS	0.35	0.87	62.0	78.3	0.36	0.87	65.2	78.3
RF	0.40	0.84	62.0	80.4	0.39	0.84	64.1	79.3
ET	0.40	0.84	63.0	78.3	0.40	0.84	65.2	77.2
Bag	0.41	0.83	62.0	80.4	0.39	0.84	63.0	80.4
GP	0.42	0.83	68.5	84.8	0.44	0.81	68.5	82.6

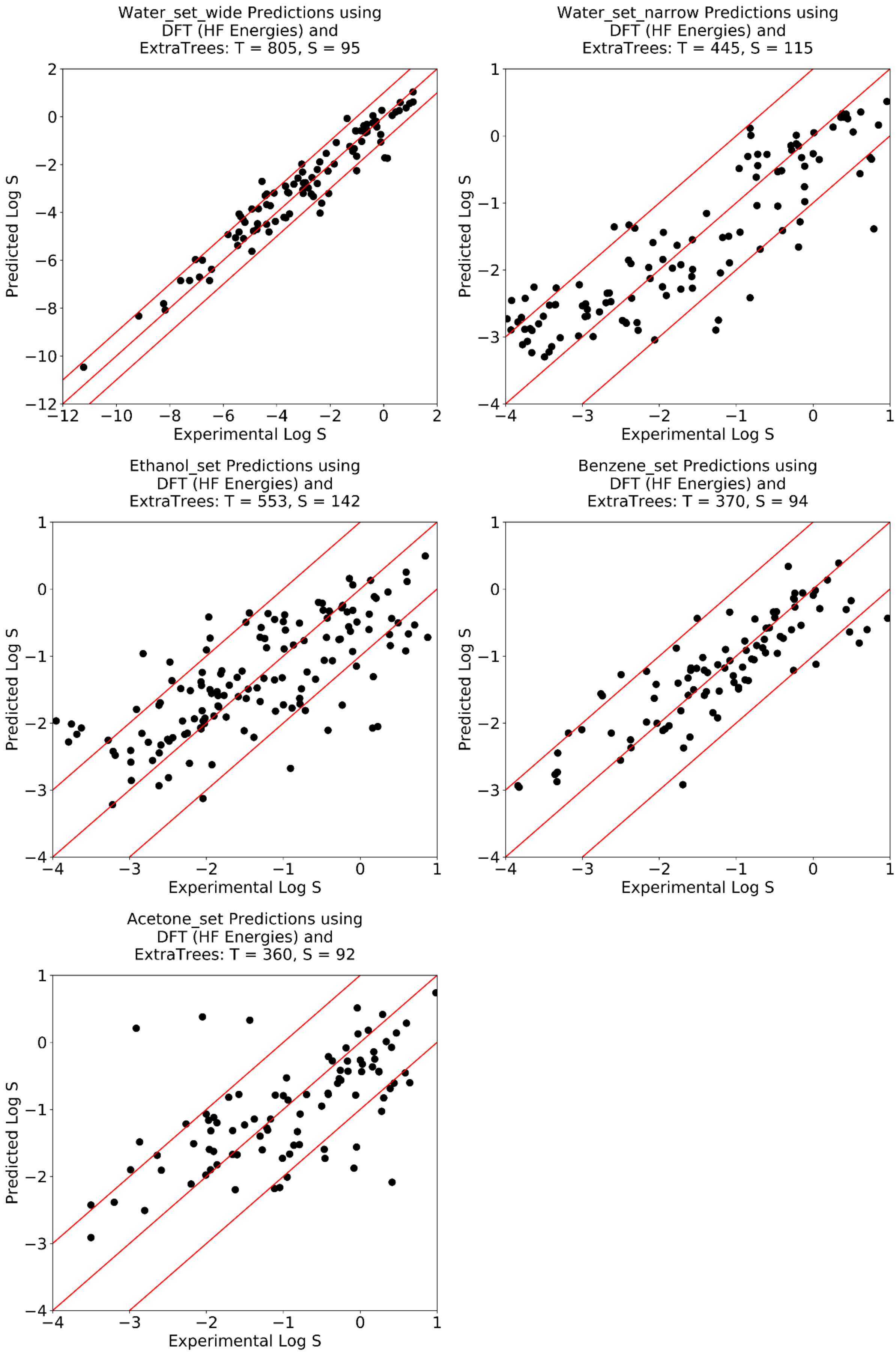
Water_set_wide Predictions using
DFT (HF Energies) and
ExtraTrees: T = 805, S = 95
Predicted Log S
Experimental Log S
Water_set_narrow Predictions using
DFT (HF Energies) and
ExtraTrees: T = 445, S = 115
Predicted Log S
Experimental Log S
Ethanol_set Predictions using
DFT (HF Energies) and
ExtraTrees: T = 553, S = 142
Predicted Log S
Experimental Log S
Benzene_set Predictions using
DFT (HF Energies) and
ExtraTrees: T = 370, S = 94
Predicted Log S
Experimental Log S
Acetone_set Predictions using
DFT (HF Energies) and
ExtraTrees: T = 360, S = 92
Predicted Log S
Experimental Log S

Figure 65. Plots for fixed train test split using HF SMD energy descriptors

4.11 Consensus predictions

The four best machine learning methods established with the initial models were: ANN (single hidden layer of 300 nodes); SVM (with optimised parameters); ET (500 trees); and GP. Each of their predictions were averaged by median and mean. The results are summarised in **Table 20** for the explicit train test split, with plots in **Figure 66** and **Figure 67**.

Table 20. ET *vs* consensus predictions for explicit train test split

Dataset	Method	R^2	RMSE	% LogS±0.7	% LogS±1.0	MPD
Water_set_wide	ET	**0.93**	**0.71**	66.3	**84.2**	**291**
Water_set_wide	Consensus Median	0.90	0.80	**68.4**	78.9	472
Water_set_wide	Consensus Mean	0.91	0.77	66.3	77.9	399
Water_set_narrow	ET	0.75	0.73	66.1	80.9	277
Water_set_wide	Consensus Median	**0.78**	0.69	71.3	**85.2**	**205**
Water_set_wide	Consensus Mean	**0.78**	**0.68**	**73.0**	**85.2**	**205**
Ethanol_set	ET	0.50	0.81	62.7	78.9	427
Ethanol_set	Consensus Median	0.53	0.79	**65.5**	**79.6**	442
Ethanol_set	Consensus Mean	**0.54**	**0.78**	64.1	**79.6**	**415**
Benzene_set	ET	0.75	0.54	76.6	90.4	**163**
Benzene_set	Consensus Median	0.76	0.53	78.7	**91.5**	175
Benzene_set	Consensus Mean	**0.77**	**0.52**	**81.9**	90.4	168
Acetone_set	ET	0.40	0.84	63.0	78.3	1872
Acetone_set	Consensus Median	0.42	0.82	70.7	81.5	1936
Acetone_set	Consensus Mean	**0.44**	**0.81**	**71.7**	**82.6**	**1852**

Table 21. Standard deviation of training sets *vs* RMSE

Dataset	SD Full Set	SD Training Set	SD Test Set	Consensus mean RMSE	Consensus median RMSE
Water_set_wide	2.44	2.42	2.55	0.77	0.80
Water_set_wide (LogS = -4 - 1)	2.44	2.42	1.36	0.72	0.77
Water_set_narrow	1.40	1.39	1.43	0.68	0.69
Ethanol_set	1.12	1.11	1.15	0.78	0.79
Benzene_set	1.08	1.09	1.06	0.52	0.53
Acetone_set	1.06	1.06	1.07	0.81	0.82

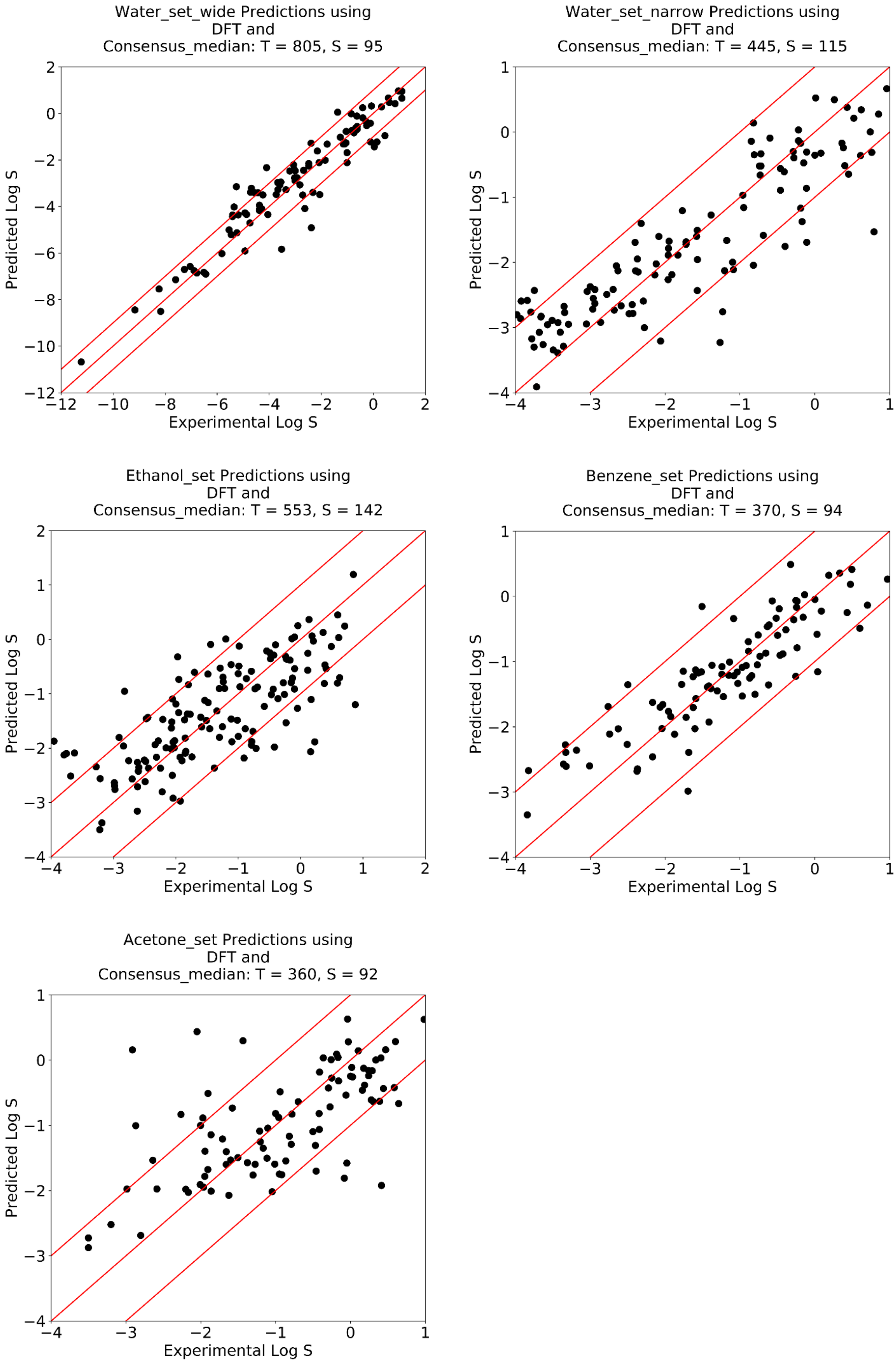

Figure 66. Plots of predictions based on median prediction of ANN, SVM, ET and GP algorithms

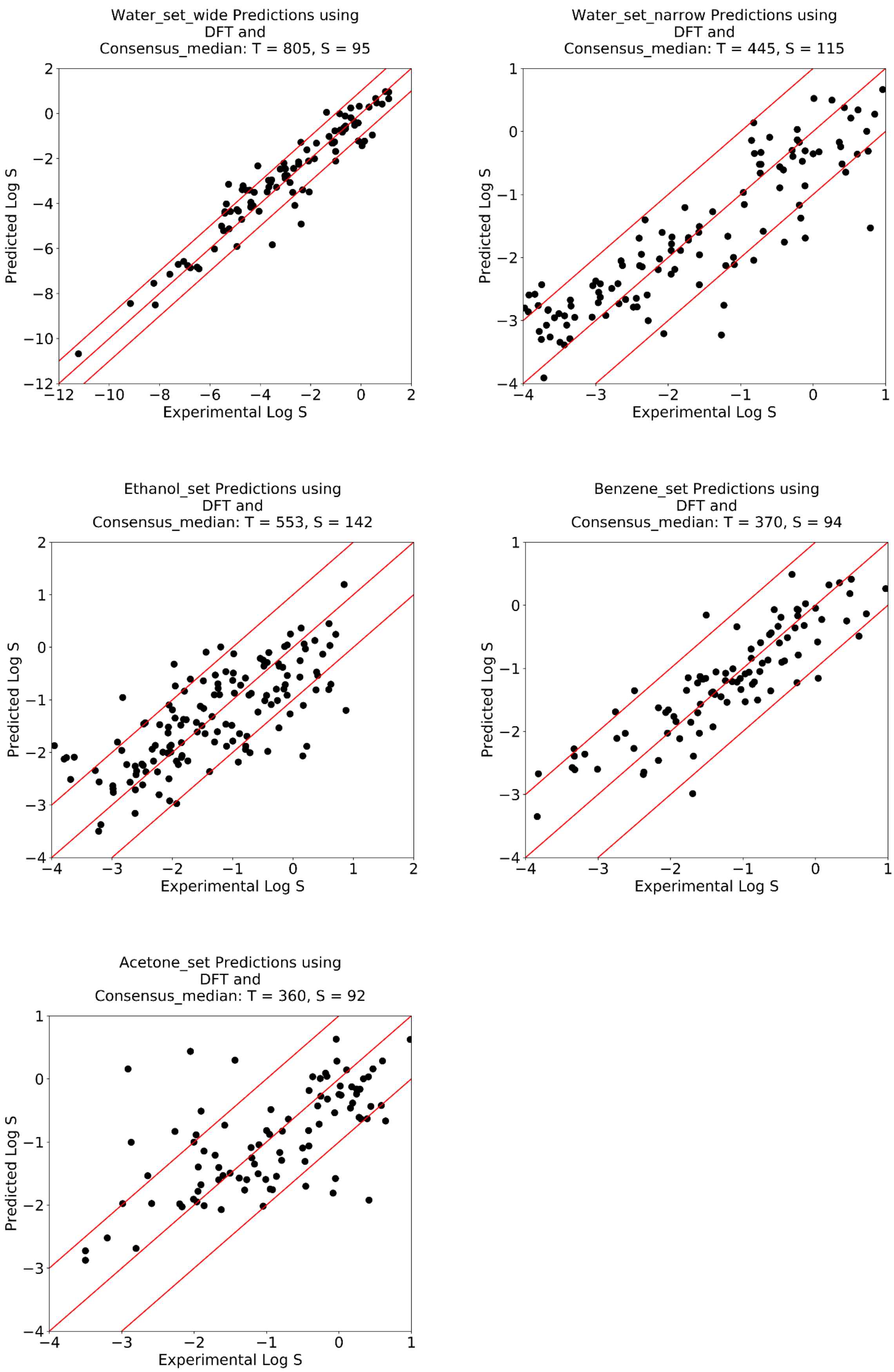

Figure 67. Plots of predictions based on mean prediction of ANN, SVM, ET and GP algorithms

4.12 Rapid models with PM6 descriptors

Descriptors calculation was approximately 100 times faster for PM6 compared to DFT method. The median CPU time for ***Benzene_set*** molecules to be optimised in the gas phase is 198 minutes using B3LYP/6-31+G(d) and 5 minutes using PM6. An exponential increase in computational time compared to molecular weight was observed for DFT, while no trend was observed with PM6. **Figure 68** shows the computational time compared to molecular weight for Benzene dataset molecules in the gas phase.

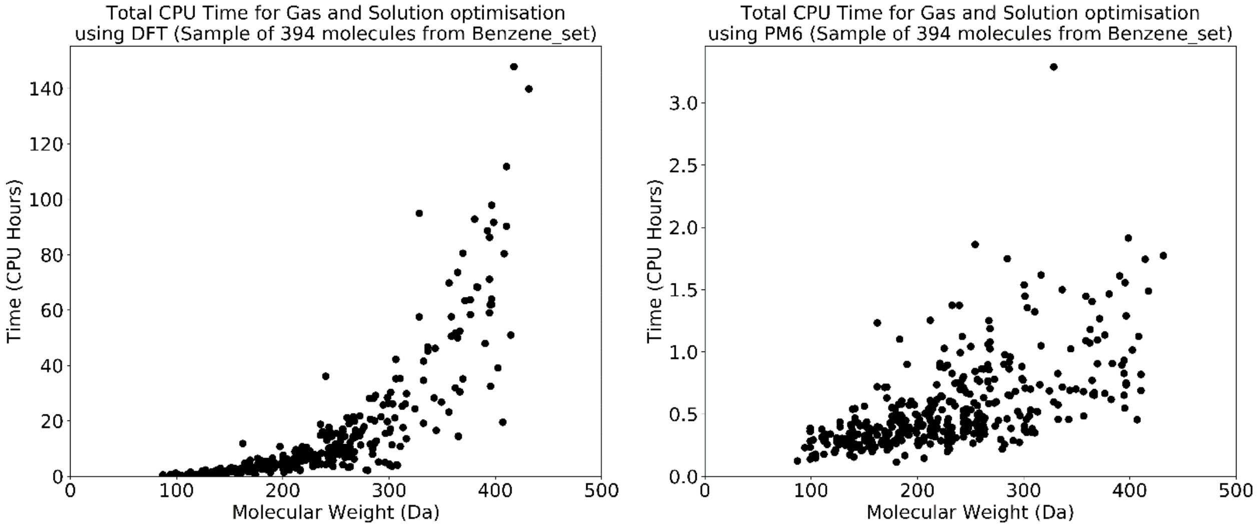

Figure 68. CPU time vs molecular weight for ***Benzene_set*** geometry optimisation

PM6 prediction models were similarly built for the 5 datasets with 14 descriptors. The results are summarised in **Table 22**, **Table 23** and **Figure 69**.

Table 22. Summary of 10-fold cross validation results using DFT and PM6 descriptors

Method	R^2	RMSE	%LogS±0.7	%LogS±1.0	R^2	RMSE	%LogS±0.7	%LogS±1.0
Water_set_wide								
	**DFT Descriptors**				**PM6 Descriptors**			
MLR	0.77	1.16	52.1	67.0	0.76	1.20	49.6	63.2
ANN	0.87	0.89	64.9	79.9	0.87	0.86	66.2	80.9
SVM	0.88	0.85	68.3	81.7	0.87	0.86	70.1	81.1
PLS	0.77	1.16	52.2	66.9	0.76	1.20	49.2	63.1

RF	0.84	0.95	62.3	75.7	0.84	0.98	60.3	74.8
ET	0.87	0.88	66.3	79.4	0.86	0.91	64.1	77.8
Bag	0.84	0.95	62.1	76.4	0.84	0.98	61.2	74.9
GP	0.88	0.86	67.0	79.2	0.87	0.87	69.7	81.6
Water_set_narrow								
MLR	0.60	0.88	61.2	76.3	0.56	0.93	58.0	73.8
ANN	0.66	0.83	67.3	79.8	0.68	0.81	69.5	83.0
SVM	0.70	0.76	68.6	82.9	0.71	0.76	68.8	84.5
PLS	0.60	0.88	61.4	76.1	0.56	0.93	58.2	72.7
RF	0.67	0.80	64.7	80.5	0.65	0.83	62.5	77.3
ET	0.69	0.77	66.4	82.3	0.68	0.79	63.2	80.5
Bag	0.67	0.80	64.8	81.1	0.65	0.83	61.8	77.7
GP	0.70	0.77	68.0	82.7	0.70	0.77	67.7	84.5
Ethanol_set								
MLR	0.39	0.87	59.9	77.1	0.42	0.86	62.6	76.7
ANN	0.51	0.80	67.8	81.6	0.53	0.78	69.4	82.6
SVM	0.54	0.76	69.8	83.6	0.54	0.76	72.2	84.6
PLS	0.39	0.87	59.7	76.7	0.42	0.85	62.7	77.1
RF	0.54	0.76	68.5	82.2	0.50	0.79	66.5	80.7
ET	0.54	0.76	69.1	81.7	0.51	0.79	67.6	81.0
Bag	0.55	0.76	69.1	82.7	0.49	0.80	66.8	80.1
GP	0.53	0.77	68.8	82.0	0.50	0.79	67.6	81.4
Benzene_set								

MLR	0.47	0.79	68.8	81.3	0.47	0.80	66.6	77.6
ANN	0.58	0.72	71.4	84.5	0.58	0.71	71.3	83.8
SVM	0.57	0.72	70.7	84.5	0.57	0.73	71.5	84.0
PLS	0.47	0.80	67.5	81.3	0.47	0.81	66.0	78.3
RF	0.62	0.67	72.2	87.3	0.59	0.70	70.1	84.3
ET	0.64	0.66	72.7	88.4	0.64	0.66	72.0	88.2
Bag	0.62	0.67	71.4	87.5	0.59	0.70	69.9	84.3
GP	0.57	0.72	70.1	83.8	0.57	0.72	72.4	83.2
Acetone_set								
MLR	0.47	0.77	69.2	81.4	0.48	0.76	69.5	83.4
ANN	0.54	0.73	71.7	83.4	0.51	0.75	71.3	86.3
SVM	0.57	0.70	76.3	84.3	0.52	0.74	74.3	84.7
PLS	0.47	0.77	68.8	81.6	0.48	0.76	69.9	83.6
RF	0.57	0.69	74.8	85.4	0.51	0.74	72.3	84.7
ET	0.56	0.70	73.7	84.5	0.51	0.75	73.7	84.1
Bag	0.57	0.70	73.7	84.9	0.51	0.75	70.8	84.1
GP	0.52	0.74	72.6	83.4	0.55	0.73	72.6	84.5

Table 23. Summary of explicit test set metrics for DFT and PM6 descriptors

Method	R^2	RMSE	%LogS±0.7	%LogS±1.0	R^2	RMSE	%LogS±0.7	%LogS±1.0
Water_set_wide								
	**DFT Descriptors**				**PM6 Descriptors**			

MLR	0.80	1.16	50.5	65.3	0.77	1.22	45.3	58.9
ANN	0.90	0.84	58.9	78.9	0.89	0.83	67.4	82.1
SVM	0.89	0.85	71.6	78.9	0.90	0.81	70.5	82.1
PLS	0.80	1.16	51.6	66.3	0.77	1.22	46.3	58.9
RF	0.90	0.83	60.0	75.8	0.89	0.86	62.1	76.8
ET	0.93	0.71	66.3	84.2	0.90	0.82	63.2	75.8
Bag	0.90	0.82	57.9	76.8	0.89	0.85	61.1	75.8
GP	0.88	0.89	68.4	73.7	0.89	0.87	68.4	82.1
Water_set_narrow								
MLR	0.68	0.82	61.7	80.0	0.62	0.88	61.7	78.3
ANN	0.74	0.74	68.7	84.3	0.73	0.76	67.8	79.1
SVM	0.76	0.71	65.2	81.7	0.72	0.77	65.2	80.0
PLS	0.68	0.83	61.7	80.0	0.61	0.90	60.9	76.5
RF	0.72	0.76	60.9	82.6	0.70	0.80	59.1	77.4
ET	0.75	0.73	66.1	80.9	0.72	0.77	64.3	77.4
Bag	0.72	0.77	60.9	81.7	0.70	0.80	58.3	77.4
GP	0.76	0.71	73.0	81.7	0.74	0.73	67.0	81.7
Ethanol_set								
MLR	0.29	0.98	50.7	72.5	0.28	1.00	56.3	71.8
ANN	0.49	0.88	64.1	76.8	0.35	1.02	57.7	73.9
SVM	0.51	0.81	64.1	78.9	0.43	0.88	65.5	77.5
PLS	0.29	0.99	51.4	71.8	0.28	1.00	56.3	72.5
RF	0.53	0.79	64.8	82.4	0.40	0.89	60.6	76.8

ET	0.50	0.81	62.7	78.9	0.41	0.88	62.0	78.2
Bag	0.52	0.80	65.5	79.6	0.41	0.89	59.2	77.5
GP	0.51	0.80	66.2	77.5	0.39	0.91	61.3	75.4
Benzene_set								
MLR	0.64	0.66	75.5	86.2	0.59	0.68	73.4	81.9
ANN	0.67	0.63	77.7	88.3	0.68	0.64	80.9	92.6
SVM	0.71	0.58	76.6	89.4	0.67	0.61	79.8	88.3
PLS	0.64	0.66	74.5	85.1	0.59	0.69	72.3	81.9
RF	0.72	0.57	76.6	90.4	0.68	0.61	76.6	88.3
ET	0.75	0.54	76.6	90.4	0.73	0.56	75.5	91.5
Bag	0.72	0.57	75.5	89.4	0.67	0.61	75.5	87.2
GP	0.70	0.58	79.8	90.4	0.71	0.57	81.9	88.3
Acetone_set								
MLR	0.36	0.87	60.9	78.3	0.39	0.85	67.4	77.2
ANN	0.42	0.87	67.4	79.3	0.38	0.90	71.7	79.3
SVM	0.42	0.83	72.8	81.5	0.44	0.81	70.7	85.9
PLS	0.35	0.87	62.0	78.3	0.39	0.85	67.4	77.2
RF	0.40	0.84	62.0	80.4	0.39	0.84	62.0	79.3
ET	0.40	0.84	63.0	78.3	0.40	0.84	65.2	81.5
Bag	0.41	0.83	62.0	80.4	0.40	0.84	60.9	80.4
GP	0.42	0.83	68.5	84.8	0.42	0.82	68.5	83.7

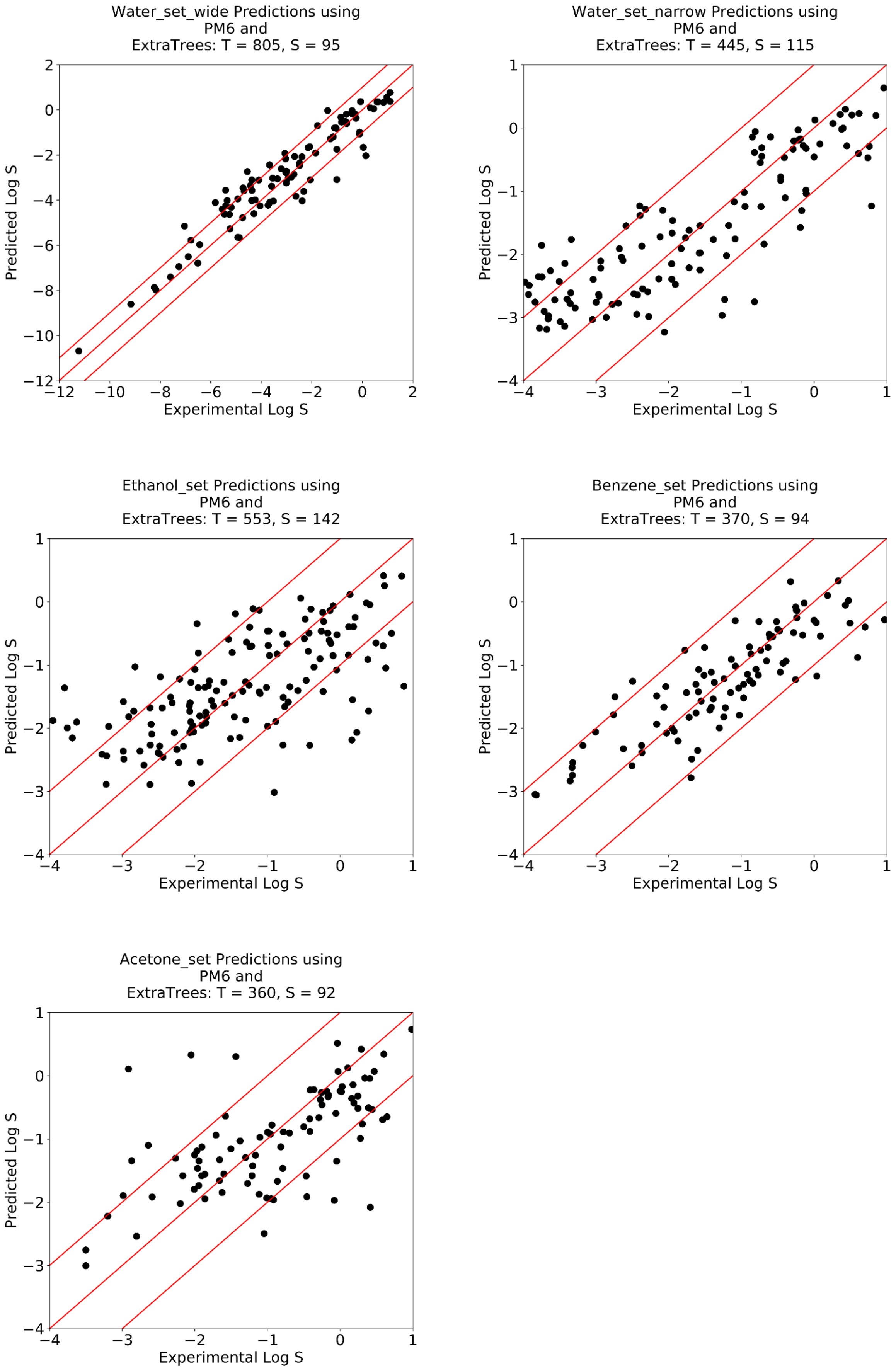

Figure 69. Explicit test set prediction plots using PM6 descriptors

4.13 Inclusion of conformation

The ***rdkit*** Python module was used to generate 20 conformations for each molecule.[17] These conformations were optimised (PM6), and all descriptors were calculated for each of them. Three descriptor sets were obtained per molecule:

(i) ***Set A*** already calculated above using the initial optimised structure without conformational analysis;

(ii) ***Set B*** from the single lowest energy conformer of the 20 generated;

(iii) ***Set C*** calculated by averaging 20 individual conformational sets with weighting based on Boltzmann distributed populations.

For ***Set C***, for each conformation *Exp* is calculated as in equation 1, where E_0 is in J/mol, k is the Boltzmann constant, *T* is the temperature, and *Av* is Avogadro's constant. Then the partition function, *Q*, is calculated as the sum of *Exp* from each molecule, as shown in equation 2. Lastly the % weighting is calculated as a fraction of *Exp* over the partition function, *Q*. Each descriptor is then weighted according to the % composition, as shown in equation 3.

$$Exp = \exp\left(\frac{-E_0}{kTAv}\right) \tag{1}$$

$$Q = \sum_{i=0}^{n} Exp \tag{2}$$

$$\% = \left(\frac{Exp}{Q}\right) * 100 \tag{3}$$

These were performed for Benzene_set and Ethanol_set, only in PM6 (due to demanding computational resources). The results are summarised in **Table 24**, **Table 25** and **Figure 70**.

Table 24. Summary of 10-fold cross validation resulting using three different conformation protocols

Dataset	Method	Descriptors	R^2	RMSE	%LogS±0.7	%LogS±1.0
Ethanol_set	ET	Set A	0.54	0.76	69.1	81.7
Ethanol_set	ET	Set B	0.52	0.78	69.0	82.7
Ethanol_set	ET	Set C	0.50	0.80	68.3	81.1
Benzene_set	ET	Set A	0.64	0.66	72.7	88.4
Benzene_set	ET	Set B	0.58	0.69	69.4	84.9
Benzene_set	ET	Set C	0.59	0.69	69.8	85.6

Table 25. Summary of fixed train test split results using three different conformation protocols

Dataset	Method	Descriptors	R^2	RMSE	%LogS±0.7	%LogS±1.0
Ethanol_set	ET	Set A	0.50	0.81	62.7	78.9
Ethanol_set	ET	Set B	0.42	0.88	63.4	76.8
Ethanol_set	ET	Set C	0.42	0.88	62.7	78.9
Benzene_set	ET	Set A	0.75	0.54	76.6	90.4
Benzene_set	ET	Set B	0.71	0.58	75.5	88.3
Benzene_set	ET	Set C	0.71	0.58	74.5	88.3

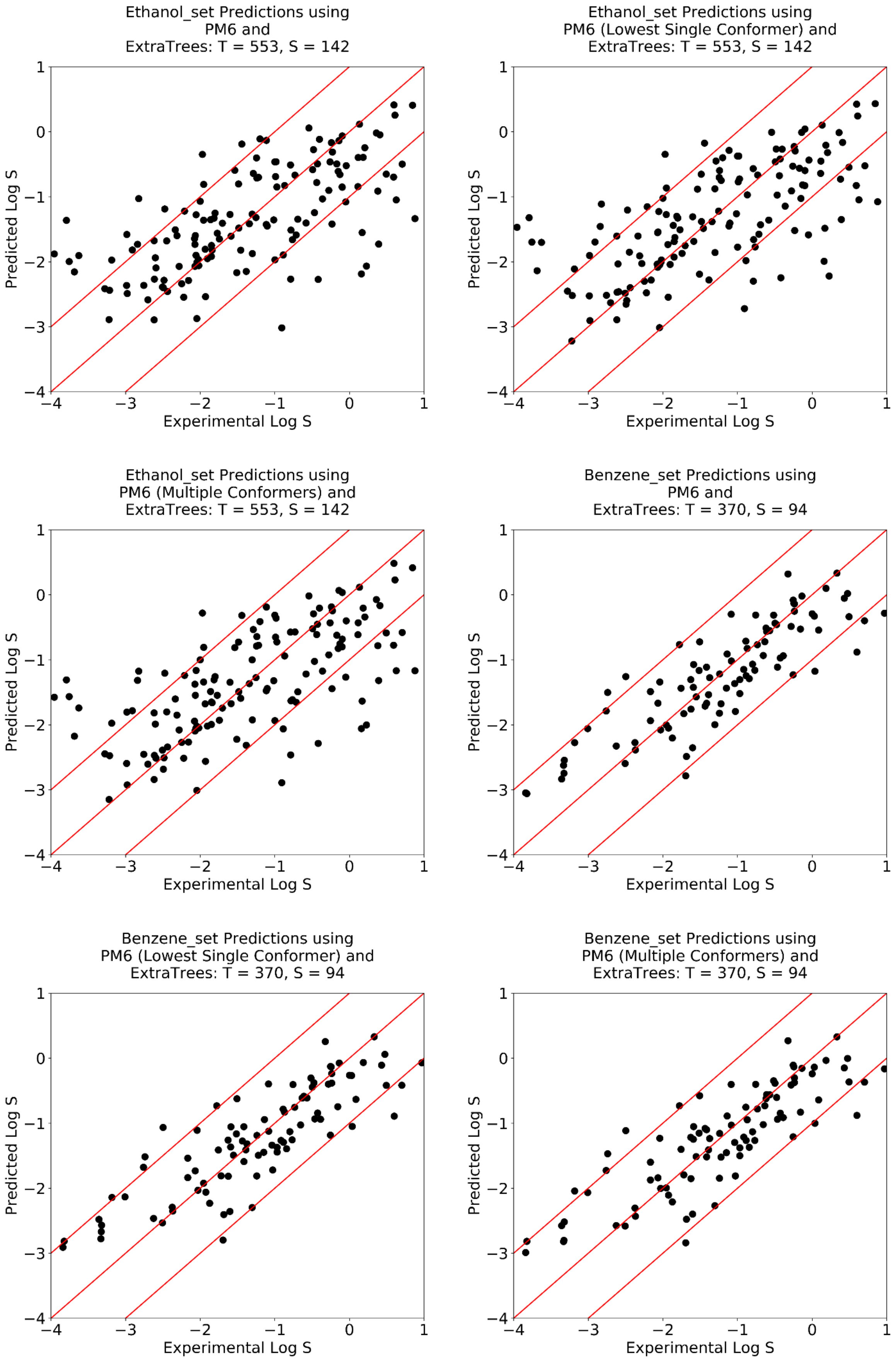

Figure 70. Plots of the different conformation protocols for fixed train test split

Supplementary Note 5: Benchmarking

Benchmarking were performed using solubility predictions of the same test sets using 4 solubility prediction tools: AquaSol, EPI Suite, COSMOtherm and General Solubility Equation (GSE). For AquaSol and EPI Suite, only aqueous solubilities were predicted and evaluated. For COSMOtherm, solubilities in water, ethanol, benzene, and acetone were predicted and evaluated.

5.1 AquaSol & EPI Suite

AquaSol is a deep neural network tool, with only 2D input given.[18] EPI Suite is a software created by the US EPA.[19] It predicts solubility using fragments, with two protocols: fragments to predict log P if not available and then solubility is predicted (EPI Suite 1); and calculating solubility directly with fragments (EPI Suite 2). This comparison is shown in **Figure 71**. It is worth noting that AQUASOL and EPISUITE give almost instant predictions; the higher accuracy of our models is offset with the longer calculation times to generate 3D structures with electronic structure methods. Metrics and plots are shown in **Table 26** and **Figure 71**.

Table 26. Metrics for predictions using our approach (ET) and AQUASOL/EPI Suite

Dataset	Method	R^2	RMSE	%LogS±0.7	%LogS±1.0
Water_set_wide	ET	0.93	0.71	66.3	84.2
Water_set_wide	AQUASOL	0.86	1.08	56.8	71.6
Water_set_wide	EPI Suite 1	0.83	1.09	62.1	73.7
Water_set_wide	EPI Suite 2	0.80	1.28	57.9	66.3

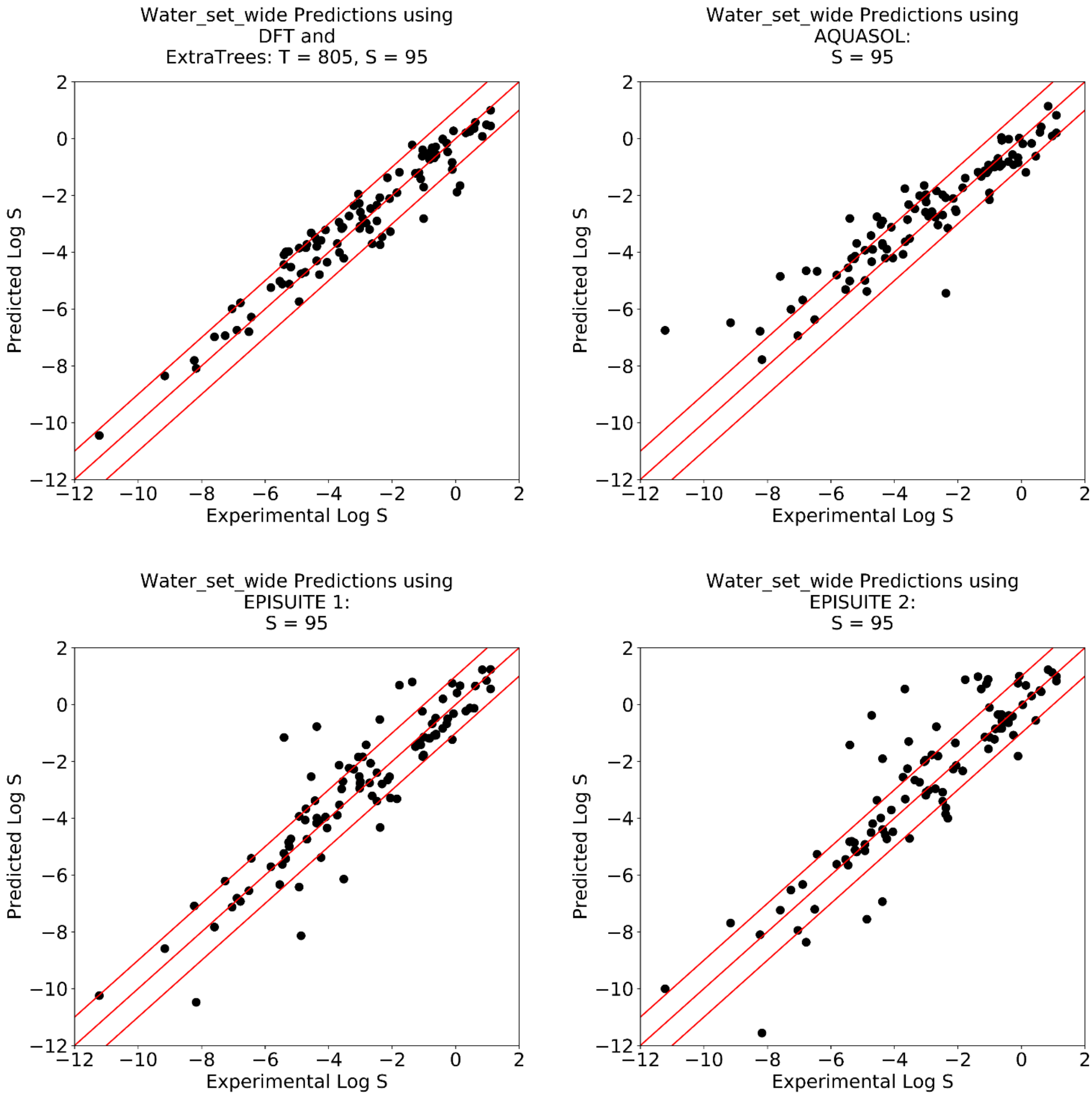

Figure 71. Comparison between solubility predictions using ET and AquaSol, EPI Suite 1 and EPI Suite 2.

5.2 COSMO-RS

COSMOtherm[20,21] uses COMSO-RS[22–24] to predict solubility using a mixture of DFT, statistical modelling and physical chemistry. Within the framework there are many options to enhance predictions, including providing solubility in several solvents before predicting in another or supplying experimental data. For this comparison, COSMOtherm was only allowed the SMILES of the test set and a QSPR method is used to estimate lattice energy. As part of the procedure, a 3D structure is built and various conformers optimised using DFT (BP-TZVPD-FINE method). These conformers, with the sublimation energy QSPR prediction, are used to obtain the solubility prediction. **Figure 72** shows the COSMOtherm predictions for our test sets; note that COSMOtherm was unable to provide predictions for a small number of the molecules, denoting them as “too soluble to measure”; these molecules are

omitted from the plots and metrics (for both COSMOtherm and our approach). This is shown in **Table 27** and **Figure 72**. Details are molecules removed are shown in **Table 28**.

Table 27. Comparison of metrics obtained by COSMOtherm and our approach (ET)

Dataset	Method	R^2	RMSE	%LogS±0.7	%LogS±1.0
Water_set_wide	ET	0.92	0.73	64.8	81.3
Water_set_wide	COSMOtherm	0.75	1.34	51.6	71.4
Water_set_narrow	ET	0.75	0.73	67.0	82.6
Water_set_narrow	COSMOtherm	0.52	1.06	60.6	71.6
Ethanol_set	ET	0.54	0.80	67.5	78.0
Ethanol_set	COSMOtherm	0.10	1.64	30.9	39.0
Benzene_set	ET	0.75	0.54	76.9	91.0
Benzene_set	COSMOtherm	0.22	1.42	47.4	57.7
Acetone_set	ET	0.43	0.82	64.9	83.8
Acetone_set	COSMOtherm	0.20	1.62	50.0	64.9

Table 28. Molecules removed from test sets for COSMOtherm comparison

Dataset	StdInChIKey/Unique Identifer	SMILES	Reason for removal
Water_set_wide	FOANIXZHAMJWOI-UHFFFAOYSA-N	Brc1ccc(C(O)(C(=O)OC(C)C)c2ccc(Br)cc2)cc1	Error in COSMOtherm calculation
Water_set_wide	GETQZCLCWQTVFV-UHFFFAOYSA-N	N(C)(C)C	COSMOtherm designated molecule as "too soluble to measure"
Water_set_wide	MLUCVPSAIODCQM-UHFFFAOYSA-N	O=CC=CC	COSMOtherm designated

			molecule as “too soluble to measure”
Water_set_wide	NBBJYMSMWIIQGU-UHFFFAOYSA-N	O=CCC	COSMOtherm designated molecule as “too soluble to measure”
Water_set_narrow	LUALIOATIOESLM-UHFFFAOYSA-N	c1ccc2c(c1)N(CCCN1CCC(CC1)O)c1cc(ccc1S2)C#N	Error in COSMOtherm calculation
Water_set_narrow	BRLQWZUYTZBJKN-UHFFFAOYSA-N	C(C1CO1)Cl	COSMOtherm designated molecule as “too soluble to measure”
Water_set_narrow	JUHORIMYRDESRB-UHFFFAOYSA-N	c1ccc(cc1)CNCCNCc1ccccc1	COSMOtherm designated molecule as “too soluble to measure”
Water_set_narrow	WEHWNAOGRSTTBQ-UHFFFAOYSA-N	CCCNCCC	COSMOtherm designated molecule as “too soluble to measure”
Water_set_narrow	WEVYAHXRMPXWCK-UHFFFAOYSA-N	CC#N	COSMOtherm designated molecule as “too soluble to measure”
Water_set_narrow	XNLICIUVMPYHGG-UHFFFAOYSA-N	CCCC(=O)C	COSMOtherm designated molecule as

			"too soluble to measure"
Ethanol_set	KJPXTDBDRLXUQN-UHFFFAOYSA-N	CC(=O)Oc1ccc(cc1C(=O)O)n1nc2ccccc2n1	Error in COSMOtherm calculation
Ethanol_set	RRKTZKIUPZVBMF-IBTVXLQLSA-N	COc1cc2c(cc1OC)N1C(=O)C[C@H]3[C@@H]4[C@H]5C[C@H]6[C@]2(CCN6CC5=CCO3)[C@@H]14	Error in COSMOtherm calculation
Ethanol_set	ons36	c1ccc2c(c1)C(=O)c1cccc(c1C2=O)Cl	Error in COSMOtherm calculation
Ethanol_set	GZLPBJKBZMALAG-OCOZRVBESA-N	c1ccc(cc1)Nc1ccc(cc1)/N=N/c1ccc(c2ccccc12)N(=O)=O	Error in COSMOtherm calculation
Ethanol_set	HCXVJBMSMIARIN-PHZDYDNGSA-N	CC[C@H](/C=C/[C@@H](C)[C@H]1CC[C@H]2[C@@H]3CC=C4C[C@H](CC[C@]4(C)[C@H]3CC[C@]12C)O)C(C)C	Error in COSMOtherm calculation
Ethanol_set	JWBYUXWEHYCVHA-BVYOUEPQSA-N	C[C@@H](Cc1ccccc1)[N](=O)=[N](=O)[C@H](C)Cc2ccccc2	Error in COSMOtherm calculation
Ethanol_set	LUYAMNYBNTVQJG-UHFFFAOYSA-N	C(CS(=O)(=O)CCCl)Cl	Error in COSMOtherm calculation
Ethanol_set	UTQSUDKKBZEXHH-UHFFFAOYSA-N	c1ccc(c(c1)Cl)Nc1ccc(cc1N)Cl	Error in COSMOtherm calculation
Ethanol_set	WOUBWJAJSXAOIV-UHFFFAOYSA-N	c1cc(ccc1S(=O)(=O)N)S(=O)(=O)N	Error in COSMOtherm calculation

Ethanol_set	QRBSSDMZLRKOGR-OCEACIFDSA-N	c1ccc(cc1)N=Nc1ccc(cc1)N(c1ccccc1)c1cccc c1	Error in COSMOtherm calculation
Ethanol_set	LPEPZBJOKDYZAD-UHFFFAOYSA-N	c1ccc(c(c1)C(=O)O)Nc1cccc(c1)C(F)(F)F	Error in COSMOtherm calculation
Ethanol_set	DTWMUZVZPXOTII-UHFFFAOYSA-N	Cc1cc(C)c(c(c1[N+](=O)[O-])[N+](=O)[O-])[N+](=O)[O-]	COSMOtherm designated molecule as “too soluble to measure”
Ethanol_set	JAKVEOCMEMGHGB-YFKPBYRVSA-N	C#CCSC[C@@H](C(=O)O)N	COSMOtherm designated molecule as “too soluble to measure”
Ethanol_set	KTFCASILCGCMLM-UHFFFAOYSA-N	C(CCCCCNCCCCCCCN)CCCCC(=O)O	COSMOtherm designated molecule as “too soluble to measure”
Ethanol_set	QWZBEFCPZJWDKC-UHFFFAOYSA-N	CCCCCCCCCCCCCCCC(=O)OC(=O)CCCC CCCCCCCCCCC	COSMOtherm designated molecule as “too soluble to measure”
Ethanol_set	RFVNOJDQRGSOEL-UHFFFAOYSA-N	CCCCCCCCCCCCCCCCCC(=O)OCCO	COSMOtherm designated molecule as “too soluble to measure”
Ethanol_set	RTOHDISBHWDUCE-UHFFFAOYSA-N	C=CCc1ccc(c(c1)OC)OCCSCCOc1ccc(CC=C)cc1OC	COSMOtherm designated molecule as

			“too soluble to measure”
Ethanol_set	TWRPLCZREDAZEZ-DVKNGEFBSA-N	C([C@@H]1[C@H]([C@@H]([C@H]([C@@H](O1)O[N+](=O)[O-])O[N+](=O)[O-])O[N+](=O)[O-])O[N+](=O)[O-])O[N+](=O)[O-]	COSMOtherm designated molecule as “too soluble to measure”
Ethanol_set	UCAHGXFXBRSTPX-UHFFFAOYSA-N	c1cc(c(cc1[N+](=O)[O-])[N+](=O)[O-])Oc1c(cc(cc1[N+](=O)[O-])[N+](=O)[O-])[N+](=O)[O-]	COSMOtherm designated molecule as “too soluble to measure”
Benzene_set	JLYXXMFPNIAWKQ-CDRYSYESSA-N	Cl[C@@H]1[C@@H](Cl)[C@H](Cl)[C@@H](Cl)[C@H](Cl)[C@H]1Cl	Error in COSMOtherm calculation
Benzene_set	IKCLCGXPQILATA-UHFFFAOYSA-N	OC(=O)c1ccccc1Cl	Error in COSMOtherm calculation
Benzene_set	VKCNNDPZFOVURD-SECBINFHSA-N	C[C@@H](C(O)=O)c1cccc2ccccc12	Error in COSMOtherm calculation
Benzene_set	YJHZQWSSAFUAIG-UHFFFAOYSA-N	Cc1cc(ccc1O)N=Nc2ccccc2	Error in COSMOtherm calculation
Benzene_set	RRKTZKIUPZVBMF-IBTVXLQLSA-N	COc1cc2N3[C@H]4[C@@H]5[C@H](CC3=O)OCC=C6CN7CC[C@]4([C@@H]7C[C@H]56)c2cc1OC	Error in COSMOtherm calculation
Benzene_set	JHWNWJKBPDFINM-UHFFFAOYSA-N	O=C1CCCCCCCCCCCN1	Error in COSMOtherm calculation

Benzene_set	VOXZDWNPVJITMN-ZBRFXRBCSA-N	C[C@]12CC[C@H]3[C@@H](CCc4cc(O)ccc34)[C@@H]1CC[C@@H]2O	Error in COSMOtherm calculation
Benzene_set	JBWKIWSBJXDJDT-UHFFFAOYSA-N	ClC(c1ccccc1)(c2ccccc2)c3ccccc3	Error in COSMOtherm calculation
Benzene_set	ABLACSIRCKEUOB-UHFFFAOYSA-N	Cc1cc(O)c2C(=O)c3c(O)cc(O)c4C(=O)C(C)(C)c5cc(O)c1c2c5c34	COSMOtherm designated molecule as “too soluble to measure”
Benzene_set	IQUPABOKLQSFBK-UHFFFAOYSA-N	Oc1ccccc1[N+]([O-])=O	COSMOtherm designated molecule as “too soluble to measure”
Benzene_set	JHMMNVQXKFUCCZ-VOTSOKGWSA-N	[O-][N+](=O)c1cc(c(\C=C\c2ccccc2)c(c1)[N+]([O-])=O)[N+]([O-])=O	COSMOtherm designated molecule as “too soluble to measure”
Benzene_set	LAWHHRXCBUNWFI-UHFFFAOYSA-N	CCCCCC(C(O)=O)C(O)=O	COSMOtherm designated molecule as “too soluble to measure”
Benzene_set	NODIXMQDAGGDJQ-UHFFFAOYSA-N	C(=CC=CC=Cc1ccccc1)C=CC=Cc2ccccc2	COSMOtherm designated molecule as “too soluble to measure”

Benzene_set	PBQLPYWWRPIKQW-UHFFFAOYSA-N	[O-][N+](=O)c1cc(c(ON=C(c2ccccc2)c3ccccc3)c(c1)[N+]([O-])=O)[N+]([O-])=O	COSMOtherm designated molecule as "too soluble to measure"
Benzene_set	PPJVBBJMDLANLB-UHFFFAOYSA-N	[O-][N+](=O)c1ccc(c2c1cccc2[N+]([O-])=O)[N+]([O-])=O	COSMOtherm designated molecule as "too soluble to measure"
Benzene_set	YDCFNPFXXJRYCZ-UHFFFAOYSA-N	[O-][N+](=O)c1cc(c(C(=O)N=[N+]=[N-])c(c1)[N+]([O-])=O)[N+]([O-])=O	COSMOtherm designated molecule as "too soluble to measure"
Acetone_set	AGUIVNYEYSCPNI-UHFFFAOYSA-N	CN(C1=C(C=C(C=C1N(=O)=O)N(=O)=O)N(=O)=O)N(=O)=O	COSMOtherm designated molecule as "too soluble to measure"
Acetone_set	AOZXETLGPZAZNK-UHFFFAOYSA-N	CCOC1=C(C=C(C=C1N(=O)=O)N(=O)=O)N(=O)=O	COSMOtherm designated molecule as "too soluble to measure"
Acetone_set	GOUHYARYYWKXHS-UHFFFAOYSA-N	OC(=O)C1=CC=C(C=O)C=C1	COSMOtherm designated molecule as "too soluble to measure"
Acetone_set	HCTVORVYPUEFSX-UHFFFAOYSA-N	CN(C)C1=CC2=C(NC3=C(C(=C(N(C)N(=O)=O)C(=C3)N(=O)=O)N(=O)=O)S2(=O)=O)C=C1	COSMOtherm designated molecule as

			"too soluble to measure"
Acetone_set	HEFNNWSXXWATRW-UHFFFAOYSA-N	CC(C)CC1=CC=C(C=C1)C(C)C(O)=O	COSMOtherm designated molecule as "too soluble to measure"
Acetone_set	HSXJIIOATUZQAZ-UHFFFAOYSA-N	O=C(OCC(COC(=O)C1=CC=C(C=C1)N(=O)=O)OC(=O)C1=CC=C(C=C1)N(=O)=O)C1=CC=CC=C1	COSMOtherm designated molecule as "too soluble to measure"
Acetone_set	IAHOUQOWMXVMEH-UHFFFAOYSA-N	NC1=C(C=C(C=C1N(=O)=O)N(=O)=O)N(=O)=O	COSMOtherm designated molecule as "too soluble to measure"
Acetone_set	IBSNKSODLGJUMQ-SDNWHVSQSA-N	CO\C=C(\C(=O)OC)C1=C(COC2=NC(=CC=C2)C(F)(F)F)C=CC=C1	COSMOtherm designated molecule as "too soluble to measure"
Acetone_set	LIKSPRLIPHRBCL-UHFFFAOYSA-N	CCCCCCCCCCCCCC(=O)CCCCCCCCC(O)=O	COSMOtherm designated molecule as "too soluble to measure"
Acetone_set	NHRIWEKOARVHSN-UHFFFAOYSA-N	COC1=C(C(=CC(=C1N(=O)=O)N(=O)=O)N(=O)=O)N(=O)=O	COSMOtherm designated molecule as "too soluble to measure"
Acetone_set	NOIRTYMOEFHJRM-UHFFFAOYSA-N	COCN1C(=O)C2=C(C=CC=C2)C1=O	COSMOtherm designated

			molecule as “too soluble to measure”
Acetone_set	NRDWUPPIGBHWAS-ZJUUUORDSA-N	CCC[C@H]1CC2=C(C[C@@H](CC)CC3=C1C(=O)OC3=O)C(=O)OC2=O	COSMOtherm designated molecule as “too soluble to measure”
Acetone_set	SLAMLWHELXOEJZ-UHFFFAOYSA-N	OC(=O)C1=CC=CC=C1N(=O)=O	COSMOtherm designated molecule as “too soluble to measure”
Acetone_set	UATJOMSPNYCXIX-UHFFFAOYSA-N	O=N(=O)C1=CC(=CC(=C1)N(=O)=O)N(=O)=O	COSMOtherm designated molecule as “too soluble to measure”
Acetone_set	XGDRLCRGKUCBQL-UHFFFAOYSA-N	N#CC1=C(N=CN1)C#N	COSMOtherm designated molecule as “too soluble to measure”
Acetone_set	XNGJUGUTYMLWIM-UHFFFAOYSA-N	CC1(C)C2CCC1(CBr)C(=O)C2Br	COSMOtherm designated molecule as “too soluble to measure”
Acetone_set	XQXPVVBIMDBYFF-UHFFFAOYSA-N	OC(=O)CC1=CC=C(O)C=C1	COSMOtherm designated molecule as “too soluble to measure”

Acetone_set	ZCJLOOJRNPHKAV-UHFFFAOYSA-N	OC(=O)C=CC1=CC=CO1	COSMOtherm designated molecule as “too soluble to measure”

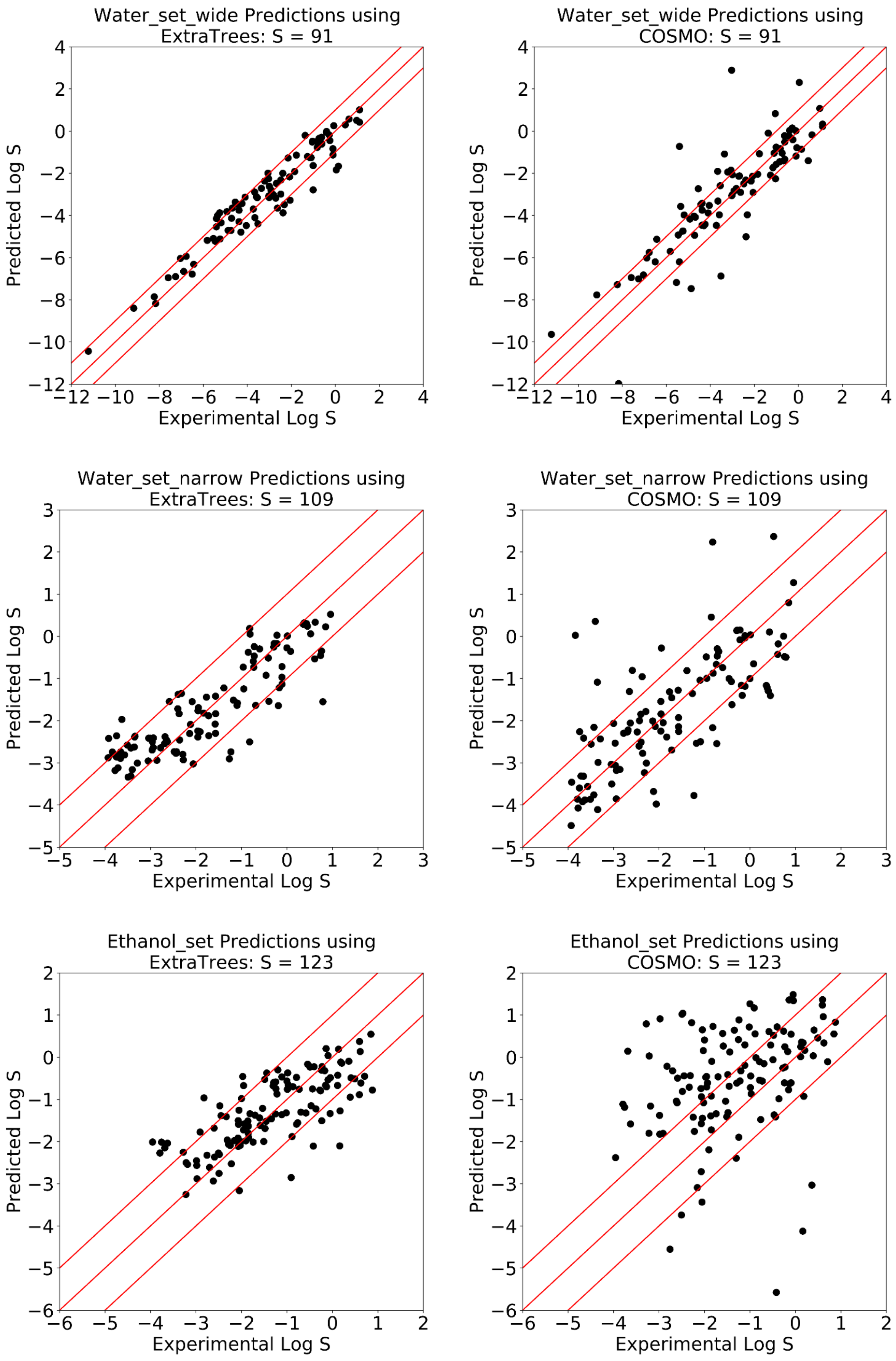
Water_set_wide Predictions using
ExtraTrees: S = 91
Predicted Log S
Experimental Log S
Water_set_wide Predictions using
COSMO: S = 91
Predicted Log S
Experimental Log S
Water_set_narrow Predictions using
ExtraTrees: S = 109
Predicted Log S
Experimental Log S
Water_set_narrow Predictions using
COSMO: S = 109
Predicted Log S
Experimental Log S
Ethanol_set Predictions using
ExtraTrees: S = 123
Predicted Log S
Experimental Log S
Ethanol_set Predictions using
COSMO: S = 123
Predicted Log S
Experimental Log S

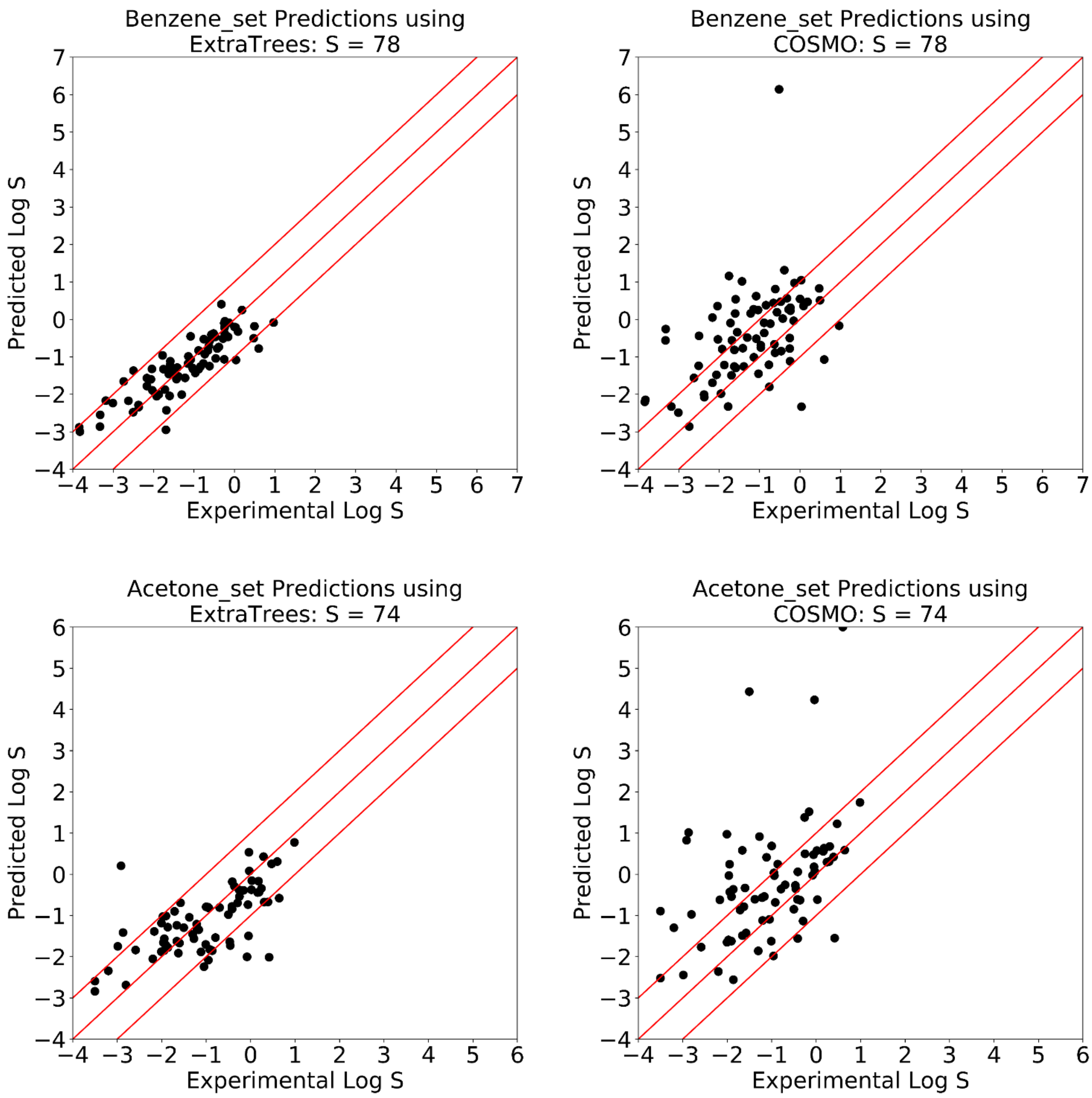

Figure 72. Comparison between solubility predictions using COSMOtherm and ET

5.3 General Solubility Equation (GSE)

The General Solubility Equation (GSE) is a basic QSPR equation that relates log P and m.p. to aqueous solubility,[25] and often used as a baseline for solubility predictions due to its simplicity. Experimental m.p. and calculated log P (clogP, calculated in ACD Labs 2018.2, Advanced Chemistry Development, Inc., Toronto, ON, Canada, www.acdlabs.com, 2019) were used with equation 6 and our internal test set as a comparison. The predictions are much worse than our models, as seen in **Table 29** and **Figure 73**.

$$\log S = -0.01(m.p. - 25) - \log P + 0.5 \tag{6}$$

Table 29. Comparison of predictions using our approach (ET) and GSE

Dataset	Method	R^2	RMSE	%LogS±0.7	%LogS±1.0
Water_set_wide	ET	0.93	0.71	66.3	84.2
Water_set_wide	GSE	0.77	1.35	49.5	62.1
Water_set_narrow	ET	0.75	0.73	66.1	80.9
Water_set_narrow	GSE	0.54	1.19	47.8	62.6

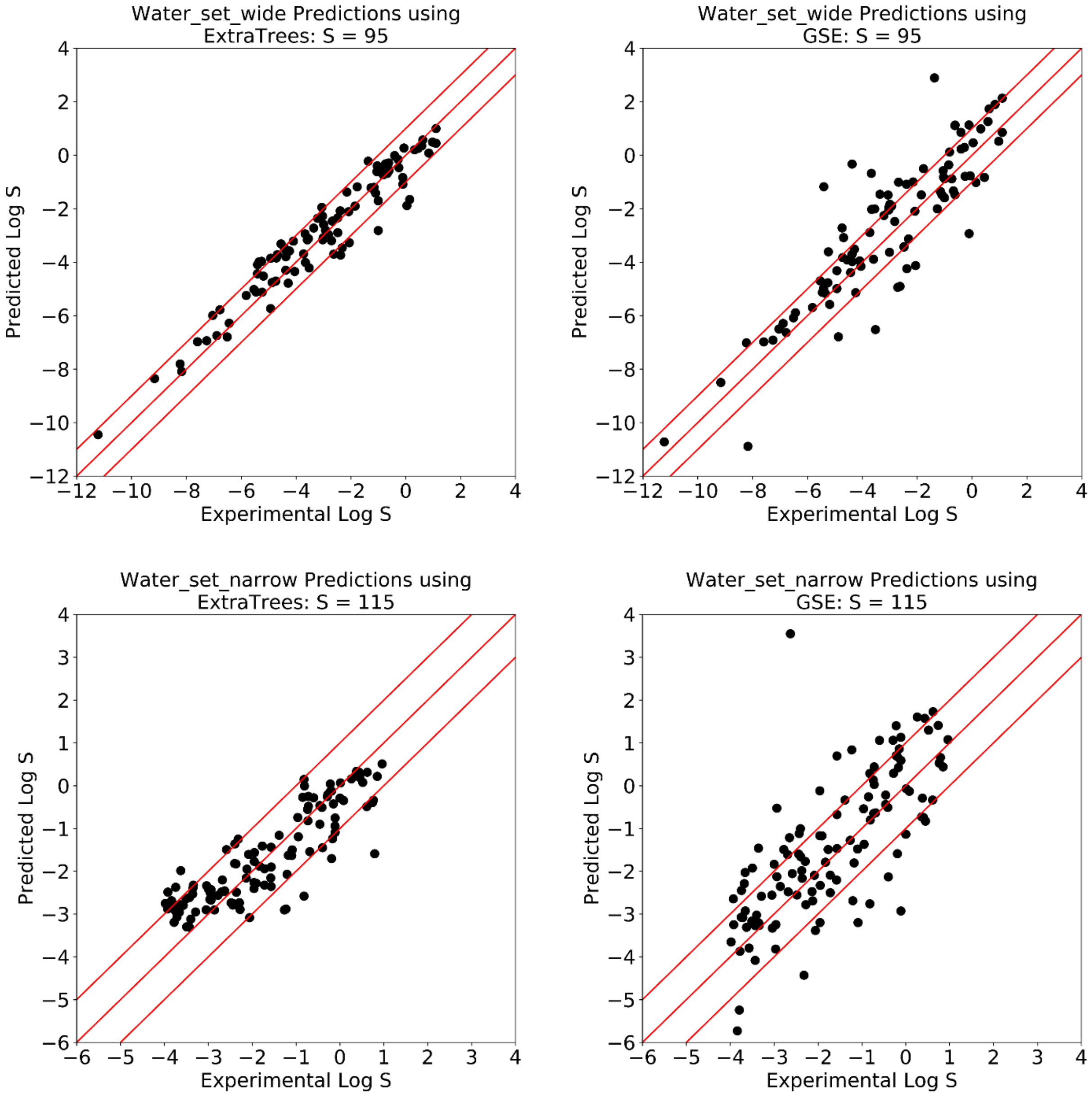

Figure 73. Comparison between solubility predictions using ET and GSE

Supplementary Note 6: Validation with external data

6.1 Solubility challenge

The Solubility Challenge (2008) external test set ***SC*** contained 75 molecules in water taken from the test and training sets within that study.[26] A high proportion of ionisable groups was apparent from a simple comparison of the functional groups present in ***SC*** and ***Water_set_wide***, as shown in **Figure 74**.

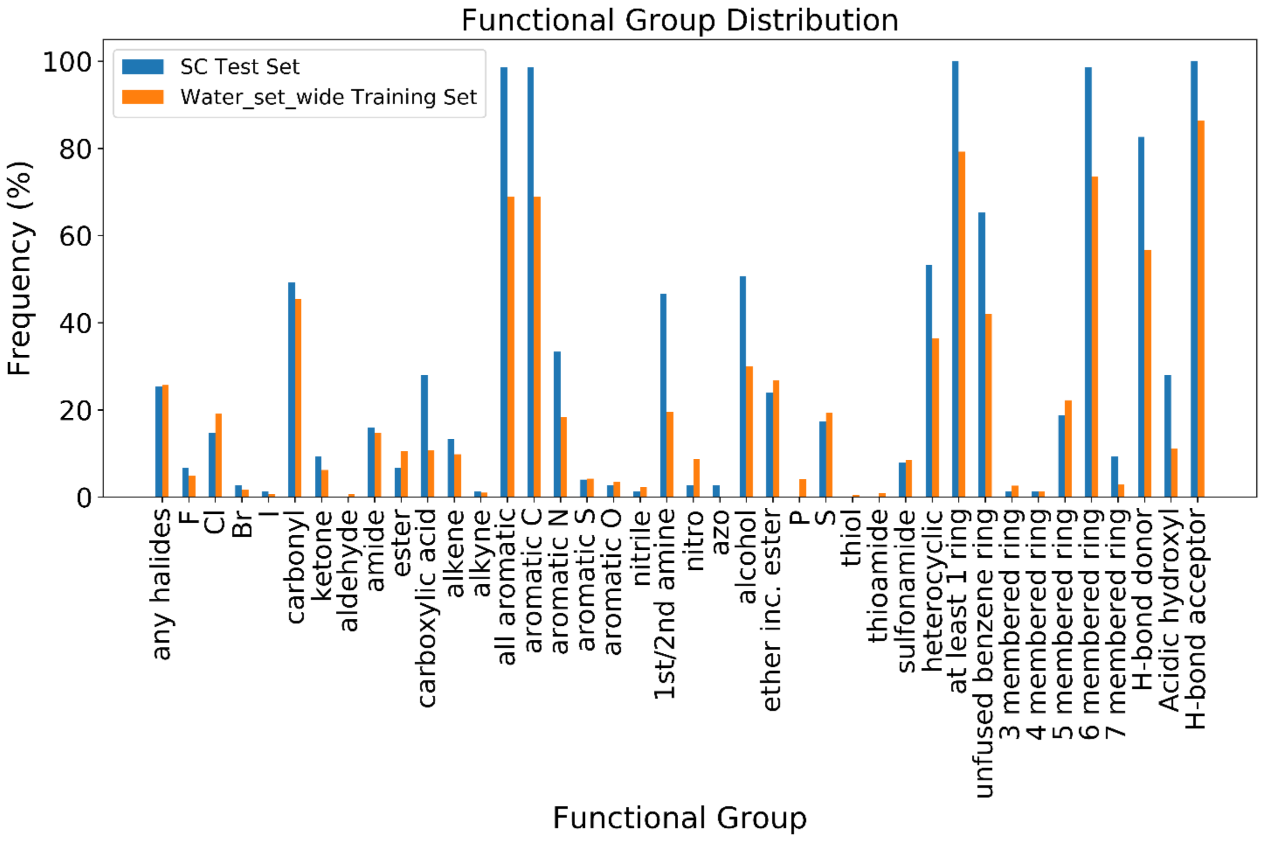

Figure 74. Functional group analysis for the Solubility Challenge external dataset compared to ***Water_set_wide*** training set

The BertzCT complexity index was calculated for both the ***SC*** test set and ***Water_Set_wide*** training set, this is shown in **Figure 75**. ***SC*** test set contained more complex molecular structures than our training set.

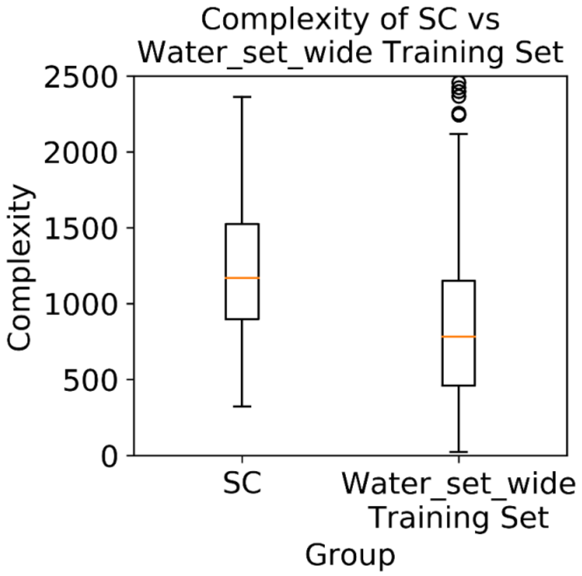

Figure 75. Mean BertzCT complexity for SC test set and Water_set_wide training set. Error bars show 1 standard deviation

Metrics and graphs for the predictions are shown in **Table 30** and **Figure 76** to **Figure 79**. They were recalculated with the following: excluding carboxylic acids; excluding amines; and excluding both carboxylic acids and amines. Predictions are only acceptable when amines were excluded.

Table 30. Metrics for ***SC*** test set. Predictions are significantly improvement if amines are excluded

Method	Modification?	Size	R^2	RMSE	%LogS±0.7	%LogS±1.0
ANN	Full set	75	0.36	1.15	57.3	**69.3**
SVM	Full set	75	**0.39**	1.10	56.0	65.3
ET	Full set	75	0.26	1.23	49.3	61.3
GP	Full set	75	0.35	1.19	56.0	65.3
Consensus Median	Full set	75	**0.39**	**1.09**	58.7	68.0
Consensus Mean	Full set	75	0.38	1.10	**60.0**	68.0
ANN	Carboxylic Acids Omitted	54	0.29	1.16	61.1	**70.4**
SVM	Carboxylic Acids Omitted	54	**0.34**	1.11	57.4	64.8

ET	Carboxylic Acids Omitted	54	0.25	1.10	51.9	63.0
GP	Carboxylic Acids Omitted	54	0.30	1.15	59.3	66.7
Consensus Median	Carboxylic Acids Omitted	54	0.32	1.09	61.1	66.7
Consensus Mean	Carboxylic Acids Omitted	54	0.32	**1.07**	**63.0**	66.7
ANN	Amines Omitted	23	0.70	**0.71**	**69.6**	**82.6**
SVM	Amines Omitted	23	0.70	0.73	60.9	**82.6**
ET	Amines Omitted	23	0.54	1.03	60.9	73.9
GP	Amines Omitted	23	**0.71**	0.72	65.2	**82.6**
Consensus Median	Amines Omitted	23	0.70	0.72	**69.6**	78.3
Consensus Mean	Amines Omitted	23	0.69	0.75	**69.6**	78.3
ANN	Both carboxylic Acids and Amines Omitted	14	**0.81**	**0.60**	**78.6**	**85.7**
SVM	Both carboxylic Acids and Amines Omitted	14	0.76	0.70	64.3	**85.7**
ET	Both carboxylic Acids and Amines Omitted	14	0.69	0.79	71.4	71.4
GP	Both carboxylic Acids and Amines Omitted	14	0.80	0.63	71.4	**85.7**

Consensus Median	Both carboxylic Acids and Amines Omitted	14	0.79	0.64	**78.6**	78.6
Consensus Mean	Both carboxylic Acids and Amines Omitted	14	0.80	0.62	**78.6**	78.6

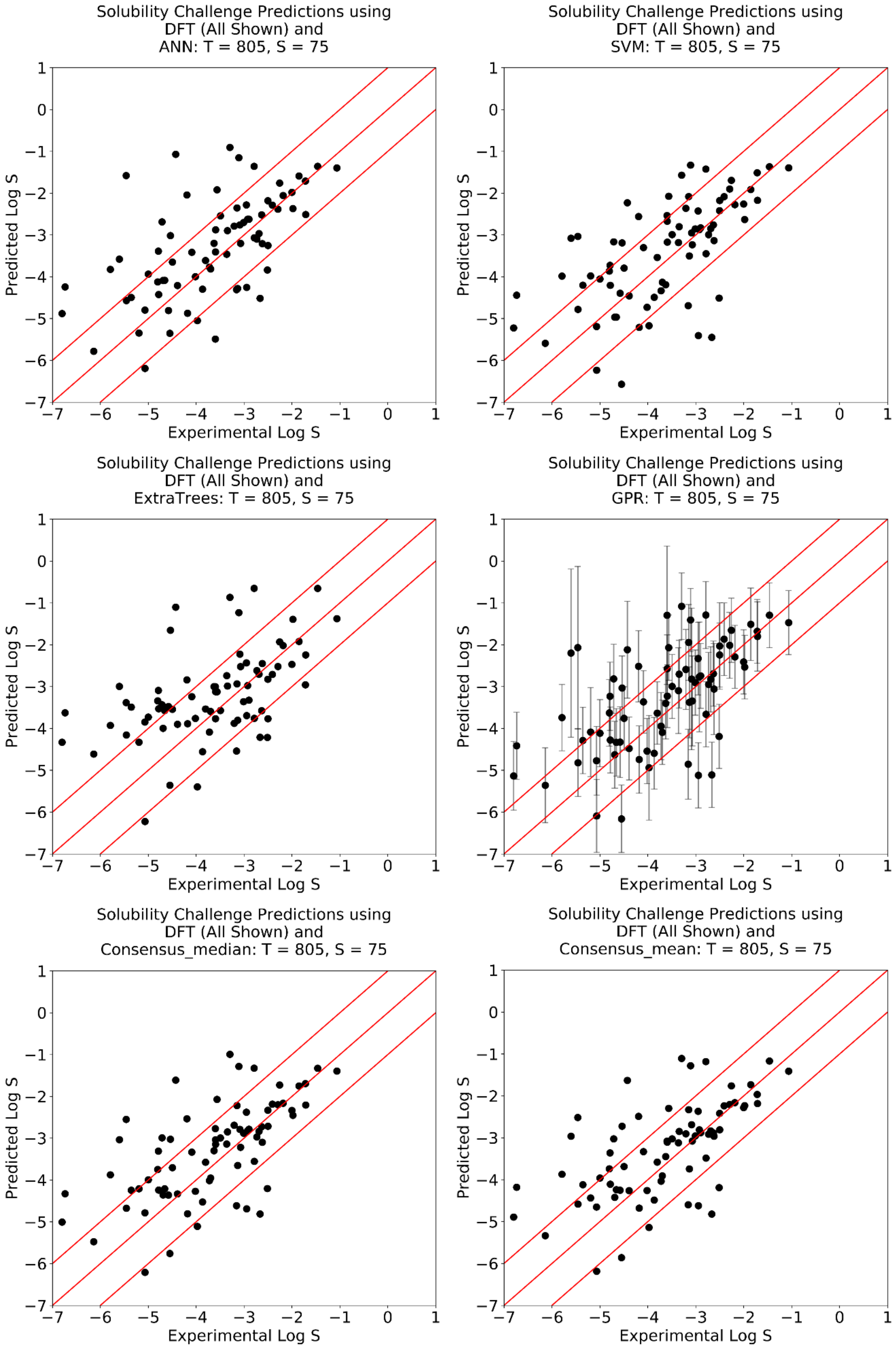

Figure 76. ***SC test*** predictions, all shown

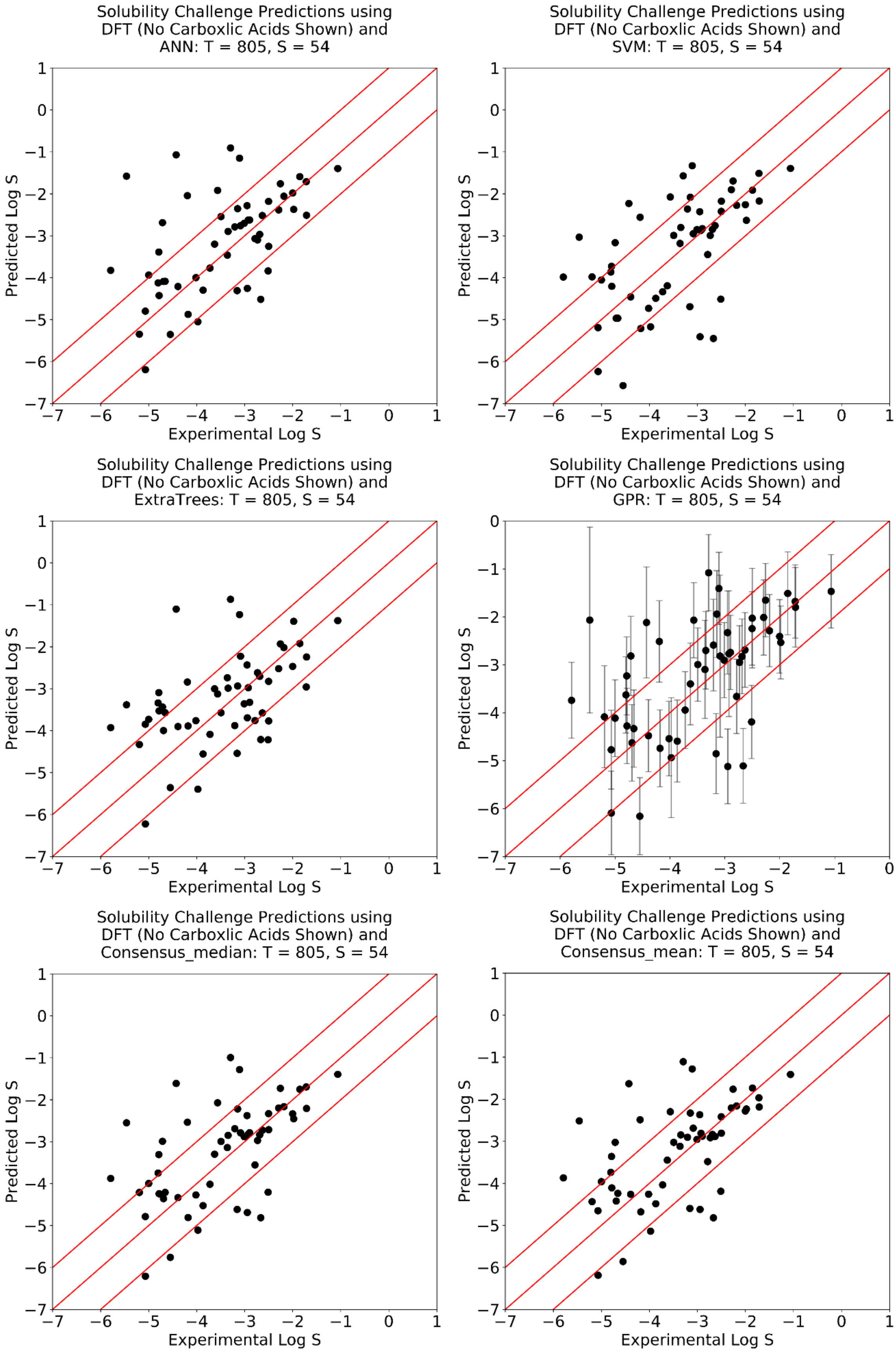

Figure 77. ***SC test*** predictions, carboxylic acids omitted

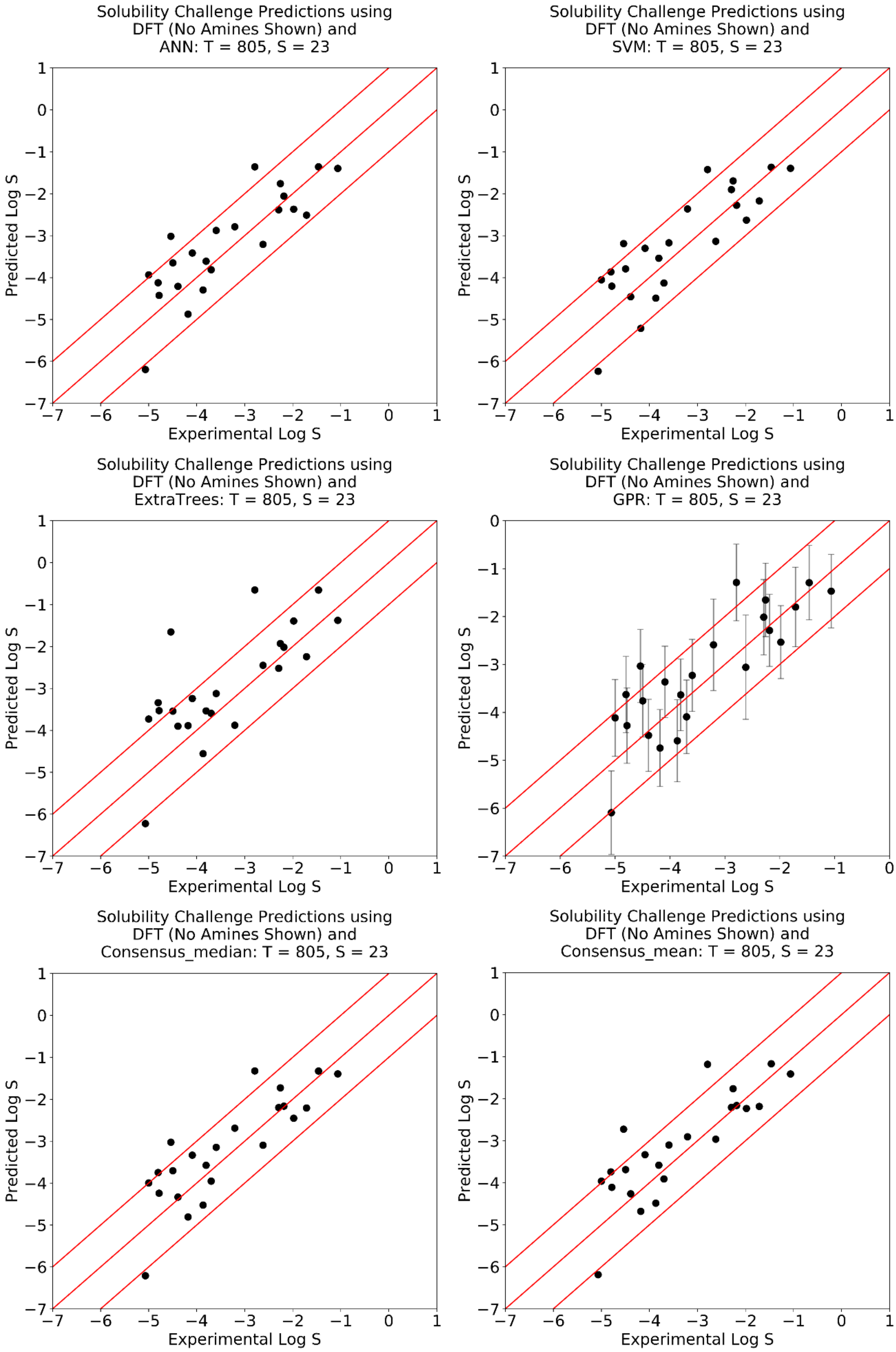

Figure 78. ***SC test*** predictions, amines omitted

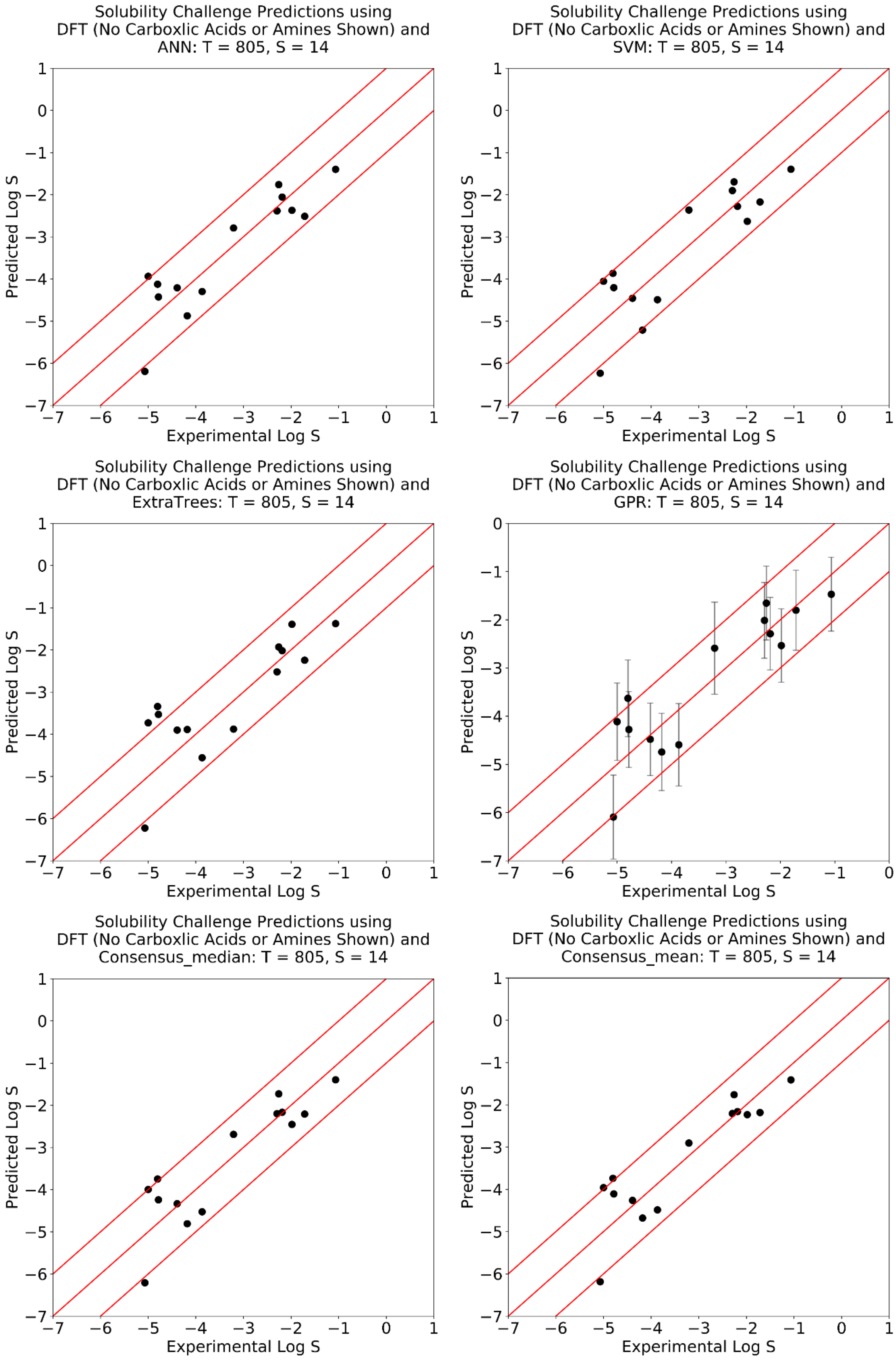

Figure 79. ***SC test*** predictions, carboxylic acids and amines omitted

6.2 AstraZeneca datasets

6.2.1 Dataset details

Data were collection from AstraZeneca internal databases of APIs and pharmaceutical intermediates. All available ethanol and acetone were retained, forming ***Ethanol_AZ*** and ***Acetone_AZ*** external test sets. A selection of data in the range -4 to -2 LogS was obtained for water, forming ***Water_AZ*** external test set. Melting point was obtained from AstraZeneca for 16 out of 26 ethanol data points and 12 out of 19 acetone data points. As water predictions did not rely strongly on melting point, no attempt was made to find melting point data for water data. Descriptors for AZ datasets were calculated using Gaussian 16,[27] due to the unavailability of Gaussian 09 at AstraZeneca.

Table 31. Summary of datasets from AstraZeneca

Name	Solvent	Number of data points	Data points with m.p.
Water_AZ	Water	191	0
Ethanol_AZ	Ethanol	26	16
Acetone_AZ	Acetone	19	12

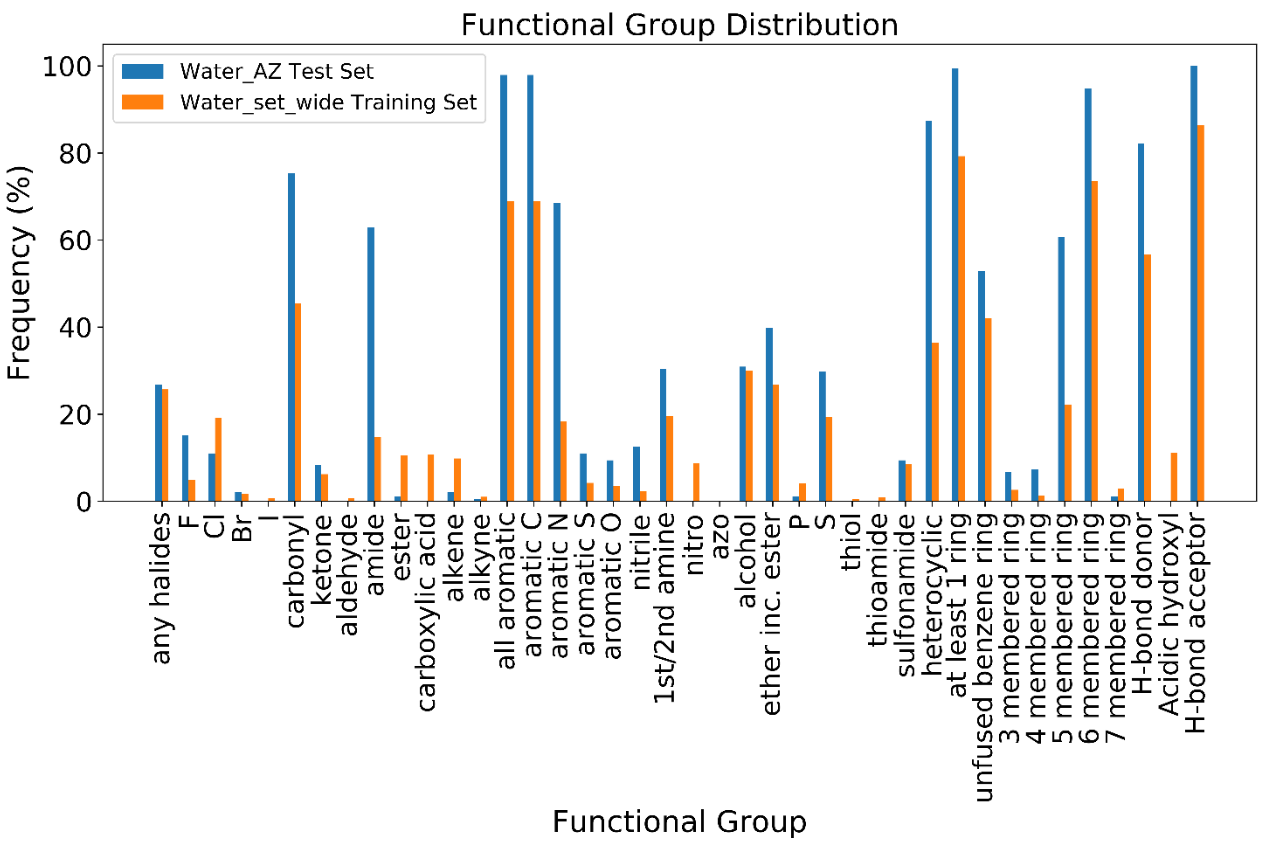

Figure 80. Functional group analysis for the ***Water_AZ*** external dataset compared to ***Water_set_wide*** training set

6.2.2 Predictions with our models

Models were built with and without melting point and with DFT, PM6 and DFT/PM6 with HF energy descriptors. Models in ethanol and acetone without melting point were also run with the same molecules as did have melting point to facilitate comparison. This is shown in **Table 32** and **Figure 81** to **Figure 83**.

Table 32. Summary of metrics for AstraZeneca external test sets using ET

Dataset	Descriptors	m.p.?	Test Size	R^2	RMSE	%LogS±0.7	%LogS±1.0
Water_AZ	DFT	N	191	0.28	0.83	57.6	74.3
Water_AZ	DFT (HF Energies)	N	191	0.30	0.85	53.9	72.8
Water_AZ	PM6	N	191	0.26	0.81	60.2	74.9

Water_AZ	PM6 (HF Energies)	N	191	0.30	0.80	61.8	77.5
Ethanol_AZ	DFT	N	26	0.26	0.62	65.4	88.5
Ethanol_AZ	DFT	N	16	0.28	0.62	68.8	87.5
Ethanol_AZ	DFT	Y	16	0.51	0.51	81.3	100.0
Ethanol_AZ	PM6	N	26	0.28	0.60	69.2	92.3
Ethanol_AZ	PM6	N	16	0.30	0.59	75.0	93.8
Ethanol_AZ	PM6	Y	16	0.21	0.55	75.0	100.0
Acetone_AZ	DFT	N	19	0.28	0.45	89.5	94.7
Acetone_AZ	DFT	N	12	0.57	0.30	100.0	100.0
Acetone_AZ	DFT	Y	12	0.21	0.55	75.0	100.0
Acetone_AZ	PM6	N	19	0.10	0.57	78.9	94.7
Acetone_AZ	PM6	N	12	0.46	0.38	100.0	100.0
Acetone_AZ	PM6	Y	12	0.17	0.64	75.0	83.3

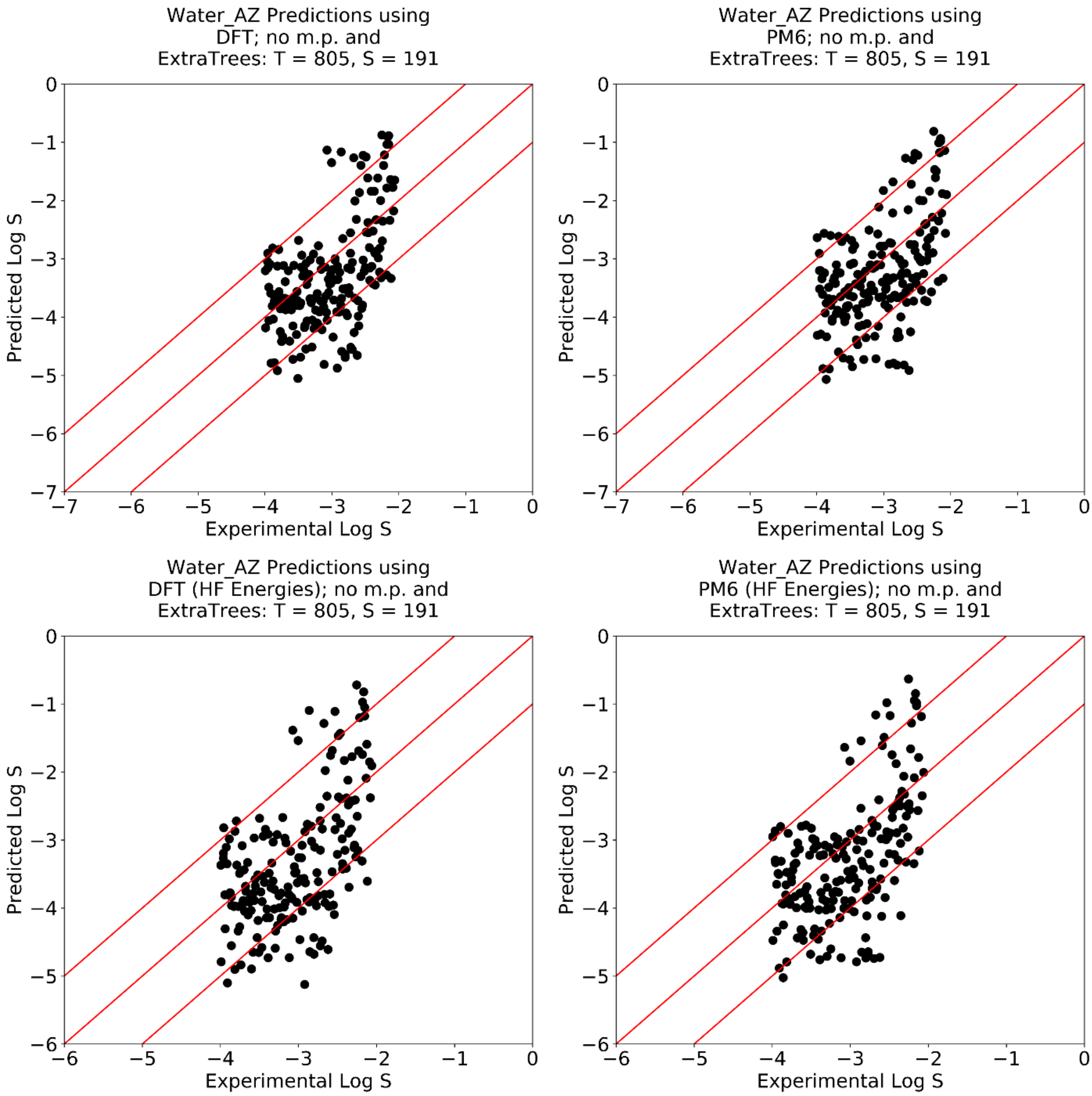

Figure 81. Plots for predictions on ***Water_AZ*** external test set using ET

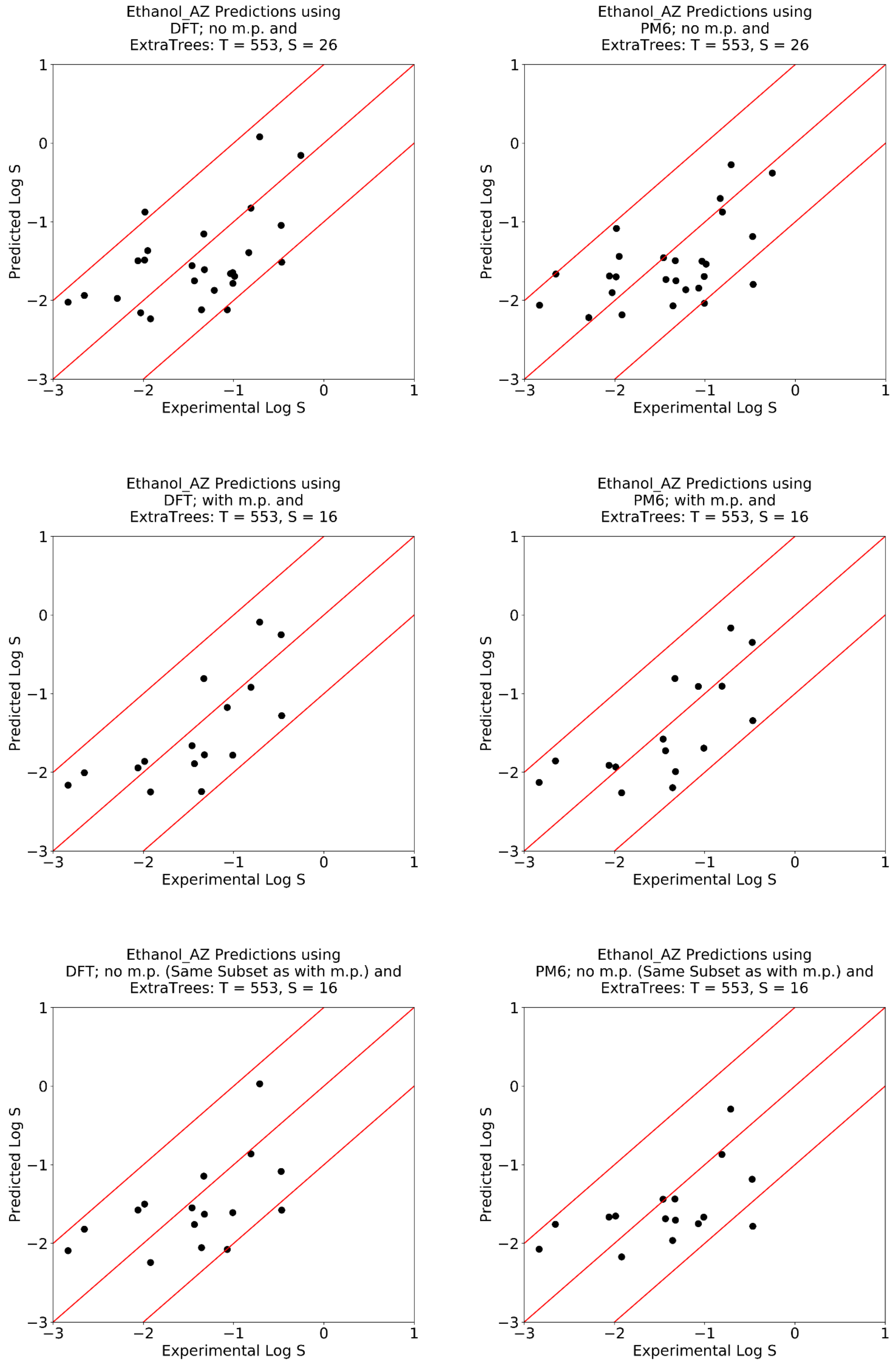

Figure 82. Plots for predictions on ***Ethanol_AZ*** external test set using ET

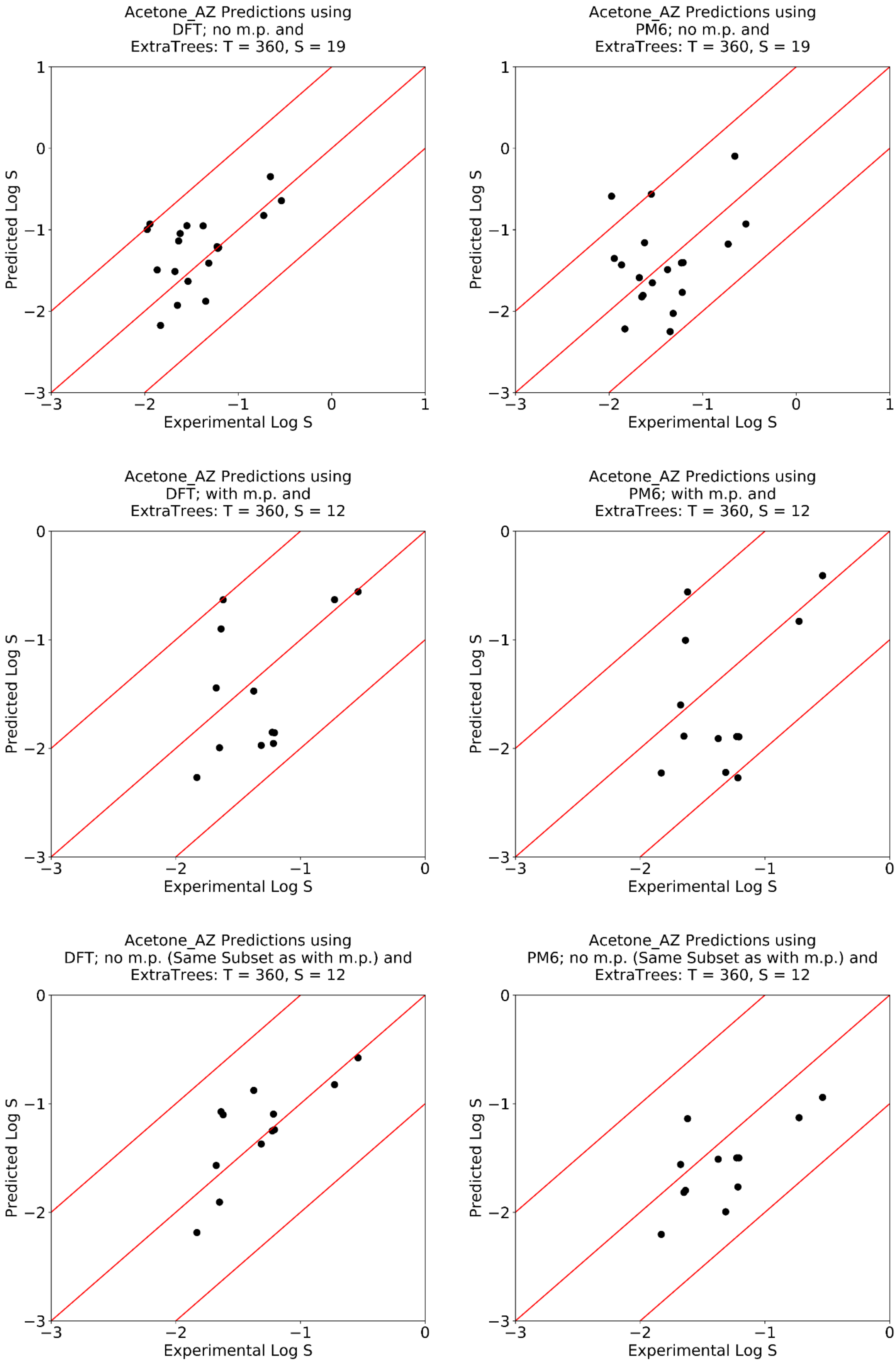

Figure 83. Plots for predictions on ***Acetone_AZ*** external test set using ET

6.2.3 Comparison to COSMOtherm

COSMOtherm predictions, using the same procedure outlines in section 5.2, were run on the same AstraZeneca test sets. For ***Water_AZ***, three molecules did not return a numerical prediction, and were hence excluded from these metrics and plots. Similarly for ***Acetone_AZ***, one molecule was excluded. This is shown in **Table 33** and **Figure 84** to **Figure 86**.

Table 33. Comparison of predictions for AstraZeneca datasets, ET and COSMOtherm

Dataset	Descriptors	m.p.?	Test Size	R^2	RMSE	%LogS±0.7	%LogS±1.0
Water_AZ	DFT	N	191	0.28	0.83	57.6	74.3
Water_AZ	PM6	N	191	0.26	0.81	60.2	74.9
Water_AZ	COSMO	N	188	0.44	1.23	45.7	59.0
Ethanol_AZ	DFT	N	26	0.26	0.62	65.4	88.5
Ethanol_AZ	PM6	N	26	0.28	0.60	69.2	92.3
Ethanol_AZ	COSMO	N	26	0.19	0.85	61.5	84.6
Acetone_AZ	DFT	N	19	0.28	0.45	89.5	94.7
Acetone_AZ	PM6	N	19	0.10	0.57	78.9	94.7
Acetone_AZ	COSMO	N	18	0.24	0.86	55.6	77.8

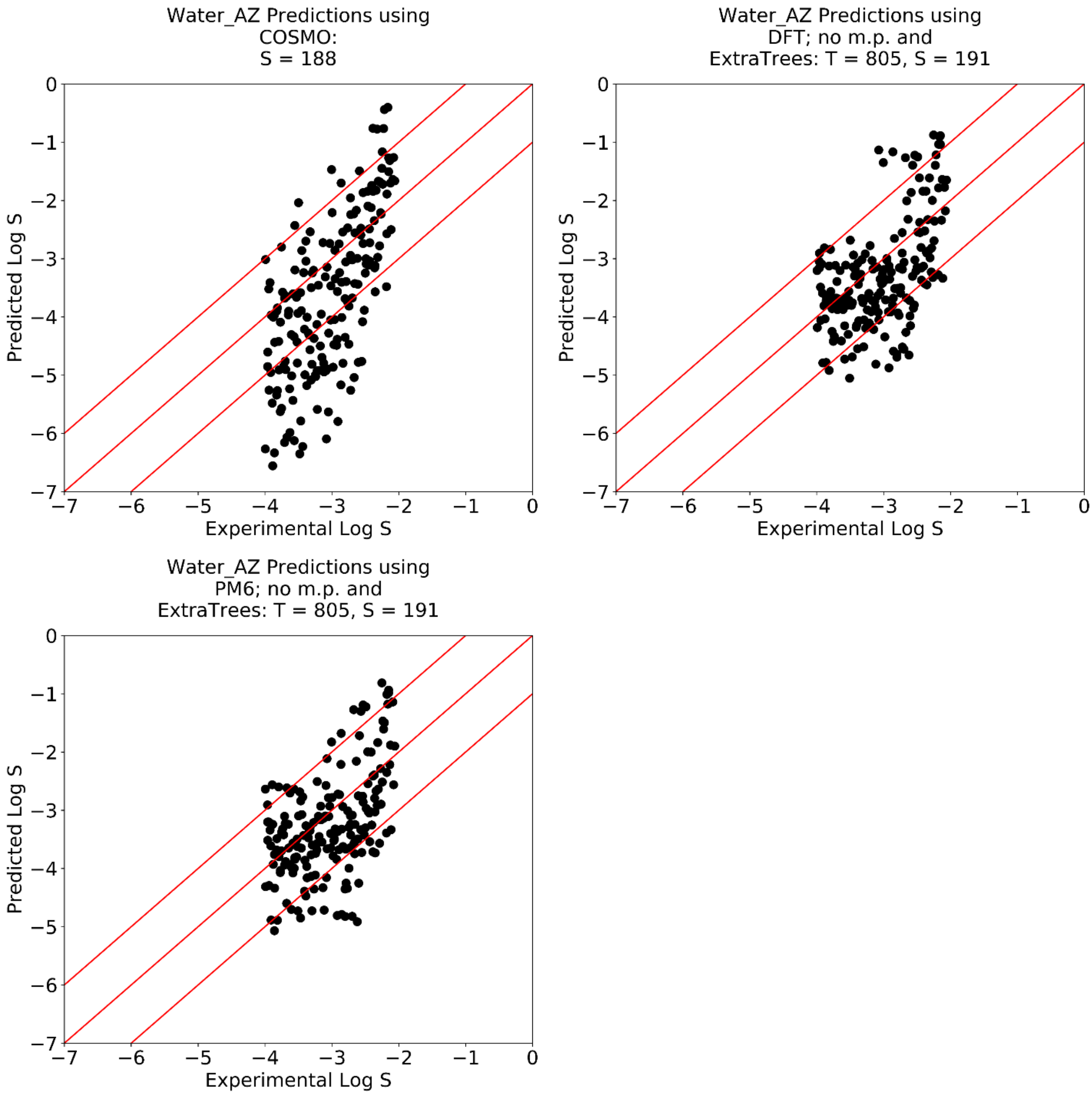

Figure 84. Plots of predictions using COSMOtherm and ET for ***Water_AZ*** external test set

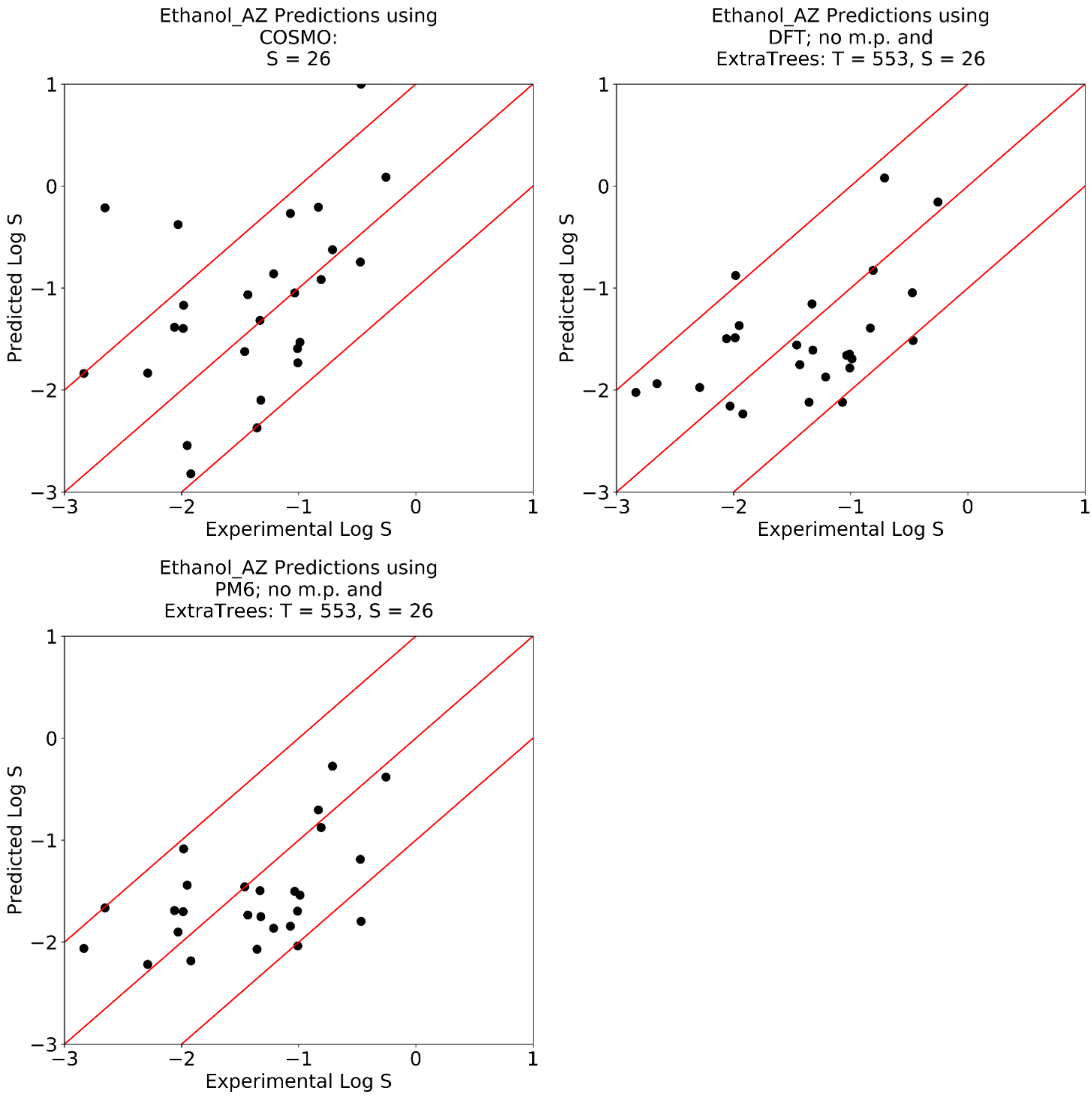

Figure 85. Plots of predictions using COSMOtherm and ET for ***Ethanol_AZ*** external test set

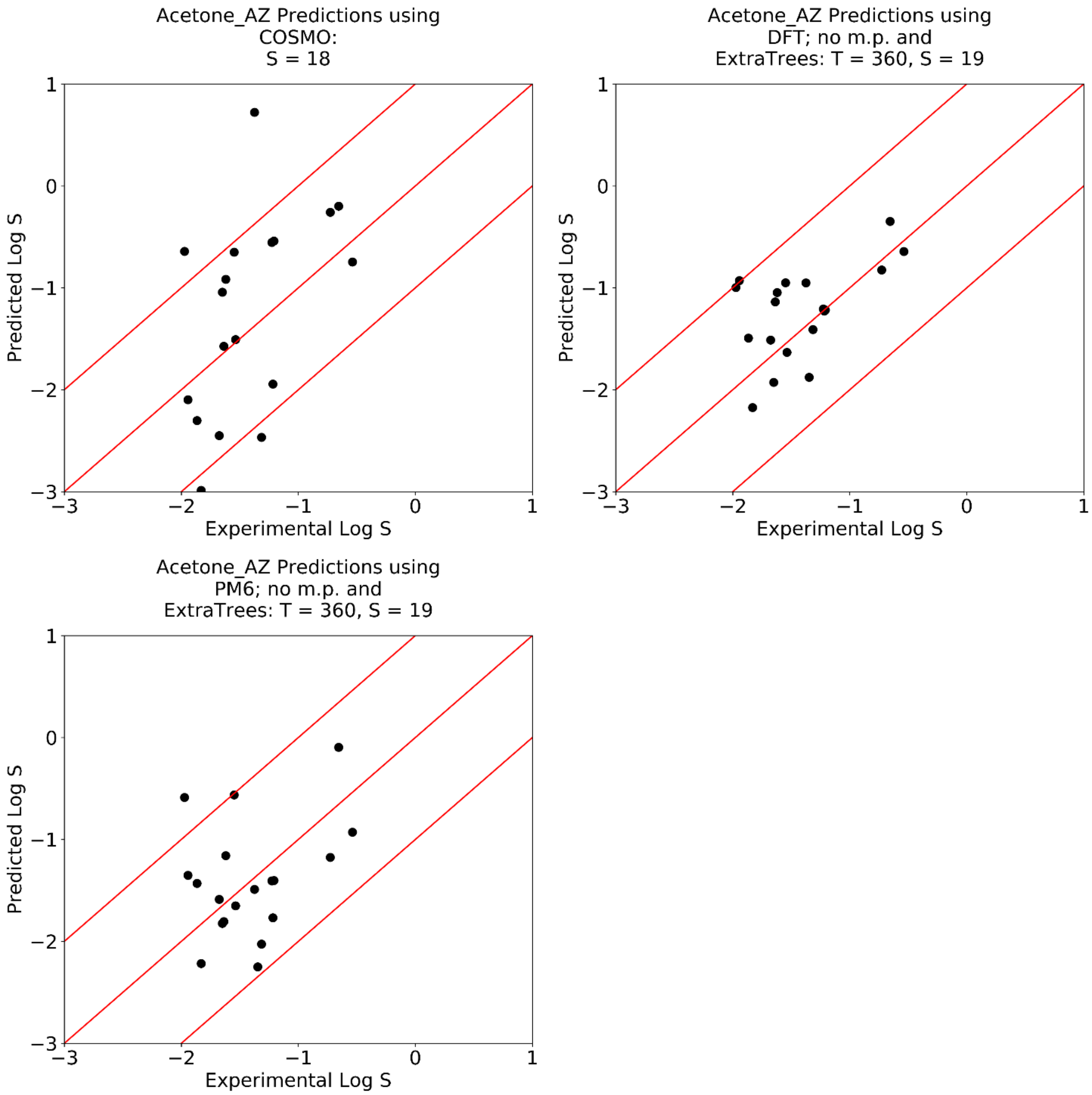

Figure 86. Plots of predictions using COSMOtherm and ET for ***Acetone_AZ*** external test set

Supplementary Note 7: Example Python codes

7.1 Filtering out molecules with disallowed elements and mixtures

```
import re,periodictable

import pandas as pd

from pybel import *

import numpy as np

#create a list of elements to keep AND elements not supported by openbabel
(cannot remove them anyway)

els=["H","C","N","O","F","P","S","Cl","Br","I","Rf","Db","Sg","Bh","Hs","Mt
","Ds","Rg","Cn","Nh","Fl","Mc","Lv","Ts","Og"]

#get the inverse of this list, elements to discard

elements=[]

for el in periodictable.elements:

    elements.append(el.symbol)

del(elements[0])

for f in els:

    elements.remove(f)

#convert list of elements to list of SMARTS codes

elements2=[]

for f in elements:

    elements2.append("[" + f + "]")

data=pd.read_csv("full_dataset.csv")

columns=list(data)

data=np.array(data)

#only keep data where SMILES does not contain a dot (mixture)

keep_data=[]

for f in range(len(data)):

    if re.findall("\.",data[f][4])==[]:##points to column containing SMILES
```

```
        keep_data.append(data[f])
#create a list of data to discard (contains one or more of the disallowed elements)
discard=[]
for f in range(len(keep_data)):
    mol=readstring("smi",keep_data[f][4])
    #match all SMARTS to smile
    for g in elements2:
        smarts=Smarts(g)
        if smarts.findall(mol) != []:
            discard.append(keep_data[f])
            continue
#get a full list of SMILES and a discard list
data_new=[]
smi1=[]
smi2=[]
for f in keep_data:
    smi1.append(f[4])
for f in discard:
    smi2.append(f[4])
#filter out discard molecules
smi_unique=[]
for f in smi1:
    if f in smi2:
        continue
    smi_unique.append(f)
for f in smi_unique:
    for g in range(len(keep_data)):
```

```
        if keep_data[g][4] == f:

            data_new.append(keep_data[g])

#make new dataframe and save

df=pd.DataFrame(data=data_new,columns=columns)

df.to_csv("trimmed_dataset.csv",index=False)
```

7.2 Melting point data from ChemSpider

```
#n.b. this code was written for Python 2.7 for compatibility and the
chemspider API has been subsequently replaced

import csv,re

import urllib2

from chemspipy import ChemSpider

cs = ChemSpider('CS-ID-KEY')

#obtained from ChemSpider on registration

#open initial file

with open('data.csv', 'rb') as csvfile:

        reader=csv.reader(csvfile, delimiter=',')

        row = reader.next()

        data = []

        for row in reader:

            data.append(row)

#loop through each molecule

for i in range(len(data)):

    #default "not found"

    m = "not found"

    stdinchikey=data[i][3]#the column that contains StdInChIKey

    #obtain CSID

    for result in cs.search(stdinchikey):

        m = result

    m=str(m)

    m=m.replace("Compound(","")

    m=m.replace(")","")

    #see if molecule is in ChemSpider

    try:
```

```
        response = urllib2.urlopen('http://www.chemspider.com/Chemical-
Structure.' + m + '.html')
    #if not write this to file and move on to next molecule
    except:
        newcsvrow = []
        newcsvrow.append(stdinchikey)
        newcsvrow.append("NOT IN CS")
        with open(r'MP.csv', 'a') as f: #output file
            writer = csv.writer(f)
            writer.writerow(newcsvrow)
        continue
    html = response.read()
    m = re.search('Experimental Melting Point(.*?)</table>', html, re.S)
    #see if any melting point data is held by ChemSpider
    try:
        n = re.findall('op\">(.*?)\\xc2\\xb0C', m.group(0), re.S)
    #if not write this to file and move on to next molecule
    except AttributeError:
        newcsvrow = []
        newcsvrow.append(stdinchikey)
        newcsvrow.append("NO MP DATA")
        with open(r'MP.csv', 'a') as f: #output file
            writer = csv.writer(f)
            writer.writerow(newcsvrow)
        continue
    #series of operation to get m.p. data
    n = [x.strip(' ') for x in n]
    x2 = []
```

```
for  i in range(len(n)):

    if re.match("-[0-9\.]+-[0-9\.]+", n[i]) is not None:

        y  = re.split("-", n[i])

        del y[0]

        y[0]= "-" + y[0]

        y[0] = float(y[0])

        y[1] = float(y[1])

        z = (y[0] + y[1])/2

        x2.append(z)

    elif re.match("-[0-9\.]+--[0-9\.]+", n[i]) is not None:

        y = re.split("(-\d+)-", n[i])

        del y[0]

        y[0] = float(y[0])

        y[1] = float(y[1])

        z = (y[0] + y[1])/2

        x2.append(z)

    elif re.match("[0-9\.]+-[0-9\.]+", n[i]) is not None:

        y = re.split("-", n[i])

        y[0] = float(y[0])

        y[1] = float(y[1])

        z = (y[0] + y[1])/2

        x2.append(z)

    else:

        x2.append(n[i])

#write this melting point data to file

for i in range(len(x2)):

    MP = x2[i]

    newcsvrow = []
```

```
newcsvrow.append(stdinchikey)

newcsvrow.append(MP)

with open(r'MP.csv', 'a') as f: #output file

        writer = csv.writer(f)

        writer.writerow(newcsvrow)
```

7.3 Obtain Molecular Weight using CIRpy

```
import cirpy

import pandas as pd

#load data

data1=pd.read_csv("data.csv")

#get list of SMILES

smi=data1["Smiles"]

mw_list=[]

#find MW

for i in smi:

    mw_list.append(cirpy.resolve(i, 'mw'))

#add column

data1["MW"]=mw_list

#save data

data1.to_csv("data",index=False)
```

7.4 Functional group analysis using OpenBabel

```
import openbabel

import csv,sys

from pybel import *

import pandas as pd

import numpy as np

#get SMARTS and Dataset

#file with column called “FG” with list of FGs to analyse, and column
called “SMARTS” of SMARTS codes

SMARTS1=pd.read_csv("SMARTS.csv")

water1=pd.read_csv("water.csv")#load dataset

Output=" FG.csv"#outputfile

#Functional Group names

FG_names=SMARTS1['FG']

#Get SMART CODES

SMARTS_codes=SMARTS1['SMARTS']

#Get SMILES

smiles=water1['Smiles']

water1=np.array(water1)

FG_list=[]

#for each molecule

for f in range(len(water1)):

    FG=[]

    #Get StdInChIKey identifier

    FG.append(water1[f][0])

    #Create mol object from smiles

    mol=readstring("smi",smiles[f])

    #match all SMARTS to SMILES
```

```
    for g in SMARTS_codes:
        smarts=Smarts(g)
        if smarts.findall(mol) == []:
            FG.append("0")
        else:
            FG.append("1")
    FG_list.append(FG)
FG_names2=list(FG_names)
FG_names2.insert(0,"StdInChIKey")
FG_list=pd.DataFrame(data=FG_list,columns=FG_names2)
#this can then be outputted/merged or graphed
FG_list.to_csv(Output,index=False)
```

7.5 Generating input structures with molecular mechanics and CIRpy

```
import cirpy

import csv,os

import pandas as pd

#parameters to change

input_file="water.csv" #input file

output_file_dir=" Input Files3" #where to put input files

commands="#PM6 opt freq" #Gaussian 09 commands to include

time="1:00:00" #time limit for calculation

data=pd.read_csv(input_file)#read input file

os.mkdir(output_file_dir)#make output folder

smi2=data["Smiles"] #column containing SMILES

std=data["StdInChIKey"] #column containing StdInChIKey

for j in range(len(smi2)): #for each molecule

    name = std[j]

    smi = smi2[j]

    try: #try to get structure

        m = cirpy.resolve(smi,'xyz')

    except:

        print(name) #print if not

        continue

    try: #make sure structure has been found

        m=m.splitlines()

    except AttributeError:

        print(name) #print if not

        continue

    del(m[0])

    del(m[0])
```

```
#open a .com Gaussian 09 input file and add details
f = open (output_file_dir + "\\" + name + ".com","a")
f.write("%nprocshared=4\n")
f.write("%mem=100MW\n")
f.write("%chk=" + name + ".chk\n")
f.write(commands + "\n")
f.write("\ngas opt\n\n")
f.write("0 1\n")
for i in range(len(m)):
    f.write(m[i])
    f.write('\n')
f.write('\n')
f.write('\n')
f.close()
#open a .sh bash file for command line submission
f = open (output_file_dir + "\\" + name + ".sh","a")
f.write("#$ -cwd -V\n")
f.write("#$ -l h_vmem=4G\n")
f.write("#$ -l h_rt=" + time + "\n")
f.write("#$ -pe smp 4\n")
f.write("#$ -m be\n")
f.write("module add gaussian\n")
f.write("g09 " + name + ".com")
f.close()
```

7.6 Calculating SASA using pymol

```
#this code works with pymol and Python installed on anaconda

import __main__

#load up pymol quiet with no GUI

__main__.pymol_argv = ['pymol','-qc']

import pymol,sys,os,re

pymol.finish_launching()

#load folder containing folder “xyz” of .xyz files

path="Sol"

#set solvent radius to 1.4

solvent_radius="1.4"

pymol.cmd.reinitialize()

pymol.cmd.set('dot_solvent', 1)

pymol.cmd.set('dot_density', 4)

pymol.cmd.set('solvent_radius', float(solvent_radius))

#make folder in same folder as xyz folder

os.makedirs(path + '\\SASA')

#write a file containing SASA for each molecule

for xyzfile in os.listdir(path + '\\xyz'):

    file_name = xyzfile[:-4]

    pymol.cmd.load (path + '\\xyz\\' + file_name + '.xyz')

    ligand_area=pymol.cmd.get_area(file_name)

    file2= open(path + "\\SASA\\" + file_name + ".sasa", "w")

    file2.write(str(ligand_area))

    file2.close()

    pymol.cmd.delete(file_name)
```

7.7 Creating shadow projections descriptors

7.7.1 Creating shadow projection images

The optimised structured provided the Cartesian coordinates for the centre of atomic interlocking spheres with the Van der Waal radii shown in **Table 34**.

Table 34. Van der Waal radii of spheres created

**Atom**	**Radius**
H	1.2
B	2
C	1.7
N	1.55
O	1.52
F	1.47
P	1.8
S	1.8
Cl	1.75
Br	1.85
I	1.98

Random points were chosen on this molecular surface and the point size increased to give solid black images. These images were viewed from three perpendicular angles and saved on the same 1557 x 1547 pixels canvas, as seen in **Figure 87**.

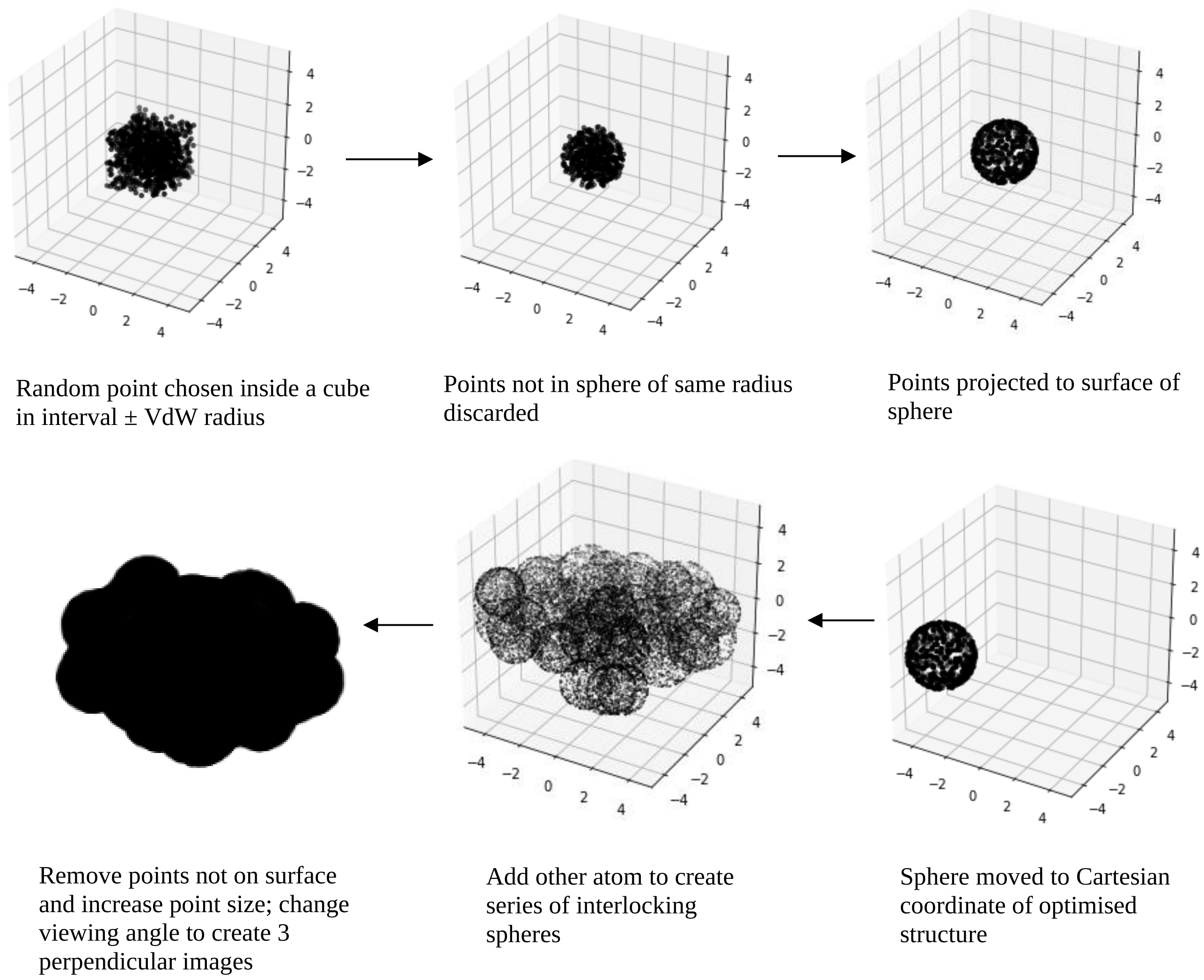

Figure 87. Procedure to create a shadow projection from atomic Cartesian coordinates and Van der Waal radii

```
import math,re,os

import numpy as np

import matplotlib.pyplot as plt

import pandas as pd

from mpl_toolkits.mplot3d import Axes3D

#number of points per atom

number_points=1000

#vdw radii

radiiDict={'H':1.2,'B':2,'C':1.7,'N':1.55,'O':1.52,'F':1.47,'P':1.8,'S':1.8
,'Cl':1.75,'Br':1.85,'I':1.98}
```

```python
def get_points(xyz):

    #open .xyz file

    f = open(xyz,"r")

    data = []

    #get contents

    for line in f:

        data.append(line)

    #delete first 2 lines which do not contain Cartesians

    del(data[0])

    del(data[0])

    #make new lists to populate

    atom_pos=[]

    atom_names=[]

    atom_radii=[]

    #for each line (atomic XYZ coordinates)

    for f in range(len(data)):

        #remove line space

        data[f]=data[f].replace('\n','')

        #split into atom,x,y,z

        data[f]=re.split(r'\s+',data[f])

        #get atom name

        atom_names.append(data[f][0])

        #delete atom name

        del(data[f][0])

    #atom position is remaining x,y,z

    atom_pos=data

    #convert coordinates to floats

    atom_pos = [[float(j) for j in i] for i in atom_pos]
```

```
    #get the corresponding radius for each atom

    for f in range(len(atom_names)):

        atom_radii.append(radiiDict[atom_names[f]])

    #define new list

    data=[]

    #for each atom

    for f in range(len(atom_names)):

        #Produce random points in a cube

        x=((2*atom_radii[f])*np.random.rand(number_points,3))-atom_radii[f]

        #Keep points inside the sphere

        keep=[]

        for point in x:

            if math.sqrt(((point[0])**2)+((point[1])**2)+((point[2])**2)) <
atom_radii[f]:

                keep.append(point)

        keep=np.array(keep)

        #Project points to surface of sphere

        x1=[]

        y1=[]

        z1=[]

        for point in keep:

            d=math.sqrt(((point[0])**2)+((point[1])**2)+((point[2])**2))

            scale=(atom_radii[f]-d)/d

            point=point+(scale*point)

            x1.append(point[0])

            y1.append(point[1])

            z1.append(point[2])

        #Move atom to correct position
```

```
        for i in range(len(x1)):
            x1[i]=x1[i]+atom_pos[f][0]
        for i in range(len(y1)):
            y1[i]=y1[i]+atom_pos[f][1]
        for i in range(len(z1)):
            z1[i]=z1[i]+atom_pos[f][2]
        data.append(x1)
        data.append(y1)
        data.append(z1)
    #Discard points in shape interior
    for f in range(len(atom_names)):
        for g in range(len(atom_names)):
            if g==f:
                continue
            keep=[]
            for i in range(len(data[3*f])):
                if math.sqrt(((data[3*f][i]-
atom_pos[g][0])**2)+((data[(3*f)+1][i]-
atom_pos[g][1])**2)+((data[(3*f)+2][i]-atom_pos[g][2])**2)) >
atom_radii[g]:
                    keep.append(i)
            x1_keep=[]
            y1_keep=[]
            z1_keep=[]
            for x in keep:
                x1_keep.append(data[3*f][x])
                y1_keep.append(data[(3*f)+1][x])
                z1_keep.append(data[(3*f)+2][x])
            data[(3*f)]=x1_keep
```

```
            data[(3*f)+1]=y1_keep

            data[(3*f)+2]=z1_keep

    x=[]

    y=[]

    z=[]

    #merge points

    for f in range(len(data)):

        if f%3 == 0:

            for g in data[f]:

                x.append(g)

        if f%3 == 1:

            for g in data[f]:

                y.append(g)

        if f%3 == 2:

            for g in data[f]:

                z.append(g)

    #return separate x, y and z point lists

    return(x,y,z)

#plot and save graphs

def graph(x,y,z,az,el):

    fig = plt.figure(figsize=(20,20)) #large canvas size for resolution and
to fit larger molecules

    #use 3d plotting

    ax = fig.add_subplot(111,projection='3d')

    #colour black with big point size so image opaque

    ax.scatter(x,y,z,color="black",s=100)

    ax.set_xlim(-20,20)

    ax.set_ylim(-20,20)
```

```
    ax.set_zlim(-20,20)

    #no axes!

    plt.axis('off')

    #axim and alev are the angles to define the view, change to get
projection down each axis

    ax.view_init(azim=az, elev=el)

#save x, y, z surface points

dir1=" xyz"

dir2="vdw_points"

os.makedirs(dir2)

for files in os.listdir(dir1):

    x,y,z = get_points(dir1 + "\\" + files)

    files=files.replace(".xyz","")

    f = open(dir2 + "\\" + files + ".csv","a")

    for i in range(len(x)):

        f.write(str(x[i]) + "," + str(y[i]) + "," + str(z[i]) + "\n")

    f.close()

#reload surface points file and save images

#new directory to place images

dir3="F:\\Data\\reaxys_acetone\\DFT\\Sol\\shadow_image_vdw"

os.makedirs(dir3)

for files in os.listdir(dir2):

    data=np.loadtxt(dir2 + "\\" + files,delimiter=",")

    files=files.replace(".csv","")

    x=data[:,0]

    y=data[:,1]

    z=data[:,2]

    graph(x,y,z,0,0)
```

```
plt.savefig(dir3 + "\\" + files + "1.png") #first angle

plt.close()

graph(x,y,z,90,0)

plt.savefig(dir3 + "\\" + files + "2.png") #perpendicular

plt.close()

graph(x,y,z,90,90)

plt.savefig(dir3 + "\\" + files + "3.png") #perpendicular again

plt.close()
```

7.7.2 Image recognition to get descriptors

An area and aspect ratio was calculated for each image (three per molecule, creating six descriptors per molecule). This was performed with OpenCV. The outline of the image was found and the number of pixels calculated to give area. A minimum area rectangle was drawn around the image to give aspect ratio (length/width to give Aspect Ratio < 0).

```
import cv2 as cv

import os

import pandas as pd

def shadow_info(image):

    #initiate list for results

    results=[]

    #load image

    img = cv.imread(image,0)

    #fit shape

    ret,thresh = cv.threshold(img,127,255,0)

    #get shape by increasing area

    contours,hierarchy=cv.findContours(thresh, cv.RETR_LIST,
cv.CHAIN_APPROX_NONE)

    #get second last (last is full frame)

    cnt=contours[-2]

    #get area

    area=cv.contourArea(cnt)

    results.append(area)

    #fit minimum area rectangle

    rect = cv.minAreaRect(cnt)

    #width,height,area and aspect ratio

    width = float(rect[1][0])

    length = float(rect[1][1])

    rect_area=width*length
```

```
    aspect_ratio = width/length
    #do not know whether width or height is larger
    if aspect_ratio>1:
        aspect_ratio = length/width
    results.append(aspect_ratio)
    return(results)
#get for every molecule
dir2="shadow_image_vdw"
dir3="shadow_desc.csv "
data=[]
for files in os.listdir(dir2):
    row=[]
    files=files.replace(".xyz","")
    results1=shadow_info(dir2 + "\\" + files + "1.png")
    results2=shadow_info(dir2 + "\\" + files + "2.png")
    results3=shadow_info(dir2 + "\\" + files + "3.png")
    areas=sorted([results1[0],results2[0],results3[0]]) #get ascending
areas
    asp_ratios=sorted([results1[1],results2[1],results3[1]]) #get ascending
aspect ratios
    row.append(files) #get names
    for ar in areas: #add areas
        row.append(ar)
    for asp in asp_ratios: #add aspect ratios
        row.append(asp)
    data.append(row)
df=pd.DataFrame(data=data,columns=["StdInChIKey","Area1","Area2","Area3","A
sp1","Asp2","Asp3"])
df.to_csv(dir3,index=False)
```

7.8 Charge surface descriptors

7.8.1 Generate isosurface points and charges

The electron density from the initial DFT calculation in Gaussian 09 was used to create an isosurface encompassing 95 % of the electron density using Multiwfn programme. This is transferred back to Gaussian 09 to get a .cube file (a standard electrostatic potential file format). This is returned to Multiwfn, where the charge at these input points is extracted.

Procedure summary:

1. Produce .fchk file for each molecule
2. Produce "wfn_pt.txt" for each molecule to use an input for multiwfn
3. Run wfn_pt.txt in multiwfn for each .fchk file to get cubegenpt.txt (this contains the points at which Gaussian 09 will compute ESP)
4. Get cube file using Gaussian 09
5. Produce "wfn_input2.txt" for each molecule to use as an input file for multiwfn
6. Run wfn_input2.txt for each .fchk file to get vtx.txt (this contains the x,y,z of each point on the isosurface and the ESP associated with it)
7. Format to get .csv file with X, Y, Z, ESP

Codes:

1. .fchk files

```
# bash script which requires Gaussian 09 and its associated programmes installed

# .fchk files produced in Gaussian 09: (how to do using UoL ARC2 HPC)

# ```

# module add gaussian

# for f in *.chk; do formchk "$f"; done

# ```

# Organise each .fchk files to be in a folder of the same name
```

2. Produce "wfn_pt.txt" for each molecule to use an input for multiwfn

```
# python script

# for each folder containing .fchk file, make a file called "wfn_pt.txt"

import os
```

```
dir1="F:\\Data\\reaxys_acetone\\DFT\\Sol\\wfn"
for folders in os.listdir(dir1):
    f = open(dir1+ "\\" + folders + "\\wfn_pt.txt","a")
    f.write("12\n")
    f.write("5\n")
    f.write("2\n")
    f.write("0\n")
    f.write("M:\\books.csv") #spike with bad file to terminate program
    f.close()
```

3. Run wfn_pt.txt in multiwfn for each .fchk file to get cubegenpt.txt

```
#
# wfn_pt.txt has the commands to run inside multiwfn. Use a simple loop
in terminal in get output:
# bash script using Multiwfn command line feature
# ```
# for f in *;
# do cd "$f";
# ./Multiwfn.exe "$f".fchk < wfn_pt.txt > null;
# cd ..;
# done
# ```
```

4. Use Gaussian 09 to get .cube file

```
# bash script which requires Gaussian 09 and its associated programmes
installed
# ```
# for f in *;
# do cd "$f";
```

```
# cubegen 0 potential "$f".fchk pot.cube -5 h < cubegenpt.txt;

# cd ..;

# done

# ```
```

5. Produce "wfn_input2.txt" for each molecule to use as an input file for multiwfn

```python
#python script

#for each folder containing .fchk file, make a file called
"wfn_input2.txt"

import os

dir1="F:\\Data\\reaxys_acetone\\DFT\\Sol\\wfn_ace"

for folders in os.listdir(dir1):

    f = open(dir1+ "\\" + folders + "\\wfn_input2.txt","a")

    f.write("12\n")

    f.write("5\n")

    f.write("2\n")

    f.write("0\n")

    f.write(dir1 + "\\" + folders + "\pot.cube\n")

    f.write("7")

    f.close()
```

6. Run wfn_input2.txt for each .fchk file to get vtx.txt (this contains the x,y,z of each point on the isosurface and the ESP associated with it)

```
# bash script using Multiwfn command line feature

# ```

# for f in *;

# do cd "$f";

# ./Multiwfn.exe "$f".fchk < wfn_input2.txt > null;

# cd ..;
```

```
# done

# ```
```

7. **Format to get .csv file with X, Y, Z, ESP**

```
#python script to reformat Multiwfn output into .csv files

import csv,os,sys

import pandas as pd

dir1="wfn_ace" #folder of folders for each molecule containing raw data

dir2="wfn_esp" #folder to put output

os.makedirs(dir2)

for folders in os.listdir(dir1):

    f = open(dir1  + "\\" + folders + "\\vtx.txt","r")

    data=[]

    for line in f:

        data.append(line)

    for i in range(len(data)):

        data[i]=data[i].split()

    del(data[0])

    data2=[]

    for f in range(len(data)):

        data3=[]

        data3.append(data[f][0])

        data3.append(data[f][1])

        data3.append(data[f][2])

        data3.append(data[f][3])

        data2.append(data3)

    names=['X','Y','Z','ESP']

    df=pd.DataFrame(data=data2,columns=names)
```

```
        df.to_csv(dir2 + "\\" + folders + ".csv",index=False)
```

7.8.2 Clustering to find charged regions on isosurfaces

Clustering techniques to find region of spatially close points in 3D space is common especially in visualising classification problems. Here it is used to find regions of charge on the isosurface. Points outside -0.02 and 0.02 are discarded and the resulting regions are charge analysed. Each region has an area (number of points, as points are assumed to be uniformly spread across isosurface), mean charge and standard deviation of the charge (indicating the polarisation of the charged region). Scipy Python module was used for clustering, all parameters were varied to give the highest variation in final descriptors obtained.

```
#step 1: produce .csv file detailing area, mean charge and charge standard deviation for each region

import os

import pandas as pd

import numpy as np

import scipy.cluster.hierarchy as hcluster

dir1="wfn_esp" #input ESP data directory

dir2=" esp_cluster" #output directory

os.makedirs(dir2)

#do for all molecules

for files in os.listdir(dir1):

    data=pd.read_csv(dir1 + "\\" + files)

    files=files.replace(".csv","")

    x=list(data['X'])

    y=list(data['Y'])

    z=list(data['Z'])

    ESP=list(data['ESP'])

    #filter "neutral points"

    #limits

    lim1=-0.02

    lim2=0.02
```

```
    #new lists
    ESP1=[];ESP2=[];x1=[];x2=[];y1=[];y2=[];z1=[];z2=[]
    #ESP1 etc. are the "keep" lists
    for f in range(len(ESP)):
        if ESP[f] > lim2: #if within limits
            ESP1.append(ESP[f])
            x1.append(x[f])
            y1.append(y[f])
            z1.append(z[f])
        elif ESP[f] < lim1:
            ESP1.append(ESP[f])
            x1.append(x[f])
            y1.append(y[f])
            z1.append(z[f])
    #ESP2 etc. are the "discard" lists
    for f in range(len(ESP)):
        if ESP[f] < lim2:
            if ESP[f] > lim1:
                ESP2.append(ESP[f])
                x2.append(x[f])
                y2.append(y[f])
                z2.append(z[f])
    ###if no point left
    if len(x1) == 0:
        charge_data=[[0,0,0,0]] #give zeros for stats

df_charge=pd.DataFrame(data=charge_data,columns=["Number","Area","Mean_char
ge","Std_charge"])
        df_charge.to_csv(dir2 + "\\" + files + ".csv") #save file
```

```
        continue
#get list of coordinates form list of x, y and z
coord=[]
for f in range(len(x1)):
    xyz=[]
    xyz.append(x1[f])
    xyz.append(y1[f])
    xyz.append(z1[f])
    coord.append(xyz)
#define a threshold for finding regions
thresh = 0.8
clusters = hcluster.fclusterdata(coord, thresh, criterion="distance")
#clusters are now labels
clusters=[str(x) for x in clusters]
#add cluster and charge to data
for f in range(len(coord)):
    coord[f].append(clusters[f])
    coord[f].append(ESP1[f])
df=pd.DataFrame(data=coord,columns=['x','y','z','cluster','ESP'])
#group data by cluster
groups = df.groupby('cluster')
#get charge areas, mean and std
charge_data=[]
for name, group in groups:
    charge=group.ESP
    charge_mean=np.mean(charge)
    charge_std=np.std(charge)
    area=len(charge)
```

```
            data=[] #add to new list
            data.append(name)
            data.append(area)
            data.append(charge_mean)
            data.append(charge_std)
            charge_data.append(data)
        #save to file

df_charge=pd.DataFrame(data=charge_data,columns=["Number","Area","Mean_char
ge","Std_charge"])
        df_charge = df_charge.sort_values('Area')
        df_charge.to_csv(dir2 + "\\" + files + ".csv")
#step 2: get descriptors
import os,re
import pandas as pd
import numpy as np
dir1="esp_cluster" #.csv files of cluster stats
data_set=[]
for files in os.listdir(dir1): #for each molecule
    df=pd.read_csv(dir1 + "\\" + files)
    data=[]
    #append StdInChIKey
    files=files.replace('.csv','')
    data.append(files)
    #get number of points per region
    no_regions=df["Area"]
    no_regions_trimmed=[]
    #append no. of regions with more than 20 points
    for f in no_regions:
```

```
    if f > 20:

        no_regions_trimmed.append(f)

data.append(len(no_regions_trimmed))

#append total charge weighted area

points=df["Area"]

charge=df["Mean_charge"]

tot_weighted_charge=0

for f in range(len(points)):

    weighted_charge=points[f]*charge[f]

    tot_weighted_charge=tot_weighted_charge+weighted_charge

data.append(tot_weighted_charge)

#append total neg charge

neg_weighted_charge=0

for f in range(len(points)):

    if charge[f]<0:

        weighted_charge=points[f]*charge[f]

        neg_weighted_charge=neg_weighted_charge+weighted_charge

data.append(neg_weighted_charge)

#append total pos charge

pos_weighted_charge=0

for f in range(len(points)):

    if charge[f]>0:

        weighted_charge=points[f]*charge[f]

        pos_weighted_charge=pos_weighted_charge+weighted_charge

data.append(pos_weighted_charge)

#append information about largest region

big_index=np.argmax(np.array(points))

data.append(points[big_index])
```

```
    data.append(charge[big_index])

    std=df['Std_charge']

    data.append(std[big_index])

    #append to master list

    data_set.append(data)

#create dataframe of descriptors

data_df=pd.DataFrame(data=data_set,columns=["StdInChIKey","No_regions","Tot
_charge","Neg_charge","Pos_charge","Big_area","Big_charge","Big_std"])

data_df.to_csv("espdesc_new.csv",index=False) #save file
```

7.9 Correlation Analysis

This code below produces a figure in the style of **Figure 13**

```
import numpy as np

import matplotlib.pyplot as plt

def corr_graph(Data,title):

    #get correlation table

    corr=Data.corr()

    #convert to R^2

    corr=corr.pow(2)

    fig = plt.figure(figsize=(10,10))

    ax = fig.add_subplot(111)

    cax = ax.matshow(corr,cmap='coolwarm', vmin=0, vmax=1)

    fig.colorbar(cax)

    ticks = np.arange(0,len(Data.columns),1)

    ax.set_xticks(ticks)

    plt.xticks(rotation=90)

    ax.set_yticks(ticks)

    ax.set_xticklabels(Data.columns)

    ax.set_yticklabels(Data.columns)
```

```
    #y parameter move title up to be legible
    ax.set_title("Correlation Table - " + title,y=1.15)
    #Find descriptors with R^2 > 0.9
    corr=Data.corr()
    corr=corr.pow(2)
    #using numpy, get values
    arr = corr.values
    index_names = corr.index
    col_names = corr.columns

    # Get indices where such threshold is crossed; avoid diagonal elements
    R,C = np.where(np.triu(arr,1)>0.9)

    # Arrange those in columns and put out as a dataframe
    out_arr = np.column_stack((index_names[R],col_names[C],arr[R,C]))
    df_corr = pd.DataFrame(out_arr,columns=[['row_name','col_name','R2']])
    return(df_corr)
```

7.10 Evaluation of metrics

Metrics were calculated with the following methods. scipy.stat.pearsonr was used to get Pearson's correlation (R), then the number extracted and converted to R^2.

```
import math,re
from scipy.stats import pearsonr
import numpy as np

#Define statistical measures and R2 conversion
#define RMSE
def rmse(predictions, targets):
```

```
    return np.sqrt(((predictions - targets) ** 2).mean())
#define % within certain range
def within_range(list1, list2, range2):
    x=0
    for i in range(len(list2)):
        if (list1[i]-range2)<= list2[i] <= (list1[i]+range2):
            x+=1
    return((float(x)/(len(list2)))*100)
#define getting R2 method (converts list of scipy.stats.pearsonr outputs to list of R2)
def get_R2(R2):
    R2_2=[]
    for i in range(len(R2)):
        x=re.findall('\d\.\d+',str(R2[i]))
        j=float(x[0])
        j=j**2
        R2_2.append(j)
    return(R2_2)
```

7.11 Descriptor importance in ET

```
#method to get feature importance in ET
from __future__ import print_function
import sys,os,re
import pandas as pd
import numpy as np
from sklearn import preprocessing
from sklearn import ensemble
from scipy.stats import pearsonr
import math
```

```
import statistics

#number of repeats
n_rep=100 ###########

#define RMSE
def rmse(predictions, targets):
    return np.sqrt(((predictions - targets) ** 2).mean())

#define % within certain range
def within_range(list1, list2, range2):
    x=0
    for i in range(len(list2)):
        if (list1[i]-range2)<= list2[i] <= (list1[i]+range2):
            x+=1
    return((float(x)/(len(list2)))*100)

#define getting R2 method
def get_R2(R2):
    R2_2=[]
    for i in range(len(R2)):
        x=re.findall('\d\.\d+',str(R2[i]))
        j=float(x[0])
        j=j**2
        R2_2.append(j)
    return(R2_2)

#Return feature importance for a run
```

```
def stat_split_metrics(train,test):
    #place target value in y
    y_train = train['LogS']
    y_test = test['LogS']
    #place descriptors in X
    X_train = train[['MW','MP','Volume','G_solv','DeltaG_sol','solv_dip',
                     'LsoluHsolv','LsolvHsolu','SASA','O_charges',
                     'C_charges','Most_neg','Most_pos','Het_charges']]
    X_test = test[['MW','MP','Volume','G_solv','DeltaG_sol','solv_dip',
                     'LsoluHsolv','LsolvHsolu','SASA','O_charges',
                     'C_charges','Most_neg','Most_pos','Het_charges']]
    #scale data
    scaler = preprocessing.StandardScaler().fit(X_train)
    X_train = scaler.transform(X_train)
    X_test = scaler.transform(X_test)
    #run models
    #ExtraTrees
    tree3 = ensemble.ExtraTreesRegressor(n_estimators=500,n_jobs=-1)
    tree3.fit(X_train, y_train)
    tree3preds = tree3.predict(X_test)
    #return feature importances
    return(tree3.feature_importances_)

#get mean metrics from these predictions
def get_cons(metrics):
    #Get mean metrics
    lst=[]
    for f in descs:
```

```
        temp=[]
        metrics2=metrics[f]
        metrics2=np.array(metrics2)
        mean=np.mean(metrics2)
        std=np.std(metrics2)
        temp.append(f)
        temp.append(mean)
        temp.append(std)
        lst.append(temp)
    mean_metrics=pd.DataFrame(data=lst,columns=["Descriptor","Mean","Std"])
    return(mean_metrics)

#for dataset
train=pd.read_csv("train.csv")
test=pd.read_csv("test.csv")
master=[]
descs=['MW','MP','Volume','G_solv','DeltaG_sol','solv_dip',
                     'LsoluHsolv','LsolvHsolu','SASA','O_charges',
                     'C_charges','Most_neg','Most_pos','Het_charges']
for g in range(n_rep):
    features=stat_split_metrics(train, test)
    master.append(features)
water1_df=pd.DataFrame(data=master,columns=descs)
water1_df=get_cons(water1_df)
```

Supplementary References

1. ONS Challenge Database. Available at: https://onschallenge.wikispaces.com/. (Accessed: 1st January 2018)

2. Reaxys Database Advanced Search. Available at: https://www.reaxys.com/#/search/advanced. (Accessed: 1st July 2019)

3. Chemspider search. Available at: http://www.chemspider.com. (Accessed: 1st July 2019)

4. CIRpy, Python interface for the Chemical Identifier Resolver (CIR). Available at: http://cactus.nci.nih.gov/chemical/structure. (Accessed: 1st January 2019)

5. Kim, S. *et al.* PubChem 2019 update: improved access to chemical data. **47**, D1102–D1109 (2018).

6. Lipinski, C. A., Lombardo, F., Dominy, B. W. & Feeney, P. J. Experimental and computational approaches to estimate solubility and permeability in drug discovery and development settings. *Adv. Drug Deliv. Rev.* **23**, 3–25 (1997).

7. O'Boyle, N. M. *et al.* Open Babel: An open chemical toolbox. **3**, 33 (2011).

8. O'Boyle, N. M., Morley, C. & Hutchison, G. R. %J C. C. J. Pybel: a Python wrapper for the OpenBabel cheminformatics toolkit. **2**, 5 (2008).

9. Gaussian 09, Revision A.02, M. J. Frisch, G. W. Trucks, H. B. Schlegel, G. E. Scuseria, M. A. Robb, J. R. Cheeseman, G. Scalmani, V. Barone, G. A. Petersson, H. Nakatsuji, X. Li, M. Caricato, A. Marenich, J. Bloino, B. G. Janesko, R. Gomperts, B. Mennucci, H. P. Hratchian, J. V. Ortiz, A. F. Izmaylov, J. L. Sonnenberg, D. Williams-Young, F. Ding, F. Lipparini, F. Egidi, J. Goings, B. Peng, A. Petrone, T. Henderson, D. Ranasinghe, V. G. Zakrzewski, J. Gao, N. Rega, G. Zheng, W. Liang, M. Hada, M. Ehara, K. Toyota, R. Fukuda, J. Hasegawa, M. Ishida, T. Nakajima, Y. Honda, O. Kitao, H. Nakai, T. Vreven, K. Throssell, J. A. Montgomery, Jr., J. E. Peralta, F. Ogliaro, M. Bearpark, J. J. Heyd, E. Brothers, K. N. Kudin, V. N. Staroverov, T. Keith, R. Kobayashi, J. Normand, K. Raghavachari, A. Rendell, J. C. Burant, S. S. Iyengar, J. Tomasi, M. Cossi, J. M. Millam, M. Klene, C. Adamo, R. Cammi, J. W. Ochterski, R. L. Martin, K. Morokuma, O. Farkas, J. B. Foresman, and D. J. Fox, Gaussian, Inc., Wallingford CT, 2016.

10. The PyMOL Molecular Graphics System, Version 1.2r3pre, Schrödinger, LLC.

11. Bondi, A. van der Waals Volumes and Radii. *J. Phys. Chem.* **68**, 441–451 (1964).

12. Bradski, G. The OpenCV Library. *Dr. Dobbs Journal* (2000). Available at: https://www.drdobbs.com/open-source/the-opencv-library/184404319. (Accessed: 4th November 2019)

13. Lu, T. & Chen, F. Multiwfn: A multifunctional wavefunction analyzer. *J. Comput. Chem.* **33**, 580–592 (2012).

14. Pedregosa, F. *et al.* Scikit-learn: Machine learning in Python. **12**, 2825–2830 (2011).

15. Bertz, S. H. The first general index of molecular complexity. *J. Am. Chem. Soc.* **103**, 3599–3601 (1981).

16. Kromann, J. C., Steinmann, C. & Jensen, J. H. Improving solvation energy predictions using the SMD solvation method and semiempirical electronic structure methods. *J. Chem. Phys.* **149**, 104102 (2018).

17. RDKit: Open-Source Cheminformatics; http://www.rdkit.org.

18. Lusci, A., Pollastri, G. & Baldi, P. Deep Architectures and Deep Learning in Chemoinformatics: The Prediction of Aqueous Solubility for Drug-Like Molecules. *J. Chem. Inf. Model.* **53**, 1563–1575 (2013).

19. US EPA, Estimation Programs Interface Suite™ for Microsoft® Windows. (2012).

20. COSMOtherm, Release 19; COSMOlogic GmbH & Co. KG, http://www.cosmologic.de.

21. Eckert, F. & Klamt, A. Fast solvent screening via quantum chemistry: COSMO-RS approach. *AIChE J.* **48**, 369–385 (2002).

22. Klamt, A. The COSMO and COSMO-RS solvation models. *WIREs Comput. Mol. Sci.* **8**, e1338 (2018).

23. Klamt, A., Eckert, F., Hornig, M., Beck, M. E. & Bürger, T. Prediction of aqueous solubility of drugs and pesticides with COSMO-RS. *J. Comput. Chem.* **23**, 275–281 (2002).

24. Klamt, A., Jonas, V., Bürger, T. & Lohrenz, J. C. W. %J T. J. of P. C. A. Refinement and parametrization of COSMO-RS. *J. Phys. Chem. A* **102**, 5074–5085 (1998).

25. Sanghvi, T., Jain, N., Yang, G. & Yalkowsky, S. H. Estimation of aqueous solubility by the general solubility equation (GSE) the easy way. *QSAR Comb. Sci.* **22**, 258–262 (2003).

26. Llinas, A., Glen, R. C. & Goodman, J. M. Solubility challenge: can you predict solubilities of 32 molecules using a database of 100 reliable measurements? *J. Chem. Inf. Model.* **48**, 1289–1303 (2008).

27. Gaussian 16, Revision B.01, M. J. Frisch, G. W. Trucks, H. B. Schlegel, G. E. Scuseria, M. A. Robb, J. R. Cheeseman, G. Scalmani, V. Barone, G. A. Petersson, H. Nakatsuji, X. Li, M. Caricato, A. V. Marenich, J. Bloino, B. G. Janesko, R. Gomperts, B. Mennucci, H. P. Hratchian, J. V. Ortiz, A. F. Izmaylov, J. L. Sonnenberg, D. Williams-Young, F. Ding, F. Lipparini, F.

Egidi, J. Goings, B. Peng, A. Petrone, T. Henderson, D. Ranasinghe, V. G. Zakrzewski, J. Gao, N. Rega, G. Zheng, W. Liang, M. Hada, M. Ehara, K. Toyota, R. Fukuda, J. Hasegawa, M. Ishida, T. Nakajima, Y. Honda, O. Kitao, H. Nakai, T. Vreven, K. Throssell, J. A. Montgomery, Jr., J. E. Peralta, F. Ogliaro, M. J. Bearpark, J. J. Heyd, E. N. Brothers, K. N. Kudin, V. N. Staroverov, T. A. Keith, R. Kobayashi, J. Normand, K. Raghavachari, A. P. Rendell, J. C. Burant, S. S. Iyengar, J. Tomasi, M. Cossi, J. M. Millam, M. Klene, C. Adamo, R. Cammi, J. W. Ochterski, R. L. Martin, K. Morokuma, O. Farkas, J. B. Foresman, and D. J. Fox, Gaussian, Inc., Wallingford CT, 2016.
